# Assessment of 24-hour physical behaviour in adults via wearables: a systematic review of validation studies under laboratory conditions

**DOI:** 10.1186/s12966-023-01473-7

**Published:** 2023-06-08

**Authors:** Marco Giurgiu, Sascha Ketelhut, Claudia Kubica, Rebecca Nissen, Ann-Kathrin Doster, Maximiliane Thron, Irina Timm, Valeria Giurgiu, Claudio R. Nigg, Alexander Woll, Ulrich W. Ebner-Priemer, Johannes B.J. Bussmann

**Affiliations:** 1 Department of Sports and Sports Science, Karlsruhe Institute of Technology (KIT), Hertzstr. 16, 76187 Karlsruhe, Germany; 2 Department of Psychiatry and Psychotherapy, Medical Faculty Mannheim, Central Institute of Mental Health, Heidelberg University, Heidelberg, Germany; 3 Health Science Department, Institute of Sport Science, University of Bern, Bern, Switzerland; 4 Baden-Wuerttemberg Cooperative State University (DHBW), Karlsruhe, Germany; 5 Sport Pedagogy Department, Institute of Sport Science, University of Bern, Bern, Switzerland; 6 Erasmus MC, Department of Rehabilitation medicine, University Medical Center Rotterdam, Rotterdam, Netherlands

**Keywords:** Validation, Physical activity, Sleep, Sedentary behavior, Adults, Wearables

## Abstract

**Background:**

Wearable technology is used by consumers and researchers worldwide for continuous activity monitoring in daily life. Results of high-quality laboratory-based validation studies enable us to make a guided decision on which study to rely on and which device to use. However, reviews in adults that focus on the quality of existing laboratory studies are missing.

**Methods:**

We conducted a systematic review of wearable validation studies with adults. Eligibility criteria were: (i) study under laboratory conditions with humans (age ≥ 18 years); (ii) validated device outcome must belong to one dimension of the 24-hour physical behavior construct (i.e., intensity, posture/activity type, and biological state); (iii) study protocol must include a criterion measure; (iv) study had to be published in a peer-reviewed English language journal. Studies were identified via a systematic search in five electronic databases as well as back- and forward citation searches. The risk of bias was assessed based on the QUADAS-2 tool with eight signaling questions.

**Results:**

Out of 13,285 unique search results, 545 published articles between 1994 and 2022 were included. Most studies (73.8% (N = 420)) validated an intensity measure outcome such as energy expenditure; only 14% (N = 80) and 12.2% (N = 70) of studies validated biological state or posture/activity type outcomes, respectively. Most protocols validated wearables in healthy adults between 18 and 65 years. Most wearables were only validated once. Further, we identified six wearables (i.e., ActiGraph GT3X+, ActiGraph GT9X, Apple Watch 2, Axivity AX3, Fitbit Charge 2, Fitbit, and GENEActiv) that had been used to validate outcomes from all three dimensions, but none of them were consistently ranked with moderate to high validity. Risk of bias assessment resulted in 4.4% (N = 24) of all studies being classified as “low risk”, while 16.5% (N = 90) were classified as “some concerns” and 79.1% (N = 431) as “high risk”.

**Conclusion:**

Laboratory validation studies of wearables assessing physical behaviour in adults are characterized by low methodological quality, large variability in design, and a focus on intensity. Future research should more strongly aim at all components of the 24-hour physical behaviour construct, and strive for standardized protocols embedded in a validation framework.

**Supplementary Information:**

The online version contains supplementary material available at 10.1186/s12966-023-01473-7.

## Introduction

Accurate and reliable assessments of 24-hour physical behaviour are a prerequisite for researchers interested in characterizing behavior patterns over time, in different settings, and across different groups. The concept of 24-hour physical behaviour covers all time-use movement and non-movement behaviours (i.e., physical activity, sedentary behaviour, and sleep) of a person in his/her own environment [1]. Over the decades, diaries and questionnaires have been the primary tool for monitoring physical behaviour [2]. Even though these self-reported methods (sometimes referred to as subjective or indirect measures) are still widely applied today, they are prone to recall and social desirability bias [3]. In recent years, technology has evolved as a unique driving force behind advances in real-time data collection. Especially wearable technology has emerged as a popular means of monitoring behaviour-related metrics, overcoming limitations in self-report measures. However, we are not aware of a published systematic review focusing on the quality of laboratory validation protocols for assessing 24-hour physical behaviour in adults via wearables.

Wearables are defined as technology worn on or close to the body, assessing e.g., posture, acceleration, impact, biomechanical forces, heart rate, muscle oxygen saturation, or sleep patterns [4]. A wide range of garments, trackers, watches, bands, and smart patches equipped with multiple sensors exist to record a multitude of health and performance variables. The assessment opens avenues for better understanding and addressing individuals’ behaviours, and thus helps design appropriate interventions. Wearable-based measures of physical behaviour have become increasingly affordable and less obtrusive. Thus, apart from commercial purposes, they are up to date a valuable tool for promoting research in physical behaviour and health. In particular, there is a rising interest among researchers to capture the integrative cycle of 24-hour physical behaviour [5] via wearables that can collect dense data over a long period of time, allowing a detailed examination of daily behaviour. Due to the continued growth of the wearable market and the increasing interest in using wearables as monitoring tools in research, high-quality laboratory-based validation is highly warranted.

The plethora of wearables may seem like a blessing for behavioural researchers and epidemiologists, offering numerous devices for their daily work. However, it can also be a curse for both consumers and researchers to select the appropriate wearable or study design to obtain meaningful and transparent results. Several methodological issues regarding wearables, especially in research, should be addressed (e.g., data processing, monitoring protocols, or quality criteria such as validity [6]). Only a small proportion of wearables have been proven effective through rigorous, independent validation. In many cases, claims of these devices outweigh the evidence to support them [7]. For example, Peake et al. (2018) reported that only 5% of the 61 consumer wearables they reviewed in 2018 matched the marketing claims based on accepted reference standards [8].

Even though the body of research validating different wearables in both controlled laboratory and free-living environments is consistently growing, the published validation protocols are heterogeneous. In order to increase the comparability of validity measurements between different devices, standardized validation procedures and protocols are highly warranted [9–11]. Sperlich and Holmberg (2017) propose that wearables developed for health and fitness purposes should be controlled and monitored by independent scientific validation procedures [9, 10]. Recently, Keadle et al. (2019) introduced a five-step validation framework for wearables assessing physical behaviour [10]. The framework starts with the device manufacture and ends with its application in health studies. After initial mechanical (Phase 0) and calibration testing (Phase I), validation studies are recommended with structured and semi-structured assessment in the laboratory (Phase II) and real-world conditions (Phase III), where participants can perform their natural daily behaviours [10]. According to the authors, starting the development and validity assessment under laboratory conditions is essential, as external influences can be more easily controlled and manipulated than in studies in a free-living environment. Furthermore, comparisons to gold-standard measurements such as indirect calorimetry (intensity), video recordings (posture or activity type), or polysomnography (biological state) are easier to apply and can therefore serve as criteria.

Embedding standardized validation protocols into a framework [10, 11] is helpful for both consumers and researchers to select the appropriate wearable or study design and obtain meaningful and transparent results [12, 13]. Frameworks, in turn, can encourage innovation by manufacturers to achieve improved validity and transparency and inform practitioners before integrating wearables into daily clinical practice [11].

### Research purpose

This review focuses on the following three purposes: First, as our main purpose, we would like to raise researchers’ and consumers´ attention to the quality of published validation protocols while aiming to identify and compare specific consistencies/inconsistencies. Second, we would like to provide a comprehensive and historical overview of which wearable has been validated for which purpose, and third, whether they show promise or not for being used in further studies.

## Methods

This study followed the Preferred Reporting Items for Systematic Reviews and Meta-Analyses (PRISMA) reporting guidelines [14] and was registered in the PROSPERO international prospective register of systematic reviews, with registration number CRD42021252128 (see Additional file 1).

### Search strategy and study selection

To identify relevant publications, we used a search string that included terms for (a) validity, (b) type of wearable, and (c) outcomes of the 24-hour physical behaviour construct. An a priori pilot search was conducted to optimize the final term (see Additional file 2). Publications were searched from 1970 to December 2020 using the following databases: EBSCOHost, IEEE Xplore, PubMed, Scopus, and Web of Science. In June 2022, we ran search updates in all databases. Further, we reviewed the reference lists of included studies for publications that may have been missed in the database searches.

All articles were imported to a Reference Manager, Citavi library (Citavi 6.8, Swiss Academic Software GmbH, Swiss). After removing all duplicates first electronical and afterwards manually, the study selection process included three screening phases for eligibility. In the first phase, two reviewers (MG & RN) independently screened the titles of the publications. Articles were only excluded if both reviewers categorized an article as not eligible for review purposes. In the second phase, two reviewers independently screened the publications’ abstracts (MG & RN) to determine whether a full-text review was warranted. Disagreements among reviewers were resolved by consulting a third reviewer (AKD). In the third phase, the full texts of the remaining articles were assessed for eligibility by six members of the author’s team (MG, CK, RN, AKD, IT, and MT). Each article was screened independently by at least two reviewers. Discrepancies in screening were resolved by discussion until a consensus was reached. Reviewers were not blinded to author or journal information.

### Inclusion and exclusion criteria

Following the PICO principle [15], we included peer-reviewed, English-language publications that met the following criteria:

1. *Population*: Participants were adults ≥ 18 years regardless of health conditions.
2. *Intervention*: Any wearable validation study in which at least one part of the study was conducted under laboratory conditions with either standardized or semi-standardized protocols.
3. *Control/comparison*: Studies were only included if they described a criterion measure.
4. *Outcomes*: Studies were included in which the wearable outcome(s) could be classified into at least one dimension of the 24-hour physical behaviour construct (i.e., biological state, posture/activity type, or intensity [16], see Additional file 3).

### Data extraction

Two authors independently extracted data (MG, CK, RN, AKD, IT, VG or MT). Discrepancies were discussed until a consensus was reached. The following study details were extracted: author, year, location, population information (sample size, mean age of participants, percentage of females, ethnicity), measurement period, validated wearable (wearing position, software, epoch-length, algorithm/cut-point), dimension of the 24-hour physical behaviour construct, validated outcome, criterion measure, statistical analyses for validation purposes, study conclusion, and funding conflict of interest information.

### Data synthesis

Given the wide range of different study protocols in terms of varying conditions (e.g., wear location, measurement duration, sample size, statistical analyses, or criterion measure), we conducted a narrative synthesis based on the reported results/conclusions. In particular, we classified the studies as ↑ (i.e., moderate to strong validity), ↔ (i.e., mixed results), and ↓ (i.e., poor or weak validity). Each article was classified independently by at least two reviewers.

### Quality assessment

The risk of bias for each article was evaluated using the Quality Assessment of Diagnostic Accuracy Studies (QUADAS-2) tool [17]. The tool comprises four domains (i.e., patient selection, index measure, criterion measure, and flow/timing). Following the QUADAS-2 guidelines, we selected a set of signaling questions for each domain and added questions modified from the QUADAS-2 background document based on core principles, recommendations, and expert statements for validation studies [10, 11, 17, 18] (see Table 1). The risk of bias assessment was conducted independently by at least two authors. Discrepancies were discussed until a consensus was reached. The study quality was evaluated at the domain level, i.e., if all signaling questions for a domain were answered “yes”, then the risk of bias was deemed to be “low”. If any signaling question was answered “no”, then the risk of bias was deemed to be “high”. The “unclear” category was only used when insufficient data were reported for evaluation. Based on the domain-level ratings, we created a decision tree to evaluate the overall study quality as “low risk”, “some concerns” or “high risk” (see Additional file 4).

**Table 1:** The risk of bias assessment and the percentage of studies meeting these criteria

Criteria items		N studies meeting criterion			
		Total (N = 570)	Biological State (N = 80)	Posture/Activity Type (N = 70)	Intensity (N = 420)
**Domain 1**: Patient selection/study design					
**1.**	Did the study include a range of activities concerning the 24-hr physical behaviour construct? (i.e., activities from both areas: physical activity (e.g., walking/exercise) and sedentary activities (e.g., sitting, lying activities))^1^	210 (43%)	NA^2^	67 (96%)	143 (34%)
**2.**	Did the study protocol include at least one part/activity with natural transitions **(i.e., activities performed without fixed order of instructions)**?^1^	340 (69%)	NA^2^	67 (96%)	273 (65%)
**3.**	Did the study provide any information about the inclusion/exclusion of the recruiting process?	423 (74%)	62 (78%)	51 (73%)	310 (74%)
**4.**	Did the study include at least a sample of 20 participants?	440 (77%)	64 (80%)	43 (61%)	333 (79%)
**Domain 2**: Index measure					
**5.**	Was the algorithm of the validated outcome reported (i.e., formula), or was at least further information cited?	146 (26%)	35 (44%)	26 (37%)	85 (20%)
**Domain 3**: Criterion measure					
**6.**	Is the selected reference the gold standard?	432 (76%)	80 (100%)	41 (59%)	311 (74%)
**Domain 4**: Flow and timing					
**7.**	Did the authors provide any information about data synchronization?	223 (39%)	52 (65%)	34 (49%)	137 (33%)
**8.**	Were all participants included in the analyses or were any exclusion reasons provided?	474 (83%)	69 (86%)	59 (84%)	346 (82%)

^1^ Only relevant for N = 490 studies; ^2^ NA = Not applicable

## Results

**Fig. 1:**
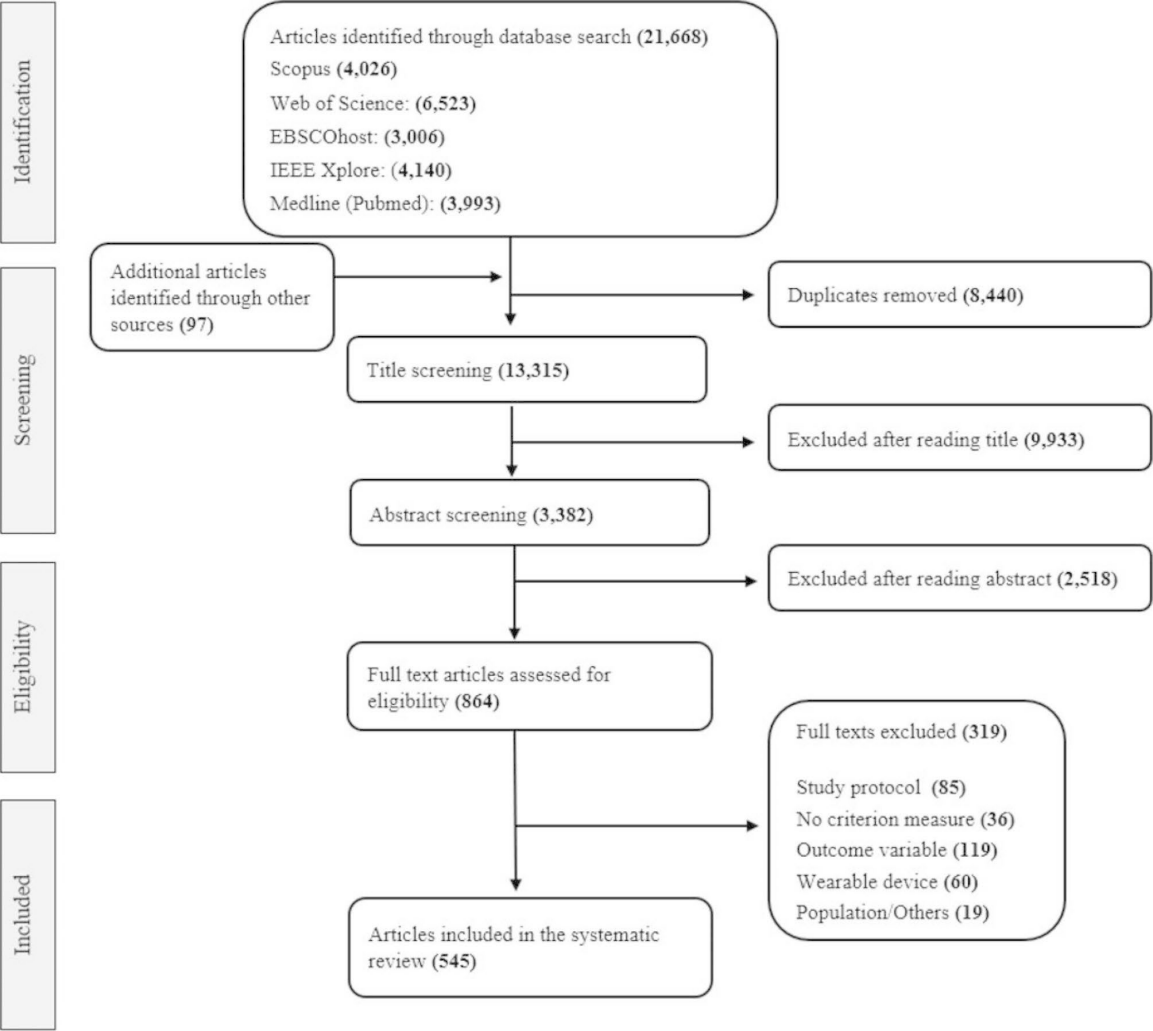
PRISMA flow chart illustrating literature search and screening process

Out of 13,285 records screened, 545 publications (see Fig. 1) were eligible for the current systematic review. Most studies investigated intensity (73.68%, N = 420), followed by biological state (14.04%, N = 80), and posture/activity type (12.28%, N = 70). The majority of the studies (95.41%, N = 520) validated only an outcome from one dimension, whereas 4.59% (N = 25) studies validated outcomes from the dimension’s intensity and posture/activity type.

### Participant and study characteristics

Of the studies included, 93.4% (N = 509) were conducted in high-income countries from North America, Europe, or Australia/Oceania, and most of them were published within the last decade (≥ 2011; 78.2%, N = 426; see Table 2). The sample size varied between 4 and 228 participants, with most studies (63.9%, N = 386) recruiting between 20 and 50 participants. In 86.2% (N = 470) of the studies, participants were between 18 and 65 years old. Ethnic background was reported in 7.5% (N = 41) of included studies. The majority of the studies reviewed included healthy participants (73.8%, N = 402), while 29.7% (N = 162) studies recruited samples with physical health disorders, such as stroke (N = 18), chronic obstructive pulmonary disease (N = 10), limb amputation (N = 10), or wheelchair users (N = 9). Besides the sleep protocols (14.9%, N = 81), most study protocols included either standardized tasks such as running/walking on a treadmill (42.2%, N = 230) or exercise tasks (3.5%, N = 19) or semi-standardized protocols, including activities of daily life (27.7%, N = 151) or walking/running tasks (33.2%, N = 181). The measurement duration for validation purposes varied between two minutes and 24 h. The majority of studies (90.5%, N = 493) conducted statistical analyses at the person/study level (e.g., t-tests, correlations, repeated measures ANOVA), 88 studies (16.1%) conducted both person/study-level analyses as well as epoch-by-epoch comparisons (e.g., accuracy, sensitivity, specificity). In 9.5% (N = 52) of all studies, the manufacturer was involved in study funding, loaned the devices or one of the authors declared a relation to the company of the validated wearable. In 56.5% (N = 308) of all studies, funding was independent of the manufacturer as well as authors declared no conflict of interest. In 15.8% (N = 86) of all studies, neither information about funding nor any information about conflict of interests were reported, whereas in the remaining 99 studies, at least funding information or conflict of interest statement was reported and without any relation to the manufacturer. Detailed information about the data extracted is reported in the supplement (see Additional file 5).

**Table 2:** Summary of data extraction: Participant and study characteristics

Category		Total (N = 545)	Biological State (N = 80)	Posture/Activity Type (N = 70)	Intensity (N = 420)
**Publication year**	≤ 1999	15	4	2	9
	2000–2010	104	17	8	82
	≥ 2011	426	59	60	329
**Study location** ^**a**^	Africa	1			1
	Asia	31	6	1	24
	Europe	191	17	45	145
	North America	268	43	18	214
	Australia/Oceania	50	14	6	32
	South America	4			4
**Number of participants [N]** ^**b**^	≤ 19	119	15	27	85
	20–50	348	46	35	282
	≥ 51	78	19	8	53
**Age [years, mean age]** ^**c**^	18–64	470	74	56	361
	≥ 65	72	6	13	57
**Sex [female %]** ^**d**^	0–25	57	11	11	37
	26–74	386	57	47	299
	75–100	69	9	10	55
**Protocol type**	Activities of daily life	151		56	114
	Exercise	19			19
	Others	100		18	88
	Sleep	81	80		1
	Treadmill	230		18	222
	Walking and running	181		17	170
**Criterion measure**	3-Dimensional Gait analysis	1			1
	Compendium	3			3
	Diary	2	1		1
	Direct calorimetry	1			1
	Indirect calorimetry	215		7	213
	Observation (direct)	131		22	112
	Observation (video)	138		41	111
	Polysomnography	79	79		
	Video-electroencephalography	1	1		
	Wearable	43		3	39
**Statistical analyses**	Epoch-by-epoch	105	54	31	28
	Person/study level	493	76	54	385

^a^ Three studies were not included in the summary statistics due to the lack of study location information; ^b^ One study was not included in the summary statistics due to the lack of sample size information; ^c^ Five studies were not included in the summary statistics due to the lack of age information; ^d^ Twenty-seven studies were not included in the summary statistics due to the lack of sex information

### Wearables

Of the 300 different wearables, 213 were classified as commercial-grade devices, 81 as research-grade devices, and six were not classified. We identified 129 different manufacturers with a range of one to 21 related models/series per manufacturer. For example, we identified 21 different models/series of Omron Healthcare Inc. and 18 different models/series of Garmin Ltd. Detailed technical information for each wearable is provided in a supplement (see Additional file 6). The most frequently validated devices in the studies included were the ActiGraph GT3X/GT3X+ (N = 95), the SenseWear Pro (N = 37), the ActivPAL (N = 32), and the Yamax Digiwalker SW-200 (N = 30). However, more than half of the 300 different devices were only validated once (55.4%; N = 166). Studies included one to seventeen different brands of wearables [19]. Most studies (52.3%, N = 285) included one wearable brand. Several studies (47.5%, N = 259) included multiple sensors or wearing positions (17.1%, N = 93) to enable comparison between different devices or wearing locations [20]. The hip/waist and wrist positions were most often used for validation purposes. Overall, ten different outcomes were validated (see Table 3). Any information about the software application used for data preprocessing was reported in 42.4% (N = 231) of all studies. Across all studies, the selected epoch length varied from 1 s to 2 min [21]. In 25.1% (N = 137) of all studies, some information about the used algorithm, equation, or cut-points was reported.

**Table 3:** Summary of data extraction: Wearables

Category		Total (N = 545)	Biological State (N = 80)	Posture/Activity Type (N = 70)	Intensity (N = 420)
**Outcome**	Sleep time	70	70		
	Sleep-wake metrics	11	11		
	Different postures/types	70		70	
	Time in SB	8			8
	Time in light **physical activity**	8			8
	Time in moderate-to-vigorous **physical activity**	13			13
	Time in walking/active	1			1
	Energy Expenditure	211			211
	Steps	235			235
	Counts	30			30
**Wear position** ^a, b^	Ankle	64		3	63
	Chest	50	4	11	38
	Ears	1			1
	Finger	2	2		0
	Foot	12		4	10
	Hip/waist	455	4	23	440
	Knee	1			1
	Leg	7		3	5
	Lower back	19		5	17
	Neck	7			7
	Pockets	34		4	32
	Shank	4			4
	Shoe	3			3
	Thigh	101		49	66
	Trunk	3		2	1
	Upper arm	94	5	3	88
	Wrist	477	99	26	361

^a^ Two studies did not report any information about the sensor wearing position

^b^ If studies included multiple devices or different wearing positions, we counted each wearing position

### Study quality

To evaluate the risk of bias in the validation studies, we used eight signaling questions (see Table 1). On average, 4.7 of 8 questions were answered with “yes” (i.e., meeting the criteria). Of studies validating a biological state, intensity, or posture/activity type as an outcome, on average, 4.5, 4.6, and 5.5 of 8 questions were answered yes. The percentage of meeting the criteria ranged from 20.0% (reported algorithm for intensity validation) to 100% (selecting gold reference standard in studies validating biological state) across activity dimensions. The majority of studies (76%, N = 432) reported a reference measure that was equivalent to the criterion measure [10]. The most frequently selected criterion measures were indirect calorimetry, observation (video or direct), and polysomnography (see Table 2). Overall, 4.4% (N = 24) of studies were classified as “low risk”, while 16.5% (N = 90) were classified as “some concerns” and 79.1% (N = 431) as “high risk”. The classification tree underlying the classification decisions can be found in the supplement (see Additional files 4 and 7).

### Validity

Across all studies (N = 545), we classified 1.269 validation results of 314 different wearables. In particular, we ranked 24% (N = 305) results/conclusions as “↑” (i.e., moderate to strong validity), 56% (N = 709) as “↔” (i.e., mixed validity), and 20% (N = 255) as “↓” (i.e., poor or weak validity). Additional file 8 provides an overview of each wearable, separated by different age groups. Of those 300 different wearables, 55.4% (N = 166) were validated once, 16.7% (N = 50) were validated in two different studies, 4.3% (N = 13) were validated in three different studies, and 23.7% (N = 71) were validated in more than three different studies.

Most wearables (N = 253) were used for the validation of only one dimension of the 24-hour physical behaviour construct. In particular, 216 wearables were used only for the validation of intensity outcomes, whereas 29 wearables for the validation of biological state outcomes, and eight wearables for the validation of posture/activity type. In contrast, we identified 23 wearables that validated both intensity and biological state outcomes, 17 wearables that validated both intensity and posture/activity type outcomes, and one wearable (i.e., USB accelerometer X16 mini) that validated biological state and posture/activity type outcomes. Moreover, six wearables (i.e., ActiGraph GT3X+, ActiGraph GT9X, Apple Watch 2, Axivity AX3, Fitbit Charge 2, and GENEActiv) had been validated for all three dimensions. None of those six wearables were ranked consistently as moderate to strong validity for measuring two or all three dimensions. We identified the ActiGraph GT3X+ (N = 95), SenseWear Pro (N = 37), ActivPAL (N = 32), Yamax Digiwalker SW-200 (N = 30), and two Fitbit models (Flex and One; each N = 28) as the most validated devices. Across all 95 studies, the ActiGraph GT3X and version GT3X + predominantly validated intensity outcomes (N = 90). Posture/activity type outcomes were validated in 17 studies, and biological states outcomes were validated in two studies. In two studies, the SenseWear Pro was validated for biological state outcomes, whereas in all other studies, the validated outcome belongs to the intensity dimension (N = 37). Studies validating the ActivPAL focused either on posture/activity type (N = 15) or intensity outcomes (N = 29). The Yamax Digiwalker SW-200 was solely validated for intensity outcomes and the Fitbit series One and Flex were tested for two of three dimensions (Intensity: N = 53; Biological state: N = 3).

## Discussion

### Summary of results

The main aim of this systematic review was to evaluate the characteristics, validity, and quality of laboratory validation studies among adults in which at least one dimension of the 24-hour physical behaviour construct [5, 16] was assessed via wearables and validated against a criterion measure. We identified the following four main results: First, the validation of biological state and posture/activity type outcomes was rare compared to intensity outcomes such as energy expenditure. Second, 253 of 300 different research and commercial-grade wearables were validated for only one aspect of the 24-hour physical behaviour construct. In particular, this review revealed that only six wearables (i.e., ActiGraph GT3X+, ActiGraph GT9X, Apple Watch 2, Axivity AX3, Fitbit Charge 2, and GENEActiv) were validated for all three dimensions. Third, none of those six wearables were ranked consistently as moderate to strong validity for measuring all three dimensions. However, single devices were extensively validated for one or two dimensions. For example, the Actiwatch series for the assessment of biological state, the ActivPAL for the assessment of posture/activity type, and the ActiGraph GT3X and GT3X + model for the assessment of intensity outcomes. Forth, only a few studies were ranked overall with “low risk” of bias or with “some concerns” based on selected criteria that align with published core principles, recommendations, and expert statements [10, 11, 17].

Therefore, one issue that emerges from the included studies is that no wearable provides valid results for all three dimensions in adults. However, the interpretation of validation study results strongly depends on the used protocols, which might vary as a function of different quality factors (e.g., criterion measures, sample size, measurement duration, statistical analyses, wearing position). Before critically evaluating our research approach in the limitation section, we want to summarize the consistencies/inconsistencies of the included studies as well as design features that have been proposed to enhance study quality.

### Criterion measure

When validating a device, the validity of the criterion measure to which the index device is being compared is of paramount importance [11]. If the criterion measure is invalid, then criterion standard bias may accrue [22]. Keadle et al. [10] recommend that physiological outcomes such as energy expenditure should be validated against indirect calorimetry. Step count or posture as behavioural criterion measures should be validated against video recordings with multiple observers (> 2) [11]. If the differentiation between sleep and wake patterns is the goal, polysomnography is the recommended criterion measure [23, 24].

In the included studies, a total of ten different criterion methods were identified. Fortunately, 76% (N = 432) of the studies used the respective gold standard. In the biological state dimension, all of the studies utilized the respective gold standard as a criterion measure. Although the majority of studies did use the recommended criterion measure (i.e., N = 215 indirect calorimetry, N = 138 video recording, and N = 79 polysomnography), 131 studies applied direct observation instead of video recording, which is prone to observation bias. Furthermore, 43 studies used wearables as a criterion measure. Since even research-grade wearable devices are susceptible to atypical gait [25, 26] and sensor wear position [25], using wearables may describe convergent validity rather than criterion validity.

Although using the gold standard, such as video recording, is undeniably challenging (e.g., low memory capacity), it is essential to apply gold standard comparisons. According to Johnston et al. (2021) alternative approaches should only be considered equivalent to the gold standard if they have been demonstrated to possess less than 5% measurement error specific to the population of interest [11]. In line with the selection of the appropriate criterion measure, researchers should take into account that no synchronization between index and criterion measures may introduce errors and bias the results. Timestamped or pragmatic solutions are recommended, such as participants performing three vertical jumps at the measurement’s beginning and end [10]. A critical aspect from the perspective of transparency is the presentation of algorithms.

### Wearing position

To assess all dimensions of the physical behaviour spectrum, the choice of an appropriate wear position of the device according to the research question is crucial [6]. Further, the wear position impacts the ability to detect transitions between specific activities and predict a spectrum of activities over a prolonged time (i.e., 1–2 days) [10]. In this review, 88% of the included studies analyzed wrist placement and 84% hip or waist placements. The recommended wear position depends on the dimension. In most of the included studies, a wrist placement was used to validate biological state outcomes. This is in line with published recommendations [24, 27, 28] indicating that wrist-worn devices enhance the detection of small movements occurring at the distal extremities in a supine position. Moreover, wrist-worn devices are likely to deliver higher compliance rates compared to hip placement in adults [29] and represent the most-used wearing position [11]. Most included studies analyzed a hip/waist position to validate intensity outcomes. The hip/waist position enables the device to be closer to the center of mass and thus captures gross muscle movements such as walking or running [30] and detects acceleration and deacceleration of the body [27]. Further, compared to waist placement, hip-worn accelerometers show a higher accuracy in predicting energy expenditure [31, 32]. However, the hip/waist wearing position increases the risk of misclassification of sitting/lying and standing postures, which is highly relevant to differentiate between physical activity and sedentary behaviour [33]. According to Stevens et al. [16], thigh-worn placements might be the most promising position to assess intensity and posture/activity types accurately. However, the number of validation studies using a thigh-worn device is underrepresented in our review, with no study validating thigh placement and biological state outcomes. To increase comparability between different accelerometer placements, brands, and types, future validation studies in adults are needed. Moreover, future signal analytical research purposes might be valuable in extracting and validating different outcomes from a single wearing position [34]. In general, we expect the fast-technological development of wearables to affect the future of physical behaviour data evaluation and processing. In particular, supervised learning approaches, such as machine learning or deep learning algorithms, are gaining popularity [35–37]. The inclusion of supervised learning approaches in health behaviour research has been slow, but this may change in the upcoming years [38].

### Study protocol

When performing a validation of a wearable monitor, a wide range of physical activities ranging from rest to vigorous exercise should be used during the validation procedures. Especially activities like lying, sitting, and standing, which most people spend the majority of the 24-hour day, should be included [18]. In this review, only 43% (N = 210) of the studies included a range of activities from the 24-hour physical behaviour continuum. Furthermore, 69% of the study protocols include at least one activity with natural transitions. Most study protocols applied either standardized tasks such as running/walking on a treadmill, exercise tasks, or semi-standardized protocols, including activities of daily life or walking/running tasks. The measurement duration varied between two minutes and 24 h.

For future research, we recommend extending laboratory validation protocols wherever possible to include different activities from the 24-hour physical behaviour cycle. Furthermore, activities with natural transitions should be included to better reflect typical behaviour patterns.

### Sample size, statistical analyses, and algorithms

Since we did not identify published recommendations about sufficient sample size for validation purposes, we chose 20 participants, a sample size that was achieved in most validation studies. However, an optimal solution for future research endeavors might be to conduct a priori sample size calculations and therefore ensure adequate power for validation purposes [11, 39]. For this purpose, researchers would need an effect size measure based on previous analyses. Ideally, recommendations on statistical procedures would gain consensus within the scientific community [40].

Within the reviewed studies, we identified a wide heterogeneity of conducted statistical analyses, ranging from traditional statistical tests on person/study level such as t-tests or ANOVA´s to epoch-by-epoch comparisons such as sensitivity, specificity, or accuracy. While traditional analyses may determine if differences exist between devices and the criterion measure, this does not necessarily imply that the two measures are statistically equivalent [40]. O´Brien [40] suggested that equivalence testing with standardized equivalence criteria could be a standard procedure for upcoming validation studies.

Only a low number of studies reported the formula or cited at least further information about the algorithm of the validated outcome. At this point, researchers often do not have access to the raw data of wearables and their “black-boxed” algorithms. More critically, several different approaches to transferring raw acceleration data into different units and metrics exist. Clevenger et al. (2022) summarized in a repository an extending overview of different analytic approaches [41]. Further, Clevenger et al. (2022) provided a first consensus method as a simple way to improve inter-study comparability [42]. Moreover, companies can update wearable’s firmware or algorithms anytime, hindering comparability [43, 44]. In addition, the pace at which technology is evolving for optimizing algorithms far exceeds the pace of published validation research [12]. We recommend that the upcoming series of wearables need an independent validation process or at least a replication of previous protocols. Furthermore, open-source methods that are more flexible to use algorithms for different devices are needed [10, 11].

### Limitations

Some limitations merit further discussion. First, the evaluation of the study quality was based on self-selected criteria. In particular, we selected the QUADAS-2 tool [17] and added further signaling questions in line with core principles, recommendations, and expert statements [10, 11, 18]. However, since we are not aware of any further quality tools and signaling questions that have been published for wearable validation purposes, our selected criteria can serve as a starting point for future reviews focusing on the study quality of wearable technology under laboratory conditions. Second, our review focused on the quality of study protocols. However, we did not account for further important considerations when using wearables such as wear/non-wear time algorithms, monitor cost, cut-points, reliability, or data processing time [10, 11, 45, 46]. Third, our included validation studies were published from 1994 to 2022. Given the rapid development of wearable technologies and the increasing availability of different research and commercial-grade devices, quality standards may have evolved. Thus, while interpreting the study protocols, the timing of the study realization should also be considered. Fourth, our findings are limited to our search strategy. Therefore, we may have missed some validation studies. However, we applied back- and forwards citation searches through reference lists of the included studies to screen articles that may not have appeared in our search. Fifth, this review was limited to articles published in English and may thus have excluded studies published in other languages. Sixth, we classified wearables as commercial or research-grade devices based on a self-selected approach (e.g., information on the manufacturer’s homepage).

### Future directions and conclusion

In line with our previous reviews [47–49] about the quality of validation studies, we identified a large number of different research and commercial-grade wearables that were validated under laboratory conditions. The quality of a validation study is a highly critical criterion to enable both researchers and consumers to make a guided decision about which studies to rely on and which device to use. To this end, our review unraveled that most validation studies did not meet recommended quality principles [11, 45]. There is a lack of validation studies that focused on biological state and posture/activity type outcomes. Moreover, most devices were validated only once. In contrast, a couple of devices were already extensively validated for at least two of three dimensions such as the ActiGraph GT3X and GT3X + or the ActivPAL. We anticipate that both existing and new devices will broaden their capabilities to capture the complete range of 24-hour physical behavior, possibly by incorporating algorithms for sleep detection. Thus, the next generation of validation studies might consider the validity of more than one aspect of the 24-hour physical behaviour construct during a study protocol or conduct a series of studies. We expect wearables to evolve as a global surveillance methodology for the 24-hour physical behaviour assessment [38, 50]. For this trend, scientific collaborations [51] are fundamentally necessary to bundle knowledge and harmonize the field of wearable devices, which is currently highly inconsistent [42, 52]. We finally conclude that standardized protocols for laboratory validation embedded in a framework [10] are urgently needed to inform and guide stakeholders (e.g., manufacturers, researchers, and consumers) in (i) selecting wearables for self-tracking purposes (ii) applying wearables in health studies and (iii) fostering innovation to achieve improved validity.

## Electronic supplementary material

Below is the link to the electronic supplementary material.

Additional file 1

Additional file 2

Additional file 3

Additional file 4

Additional file 5

Additional file 6

Additional file 7

Additional file 8

## Data Availability

Not applicable.

## Abbreviations

METs: Metabolic equivalents
