# Supplementary material for: Assessment of 24-hour physical behaviour in adults via wearables: a systematic review of validation studies under laboratory conditions
Source: Int J Behav Nutr Phys Act. 2023 Jun 8;20:68. doi: 10.1186/s12966-023-01473-7 (PMC10249261; doi:10.1186/s12966-023-01473-7)
Supplement: Supplementary file 2 — Additional file 2 [file 12966_2023_1473_MOESM2_ESM.docx]

**Additional file 2.** Search terms used in EbscoHost, IEEE Explore, PubMed, Scopus and Web of Science databases.

| **EbscoHost** | valid* OR reliab* OR accuracy OR precision OR specificity OR sensitivity OR reproduc* OR OR Feasibility OR criterion OR comparability OR “construct validity” OR calibration OR determination OR recognition  *AND*  acceleromet* OR “wearable device*” OR “wearable technology” OR “wearable tracker” OR “motion sensor” OR pedomet* OR inclinom* OR “actigraph*” OR “activity monitor*” OR “activity tracker*” OR “motion sensor” OR “motion sensing” OR “heart rate monitor*” OR “fitness tracker*”  *AND*  “physical activity” OR “physical fitness” OR walking OR exercis* OR “energy expenditure” “step-count” OR “locomotor activity” OR “ambulatory movement” OR “activity intensity” OR OR “human movement classification” OR “free-living condition” OR locomotion OR “activity patterns” OR sport OR “everyday activities” OR “free-living activites” OR Sedentar* OR sitting OR “sedentary behav*” OR “body postures” OR sleep OR “physical behav*” |
| --- | --- |
| **IEEE Explore** | valid* OR reliab* OR accuracy OR precision OR specificity OR sensitivity OR reproduc*  *AND*  acceleromet* OR “wearable” OR pedomet* OR inclinom* OR “activity monitor*” OR “heart rate monitor*” OR “fitness tracker*”  *AND*  “physical activity” OR exercis* OR “energy expenditure” “step-count” OR “free-living activites” OR sedentar* OR sleep |
| **Scopus** | (TITLE-ABS-KEY(valid* OR reliab* OR accuracy OR precision OR specificity OR sensitivity OR reproduc* OR feasibility OR criterion OR comparability OR calibration OR determination OR recognition) *AND* TITLE-ABS-KEY(acceleromet* OR “wearable device*” OR “wearable technology” OR “wearable tracker” OR “motion sensor” OR pedomet* OR inclinom* OR “actigraph*” OR “activity monitor*” OR “activity tracker*” OR “motion sensor” OR “motion sensing” OR “heart rate monitor*” OR “fitness tracker*”) *AND* TITLE-ABS-KEY(“physical activity” OR “physical fitness” OR walking OR exercis* OR “energy expenditure” “step-count” OR “locomotor activity” OR “ambulatory movement” OR “activity intensity” OR “free-living condition” OR locomotion OR “activity patterns” OR sport OR “everyday activities” OR “free-living activites” OR sedentar* OR sitting OR “sedentary behav*” OR “body postures” OR sleep OR “physical behav*”) |
| **PubMed** | ((valid*[Title/Abstract] OR reliab*[Title/Abstract] OR accuracy[Title/Abstract] OR precision[Title/Abstract] OR specificity[Title/Abstract] OR sensitivity[Title/Abstract] OR reproduc*[Title/Abstract] OR feasibility[Title/Abstract] OR criterion[Title/Abstract] OR comparability[Title/Abstract] OR calibration[Title/Abstract] OR determination[Title/Abstract] OR recognition[Title/Abstract]) *AND* (acceleromet*[Title/Abstract] OR “wearable device*”[Title/Abstract] OR “wearable technology”[Title/Abstract] OR “wearable tracker”[Title/Abstract] OR “motion sensor”[Title/Abstract] OR pedomet*[Title/Abstract] OR inclinom*[Title/Abstract] OR “actigraph*”[Title/Abstract] OR “activity monitor*”[Title/Abstract] OR “activity tracker*”[Title/Abstract] OR “motion sensor”[Title/Abstract] OR “motion sensing”[Title/Abstract] OR “heart rate monitor*”[Title/Abstract] OR “fitness tracker*”[Title/Abstract])) *AND* (“physical activity”[Title/Abstract] OR “physical fitness”[Title/Abstract] OR walking[Title/Abstract] OR exercis*[Title/Abstract] OR “energy expenditure” “step-count”[Title/Abstract] OR “locomotor activity”[Title/Abstract] OR “ambulatory movement”[Title/Abstract] OR “activity intensity”[Title/Abstract] OR “free-living condition”[Title/Abstract] OR locomotion[Title/Abstract] OR “activity patterns”[Title/Abstract] OR sport[Title/Abstract] OR “everyday activities”[Title/Abstract] OR “free-living activites”[Title/Abstract] OR sedentar*[Title/Abstract] OR sitting[Title/Abstract] OR “sedentary behav*”[Title/Abstract] OR “body postures”[Title/Abstract] OR sleep[Title/Abstract] OR “physical behav*”[Title/Abstract]) |
| **Web of Science** | (((((TS=(Valid* OR reliab* OR accuracy OR precision OR specificity OR sensitivity OR reproduc* OR feasibility OR criterion OR comparability OR calibration OR determination OR recognition) AND TS=( acceleromet* OR “wearable device*” OR “wearable technology” OR “wearable tracker” OR “motion sensor” OR pedomet* OR inclinom* OR “actigraph*” OR “activity monitor*” OR “activity tracker*” OR “motion sensor” OR “motion sensing” OR “heart rate monitor*” OR “fitness tracker*”) AND TS=( “physical activity” OR “physical fitness” OR walking OR exercis* OR “energy expenditure” “step-count” OR “locomotor activity” OR “ambulatory movement” OR “activity intensity” OR “free-living condition” OR locomotion OR “activity patterns” OR sport OR “everyday activities” OR “free-living activites” OR sedentar* OR sitting OR “sedentary behav*” OR “body postures” OR sleep OR “physical behav*”) NOT KP=valid*) NOT KP=reliab*)NOT KP=recog*) NOT KP=feasib*) NOT KP=sensitiv*) |
