# Supplementary material for: Assessment of 24-hour physical behaviour in adults via wearables: a systematic review of validation studies under laboratory conditions
Source: Int J Behav Nutr Phys Act. 2023 Jun 8;20:68. doi: 10.1186/s12966-023-01473-7 (PMC10249261; doi:10.1186/s12966-023-01473-7)
Supplement: Supplementary file 3 — Additional file 3 [file 12966_2023_1473_MOESM3_ESM.docx]

**Additional file 3**. Characteristics of 24-hour physical behaviour.

| **Behavior** | **SLEEP** | **PHYSICAL ACTIVITY** | **SEDENTARY BEHAVIOUR** |
| --- | --- | --- | --- |
| **Definition** | A naturally recurring and easily reversible state that is characterized by reduced or absent consciousness, perceptual disengagement, immobility, and the adoption of a characteristic sleeping posture | Any voluntary movement produced by skeletal muscles that results in energy expenditure | Any waking behavior characterized by an energy expenditure of 1.5 metabolic equivalents (METs; 1 MET = energy expenditure in rest), while in a sitting, reclining, or lying posture |
| **Intensity category** (Individual is sedentary or conducting LPA or MVPA – e.g. differentiated by MET-thresholds) | Sedentary (e.g., ~ 1 MET) | LPA, MVPA (e.g., ≥ 1 MET) | Sedentary (≤ 1.5 MET) |
| **Body Posture and/or movement** (postural location – e.g., differentiated by specific activity types) | Sitting, reclining, lying | Standing, cycling, walking running, wheelchair driving | Sitting, reclining, lying |
| **Activity type** | e.g., sleep at night, nap | e.g. vacuuming, gardening, walking | e.g. desk work, watching TV, driving a car |
| **Biological State** (condition asleep or awake) | Asleep | Awake | Awake |
| **Domain** | At home, not at home | Work, home, leisure, transportation | Work, home, leisure, transportation |
| **Bout Length** | Short, moderate, long | Short, moderate, long | Short, moderate, long |
| **Outcomes (examples)** | Sleep time, sleep-wake metrics, time awake | Energy expenditure, steps, time spent in different intensity categories, Time spent in standing or walking | Sedentary time, number of sedentary bouts |
