# Supplementary figures and images for: Assessment of 24-hour physical behaviour in adults via wearables: a systematic review of validation studies under laboratory conditions
Source: Int J Behav Nutr Phys Act. 2023 Jun 8;20:68. doi: 10.1186/s12966-023-01473-7 (PMC10249261; doi:10.1186/s12966-023-01473-7)

**Additional file 4.** Risk of bias: Classification tree for the judgment of overall study quality.


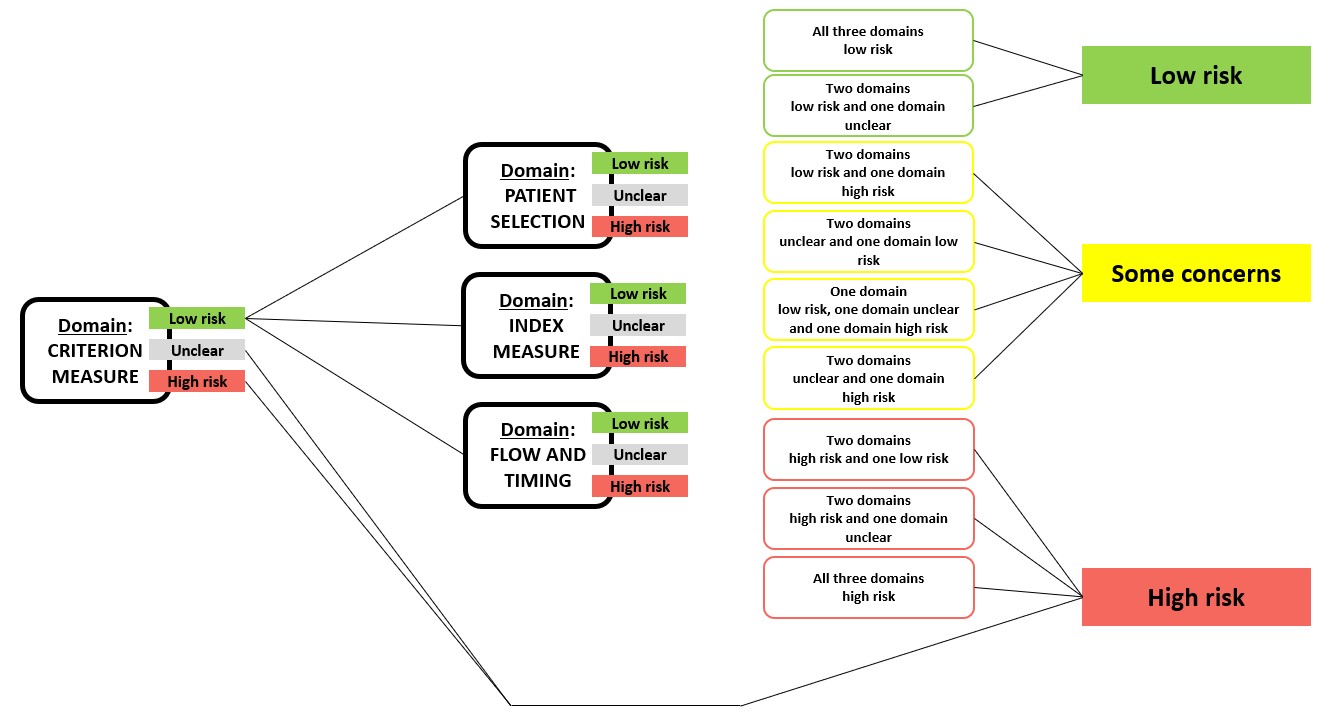

Supplement: Supplementary file 4 — Additional file 4 [file 12966_2023_1473_MOESM4_ESM.docx]
