# Supplementary material for: Assessment of 24-hour physical behaviour in adults via wearables: a systematic review of validation studies under laboratory conditions
Source: Int J Behav Nutr Phys Act. 2023 Jun 8;20:68. doi: 10.1186/s12966-023-01473-7 (PMC10249261; doi:10.1186/s12966-023-01473-7)
Supplement: Supplementary file 5 — Additional file 5 [file 12966_2023_1473_MOESM5_ESM.docx]

**Additional file 5**. Data Extraction.

| **No.** | **Author (Year, location)** | **Study population (N; mean age±SD or range [yrs]; % females; ethnicity)** | **Study Protocol (measurement duration)** | **Wearable (placement, software, epoch-length, algorithm)** | **Dimension (Outcome(s))** | **Criterion measures** | **Statistical analysis** | **Study conclusion** | **Funding/ Conflict of interest** | **Overall risk of bias (low, some, high)** |
| --- | --- | --- | --- | --- | --- | --- | --- | --- | --- | --- |
| 1 | Abel et al. (2008, United States) | Adults (N=20; 29.4±7.1 yrs; 50% females; NR) | Treadmill (60 minutes) | *ActiGraph GT1M* (waist; NR; 1 minute; Freedson equation); *Kenz Lifecorder EX* (waist; NR; 4 sec; NR) | Intensity (Energy expenditure; Steps) | Indirect Calorimetry; observation (Video) | Mixed ANOVA; Pearson correlation | The Freedson equation inaccurately measured total EE at most walking and running speeds. The KL and the AG are moderately priced accelerometers that provide researchers and clinicians with accurate estimates of step counts and activity EE at most walking and running speeds. | NR^1^/NR | High |
| 2 | Albaum et al. (2019, Canada) | Adults with subacute motor incomplete spinal cord injury (N=17; Median 62.0 yrs; 24% females; NR) | Activities of daily life (< 45 minutes) | *ActiGraph wGT3x-BT* (ankle of least affected leg; ActiLife v6.13.3; 1 sec; NR) | Intensity (Steps) | Observation (Manually-counted) | Intraclass correlation coefficient; Bland-Altman analyses; Paired t-test; Wilcoxon test | The Actigraph wGT3x-BT accurately counts steps during PT sessions and walking periods in individuals with subacute motor iSCI. | N^2^/N | High |
| 3 | Alberto et al. (2017, United States) | Adults (N=16; 25.38±8.58 yrs; 50% females; NR) | Treadmill and activities of daily life (NR) | *ActiGraph GT3X+* (right hip; NR; 1 minute; Freedson equation); *ActivPAL* (right thigh; NR; 1 minute; MET*h/60); *SenseWear 2* (left upper arm; NR; 1 minute; proprietary algorithm) | Intensity (Energy expenditure (MET)) | Indirect Calorimetry | Mean percentage error; Equivalency testing; Bland-Altman analyses; Kappa statistics; Sensitivity; Specificity | None of the WMs tested in this study were equivalent with the criterion measure (VO2) in estimating sedentary-to-light activities; however, the activPAL had greater overall accuracy in measuring SB and LPA than did the ActiGraph and SenseWear 2 monitors. | N/N | High |
| 4 | Ali et al. (2018; United States) | Adults (N=20; 26±3 yrs; 85% females; NR) | Treadmill (15 minutes) | *Fitbit Flex* (wrist; NR; NR; NR); *StepWatch* (ankle; NR; NR; NR) | Intensity (Steps) | Observation (Video) | Bland-Altman analyses; Intraclass correlation coefficient | WWAMs recorded steps consistently between-sessions and between-devices for treadmillwalking among healthy adults at each speed but exhibited limited agreement for recording steps at each speed compared to AWAMand video. | NR/N | High |
| 5 | Alinia et al. (2017, United States) | Adults (N=15; 21-31 yrs; 47% females; NR) | Treadmill and activities of daily life (30 minutes) | *Fitbit Zip* (chest, pants pocket, wrist; NR; NR; NR); *Fitbit One* (chest, pants pocket, wrist; NR; NR; NR); *Fitbit Flex* (chest, pants pocket, wrist; NR; NR; NR) | Intensity (Steps) | Observation (Video) | One-way ANOVA; Error rate; Intraclass correlation coefficient | This feasibility study focused on 6 PAs and demonstrated that Fitbit trackers were most accurate when walking on a treadmill and least accurate during walking with a walking aid and for low-intensity activities. | NR/N | High |
| 6 | Almeida et al. (2015, United States) | Adults after total knee arthroplasty (N=21; 68±7 yrs; 67% females; NR) | Treadmill and activities of daily life (80 minutes) | *ActiGraph GT1M* (waist; ActiLife 4 software; 1 minute; work-energy theorem and Freedson equation); *Sensewear Pro3 Armband* (upper arm; InnerView Professional Research software v6.1; NR; proprietary algorithm) | Intensity (Energy expenditure) | Indirect Calorimetry | Paired t test; Intraclass correlation coefficient; Bland-Altman analyses | The multisensor-based monitor showed better criterion-related validity than the accelerometer-based monitor and should be considered as a tool to measure physical activity in individuals after TKA. | N/NR | Low |
| 7 | Alsubheen et al. (2016, Canada) | Adults (N=13; 40±11.9 yrs; 38% females; NR) | Activities of daily life, walking, and treadmill (90 minutes) | *Garmin vivofit* (wrist, Garmin Connect; NR; NR) | Intensity (Energy expenditure; Steps) | Indirect Calorimetry; Observation (Video) | Paired t-tests; Repeated-measures ANOVA; Linear regressions | The vivofit significantly underestimated EE for treadmill walking, but responded to the differences in the inclination. Vivofit underestimated step count for level walking but provided an accurate estimate for incline walking. | N/N | High |
| 8 | Ameen et al. (2019, Austria) | Adults (N=19; 29±13 yrs; 68% females; NR) | Sleep (1 night) | *Mi Band 2* and *Mi Band 3* (wrist, Mi Fit software v1.1.14; 30 sec; NR); *MotionWatch 8* (wrist, MotionWare software v1.1.20; 30 sec; NR) | Biological state (Total sleep time) | Polysomnography | Spearman correlation; Bland-Altman analyses; Sensitivity; Positive predictive value; Kappa statistics | Results suggest that, to date, the available sleep trackers do not provide meaningful sleep analysis but may be interesting for simply tracking time in bed. | N/N | High |
| 9 | Aminian et al. (1999, Switzerland) | Adults (N=5; NR; 20% females; NR) | Activities of daily life (60 minutes) | *Physilog* (chest & thigh; NR; NR; physical-activity detection algorithm) | Posture/Activity type (lying, sitting, standing, dynamic, others) | Observation (Video) | Predicted error; Sensitivity; Missclassification error | Physilog can be used in the clinical setting for the reliable measurement and long-term recording of most usual physical activities. | NR/NR | Some |
| 10 | An et al. (2017, United States) | Adults (N=62; males: 25.1*±*5.6 yrs; females: 22.4*±*4.1 yrs; 40% females; NR) | Activities of daily life and treadmill (75 minutes) | *ActiGraph GT3X+* (wrist, waist; ActiLife v6.5.3; 1 sec; NR); *ActivPAL* (right thigh; activPAL Research Edition v6.4.1; 15 sec; NR) | Posture/Activity type (sitting, standing, stepping) | Observation (Direct) | Mean absolute percent error; Cohen’s D; Equivalence testing | The AP was reasonably accurate for detecting sitting, standing, and stepping, and the AG was very accurate for classifying stepping when the stepping activity was determined by the formula created by 0.7 step/s threshold. | Y^3^/Y | High |
| 11 | An et al. (2017, United States) | Adults (N=35; 31.0±11.8 yrs; 51% females; NR) | Treadmill (18 minutes) and indoor walking (600 meter) | *SenseWear Armband Mini* (non-dominant upper arm; SenseWear 8.1.; NR; NR); *Basis B1 Band* (right wrist; Basis App; NR; NR); *Withings Pulse* (waist, Health Mate App; NR; NR); *Misfit Shine* (wrist; Misfit App; NR; NR); *Fitbit Flex* (left wrist; Fitbit App; NR; NR); *Fitbit Zip* (waist, Fitbit App; NR; NR); *Garmin vivofit* (wrist, Garmin Connect App; NR; NR); *Jawbone UP24* (right wrist; UP App; NR; NR); *Nike + FuelBand* (left wrist; Nike + Fuel App; NR; NR); *Polar Loop* (wrist; PolarFlow App; NR; NR); *New Lifestyle NL-1000* (waist; NR; NR; NR) | Intensity (Steps) | Observation (Manually-counted) | Mean absolute percentage error; Pearson correlation; Bland-Altman analyses; ANOVA; Equivalence testing | Results show that the Fitbit Zip and Withings Pulse provided the most accurate measures of step count under all three different conditions (i.e. treadmill, over-ground, and 24-hour condition), and considerable variability in accuracy across monitors and also by speeds and conditions. | NR/N | High |
| 12 | Anastasopoulou et al. (2014; Germany | Adults (N=19; 30.4±9.0 yrs; 42% females; NR) | Posture and walking (75 minutes) | *ActiGraph GT3X* (waist; Actilife 5; 1 sec; vector magnitude algorithm); *Move II* (waist; DataAnalyzer; 1 sec; NR) | Intensity (Activity energy expenditure) | Indirect Calorimetry | Mean difference; Bland-Altman analyses | Data suggest that the activity monitor using activity-dependent calculation models is more appropriate for predicting AEE in daily life than the activity monitor using a single regression model. | N/N | Some |
| 13 | Anderson et al. (2019, United Kingdom) | Ward based hospitalised adults recovering from critical illness (N=20; 62.3±11.5 yrs; 35% females; NR) | Indoor walking (NR) | *ActiGraph GT3X* (non-dominant thigh, non-dominant ankle; NR; NR; NR) | Intensity (Steps) | Observation (Manually-counted) | Bland-Altman analyses; Mean difference; Absolute percentage error; Intraclass correlation coefficient | Step count quantified by an ankle mounted Actigraph GT3X accelerometer demonstrates concordance with observed step count within ward-based adults recovering from critical illness. | N/N | High |
| 14 | Andersson et al. (2014, Sweden) | Adults with chronic obstructive pulmonary disease (N=15; 59-69 yrs; 53% females; NR) | Activities of daily life (53 minutes) | *DynaPort ADL* (waist; manufacturer’s website; NR; NR); *DynaPort MiniMod* (waist; manufacturer’s website; NR; NR); *SenseWear Armband Pro 3* (right upper arm; Inner View PC software v6.1; NR; NR) | Intensity (Steps); Posture/activity type (sitting, lying, standing) | Observation (Video) | Bland Altman analyses; Friedman ANOVA; Wilcoxon test; Intraclass correlation coefficient | Activity monitors are not equivalent in their abilities to detect steps or body positions. | N/N | High/High |
| 15 | Anens et al. (2021, Sweden) | Adults with multiple sclerosis (N=30; 49.2±14 yrs; 70% females; NR) | Activities of daily life and walking (35 minutes) | *PiezoRX* (waist; NR; NR; NR); *Yamax SW200* (waist; NR; NR; NR); *ActiGraph GT9X Link* (waist; ActiLife v6; 1 sec; Freedson VM3) | Intensity (Steps); Posture/Activity type (sitting, supine, lying) | Observation (Video) | Percentage error; Median absolute percentage error; Spearman correlation; Freidman ANOVA; Bland-Altman analyses | AGlink and PiezoRX were valid measures of steps in persons with multiple sclerosis. | N/N | Some/High |
| 16 | Annegarn et al. (2011, Netherlands) | Healthy adults (N=5; 23.8±3.6 yrs; 40% females; NR), Adults with chronic heart failure patients (N=5; 54.2±16.8 yrs; 20% females; NR); Adults with chronic obstructive pulmonary disease patients (N=5; 60.0±9.9 yrs; 60% females; NR) | Activities of daily life and walking (60 minutes) | *CAM* (leg, trunk; NR; NR; signal magnitude area) | Posture/Activity type (Dynamic activities, weight-bearing and non-weight-bearing postures) | Observation (Video) | Means of the generalized linear mixed models; Bland-Altman analyses; Kappa statistics | The CAM is a promising single-sensor unobtrusive tool for providing accurate data on the type and duration of daily activities in the home environment of patients with chronic organ failure. | N/N | High |
| 17 | Arch et al. (2018, United States) | Adult prosthetic users with a unilateral transtibial amputation (N=50; 56± 10 yrs; 27% females; NR) | Indoor walking (6 minutes) | *StepWatch* (prosthetic pylon; NR; NR; NR); *Fitbit One* (prosthetic pylon; NR; NR; proprietary algorithm) | Intensity (Steps) | Observation (Manually-counted) | Absolute percentage errors; Intraclass correlation coefficient; Bland-Altman analyses | Both monitors accurately counted steps during forward linear walking. StepWatch appears to be more accurate than FitBit during complex walking but a larger sample size may confirm these findings. FitBit consistently counted fewer steps than StepWatch during free-living walking. | N/N | High |
| 18 | Ayabe et al. (2008, Japan) | Adults (N=10; 24±3 yrs; 0% females; NR) | Stairs climbing and bench stepping exercise (NR) | *Yamax DW-800* (waist; NR; NR; NR); *Kenz* *Lifecorder* (waist; NR; NR; NR); *Omron HJ701IT* (waist, NR; NR; NR) | Intensity (Steps) | Observation (Video) | Paired t-tests; Bland-Altman analyses | Results indicate that the KZ and the YM can accurately assess the number of steps during stair climbing using 20 to 30 cm high platforms at 80 to 120 steps·min^-1^. | NR/NR | Some |
| 19 | Backhouse et al. (2013, United Kingdom) | Adults with rheumatoid arthritis (N=12; 51.6±18.0 yrs; 83.3% females; NR); Healthy adults (N=12; 41.6±9.8 yrs; 50% females; NR) | Indoor walking (2 minutes) | *Step-N-Tune* (waist; NR; NR; NR); *Activ4Life Pro V3.8* (hip; NR; NR; proprietary onboard algorithms); *Intelligent Device for Energy Expenditure and Activity* (two at feet, two at thigh, one at chest; NR; NR; NR) | Intensity (Steps) | Observation (Video) | Bland-Altman analyses | Despite some variation between devices, all the activity monitors tested performed reasonably well in healthy young volunteers. All except the Activ4Life showed a marked decrease in performance in patients with rheumatoid arthritis, suggesting Activ4Life could be the most suitable for use in this patient group. | N/N | High |
| 20 | Bai et al. (2016, United States) | Adults (N=52; 18–60 yrs; 46.2% females, NR) | Sedentary, treadmill and resistance exercise (70 minutes) | *ActiGraph GT3X+* (waist; NR; 1 minute; Freedson´s 98 regression, Work–Energy Theorem); *Sense Wear* (non-dominant arm; NR; NR; Mifflin–St. Jeor method); *Fitbit Flex* (left wrist; NR; NR; Mifflin–St. Jeor method); *Jawbone Up24* (right wirst; NR; NR; Mifflin–St. Jeor method); *Misfit Shine* (left wrist; NR; NR; Mifflin–St. Jeor method); *Nike+ Fuelband SE* (right wrist; NR; NR; Mifflin–St. Jeor method); *Polar Loop* (left wrist; NR; NR; Mifflin–St. Jeor method) | Intensity (Energy expenditure) | Indirect Calorimetry | Pearson correlation; Mean absolute percent error; Bland-Altman analyses | Overall, the research monitors and Fitbit Flex, Jawbone Up24, and NFS provided reasonably accurate total EE estimates at the individual level. However, larger error was evident for individual activities, especially resistance exercise. | N/N | High |
| 21 | Bai et al. (2018, United States) | Adults (N=39; 32.0±11.0 yrs; 41% females; NR) | Activities of daily life and treadmill (80 minutes) | *Apple Watch 1* (left wrist; NR; NR; NR); *Fitbit Charge HR* (left wrist; NR; NR; NR) | Intensity (Energy expenditure; Steps) | Indirect Calorimetry; Wearable (Yamax SW-200 DigiWalker (waist)) | Pearson correlation; Bland-Altman analyses; Mean absolute percent errors; Root-mean-square errors; Equivalence testing | The Apple Watch 1 had stronger validity than the Fitbit Charge HR for assessing overall EE and steps during aerobic exercise. The Fitbit Charge HR provided heart rate estimates that were statistically equivalent to Polar monitor. | N/N | Low |
| 22 | Bajaj et al. (2018, Australia) | Adults with visual impairment (N=32; 73±9 yrs; 62% females; NR) | Walking (4 minutes) | *ActiGraph GT3X-BT* (ankle, hip, wrist; ActiLife v6.13.3; 1 sec; NR) | Intensity (Steps) | Observation (Manually-counted) | Bland-Altman analyses; Accuracy; Linear regression | Results demonstrate that the most accurate location of activity monitor placement is the ankle and that when using the low-frequency filter the level of agreement becomes more acceptable on the wrist and hip, in this population. | N/N | High |
| 23 | Balogun et al. (1989, United States) | Adults (N=25; 24.7±5.4 yrs; 60% females; NR) | Treadmill (32 minutes) | *Caltrac accelerometer* (waist; NR; NR; NR) | Intensity (Counts) | Indirect Calorimetry | Pearson product-moment correlation; Paired t-test; ANOVA | The results of the analyses revealed that the Caltrac accelerometer output is a valid predictor of Ee during level walking when the appropriate regression equation is used to adjust the values. Because the accelerometer device tends to overestimate Ee, the raw accelerometer readings should be applied with caution | N/NR | High |
| 24 | Balto et al. (2016, United States) | Adults with multiple sclerorsis (N=45; 46.7±10 yrs; NR; NR) | Treadmill (1000 steps) | *Yamax Digiwalker SW-200* (non-dominant hip; NR; NR; NR); *Jawbone UP2* (non-dominant wrist; Jawbone UP v.4.4.1.; NR; NR); *Jawbone UP Move* (non-dominant hip; Jawbone Move v.4.4.1; NR; NR); *Fitbit Flex* (non-dominant wrist; Fitbit v.2.10; NR; NR); *Fitbit One* (non-dominant hip; Fitbit v.2.10; NR; NR) | Intensity (Steps) | Observation (Manually-counted) | Mean confidence interval; 95% confidence interval; Mean percentage error; Coefficient of variation; Spearman correlations | The results suggest that the waist-worn Fitbit One is the most precise and accurate sensor for measuring steps when walking on a treadmill. | N/N | High |
| 25 | Bania (2014, Australia) | Adults with diplegic cerebral palsy (N=10; 18.6±2.7 yrs; 40% females; NR) | Postures (6 minutes); Walking (6 minutes) | *ActivPAL* (thigh; NR; NR; NR) | Intensity (Steps); Posture/ Activity Type (Sitting; standing) | Observation (Video) | Bland-Altman analyses; Coefficient of determination; Paired t-tests | The results support the criterion validity and the retest reliability of the ActivPAL to measure physical activity and sedentary behaviour in groups of young people with diplegic CP but not in individuals. | N/NR | High/High |
| 26 | Barkley et al. (2019, United States) | Adults (N=23; 21.9±1.6 yrs; 70% females; NR) | Treadmill (20 minutes) | *Movband 2* (non-dominant wrist; NR; NR; proprietary algorithm) | Intensity (Counts) | Wearable (Actigraph GT1M (waist)); Indirect Calorimetry | Pearson’s correlation analyses; one sample t-test; Bland-Altman analyses; linear regression | The low-cost, Movband accelerometer appears to provide a valid assessment of physical activity behavior/intensity. | N/N | High |
| 27 | Barouni et al. (2020, Germany) | Adults with and without sleep disorders (N=98; 28.5 yrs; 43.9% females; NR) | Sleep (1 night) | *Move3* (wrist, chest; NR; 30 sec; algorithm reported) | Biological state (Sleep-wake metric; Non-wear time) | Polysomnography | Chi-square test; Mann-Whitney U Test; Kappa statistics; Bland-Altman analyses; Pearson correlation; Sensitivity, Specificity; positive predictive value; negative predictive value; Informedness; accuracy; confusion matrix | Based on the Bland-Altman analysis, the chest-worn accelerometer showed better results than the wrist-worn accelerometer. | NR/Y | High |
| 28 | Bartholdy et al. (2018, Denmark) | Adults with knee osteoarthritis (N=24; 67.3±6.5 yrs; 75% females; NR) | Postures (6 minutes); Treadmill (>2 minutes); outdoor walking (5 minutes); stairs (20 seconds); ergometer (2 minutes) | *SENS motion system* (thigh; smartphone application; 10 sec; algorithm reported) | Posture/Activity type (Sedentary; walking; standing; other activities) | Observation (Direct) | Percentage of agreement | The SENS motion activity measurement system can be regarded as a reliable and valid device for measuring sedentary behaviour in patients with knee OA, whereas detection of walking is not reliable and would require further work. | NR/Y | High |
| 29 | Bassett et al. (2000, United States) | Adults (N=81; 40±15 yrs; 53% females; 3% Asian, 19% African America, 1% Hispanic, 77% Caucasian) | Activities of daily life (NR); Treadmill (NR); Exercise (NR) | *CSA model 7164 accelerometer* (waist; NR; 1 minutes; regression equations); *Caltrac* (waist; NR; 2 minutes; NR); *Kenz select 2* (waist; NR; NR; NR); *Yamax SW-701* (waist; NR; NR; NR) | Intensity (Energy expenditure) | Indirect Calorimetry | One-way ANOVA with repeated measures; t-tests one-sample t-tests; Bland-Altman plots; Pearson correlation | Motion sensors tended to overpredict EE during walking. However, they underpredicted the energy cost of many other activities because of an inability to detect arm movements and external work. | N/N | Low |
| 30 | Bassett et al. (2014, United States) | Adults (N=15; 25±9.4 yrs; NR; NR) | Postures (15 minutes); Activities of daily life (3 minutes); Treadmill (6 minutes) | *ActivPAL* (right thigh, chest; activPAL software; 15 sec; NR) | Posture/Activity type (lying, sitting, standing, stepping) | Observation (Direct) | Confusion matrix; Cohen k | The use of two activPAL devices enabled four behaviors to be accurately classified. | N/N | High |
| 31 | Battenberg et al. (2017, United States) | Adults (N=30; 25.6±2.5 yrs; 40% females; NR) | Walking (2430 meters); stairs (60 steps); treadmill (16 minutes) | *Fitbit One* (waist; NR; NR; NR); *Omrom HJ-321 (*waist; NR; NR; NR); *Sportline 340 Strider* (waist; NR; NR; NR); *Fitbit Force* (wrist; NR; NR; NR); *Nike+ Fuelband SE* (wirst; NR; NR; NR); *StepWatch Activity Monitor* (ankle, NR; NR; NR) | Intensity (Steps) | Observation (Manually counted) | Percentage error | Waist-based, dedicated activity monitors are highly accurate in a variety of activities. | NR/NR | High |
| 32 | Beecroft et al. (2008, Canada) | Adults that are mechanically ventilated (N=12; 68±13 yrs; 25% females; NR) | Sleep (1 night) | *Actiwatch 64* (wrist; Actiware-Sleep v. 3.4; 30 sec; NR) | Biological state (Total sleep time) | Polysomnography | Wilcoxon non-parametric test; Paired t-test; Pearson correlation; Sensitivity; Specificity; Accuracy | Actigraphy and behavioural assessment by the bedside nurse are inaccurate and unreliable methods to monitor sleep in critically ill patients. | N/NR | High |
| 33 | Beevi et al. (2016, Denmark) | Adults (N=14; 29.93±4.93 yrs; 35.7% females; NR) | Treadmill (1200 steps) | *Yamax DigiWalker SW-200* (waist; NR; NR; NR); *Omron HJ-720* (waist; NR; NR; NR); *Fitbit Zip* (waist; NR; NR; NR) | Intensity (Steps) | Observation (Manually counted) | Chi-squared test; Bland-Altman analyses | It was observed that all the evaluated devices have high error rates at 1 km/h and mixed error rates at 2 km/h, and at 3 km/h the error rates are the smallest of the three assessed speeds, with the OM and the FB having a slight overcount. | N/N | High |
| 34 | Bélanger et al. (2014, Canada) | Adults with and without obesity (N=28; 39±9 yrs; 57.1% females; NR) | Treadmill (15 minutes) | *ActiGraph GT3X* (waist; Actilife 5; 1 minute; NR) | Intensity (Steps) | Observation (Video) | Percentage error; ANOVA; unpaired t-test | In comparison with visual verification, the accelerometer step count function measurement error was larger for obese individuals walking at low speeds (2.5 km^h−1^). This error equated to an approximate 50% underestimation at these speeds. | NR/NR | High |
| 35 | Benito et al. (2012, Spain) | Adults (N=29; 24.2±1.9 yrs; 58.6% females; NR) | Resistance training (60 repetitions) | *SenseWear* (dominant arm; Innerview v4.02; NR; proprietary algorithm) | Intensity (Energy expenditure) | Indirect Calorimetry | Paired student t-test; Bland-Altman analyses; Three-way ANOVA; two-way ANOVA | The EE rose as exercise intensity increased, but was underestimated by the SWAs. | NR/NR | High |
| 36 | Berendsen et al. (2014, Netherlands) | Adults (N=5; 22.4±2.2 yrs; 20% females; NR) | Postures (15 minutes); Walking (10 minutes); Treadmill (14 minutes) | *ActivPAL3* (thigh; ActivPAL software 6.0.2; 1 sec; NR); *ActiGraph GT3X* (waist; Actilife v5.10.0; 1 sec; NR), *CAM* (thigh; Matlab; 1 sec; NR) | Posture/Activity Type (lying; sitting; upright; standing) | Observation (Direct) | Cohen’s kappa; Friedman’s ANOVA; Correlations | The ActivPAL3 is valid, reproducible and user friendly. The posture classification by the ActiGraphGT3X is not valid, but reflection of walking intensity and user friendliness are good. The CAM is valid; however, reproducibility at higher walking intensity and user friendliness might cause problems. | N/N | High |
| 37 | Bergamin et al. (2012, Italy) | Adults (N=30; 24.5±2.6 yrs; 50% females; NR) | Treadmill (20 minutes) | *MyWellness Key* (hip; NR; 1 sec; proprietary algorithm) | Intensity (Energy expenditure) | Indirect Calorimetry | One-way ANOVA; Linear regression; Pearson correlation; Bland-Altman analyses | A high correlation were found between oxygen utilization and the MWK with low standard errors estimates. This indicates that this accelerometer can be used to identify exercise intensities that are related to walking and running. | NR/NR | High |
| 38 | Bergman et al. (2008, United States) | Adults (N=21; 78.6±13.1 yrs; 76% females; NR) | Indoor walking (161 meters) | *StepWatch 3* (ankle, StepWatch analysis software; NR; NR); *Yamax Digi-Walker SW-200* (waist, NR; NR; NR) | Intensity (Steps) | Observation (Manually-counted) | Pearson correlations; repeated measures ANOVA | The SW3 pedometer was more accurate in counting steps and recorded higher 24-hour step counts than the DW pedometer. Thus, the SW3 is a valid research instrument for monitoring activity in the assisted-living population | NR/NR | High |
| 39 | Berninger et al. (2018, Netherlands) | Adults (N=11; 27.1±5.8 yrs; 78.6% females; NR) | Activities of daily life (Approx. 20 minutes) | *VitaBit* (pocket; web-based analytics portal; NR; proprietary algorithm) | Posture/Activity type (Sitting, standing, walking) | Observation (Direct) | Sensitivity; Specificity; Positive predictive rates; Negative predictive rates | According to the laboratory findings, high performance for sitting, standing, and walking makes the VitaBit eligible for SB monitoring. | NR/Y | High |
| 40 | Berntsen et al. (2010, Norway) | Adults (N=20; Males: 31±9.6 yrs, females: 39±7.6 yrs; 30% females; Caucasian (N=20)) | Activties of daily life and sport (120 minutes) | *ActiGraph 7164* (hip; NR; 1 minute; Freedson cut-points); *ActiReg* (chest, thigh; ActiCalc 32; 1 minute; NR); *ikcal* (chest; NR; 1 minute; NR); *SenseWear Pro2* (right upper arm; Innerview Professional Research Software v5.1; 1 minute; NR) | Intensity (Energy expenditure; Time spent in MVPA) | Indirect Calorimetry | Bland-Altman analyses; Intraclass correlation coefficient; Two-way ANOVA; standard one-sample t-test | Recorded time in MVPA and energy expenditure varies substantially among physical activity monitors. | N/N | Some |
| 41 | Berntsen et al. (2011, Norway) | Adults that are pregnant (N=29; 31±4.1 yrs; 100% females; 100% Caucasian) | Posture (25 minutes; Calisthenics (60 minutes); Bike ergometer (15 minutes); Outdoor walking (30 minutes) | *SenseWear Pro 2* (right arm; SenseWear Professional Research software v6.1; 1 minute; NR) | Intensity (Energy expenditure) | Indirect Calorimetry | Bland-Altman analyses; Two-way mixed Intraclass correlation | SenseWearTM Pro2 Armband is a valid measure of energy expenditure during pregnancy. | N/N | Some |
| 42 | Bertapelli et al. (2019, United States) | Adults with down syndrome (N=17; 33±15 yrs; 47.1% females; NR) | Walking (6 minutes) | *NL-1000* (waist; NR; NR; NR); *ActiGraph wGT3X+* (hip, wrist; ActiLife v6.13.3; NR; NR) | Intensity (Steps) | Observation (Manually counted) | Absolute percent error; ANOVA; Bland-Altman analyses | Results demonstrated that the pedometer and ActiGraph accelerometers have considerable error in measuring steps of persons with DS. Application of LFE, however, significantly improved the step-counting performance of the Actigraph accelerometers. | NR/NR | High |
| 43 | Bezuidenhout et al. (2021, Sweden) | Adults (N=30; 42±13 yrs; 53.3% females; NR) | Walking (74 meters) | *ActiGraph GT3X+* (right hip, left ankle, ActiLife 6 software v6.13.4; 1 sec; AG step detection algorithm) | Intensity (Steps) | Wearable (Stepwatch Acitivity monitor (right ankle)) | Mean percentage agreement; two-way random inter class correlation coefficient; percentage agreement; Bland-Altman plots; mean percentage bias | Ankle worn AG was the most sensitive to measure steps at a vast range of gait speeds. Our results suggest that sensor placement and filter settings need to be taken into account to provide accurate estimates of step counts. | N/N | High |
| 44 | Bhammar et al. (2016, United States) | Adults (N=34; 30.1±8.7 yrs; 76.5% females; NR) | Activities of daily life (116 minutes) | *SenseWear* (upper arm left triceps, SenseWear 7.0 and SenseWear 8.0; 1 minute; algorithm v2.2, v5.2 h) | Intensity (Energy expenditure; Time spent in MVPA) | Indirect Calorimetry | Linear mixed models; Mean absolute percentage errors; Paired t-tests; Bland-Altman analyses; Pearson correlation | Although both algorithms overestimated energy expenditure as well as time spent in moderate-intensity physical activity (P < 0.05), v5.2 offered better estimates than v2.2. | N/N | High |
| 45 | Bigué et al. (2020, Canada) | Adults with severe traumatic injuries (N=17; 30.4±14.7 yrs; 35.3% females; NR) | Sleep (1 night) | *Actiwatch-L or Actiwatch-Spectrum* (non-paralyzed arm; Actiware 5.0; 1 minute; threshold algorithms, regression equation-based algorithms) | Biological state (Total sleep time) | Polysomnography | Sensitivity; Specificity; Accuracy; repeated-measure ANOVA; Bland-Altman analyses | Actigraphy is valid for monitoring nighttime sleep and wakefulness in patients hospitalized with traumatic injuries, with sensitivity, specificity and accuracy comparable to actigraphic recordings in healthy individuals. A scoring algorithm using a low wake threshold is best suited for this population and setting. | N/N | High |
| 46 | Bijnens et al. (2019, Netherlands) | Adults (Study 1: N=10; 66–73 yrs; 70% females; NR; Study 2: N=10; 69–88 yrs; 60% females; NR) | Treadmill; Activities of daily life (NR) | *MOX Activity Logger* (thigh; NR; NR; physical activity classification algorithm) | Posture/Activity Type (dynamic, static) | Observation (Video) | Absolute percentage error; Bland-Altman analyses | Dynamic behavior is within acceptable limits under fixed conditions but has some limitations under simulated free-living conditions. We propose that this approach should be adopted by developers of activity trackers to facilitate the activity tracker selection process for researchers and clinicians. | N/N | Low |
| 47 | Boolani et al. (2019, United States) | Adults (N=120; 21.4±3.7 yrs; 38.3% females; NR) | Indoor walking (12 minutes) | *Fitbit Zip* (left waist or pocket, NR; NR; NR); *Garmin Vivofit* (right wrist; NR; NR; NR); *Basis B1 band* (left wrist, NR; NR; NR); *Misfit Shine* (right wrist; NR; NR; NR); *Nike Fuelband SE* (left wrist; NR; NR; NR) | Intensity (Steps; Energy expenditure) | Observation (Video); Indirect Calorimetry | Intraclass correlation coefficient; Bland-Altman analyses; Mean absolute percent error | The AMs exhibited varying degrees of accuracy for identifying steps, calories expended, HR, and distance walked across all different walking conditions. No single AM was accurate across conditions or metrics. Clinicians should be cautious when using data from these AMs to estimate patient activity levels. | N/N | High |
| 48 | Boudreaux et al. (2018, United States) | Adults (N=50; males: 22.0±2.67 yrs; females: 22.71±2.99 yrs; 56% females; NR) | Cycle ergometer and strength training (NR) | *Apple Watch 2* (wrist; NR; NR; NR); *Fitbit Blaze* (wrist; NR; NR; NR); *Fitbit Charge 2* (wrist; NR; NR; NR); *Garmin Vivosmart HR* (wrist; NR; NR; NR); *TomTom Touch* (wrist; NR; NR; NR); *Polar A360* (wrist; NR; NR; NR); *Polar H7* (chest; NR; NR; NR) | Intensity (Energy expenditure) | Indirect Calorimetry | Paired t-tests; Intraclass correlation; Mean absolute percent error; Bland-Altman analyses | EE estimates from wearable devices were inaccurate. Wearable devices are not medical devices, and users should be cautious when using these devices for monitoring physiological responses to exercise. | N/N | High |
| 49 | Bourke et al. (2016, Norway) | Adults (N=20; 76.4±5.6 yrs; 50% females; NR) | Activities of daily life (NR) | *ActiGraph GT3X+* (hip; NR; NR; State machine algorithm) | Posture/Activity Types (Standing, sitting, lying, walking) | Observation (Video) | Agreement; Kappa statistics; Krippendorff´s alpha | This is one of the most detailed validations of a body worn sensor algorithm to date and offers an insight into the challenges of developing a real-time physical activity classification algorithm for a tri-axial accelerometer based sensor worn at the waist. | N/NR | High |
| 50 | Bourke et al. (2019, Norway) | Adults (N=20; 76.4±5.6 yrs; NR; NR) | Activities of daily life (NR) | *ActivPAL3* (left thigh; activPAL software v7.1.2.142; NR; proprietary algorithms) | Intensity (Steps); Posture/Activity type (Sitting; lying; standing; walking; postural transitions)) | Observation (Video) | Percentage of agreement; Sensitivity; Specificity; Positive predictive value/percentage of agreement (PPV); Negative predictive value (NPV); accuracy; F1-score | Caution is advised when measuring relatively more intensive physical activity protocols (e.g. in-lab), assessing postural transfer quantity or during sedentary behaviour analysis, as some short duration sedentary bouts are ignored and postural transfers underreported. | N/NR | High/Some |
| 51 | Bowden & Behrman (2007, United States) | Adults with spinal cord injury (N=11; 21–63 yrs; 18.2% females; NR) | Walking (12 minutes; 40 meters) | *Stepwatch Activity Monitor* (ankle; Easy Start Programming software; 6 sec; NR) | Intensity (Steps) | Observation (Manually counted) | Intraclass correlation coefficient; Coefficients of variation | The SAM is an accurate and reliable device for capturing walking activity in individuals with iSCI. | N/N | High |
| 52 | Brazeau et al. (2011, Canada) | Adults (N=31; 26.7±6.3 yrs; 52% females; NR) | Cycling ergometer (45 minutes) | *SenseWear Pro 3* (right upper arm; Innerview Research Software 6.1; NR; NR) | Intensity (Energy expenditure) | Indirect Calorimetry | Paired t-tests; Pearson correlations; Intraclass correlations; Bland-Altman analyses | The results of the present study indicate that the SenseWear Armband underestimated energy expenditure during a 45-min ergocycling session at a 50 % VO_2peak_ intensity, mainly during the first 10 min. | N/NR | High |
| 53 | Brazeau et al. (2014, Canada) | Adults (N=38; 26.8±5.2 yrs; 47.4% females; Caucasian (N=18), Black (N=20)) | Treadmill (45 minutes); Cycling ergometer (45 minutes); Posture (30 minutes) | *SenseWear Pro 3* (right upper arm; Innerview Research Software 6.1); *Actical* (hip; manufacturer software v2.1; NR; 1-regression equation, 2-regression equation) | Intensity (Energy expenditure) | Indirect Calorimetry | Independent t-test; Paired t-test; Intraclass correlations; Pearsons correlation | Equations used to estimate energy expenditure from accelerometer data is less precise among Black adults than Caucasian adults. | N/N | High |
| 54 | Brazeau et al. (2016, Canada) | Adults (N=20; 26.2±3.6 yrs; NR; 85% Caucasian) | Postures (60 minutes); Treadmill (45 minutes); Ergometer (45 minutes) | *SenseWear Pro 3* (right upper arm; Innerview Research Software version 6.1; NR; NR); *Actical* (hip; manufacturer software v2.1; 1 minute; 2-regression equation) | Intensity (Energy expenditure) | Indirect Calorimetry | Pearson correlation coefficient; Intraclass correlation coefficient; Bland-Altman analyses; Paired t-tests | Acceptable estimation of total energy expenditure was observed with the SWA. Both devices were reliable but not accurate for energy expenditure’s estimations during rest and for specific exercises. | N/N | High |
| 55 | Brian & Haegele (2017, United States) | Adults (N=35; 20 yrs.; 34.3% females; African-American (N=5), Caucasian (N=30)) | Indoor walking (2 minutes); Exercise (25 minutes) | *Gopher FITStep Pro* (waist; NR; NR; NR) | Intensity (Time spent in MVPA; Steps) | Wearable (ActiGraph GT3X+ (waist)); Observation (Manually counted) | Agreement; Bland-Altman analyses; Paired sample t-tests; Mean absolute percent error | The GFSP may be an appropriate instrument for estimating steps, however users should be cautious when consuming MVPA estimates for educational, research, or health-related purposes | N/N | High |
| 56 | Briseno & Smith (2014, United States) | Adults with lower limb amputations (N=39; 48.0±14.1 yrs; 38.5% females; NR) | Indoor walking (200 meters) | *Digiwalker SW-701* (right hip; NR; NR; NR); *New-Lifestyles NL-800* (right hip; NR; NR; NR); *Omron HJ-112* (right hip; NR; NR; NR) | Intensity (Steps) | Observation (Manually counted) | Repeated-measures ANOVA; Intraclass correlation coefficient; Bland-Altman analyses; Percentage error | The HJ-12 and the NL-800 are viable options for persons with lower-limb amputations seeking to assess and/or monitor their physical activity levels. | N/N | High |
| 57 | Brown et al. (2013, United Kingdom) | Adults (N=22; 28±7 yrs; 31.8% females; NR) | Treadmill (25 minutes); Outdoor walking (2080 meters) | *ActiPed* (right shoe; ActiHealth website; NR; NR); *ActiGraph GT1M* (hip; ActiLife v5.10.0; 1 sec; NR); *Yamax Digiwalker* (hip; NR; NR; NR) | Intensity (Steps) | Observation (Video; Manually counted) | Pearson correlation coefficient; Bland-Altman analyses; Repeated measure ANOVA | The ActiPed showed acceptable levels of accuracy comparable to previous validated pedometers and accelerometers. The accuracy combined with the simple and informative remote gathering of data, suggests that the ActiPed could be a useful tool in objective physical activity monitoring. | N/N | High |
| 58 | Bunn et al. (2018, United States) | Adults (N=20; 26.6±11.5 yrs; 50% females; NR) | Treadmill (40 minutes) | *Apple Watch series 1* (wrist; NR; NR; NR); *Garmin 235* (wrist; NR; NR; NR); *Fitbit Surge* (wrist; NR; NR; NR); *Moto 360* (wrist; NR; NR; NR); *Polar A360* (wrist; NR; NR; NR); *Suunto Spartan Sport* (wrist; NR; NR; NR); *Suunto Spartan Trainer* (wrist; NR; NR; NR); *TomTom Spark 3* (wrist; NR; NR; NR) | Intensity (Steps) | Observation (Video) | Mean absolute percent error; Paired sample correlations; Equivalence tests; Cohen’s d; two one-sided tests | The devices tested had higher step accuracy with running than walking, except for the Polar. Overall, the Apple iWatch series 1, Moto 360, Garmin, and Suunto Spartan Trainer met the CTA standard for both walking and running. | N/N | High |
| 59 | Burton et al. (2018, Australia) | Adults (N=31; 74.2±5.8 yrs; 64.5% females; NR) | Walking (4 minutes) | *Fitbit Flex* (wrist; NR; NR; NR); *Fitbit Charge HR* (wrist; NR; NR; NR) | Intensity (Steps) | Observation (Video) | Intraclass correlation; Bland-Altman analyses | Reliability and validity of the Flex and ChargeHR when worn by older adults is good, however both devices underestimated step count within the laboratory environment. | N/N | High |
| 60 | Busse et al. (2009, United Kingdom) | Adults (N=18; 22-39 yrs; NR; NR) | Indoor walking (200 meters); Outdoor walking (1100 meters); Activities of daily life (30 minutes) | *StepWatch activity monitor* (ankle; NR; NR; NR) | Intensity (Steps) | Observation (Video) | Accuracy; Percentage error; Bland-Altman analyses; paired samples t-test | Activity monitors provide information that is related to actual activity and provide accurate and reliable data when tested on functional walking circuits. | N/N | High |
| 61 | Bussmann et al. (2004, Netherlands) | Adults with transtibial amputation (N=12; 61±10 yrs; 8% females; NR) | Activities of daily life (10 minutes); Treadmill (6 minutes); Walking (6 minutes); Bicycle ergometer (2 minutes); Stairs (2 minutes) | *Prosthetic Activity Monitor* (prosthesis; NR; NR; stride detection algorithm) | Posture/ Activity Type (inactive; active; locomotion); Intensity (steps) | Observation (Video) | Paired t-tests | The PAM provides valid data on activity classes and number of strides. | NR/N | Some/Some |
| 62 | Cakmak et al. (2020, United States) | Adults (N=102; 68±1.93 yrs; 0 % females; NR) | Sleep (1 night) | *Empatica E4* (wrist; NR; 30 sec; Oakley sleep/wake detection method) | Biological state (Sleep-wake states) | Polysomnography | Accuracy; Kappa statistics; Error rates; F1-score | CPD classification achieved balanced performance and higher AUC, despite underestimating sleep–wake transitions. | N/N | Some |
| 63 | Calabro et al. (2014, United States) | Adults (N=40; 27.4±6.7 yrs; 47.5% females; 42.5% Caucasian, 32.5% Asian, 20% Hispanics, 5% African-Americans) | Activities of daily life, exercise and treadmill (120 minutes) | *SenseWear Pro 3* (upper arm; SenseWear Professional Software v6.1; NR; algorithm v2.2.3); *SenseWear Mini* (wrist; SenseWear Professional Software v7.0; NR; algorithm v2.2.4); *Actiheart* (chest; v4.0.3.2; NR; combined activity and HR algorithm); *ActiGraph GT3X* (hip; NR; 1 minute; Freedson equation, Work-Energy Theorem formula); *ActivPAL* (right thigh; AP software v5.8.3.4; NR; equation reported) | Intensity (Total energy expenditure (MET)) | Indirect Calorimetry | Paired t-tests; Bland-Altman analyses; Pearson correlation; Absolute agreement; Sensitivity; Specificity; Kappa statistics | The SenseWear Mini provided more accurate estimates of EE during light to moderate intensity semi-structured activities compared to other activity monitors. | Y/N | Some |
| 64 | Carroll et al. (2012, Scotland) | Stroke patients (N=50; 72.4±12.3 yrs; 58% females; NR) | Walking (6.2 minutes) | *Omron HJ-113-E* (neck, left and right hip; NR; NR; NR) | Intensity (Steps) | Observation (Video) | Bland-Altman analyses; Mean differences; Estimation of the bias; Wilcoxon signed-rank test | Pedometers are feasible but generally do not detect steps at gait speeds below about 0.5m/s, and they undercount steps at gait speeds above 0.5m/s. | N/N | High |
| 65 | Casiraghi et al. (2013, United States) | Adults exercise group (N=18; 48.6±21.0 yrs; 61% females; NR); Adults resting group (N=22; 44.5±19.6 yrs; 59% females; NR) | Cycle-ergometer (NR) | *SenseWear* (right upper arm, waist; InnerView Research Software 6.1; NR; NR) | Intensity (Energy expenditure) | Indirect Calorimetry | Bland-Altman analyses; Pearson correlation | SWA, an extremely simple and inexpensive apparatus, provides quite accurate measurements of energy expenditure in humans and in baboons. Energy expenditure data obtained with SWA are highly correlated with the data obtained with ‘‘gold standard’’, IC, in humans. | N/N | High |
| 66 | Cavalheri et al. (2011, Brazil) | Patients with chronic obstructive pulmonary disease (N=36; 67±9 yrs; 44.4% females; NR) | Walking and activities of daily life (5 minutes) | *SenseWear* (right upper arm; SenseWear Professional 6.1; NR; manufacturer algorithms); *Yamax Digiwalker SW701* (waist; NR; NR; NR) | Intensity (Energy expenditure) | Indirect Calorimetry | Repeated measures ANOVA; Pearson correlation; Bland-Altman analyses | EE estimation by the SAB did not show difference in comparison to IC for the sum of the five activities although overestimation was found in activities involving walking. DW showed significant EE underestimation in the sum of the activities and for each activity. | N/N | Some |
| 67 | Cederberg et al. (2021, United States) | Adults with and without Parkinson’s disease (N=60, NR, NR, NR) | Indoor walking (6 minutes), Treadmill (6 minutes) | *ActiGraph GT3X+* (right wirst, left wrist; NR; 1 minute; ActiLife algorithm) | Intensity (Steps) | Observation (Manually- counted) | Absolute accuracy, relative accuracy, absolute precsision, Bland-Altman plots | Results suggest that placement of the device (i.e., dominant vs. non-dominant), type of activity (i.e., over-ground vs. treadmill walking), and presence of clinical conditions may impact the accuracy and precision of data when using the research-grade ActiGraph GT3X + accelerometer for measuring step counts. | N/N | High |
| 68 | Cellini et al. (2013, United States) | Adults (N=34; 20.77±3.14 yrs; 55.9% females; NR) | Sleep (daytime nap 2 hrs) | *Actiwatch-64* (non-dominant wrist, Actiware 5.52.0003; 1 minute; Actiware software algorithms); *ActiGraph GT3X+* (non-dominant wrist; ActiLife 6.4.3; 1 minute; Sadeh algorithm, Cole-Kripke algorithm) | Biological State (Total sleep time) | Polysomnography | Mann-Whitney U test; Intraclass correlation coefficients; Bland-Altman analyses; Accuracy; Sensitivity, Specificity; Cohen´s kappa | We conclude that both actigraph are valid and reliable devices for detecting sleep/wake diurnal patterns. The choice between devices should be based on several parameters as reliability, cost of the device, scoring algorithm, target population, experimental condition, and aims of the study (e.g., sleep and/or physical activity). | N/N | Some |
| 69 | Cereda et al. (2007, Italy) | Adult affected by newly diagnosed acute myelogenous leukemia (N=10; 56.6±13.3 yrs; 40% females; NR) | Resting (90 minutes) | *SenseWear* (upper arm; Innerview Research Software 5.1; NR; specific algorithms) | Intensity (Resting Energy expenditure) | Indirect Calorimetry | Wilcoxon nonparametric test; Kruskal-Wallis nonparametric test; Bland-Altman analyses; Correlation | SWA seems to provide accurate and reliable estimation of REE and useful information on TDEE also in cancer patients. Its use appears promising. Validation studies on larger samples and different cancer types should be considered | NR/NR | High |
| 70 | Chakar et al. (2017, Belgium) | Adults (N=38; 23.5±1.5 yrs; 47.4% females; NR) | Sleep (1 night) | *Actiwatch 2* (non-dominant wrist, Philips ActiWare software v. 6.0.1; 30 sec; NR) | Biological state (Total sleep time) | Polysomnography | Sensitivity; Specificity; Likelihood ratios; Cohen’s Kappa; Pearson correlation; Paired t-test | Midsagittal jaw movements analysis is a reliable method to measure sleep. In healthy adults, this device proved to be superior to actigraphy in terms of estimation of all sleep parameters and distinction of sleep-wake status. | NR/NR | High |
| 71 | Chandrasekar et al. (2018, United Kingdom) | Patients with polymyalgia rheumatica (N=27; 69.2±8.8 yrs; 89% females; NR) | Indoor walking (Approx. 10 minutes) | *Fitbit Zip* (right hip, shirt; NR; NR; manufacturers’ algorithms); *ActiGraph-GT3X+* (right hip, ActiLife 6 software; 1 minute; manufacturers’ algorithms) | Intensity (Steps) | Observation (Video) | Bland-Altman analyses; Percentage error | Preliminary results suggest that in controlled conditions, the Fitbit-Zip fairly accurately measures step-count during walking in people with PMR receiving treatment. However, device error was greater than data published in healthy people. The ActiGraph may not be recommended without activation of the LFE. | N/N | High |
| 72 | Chang et al. (2020, Taiwan) | Sedentray group (N=30; 21.9±1.9 yrs; 60% females; NR); Exercise-habit group (N=30; 21.7±1.6 yrs; 53.3% females; NR); Non-endurance group (N=30; 21.1±1.7 yrs; 43.3% females; NR); Endurance group (N=30; 20.9±1.7 yrs; 36.7% females; NR) | Treadmill (15 minutes) | *ActiGraph GT9X-Link* Version 1.7.1 (waist, ActiLife6 v.6.12.1; 10 sec; Weir equation) | Intensity (Energy expenditure) | Indirect Calorimetry | Paired t-tests; Cohen´s d effect size; Mean absolute percentage error; Pearson correlation; Intraclass correlation coefficient | Using accelerometers with a heart rate monitor can accurately predict Ees of athletes and non-athletes with an optimized predictive equation integrating the VM, HRR, and BM parameters. | N/N | Some |
| 73 | Chen & Sun (1997, United States) | Adults (N=125; males: 89.1±21.4 yrs, females: 76±24.0 yrs; 57.6% females; NR) | Activities of daily life (48 hrs) | *Tritrac* (right hip; MATLAB software package; NR; least-square simplex algorithm) | Intensity (Energy expenditure) | Indirect Calorimetry | Pearson correlation; Averaged relative differences; Standard errors of estimation; ANOVA; t-test | Furthermore, with our generalized models and by using subjects’ physical characteristics and body acceleration, EE can be estimated with higher accuracy (averaged SEE 5 0.418 W/kg) than with the Tritrac model. | N/NR | Low |
| 74 | Chen et al. (2003, United States) | Women (N=60; 35.4±9.0 yrs; 100% females; NR) | Activities of daily life (24 hrs) | *Tritrac R3D* (right hip; MATLAB software Package; NR; predictive equation); *Actiwatch* (dominant wrist; MATLAB software Package; NR; predictive equation) | Intensity (Energy expenditure) | Indirect Calorimetry | Pearson correlation; Standard errors of estimation; ANOVA | In this study, we demonstrated that movement measured using accelerometers at the hip and wrist could be used to accurately predict EEACT of various types and intensity of activities. | N/NR | Low |
| 75 | Chen et al. (2016, Taiwan) | Adults (N=30; 21.5±2.0 yrs; 50% females; NR) | Activites of daily life and treadmill (50 minutes) | *Fitbit Flex* (wrist; Fitbit.com Dashboard; NR; NR); *Garmin Vivofit* (wrist; Garmin Connect; NR; NR); *Jawbone UP* (wrist; UP App; NR; NR) | Intensity (Steps) | Observation (Video) | Percentage error; One sample t-test; Two-way repeated-measures ANOVA; Bland-Altman analyses | The wristband activity monitors examined were more accurate for measuring step counts between 80 and 134 m*min ^-1^ as compared with a slower speed. Accuracy under each common daily activity condition ranged widely between monitors and activity, with less error when worn on the nondominant wrist. | N/N | Some |
| 76 | Cheung et al. (2020, United States) | Sleep clinic patients (N=41; 42.2±14.7 yrs; 59% females; 63.4% White, 36.6% Non-White) | Sleep (1 night) | *Huami Arc* (non-dominant wrist; NR; 1 minute; Cole-Kripke algorithm); *Actiwatch Spectrum* (non-dominant wrist; Philips Actiware software version 6.0.9; 60 sec; Actiware algorithms) | Biological state (Total sleep time) | Polysomnography | Students-t test; Chi-square tests; Kruskal-Wallis and Fisher’s Exact (Non-parametric alternatives); Accuracy; Sensitivity; Specificity; Bland-Altman analyses; Box and whisker plots | An optimized sleep/wake threshold value was identified for a consumer-grade wearable Arc trained by PSG data. By applying his sleep/wake threshold value for Arc generated accelerometer data, when compared to PSG, sleep and wake estimates were adequate and comparable to those generated by a clinical-grade actigraph. | N/N | Some |
| 77 | Chinoy et al. (2020, United States) | Adults (N=34; 28.1±3.9 yrs; 64.7% females; NR) | Sleep (3 nights) | *Actiwatch 2* (wrist; NR; 1 minute; NR); *Fatigue Science Readiband* (wrist; NR; 1 minute; NR); *Fitbit Alta HR* (wrist; NR; 1 minute; NR); *Garmin Fenix 5S* (wrist; NR; 1 minute; NR); *Garmin Vivosmart 3* (wrist; NR; 1 minute; NR) | Biological state (Total sleep time) | Polysomnography | Sensitivity; Specificity; Positive and negative predictive value; Accuracy; Prevalence and bias adjusted kappa; Bland-Altman analyses; Student´s paired t-tests; Hedges´g effect sizes; R2 proportional biases | Consumer sleep-tracking devices exhibited high performance in detecting sleep, and most performed equivalent to (or better than) actigraphy in detecting wake. Device sleep stage assessments were inconsistent. | N/N | High |
| 78 | Choi et al. (2017, South Korea) | Patients with sleep-disordered breathing (N=36; 49.76±14.60 yrs; 16.7 % females; NR), Patients with chronic insomnia disorder (N=30; 58.11±9.73 yrs; 80% females; NR) | Sleep (1 night) | *Actiwatch 2* (non-dominant wrist; Actiware v.5.70 sleep software; NR; actiwatch algorithms) | Biological state (Total sleep time) | Polysomnography | Wilcoxon rank test; Friedman test; Intraclass correlation coefficients; Bland-Altman analyses | ACT is useful to monitor sleep and sleep quality in patients with CID. In patients with SDB, TST by ACT is reliable. However, ACT is insufficient to assess sleep quality due to its low agreement with PSG. | N/N | High |
| 79 | Choi et al. (2019, United States) | Adults (N=36; 21±4 yrs; 44.4% females; NR) | Activities of daily life (48 minutes) | *NL-1000* (non-dominant hip; NR; NR; NR) | Intensity (Energy expenditure) | Indirect Calorimetry | ANOVA; percentage error; Bland-Altman analyses; Multilevel regression analyses | Pedometer-determined step rate and its square were significant predictors of VO2 across different activities in healthy young adults. Height, BMI, or sex did not contribute to VO2 prediction. Accuracy of prediction across activities was low to moderate. | N/N | High |
| 80 | Choi et al. (2020, United States) | Adults (N=15; 31±14 yrs; 40% females; NR), Adults with down syndrome (N=15; 25±6 yrs; 40% females; NR) | Indoor walking (18 minutes) | *NL-2000* (hip; NR; NR; NR) | Intensity (Steps; Energy expenditure) | Indirect Calorimetry; Observation (Manually- counted) | Multilevel modeling; Percentage error; 2x3 ANOVA; Pearson correlation | The NL-2000i pedometer has high accuracy across speeds. Step-rate measured by NL-2000i predicts energy expenditure with a relatively low error in adults with and without DS. | N/N | High |
| 81 | Chou et al. (2009, United States) | Persons with lower limb amputation (N=9; 34-75 yrs; 0% females; NR) | Indoor walking (5 minutes) | *ActiGraph GT1M* (waist, ankle; ActiLife software; 1 minute; NR) | Intensity (Steps) | Observation (Manually- counted) | Simple linear regression; Squared r | GT1M demonstrated the highest accuracy when worn on the prosthetic ankle and when ambulating over level surfaces without the use of a walking aid. | N/NR | High |
| 82 | Chow et al. (2017, Australia) | Adults (N=31; 24.3±5.2 yrs; 61% females; NR) | Treadmill (15 minutes) | *ActiGraph wGT3XBT-BT* (waist, wrist; ActiLife v.6.11; 10 sec; NR); *Fitbit One* (waist; NR; NR; NR); *Fitbit Flex* (wrist; NR; NR; NR); *Fitbit Charge HR* (wrist; NR; NR; NR); *Jawbone UP24* (wrist; NR; NR; NR) | Intensity (Steps) | Observation (Video) | Percentage error; Two-way ANOVA | The accuracy and reliability of consumer-based PAMs and the Actigraph is affected by anatomical placement site and walking speed. The Fitbit One and Actigraph on the waist were the strongest performers across all speeds. | N/N | High |
| 83 | Chowdhury et al. (2017, United Kingdom) | Adults (N=30; 27±6; 50% females; NR) | Activities of daily life and treadmill (88 minutes) | *Microsoft Band* (wrist; app version 1.3.10506.1; NR; NR); *Apple Watch* (wrist; Watch OS v.1.0.1; NR; NR); *Fitbit Charge HR* (wrist; Fitbit app v.2.9.1; NR; NR); *Jawbone UP24* (wrist; UP app v.4.4; NR; NR); *Actiheart* (chest; Actiheart software; NR; NR); *SenseWear* (upperarm; SenseWear Pro 8.0; proprietary algorithm; NR) | Intensity (Energy expenditure) | Indirect Calorimetry | Bland-Altman analyses; Percentage error; Root mean squared error; Pearson correlation | None of the consumer devices were deemed equivalent to the reference method for daily energy expenditure. For all devices, there was a tendency for negative bias with greater daily energy expenditure. No consumer monitors performed as well as the research-grade devices although in some (but not all) cases, estimates were close to criterion measurements. | N/N | Some |
| 84 | Claridge et al. (2019, Netherlands) | Adults with cerebral palsy (N=14; 35.4±13.1 yrs; 35.7% females; NR) | Activities of daily life (NR) | *Activ 8* (two at thigh; Activ 8 software v.2.1.0.22; 5 sec; Activ8 classification algorithm) | Posture/Activity Type (lying, sitting, standing, bicycling, stair climbing) | Observation (Video) | Spearman correlation; Meng´s tests; Bland-Altman analyses | The activity monitor, positioned on the frontolateral thigh, demonstrated good criterion validity in ambulatory adults with cerebral palsy. Though the Activ8 offers potential as an objective measure of physical activity, appropriate positioning is paramount for valid measurement. | N/N | High |
| 85 | Clay et al. (2019, New Zealand) | Community-dwelling stroke survivors (N=19; 65.6±8.2; 58% females; NR) | Indoor walking (6 minutes) | *Fitbit Zip* (non-paretic hip; NR; NR; NR) | Intensity (Steps) | Observation (Video) | Kendall’s Tau-b correlation; Bland-Altman analyses | FBZ is an accurate measure of step activity in independent ambulators with stroke walking at speeds > 0.8m/s, but accuracy can be compromised with lower speed and poor gait quality. | N/NR | High |
| 86 | Clemes et al. (2009, United Kingdom) | Adults (N=68; 19.2±2.7 yrs; NR; NR) | Treadmill (15 minutes) | *Silva pedometer* (right and left hip; NR; NR; NR); *NL1000* (hip; NR; NR; NR) | Intensity (Steps) | Observation (Video) | Repeated-measure ANOVA; Percentage error | The Silva pedometer unacceptably inaccurate for activity promotion purposes, particularly in overweight and obese adults. | N/N | High |
| 87 | Colley et al. (2012, Canada) | Adults (N=40; 31.1±11.2 yrs; 60% females; NR) | Treadmill (28 minutes) | *SC-StepMX* (waist; NR; NR; NR); *Yamax DigiWalker* (waist; NR; NR; NR); *Actical* (waist; NR; NR; NR) | Intensity (Steps) | Observation (Manually-counted) | Absolute percent error; Bland-Altman analyses; Coefficients of determination | The SC-StepMX accurately measures step counts at slower walking speeds when compared with 2 other commercially available activity monitors. This makes the SC-StepMX useful in measuring step counts in populations that are active at lower intensities | Y/NR | High |
| 88 | Compagnat et al. (2018, France) | Adult stroke subjects (N=46; 64.4±14.4 yrs; NR; NR) | Activities of daily life (45 minutes) | *ActiGraph GT3X* (wrist, hip, ankle; NR; 1 sec; NR) | Intensity (Energy expenditure) | Indirect Calorimetry | Root mean square error; Pearson correlation; Bland-Altman analyses; Wilcoxon tests for paired data | This study found large differences and a poor agreement between the active energy expenditure as measured by the Actigraph and the Metamax according to the location of the sensor and the type of task performed by the subject. | N/N | Some |
| 89 | Compagnat et al. (2019, France) | Adult after-stroke (N=26; 64.6±14.8 yrs; NR; NR) | Walking (6 minutes) | *ActiGraph GT3X* (hip; NR; NR; Actigraph GT3X manufacturer algorithm); *Geonaute ONStep 400* (hip; NR; NR; NR) | Intensity (Energy expenditure) | Indirect Calorimetry | Bland-Altman analyses; Root mean square error; Pearson coefficient | This new method based on the energy cost and distance estimated by wearable devices provided better energy expenditure estimates for the pedometer than did the manufacturer´s algorithm. The validity of this method depended on the accuracy of the sensor to measure the distance walked by an individual after stroke. | N/N | High |
| 90 | Connolly (2010, United States) | Pregnant women (N=30; 30.6±5.6 yrs; 100% females; NR) | Treadmill (8-13 minutes) | *Yamax Digiwalker SW-200* (hip; NR; NR; NR); *New Lifestyles 2000* (hip; NR; NR; NR); *ActiGraph GT3X* (thigh; NR; NR; NR); *Omron HJ-720* (pants pocket, NR; NR; NR) | Intensity (Steps) | Observation (Manually- counted) | ANOVA; Bonferroni adjustments; Pearson correlation coefficient; Bland-Altman analyses | In pregnant women, the ACT and DW are less accurate than the NL and HJ. The HJ appeared to be the most accurate. | N/N | High |
| 91 | Connolly et al. (2020, United States) | Pregnant Women (N=39; 28.6±8.4 yrs; 100% females; NR) | Outdoor walking (600 steps) | *Omron HJ-720* (waist; NR; NR; NR), *New Lifestyles 2000* (front left pants pocket; NR; NR; NR), *Fitbit Flex* (non-dominant wrist, NR; NR; NR), *ActiGraph GT9X Link* (waist, NR; NR; NR), *StepWatch* (right ankle, software version 3.4; NR; NR) | Intensity (Steps) | Observation (Manually-counted) | Mean absolute percent error, two-way repeated measures ANOVA, independent samples t-tests | The OM, NL, and SW monitors are valid measures for overground step-counting during pregnancy walking. However, the OM and NL significantly underestimate steps by second and third trimester pregnant women in free-living conditions. | NR/NR | High |
| 92 | Conway et al. (2018, United States) | Women during pregnancy and postpartum (N=33; 29.6±3.5 yrs; 100% females; NR) | Activities of daily life and treadmill (35 minutes) | *ActiGraph GT3X+* (right hip, right ankle; NR; 1 sec; NR); *Omron Hj-720it* (right hip; NR; NR; NR); *SenseWear MF-SW* (left upper arm; NR; NR; NR) | Intensity (Energy expenditure; Steps) | Indirect Calorimetry | Pearson correlation; ANOVA | PA devices show moderate/strong reliability and moderate validity for measuring PA during pregnancy and postpartum. | N/N | Some |
| 93 | Cook et al. (2017, United States) | Patients with major depressive disorder (N=21; 26.5±4.6 yrs; 80.95% females; NR) | Sleep (1 night) | *Fitbit Flex* (non-dominant wirst; NR; 30 sec; NR) | Biological state (Total sleep time) | Polysomnography; Wearable (Actiwatch 2 (non-dominant wrist)) | Bland-Altman analyses; Sensitivity; Specificity; Accuracy | The FBF is not an adequate substitute for PSG when quantifying sleep in MDD, and the settings of the device sizably impact its performance relative to PSG and other standard actigraphs. The limitations and capabilities of the FBF should be carefully considered prior to clinical and research implementation. | N/N | High |
| 94 | Cook et al. (2019, United States) | Patients with suspected central disorders of Hypersomnolence (N=49; 30.3±9.84 yrs; 93.88% females; NR) | Sleep (1 night) | *Fitbit Alta HR* (non-dominant wrist; NR; 30 sec; NR) | Biological state (Total sleep time) | Polysomnopgraphy | Bland-Altman analyses; Sensitivity; Specificity; Accuracy | These results suggest FBA-HR cannot replace EEG-based measurements of sleep and wake in the diagnostic assessment of suspected CDH, and that improvements in device performance are required prior to adoption in clinical or research settings. | N/N | High |
| 95 | Cook et al. (2018, United States) | Patients with suspected central disorders of Hypersomnolence (N=43; 33.3±11 yrs; 67.4% females; NR) | Sleep (1 night) | *Actiwatch 2* (wrist; NR; 30 sec; NR); *Jawbone UP3* (wrist; Jawbone UP Android Application v4.24; 30 sec; NR) | Biological state (Total sleep time) | Polysomnopgraphy | Sensitivity; Specificity; Accuracy; Bland-Altman analyses | The JB3 did not accurately quantify or classify sleep in patients with suspected central disorders of hypersomnolence, and was particularly poor at identifying REM sleep. | N/N | High |
| 96 | Coote & O’Dwyer (2012, Ireland) | Adults (N=15; 46.1 yrs; 73.33% females; NR); Patients with Multiple Sclerosis (N=19; 50.6 yrs; 73.68% females; NR); Patients with multiple sclerosis (bilateral support) (N=11; 56.1 yrs; 63.64% females; NR) | Activites of daily life (75 minutes) | *ActivPAL* (thigh; ActivPAL Professional Software; NR; Proprietary algorithms); *SenseWear* (upper arm; SenseWear Innerview Professional Software v6.1; NR; proprietary algorithms) | Intensity (Energey expenditure, Steps) | Indirect Calorimetry; Observation (Video) | Intraclass correlation coefficients; Bland-Altman plots; Paired t-tests; Percentage error | The agreement between steps and MET estimates from both devices and the criterion was poor, particularly for people with MS. Only the step and MET estimates for the control group for the integrative accelerometer were not significantly different from the criterion. | N/N | Some |
| 97 | Coulter et al. (2017, United Kingdom) | People moderately affected by Multiple Sclerosis (N=20; 53.7±7.4 yrs; 55% females; NR) | Indoor walking (20-30 m) | *ActivPAL3* (thigh; ActivPAL Professional Software v7.2.23; NR; NR) | Intensity (Steps); Posture/Activity Type (upright) | Observation (Video) | Intraclass correlation coefficients; Bland–Altman analyses; Paired t -tests | The activPAL3 is valid for measuring walking activity in people moderately affected by MS. It is accurate for upright duration regardless of cadence. In participants with slow walking cadences, outcomes of steps taken and walking duration should be interpreted with caution. | N/N | High/High |
| 98 | Cox et al. (2014, Australia) | Adults with cystic fibrosis (N=26; 28±7 yrs; 57.69% females; NR) | Activities of daily life and walking (NR) | *SenseWear* (upper arm; NR; NR; proprietary algorithm) | Intensity (Energy expenditure) | Indirect Calorimetry | Intraclass correlation coefficients; Bland-Altman analyses | Overall, the SWA demonstrated good agreement with IC for EE estimates in CF adults during a series of free-living activities, however accuracy was variable when assessing EE for specific activities of shorter duration. | N/N | High |
| 99 | Crisafulli et al. (2011, Italy) | Patients with Chronic Respiratory Failure (N=40; 71.5±7.3 yrs; 35% females; NR); Adults (N=35; 69.4±6.1 yrs; 51.43% females; NR) | Indoor walking (6 minutes) | *SenseWear* (upper arm; InnerView Research Software, Professional v6.1; NR; NR) | Intensity (Energy expenditure) | Indirect Calorimetry | Bland-Altman analyses; T-test of Student; Two ways ANOVA; Wilcoxon and Kruskal-Wallis tests | SWA provides a feasible and valid method to assess the energy expenditure in CRF patients on LTOT, and it shows that aided walking results in a substantial energy saving in this population. | N/N | High |
| 100 | Crouter et al. (2003, United States) | Adults (N=10; 33±12 yrs; 50% females; NR) | Treadmill (25 minutes) | *Yamasa Skeletone* (waist, NR; NR; NR); *Sportline 330 and 345* (waist; NR; NR; NR); *Omron HJ-105* (waist; NR; NR; NR); *Yamax Digiwalker SW-701* (waist; NR; NR; NR); *Kenz Lifecorder* (waist; NR; 1 day; NR); *New Lifestyles 2000* (waist; NR; NR; NR); *Oregon Scientific PE316CA* (waist; NR; NR; NR); *Freestyle Pacer Pro* (waist; NR; NR; NR); *Walk4Life LS 2525* (waist; NR; NR; NR) | Intensity (Steps; Energy expenditure) | Observation (Manually- counted); Indirect Calorimetry | Intraclass correlation coefficients; Two-way ANOVA | In general, pedometers are most accurate for assessing steps, less accurate for assessing distance, and even less accurate for assessing kilocalories. | N/N | High |
| 101 | Crouter et al. (2004, United States) | Adults (N=20; males: 26±3.1 yrs, females: 23±2.4 yrs; 50% females; NR) | Treadmill and cycle rowing ergometer (NR) | *Polar S410* (wrist; NR; NR; proprietary algorithm) | Intensity (Energy expenditure) | Indirect Calorimetry | Three-way repeated measures ANOVA; Paired t-tests; Pearson correlation; Bland-Altman analyses | When the predicted values of VO_2max_ and HR_max_ are used, the Polar S410 HRM provides a rough estimate of EE during running, rowing, and cycling. Using the actual values for VO_2max_ and HR_max_ reduced the individual error scores for both genders, but in females the mean EE was still overestimated by 12%. | N/N | High |
| 102 | Crouter et al. (2008, United States) | Adults (N=48; 35±11.4 yrs; 50% females; NR) | Activites of daily life (Approx. 3 hrs) | *Actiheart* (chest; NR; 15 sec; prediction equations) | Intensity (Energy expenditure) | Indirect Calorimetry | Pairwise comparisons; Root mean square error; Bland-Altman analyses | The Actiheart combined activity and HR algorithm and HR algorithm provide similar estimates of AEE on both a group and individual basis. | N/N | Low |
| 103 | Crowley et al. (2019, Denmark) | Adults (N=20; 33±12 yrs; 60% females; NR) | Activities of daily life and walking (15 minutes) | *ActiGraph GT3X+* (thigh; NR; NR; NR), *Axivity AX3* (thigh; NR; NR; NR), *ActivPAL Micro 4* (thigh; NR; NR; rule-based algorithm) | Posture/Activity Type (sitting, standing, moving, walking, running, stairs, cycle) | Observation (Video) | Bland-Altman analyses; Sensitivity; Specificity; Coefficient of variation | Physical behaviors were classified with negligible difference between the accelerometer brands. | N/N | Some |
| 104 | Cruz et al. (2017, Portugal) | Adults (N=63; 45.8±20.6 yrs; 66.67% females; NR) | Indoor walking (120 meters) | *Yamax PW/EX-510* (neck, four at waist, right and left pockets of the trousers; NR; NR; NR); *ActiGraph GT3X+* (waist; ActiLife v6.7.2; 1 sec; NR) | Intensity (Steps) | Observation (Video) | Absolute percent error; Repeated-measure ANOVA; Bland-Altman analyses | Yamax PW/EX-510 pedometers may be preferable than GT3X+ accelerometers to count steps, as they provide more accurate results. These pedometers should be worn at the front right or left positions of the waist or inside the front pockets of the trousers. | N/N | High |
| 105 | Culhane et al. (2004, Ireland) | Adults (N=5; 72±13 yrs; 60% females; NR) | Activites of daily life (6 hrs) | *ADXL202 accelerometer* (thigh, chest; NR; NR; software algorithm) | Posture/Activity Type (sitting, standing, lying and moving) | Observation (Video) | Accuracy | In a population of older adults, the static activities of sitting, standing and lying and dynamic activities can be distinguished using the technique and threshold values outlined here to a degree of accuracy of 92% and higher. | Y/NR | High |
| 106 | Curran et al. (2021, Ireland) | Adults with cystic fibrosis (N=21; 25.3±5.98 yrs; 71.4% females; NR) | Treadmill and walking (35 minutes) | *ActivPAL3* (thigh; NR; NR; proprietary algorithms); *Fitbit Charge 2* (non-dominant wrist; NR; 1 minute; proprietary algorithms) | Intensity (Steps) | Observation (Video) | Pearson correlation; Bland-Altman analyses; Absolute mean percentage difference | The ActivPAL and Fitbit Charge 2 demonstrated acceptable validity for step count measurement in cystic fibrosis. | N/N | High |
| 107 | Cyarto et al. (2004, United States) | Adults (N=26; 79.4±8.2 yrs; 80.77% females; NR); Older adults from senior’s center (N=28; 70.6± 5.5 yrs; 82.14% females; NR) | Indoor walking (13 meters) | *Yamax Digiwalker DW-200* (waist; NR; NR; NR) | Intensity (Steps) | Observation (Video) | Percentage error; Independent t-tests; Repeated-measures ANCOVA | Slow walking speed and gait disorders hamper the utility of pedometers for physical activity measurement in frail seniors, such as NH residents, when worn at the usual attachment site. Pedometers, however, can be confidently used with ostensibly healthy older adult populations for both assessment and motivation purposes. | N/N | High |
| 108 | Daligadu et al. (2018, Canada) | Adults with post–cardiac surgery (N=20; 61.3±10.2 yrs; 10% females; NR) | Indoor walking (6 minutes) | *Fitbit Flex* (non-dominant wrist; NR; NR; NR) | Intensity (Steps) | Observation (Video) | Pearson correlation; Paired t-tests; Concordance correlation coefficient; Relative error; Bland-Altman analyses | The Fitbit Flex activity monitor was not a valid measure of step count and distance walked in this sample of post–cardiac surgery patients. The lack of agreement between outputs and criterion measures suggests the Fitbit Flex alone would not be an acceptable clinical outcome measure for monitoring walking progression in the early postoperative period. | N/N | High |
| 109 | Dannecker et al. (2013, United States) | Adults (N=19; 26.9±6.6 yrs; 47.37% females; NR) | Treadmill and activities of daily life and posture (4 hrs) | *Actical* (waist, manufacturer’s software; 1 minute; NR); *ActiGraph GT3X* (waist; Actilife v5.10; 1 sec; Freedson equation); *IDEEA* (each foot, each thigh, chest; manufacturer’s software; NR; NR); *DirectLife activity monitor* (waist, Web-based software; NR; NR); *Fitbit tracker* (waist, Web-based software; NR; NR) | Intensity (Energy expenditure) | Indirect Calorimetry | Kruskal–Wallis one-way ANOVA; Paired t-test | The shoe-based physical activity monitor provides a valid estimate of EE, whereas the other physical activity monitors tested have a wide range of validity when estimating EE. Our results also demonstrate that estimating EE based on classification of physical activities can be more accurate and precise than estimating EE based on total physical activity. . | N/Y | Some |
| 110 | Danzig et al. (2019, United States) | Adults (N=102; 56.4±16.3 yrs; 53.9 % females; NR) | Sleep (1 night) | *Jawbone UP3* (wrist, NR; NR; NR); *Actiwatch 2* (wrist; Actiware software v6.0.8; 15 sec; NR) | Biological state (Sleep time) | Polysomnography | Pearson correlation; Bland‐Altman analyses; Difference scores | Actiwatch and Jawbone misestimate sleep measures with very wide confidence limits and accuracy varies with multiple patient‐level characteristics. | N/N | High |
| 111 | Davoudi et al. (2019, United States) | Adults (N=40; 55.2±17.8 yrs; 70% females; NR) | Treadmill and activities of daily life (132 minutes) | *Samsung Gear S smartwatch* (right wrist, NR; NR; NR); *ActiGraph* *GT3X+* (right wrist; NR; NR; NR) | Intensity (Energy expenditure); Posture/Activity Type (Simple and complex activities) | Indirect Calorimetry | Pearson correlation; Root mean square; Accuracy | Results suggest that a commercial brand smartwatch can be used in place of validated research grade activity monitors for individual activity recognition, major body movement location detection, activity intensity detection, and locomotion detection tasks. | N/N | High/High |
| 112 | De Cocker et al. (2012; Belgium) | Adults (N=40; 29.5±7.7 yrs; 50% females; NR) | Treadmill and walking (Approx. 35 minutes) | *Omron HJ-203* (pants pocket, carrier bag, neck; NR; NR; NR) | Intensity (Steps) | Observation (Video), Wearable (Yamax Digiwalker SW-200 (right hip)) | Spearman correlation; Percentage error; ANOVA | The HJ-203 Omron pedometer showed acceptable accuracy for all wearing positions during stairs walking and treadmill walking at higher speeds | NR/NR | High |
| 113 | De Man et al. (2016, Australia) | Adults (N=6; 35.83±12.43 yrs; 83% females; NR) | Indoor walking (500 meters) | *Fitbit Charge HR* (wrist, NR; NR; proprietary algorithm) | Intensity (Steps) | Inertial measurement unit (hip); Self-reported step count | Paired sample t-test; Intraclass correlation coefficient; Mean absolute percentage errors | The findings suggest that interdevice measurement from dominant and nondominant hands is reasonably reliable, however less valid as compared to more robust researchgrade devices. | NR/NR | High |
| 114 | De Ridder & De Blaiser (2019, Belgium) | Adults (N=30; 24.9±5.33 yrs; 50% females; NR) | Indoor waking (1200 meters) | *Garmin Vivofit 3* (wrist; NR; NR; NR); *Nokia Go* (two at wrist and waist; NR; NR; NR) | Intensity (Steps) | Observation (Manually counted) | Repeated-measures ANOVA; Intraclass correlation coefficients; Paired sample t-test; Bland-Altman analyses; Percentage bias | Activity trackers showed no concurrent validity when monitoring step count during gait with crutches. This should be taken into account when implementing this technology in e.g. post-operative goal setting in patients with TKA. | N/N | High |
| 115 | Deans et al. (2020, United Kingdom) | Adults with unilateral lower limb absence (N=15, 59.20±12.03 yrs, 13% females, NR) | Activities of daily life (6 minutes) | *ActivPAL* (non-amputated thigh/ prosthetic; activPAL software; NR; proprietary algorithm) | Intensity (Steps); Posture/ Activity type (standing, sitting/lying) | Observation (Video) | Intraclass correlation coefficient; Paired t-test; Bland‐Altman analyses | The activPAL is a reliable measurement tool in adults with lower limb absence when used in a laboratory setting. Placement of the monitor on the sound side limb is recommended for testing. The activPAL shows evidence of relative validity, but not absolute validity. | N/NR | High/High |
| 116 | Delaney et al. (2021, Australia) | Adults with intensive care (N=46; 60.5 yrs; 30.4% females; NR) | Sleep (1 night) | *Actiwatch* *Plus* (wrist; Actiware software v6.0.9; 30 sec; proprietary algorithm) | Biological State (Sleep time) | Polysomnography | Kappa statistics; Sensitivity; Specificity; Pearson correlation; Bland-Altman analyses | Actigraphy was easy and safe to use, provided moderate level of agreement with polysomnography in distinguishing between sleep and wakeful states, and may be a reasonable alternative to measure sleep in intensive care patients. | N/N | High |
| 117 | Devine et al. (2021, United States) | Adults (N=8; 30.4±3.2 yrs; 50% females; NR) | Sleep (3 nights) | *Zulu watch* (non-dominant wrist; NR; 2 min; proprietary algorithm) | Biological state (Sleep time) | Polysomnography; Wearable (Actiwatch 2 (non-dominant wrist)) | Paired t-test; Bland‐Altman analyses Intraclass correlation coefficient; Accuracy; Sensitivity; Specificity | The Zulu watch showed mixed results but performed well in determining total sleep time, sleep efficiency, sleep onset, and final awakening in healthy adults compared with PSG or actigraphy. | N/N | High |
| 118 | DeVoe et al. (2003, United States) | Adults (N=17; males: 22.7±2.3 yrs; females: 24.2±1.5 yrs; 29.41% females; NR) | Treadmill and outdoor walking (Approx. 60 minutes) | *RT3 accelerometer* (lower back; NR; 1 sec; proprietary algorithm); *Tritrac* *R3D accelerometer* (lower back; NR; 1 min; proprietary algorithm) | Intensity (Counts) | Indirect Calorimetry | Factorial analysis of variance; Pearson correlation; Bland-Altman analyses | Differences in agreement between the RT3 and R3D did not vary in any systematic way over the range in testing conditions which substantiates that the RT3 and R3D accelerometers are sensitive on flat surfaces but are insensitive to changes in grade. | NR/NR | High |
| 119 | Diaz et al. (2016, United States) | Adults (N=13; 32.0±9.2 yrs; 100% females; White (N=6), Hispanic (N=7) | Treadmill (24 minutes) | *Fitbit One* (upper torso, hip; NR; 1 minutes; proprietary algorithm); *Fitbit Flex* (right wrist; NR; 1 minute; proprietary algorithm) | Intensity (Steps; Energy expenditure) | Observation (Video); Indirect Calorimetry | Lin’s concordance correlation coefficient; Paired t-test; Bland‐Altman analyses | Physical activity measures obtained from the upper torso attachment site of the Fitbit One are accurate across different walking and running speeds in female adults. The upper torso attachment site of the Fitbit One outperformed the wrist-based Fitbit Flex and yielded similar step count estimates to hip attachment. | N/N | High |
| 120 | Dick et al. (2010, Germany) | Adults with sleep apnoea (N=28; 56±10 yrs; 25% females; NR) | Sleep (1 night) | *SOMNOwatch* (wrist of the non-dominant arm; DOMINO light software; 30 sec; equation reported) | Biological state (Sleep time) | Polysomnography | Pearson correlation; Bland–Altman analyses; Sensitivity; Specificity | Actigraphy is not identical with PSG recording but gives good results in sleep/wake patterns and predicting TST, SPT, SSE, SE and SL also in sleep apnoea patients not suffering from other sleep disorders. | NR/NR | Some |
| 121 | Dijkstra et al. (2008, Netherlands) | Adults (N=20; 68*.*5±7*.*4 yrs; 50% females; NR); Adults with Parkinson’s disease (N=32; 67*.*3±6*.*6 yrs; 46.9% females; NR) | Indoor walking (45 meters) | *DynaPort MicroMod* (waist; NR; NR; NR); *Yamax Digi-Walker SW-200* (left and right hip; NR; NR; NR) | Intensity (Steps) | Observation (Video) | Percentage error; Repeated-measures ANOVA; Independent t-tests | Step counting of both pedometers was significantly less accurate for short trajectories (3 or 5 m) and as walking pace decreased The Yamax pedometer can be reliably used for this study population when walking at sufficiently high gait speeds (>1.0 m/s). The DynaPort is less speed-dependent and proved to be more appropriate in the PD patients for walking trajectories of 5 m or more. | N/N | High |
| 122 | Dijkstra et al. (2010, Netherlands) | Adults with mild to moderate Parkinson’s disease (N=32; 67.3±6.6 yrs; 46.9% females; NR) | Activities of daily life (Approx. 30 minutes) | *DynaPort MiniMod* (lower back; NR; NR; proprietary algorithm) | Posture/Activity Type (lying, sitting, standing, walking, shuffling) | Observation (Video) | Sensitivity; Specificity; Positive predictive values; One-way ANOVA | This triaxial monitor system is a practical and valuable tool for objective, continuous evaluation of walking and postures in patients with mild to moderate PD. Detection of sitting and standing requires further fine-tuning. | N/N | Some |
| 123 | Domene & Easton (2014, United Kingdom) | Adults (non-professional Latin dancers) (N=22; 36±10 yrs; 63.6% females; NR) | Dance (Approx. 60 minutes) | *ActiGraph GT3X+* (right wrist, right hip, right ankle; Actilife v.6.2; 1 sec; equation reported) | Intensity (Energy expenditure; Steps) | Indirect Calorimetry | Dependent t-tests; Bland-Altman analyses; Shapiro-Wilk test | Latin dance to salsa music elicits physiological responses representative of moderate to vigorous physical activity, and a wrist-worn accelerometer with simultaneous heart rate measurement constitutes a valid and reliable technique for the prediction of energy expenditure and step count during Latin dance. | NR/NR | High |
| 124 | Dondzila & Garner (2016, United States) | Adults (N=19; 24.6±3.1 yrs; 26% females; NR) | Treadmill (20 minutes) | *Fitbit Charge* (left wrist; NR; NR; NR); *Jabra Sport Pulse Wireless Earbuds* (both ears; NR; NR; NR) | Intensity (Energy expenditure) | Indirect Calorimetry | Mean absolute percentage errors; Paired t-test; Pearson correlation | Both devices considerably underestimated kcals, suggesting that caution be used when incorporating such data into fitness/health goals. | NR/N | High |
| 125 | Dondzila et al. (2012, United States) | Adults (N=102; Younger adults: 32.9±10.8 yrs; Older adults: 65.4±6.9 yrs; NR, NR) | Treadmill (20 minutes) and indoor walking (1.182 meters) | *Omron HJ-720ITC* (waist; NR; NR; NR); *Kenz Lifecorder* (waist; NR; NR; NR) | Intensity (Steps) | Observation (Manually counted) | Error score; One-sample t-tests | Both the OM and LC pedometers were more accurate as TM and OG walking speed increased. The OM significantly underestimated steps during the 24 hr compared with a standard of care evaluation. Overall, both uploadable pedometers appear acceptable to use in young or old age groups to measure walking behavior. | N/N | High |
| 126 | Dondzila et al. (2018, United States) | Adults (N=40; 21.6±2.0 yrs; 57.5% females; NR) | Treadmill (20 minutes) | *Fitbit Charge HR* (non-dominant wrist; NR; NR; NR); *Mio FUSE* (non-dominant wrist, NR; NR NR) | Intensity (Steps; Energy expenditure) | Observation (Manually counted); Calculated MET of treadmill intensities | One-way ANOVA; Bland-Altman analyses | Increasing exercise intensity is indicative of heightened accuracy for step detection and kcal estimation for the FB and MF, while decreasing heart rate accuracy for the FB. However, the MF performed poorly for estimating total daily activity. | NR/NR | High |
| 127 | Dooley et al. (2017, United States) | Adults (N=62; 22.55±4.34 yrs; 58.1% females; 47% nonwhite) | Treadmill (20 minutes) | *Apple Watch* (right/left wrist; NR; NR; proprietary algorithm); *Fitbit Charge HR* (right/left wrist; NR; NR; proprietary algorithm); *Garmin Forerunner 225* (right/left wrist; NR; NR; proprietary algorithm) | Intensity (Energy expenditure) | Indirect Calorimetry | Pearson correlation; Repeated-measures ANOVA; Bland-Altman analyses | This study provides one of the first validation assessments for the Fitbit Charge HR, Apple Watch, and Garmin Forerunner 225. An advantage and novel approach of the study is the examination of HR and EE at specific physical activity intensities. | N/N | High |
| 128 | Dorn et al. (2019, United States) | Adults (N=69; 26.4±8.7 yrs; 61% females; NR) | Treadmill (55 minutes); Outdoor walking (45 minutes); Bike (15 minutes); Swim (15 minutes) | *Fitbit Flex 2* (right/left wrist; NR; NR; SmartTrack*); Fitbit Charge 2* (right/left wrist; NR; NR; SmartTrack); *Fitbit Alta HR* (right/left wrist; NR; NR; SmartTrack); *Garmin Vívosmart HR* (right/left wrist; NR; NR; Move IQ) | Posture/Activity Type (walking, running, cycling, swimming) | Observation (Laptop timer) | Mean absolute percent error | In a controlled setting, wearable activity trackers provide accurate recognition of the type of some common physical activities, especially outdoor walking and running and walking on a treadmill. The accuracy of measurement of activity duration varied considerably by activity type and tracker model and was poor for complex sets of activity, such as a run embedded within 2 walking segments | N/N | High |
| 129 | Downs et al. (2015, Australia) | Adults with Rett syndrome (N=26; 18±8 yrs; 100% females; NR) | Walking (20-30 minutes) | *ActiGraph GTX3* (waist; NR; NR; proprietary algorithm); *ActivPAL* (thigh; NR; NR; proprietary algorithm); *StepWatch Activity Monitor* (ankle; NR; NR; proprietary algorithm) | Intensity (Steps) | Observation (Video) | Bland-Altman analyses; Hierarchical random effects modelling | The capacity of the SAM to measure physical activity in Rett syndrome allows focus on participation-based activities in clinical practice and clinical trials. | N/N | High |
| 130 | Duclos et al. (2019, Canada) | Adults with chronic hemiparesis (N=20; 53.9±10.8 yrs; 35% females; NR) | Walking (6 minutes) | *Fitbit One* (ankle, hip; NR; NR; proprietary algorithm) | Intensity (Steps) | Observation (Video) | Percentage error; Friedman ANOVA; Spearman correlation; Wilcoxon signed-rank test | Step counts are accurately measured with AM placed at the nonparetic ankle in laboratory and community settings. Accuracy can be altered by stairs and ramps among the slowest walkers and by prolonged walking tasks among faster walkers. | N/N | High |
| 131 | Dudek et al. (2008, Canada) | Adults with transtibial amputation (N=20; 58.6±10.8 yrs; 35% females; NR) | Activities of daily life (NR) | *Yamax Digi-Walker SW-700* (hip; NR; NR; NR); *Ossur patient activity monitor* (leg, NR; NR; proprietary algorithm) | Intensity (Steps) | Observation (Video) | Accuracy; T-tests | With acceptable step count accuracy, both devices are appropriate for assessing relatively continuous ambulation. | N/N | High |
| 132 | Duncan et al. (2011, United States) | Adults (N=57; 39.2±13.5 yrs; 56.1% females; NR) | Treadmill (28 minutes); Activities of daily life (10-15 minutes) | *Multi-sensor board* (right hip; NR; NR; proprietary algorithm); *Actical* (left hip; Actical Software v2.1; NR; NR) | Intensity (Energy expenditure); Posture/Activity Type (Sitting, walking, running) | Indirect Calorimetry; Observation (Direct) | Inference; Accuracy; Absolute Accuracy; Sensitivity; Specificity; Unpaired t-test; One-way ANOVA; Bland-Altman analyses | The MSB provides accurate measures of activity type in laboratory and field settings and energy expenditure during treadmill walking and running although the device underestimates energy expenditure in the field. | N/N | High/High |
| 133 | Durkalec-Michalski et al. (2013, Poland) | Adults (N=20; males: 26.5±5.0 yrs; females: 26.0±4.5 yrs; 55%, females; NR) | Activities of daily life (6 hrs) | *ActiGraph GT1M* (waist; NR; NR; Work-Energy Theorem/Freedson equation) | Intensity (Energy expenditure) | Indirect Calorimetry | Correlations; One-way ANOVA | Although AM and MR provided less accurate results than HRM in laboratory conditions, there were no significant differences between the three methods (HRM, AM and MR) when total daily energy expenditure was calculated for the participants in free-living condition. | NR/NR | High |
| 134 | Dutta et al. (2018, United States) | Adults (N=152; 18-64 yrs; 52% females, NR) | Activities of daily life (NR) | *GENEActiv* (non-dominant wrist, NR; 10 sec; sequential forward selection) | Posture/Activity Type (Stationary, walking, running, stair-climbing) | Observation (Video) | Accuracy | The free-living activity intensities were estimated with 80% accuracy and showed the dominance of stationary and light intensity activities in 36 out of 40 recorded sessions. This work proposes a novel activity recognition process to identify unsupervised free-living activities using lab-based classification models. | N/N | Some |
| 135 | Dwyer et al. (2009, Australia) | Adults with cystic fibrosis (N=17; 26±6 yrs; 35.3% yrs; NR); Adults (N=17; 29± 7 yrs; 47.1% females; NR) | Treadmill (20 minutes) | *SenseWear Pro3* (upper arm; InnerView Research Software v6.1; NR; proprietary algorithm) | Intensity (Energy expenditure; Steps) | Indirect Calorimetry; Observation (Direct) | Paired t-tests; Pearson correlation; Bland-Altman analyses; Standard multiple regression | Diagnosis of CF had no significant negative impact on the accuracy of the SWA estimate of EE. The SWA provided a reasonably accurate estimate of EE and step count during treadmill walking. | N/N | High |
| 136 | Edwardson et al. (2016, United Kingdom) | Adults (N=34; 27.2±5.9 yrs; 58.8% females; NR) | Activities of daily life (approx. 80 minutes) | *ActivPAL3* (right thigh; activPAL v7.2.29; 15 sec; proprietary algorithm); *GENEActiv* (right thigh; GENEActiv PC software v2.2; 15 sec; open-source algorithm); *ActiGraph GT3X+* (thigh, hip; AcitLife v6.10.2; 15 sec; proprietary algorithm) | Posture/Activity type (Lying, sitting, upright) | Observation (Direct) | Accuracy | All postural allocation algorithms when applied to devices worn on the thigh were highly accurate in identifying lying, sitting and upright posture. Given the poor accuracy of the waist algorithm for detecting sitting, caution should be taken if inferring sitting time from a waist-worn device. | NR/Y | High |
| 137 | Ehrler et al. (2016, Switzerland) | Adults (N=21; 34.5±15.7 yrs; 57.1% females; NR) | Walking and running (100+200 meters) | *iHealth activity monitor* (wrist, waist; NR; NR; NR); *Withings Pulse O2* (wrist, waist, necklace; NR; NR; NR); *Misfit Shine* (wrist, waist, necklace; NR; NR; NR); *Garmin vívofit* (wrist; NR; NR; NR) | Intensity (Steps) | Observation (Video) | Relative error; One-way ANOVA | At all positions, all tested pedometers generated significant errors at slow speeds and therefore cannot be used reliably to evaluate the amount of physical activity for people walking slower than 0.6 m/s (2.16 km/h, or 1.24 mph). | N/N | High |
| 138 | Ehrlich et al. (2021, United States) | Adults with gestational diabetes (N=15; 27.0±4.2 yrs; 100% females; NR) | Walking and running (12 minutes) | *Fitbit Charge 3* (non-dominant wrist; NR; NR; NR) | Intensity (Steps) | Observation (Manually- counted) | Mean absolute percentage error; Equivalence testing; Two one-sided tests method; Bland Altman analyses | The FC3 appears to be a valid step counter during the third trimester, particularly when walking or stepping-in-place at or close to women’s preferred cadence. | N/N | High |
| 139 | Ekelund et al. (2002, Schweden) | Adults with coronary artery disease (N=34; males: 59.9±7.3 yrs; females: 64.6±3.3 yrs; 14.7% females; NR) | Treadmill (18 minutes) | *ActiGraph AM7164* (lower back; NR; 15 sec; NR) | Intensity (Counts, Energy expenditure) | Indirect Calorimetry | ANOVA; Linear regression; Bland Altman analyses; Paired t-test | CSA activity monitor is a valid instrument for assessing the intensity of physical activity during treadmill walking in CAD patients. | NR/NR | Some |
| 140 | El-Amrawy & Nounou (2015, NR) | Adults (N=4; 26.5±12.8 yrs; 0% females; NR) | Walking and running (NR) | *Apple Watch* (wrist; NR; NR; NR); *Samsung Gear Fit* (wrist; NR; NR; NR); *Samsung Gear 1* (wrist; NR; NR; NR); *Samsung Gear 2* (wrist; NR; NR; NR); *Samsung Gear S* (wrist; NR; NR; NR); *iHealth Tracker* (wrist; NR; NR; NR); *Pebble Steel* (wrist, NR; NR; NR); *Pebble Watch* (wrist; NR; NR; NR); *Qualcomm Toq* (wrist; NR; NR; NR); *Motorola Moto 360* (wrist; NR; NR; NR); *Garmin Vivofit* (wrist; NR; NR; NR); *Mi Band* (wrist; NR; NR; NR); *MisFit Shine* (wrist; NR; NR; NR); *Jawbone UP* (wrist; NR; NR; NR); *Nike+ Fuelband SE* (wrist; NR; NR; NR); *Sony Smartband* (wrist; NR; NR; NR); *FitBit Flex* (wrist; NR; NR; NR) | Intensity (Steps) | Observation (Manually- counted) | Accuracy; Coefficient of variability | The accuracy and precision of the selected fitness trackers are reasonable and can indicate the average level of activity and thus average energy expenditure. | N/N | High |
| 141 | Ellender et al. (2021, Australia) | Sleep-clinic patients (N=54; 48.1±18.1 yrs; 57% females; NR) | Sleep (1 night) | *Jawbone UP3* (non-dominant wrist; Jawbone app v4.0.0; NR; NR) | Biological state (Total sleep time) | Polysomnography | Intraclass correlation coefficients; Bland-Altman analyses; Cohen´s d | Poor to moderate agreement was found between PSG and each of the tested devices, however, Jawbone UP3 had relatively better absolute agreement than other devices in sleep measurements compared with PSG. | N/N | High |
| 142 | Ellingson et al. (2016, United States) | Adults (N=49; 23.9±5.3 yrs; 63% females; NR) | Activites of daily life (75 minutes) | *ActiGraph GT3X+* (right hip; ActiLife v6.5.1; 1 sec; Schofield equation); *ActivPAL3* (right thigh; AP software; NR; Schofield equation) | Posture/Activity type (Lying, reading, walking); Intensity (Energy expenditure; Counts) | Indirect Calorimetry; Observation (Direct) | Confusion matrices; Log error; Root mean squares; Mean absolute error; Mean absolute percent error; Bland-Altman analyses | The SIP method was superior to SOJ for distinguishing between sedentary and light activities as well as estimating EE at higher intensities. | Y/NR | Low/High |
| 143 | Elsworth et al. (2009, United Kingdom) | Adults with neurological conditions (N=43; 54±13 yrs; 59.5% females; NR); Healthy adults (N=13; 29±12 yrs; 53.9% females; NR) | Walking and running (2 minutes) | *Yamax digiwalker SW-200* (right hip; NR; NR; NR) | Intensity (Steps) | Observation (Manually- counted) | Student’s t-test and bias; Random error; Intraclass correlation coefficient; Percentage variability | Pedometers may undercount when used for people with neurological conditions. There may be variability in pedometer accuracy but this was not strongly related to walking speed. | N/N | High |
| 144 | Enomoto et al. (2009, Japan) | Adults (N=31; 31.6±10.4 yrs; 35.5% females; NR) | Sleep (1 night) | *Lifecorder PLUS* (waist; NR; 2 minutes; equation reported) | Biological State (Sleep/wake algorithm) | Polysomnography | Sensitivity; Specificity; Agreement | Results demonstrate that sleep/wake activity in young to middle-aged healthy subjects can be assessed with a reliability comparable to that of conventional actigraphy through LC waist actigraphy and the optimal S/W scoring algorithm. | N/NR | High |
| 145 | Erdogan et al. (2010, Turkey) | Adults (N=43; 34.9±5.5 yrs; 62.8% females; NR) | Rowing (20 minutes) | *Polar S810i* (wrist; NR; 5 sec; NR); *SenseWear Pro Armband* (right upper arm; SenseWear Professional 6.1; NR; NR) | Intensity (Energy expenditure) | Indirect Calorimetry | Pearson correlation; Two-way repeated measures ANOVA; Bland-Altman analyses | Polar S810i and SWA showed reasonable concordance with IC for measuring EE of the moderate intensity exercise but not the lower intensity exercise on indoor rowing in the participants of this study. | NR/NR | High |
| 146 | Esliger et al. (2007, Canada) | Adults (N=38, 34.3±18 yrs, 58% females, NR) | Treadmill (18 minutes) | *Actical* (waist; NR; 1 minute; NR); *ActiGraph 7164* (waist, NR; 1 minute; NR) | Intensity (Steps) | Observation (Manually- counted) | Coefficient of variation; Paired samples t-test; Pearson correlation; Bland Altman analyses | The new step count function of the Actical accelerometer provides valid estimates of step counts at 83 and 133 m*min^-1^ on a range of healthy participants. | N/NR | High |
| 147 | Eyre et al. (2019, United Kingdom) | Adults (N=31; 22±3 yrs; 29 % females; NR) | Activities of daily life and treadmill (25 minutes) | *Research Tracker 6* (hip; NR; 1 minutes; NR); *ActiGraph* *GT9X Link* (both hips, both wrists; 1 minute; NR); *GENEActive* (both wrists; NR; 1 minute; NR) | Intensity (Energy expenditure) | Indirect Calorimetry | Spearman correlation; Bland-Altman analyses | The Research Tracker 6 performed similar to the ActiGraph and GENEActiv and is capable of classifying the intensity of physical activity in young adults. | N/N | High |
| 148 | Falter et al. (2019, Belgium) | Adults with cardiovascular disease (N=40; 61.9±15.2 yrs; 20% females; NR) | Cycle ergometer (NR) | *Apple Watch* (left wrist; NR; NR; NR) | Intensity (Energy expenditure) | Indirect Calorimetry | Bland-Altman analyses; mean absolute error; mean absolute percentage error; intraclass correlation coefficient | The Apple Watch systematically overestimates EE in this group of patients. Caution might therefore be warranted when using the Apple Watch for measuring EE. | N/N | High |
| 149 | Fanchamps et al. (2018, Netherlands) | Adults after a stroke (N=25; 56±12 yrs; 16% females; NR) | Activities of daily life (60 minutes) | *Activ8* (less-affected thigh; NR; 5 sec; NR) | Posture/Activity Type (IUpright position; standing; walking; lying/ sitting; cycling) | Observation (Video) | Agreement; Bland-Altman analyses | The Activ8 is sufficiently accurate in detecting different classes of body postures and movements of people after a stroke during basic activities and daily-life activities in a laboratory and/or at home. | N/N | Some |
| 150 | Farabi et al. (2017, United states) | Adults with Type 1 Diabetes (N=27; 23.8±4.1 yrs; 59.3% females; NR) | Sleep (1 night) | *Actiwatch 2* (non-dominant wrist; Actiware 6.0 software; 30 sec; NR) | Biological state (Total sleep time) | Polysomnography | Intraclass correlation coefficients; Bland-Altman analyses; Kappa statistics | When measuring sleep with the Actiwatch2 in young adults with T1DM, the low threshold setting provides the most accurate estimates of sleep parameters in comparison with PSG. | N/N | High |
| 151 | Faria et al. (2019, Brazil) | Adults with chronic stroke (N=30; 62±12 yrs; 30% females; NR) | Indoor walking (5 minutes) | *ActiGraph GT3X* (ankle, Actilife v4.1.0; 1 minute; work-energy theorem equation, Freedson equation, combined formula) | Intensity (Energy expenditure) | Indirect Calorimetry | Pearson correlation | The findings demonstrated that both the GT3X ActiGraph accelerometer and the Google Fit smartphone application do not provide valid measures of energy expenditure in chronic stroke individuals during fast overground walking. | N/N | High |
| 152 | Farmer et al. (2022, Australia) | Adults attending a rehabilitation clinic (N=88; 73±11 yrs; 51% females; NR) | Walking (4 minutes) | *Fitbit Zip* (foot; NR; NR; proprietary algorithm) | Intensity (Steps) | Observation (Manually-counted) | Paired sample t-test; Accuracy; Agreement; Intraclass correlation coefficient; Bland-Altman analyses; independent t-tests | The Fitbit Zip shows high step count accuracy with manual step count in a mixed subacute rehabilitation population. | N/N | High |
| 153 | Feehan et al. (2016, Canada) | Adults (N=22; 35.7±13.9 yrs; 68% females; NR) | Activities of daily life (35 minutes); Treadmill (5 minutes); Cycling (5 minutes) | *SenseWear Mini* (dominant upper arm, SenseWear Professional software v7; 1 minute; NR), *ActiGraph GT3X* (dominant waist, Actilife software v5; 1 minute; NR) | Intensity (Sedentary activity, non-sedentary activity) | Observation (Direct) | Sensitivity; Specifity | SWm may be a more suitable monitor for detecting time spent in sedentary and light-intensity activities. | N/N | High |
| 154 | Feito et al. (2012, United states) | Adults (N=56; normal weight: 28.3±10.5 yrs; overweight: 31.2±9.9 yrs; obese: 29.0±7.9 yrs; 50% females; NR) | Treadmill (NR) | *Digi-Walker SW-200* (right thigh; NR; 15 sec; NR); *Actical* (hip, Actireader v2.10; 15 sec; NR); *ActiGraph 7164* (hip; ActiSoft Analysis Software v.3.2.1.1; 15 sec; NR) *ActiGraph GT1M* (hip; ActiLife v4.4.1; 15 sec; NR); *ActiGraph GT3X* (hip; ActiLife v4.4.1; 15 sec; NR); *ActivPAL* (right thigh; Professional Research edition v5.8.5.0; 15 sec; NR); *StepWatch 3* (right ankle; Step-Watch v3.0; 15 sec; NR) | Intensity (Steps) | Observation (Manually counted) | Three-way repeated-measure ANOVA; Individual repeated-measure ANOVA; One-sample t-test | 67 m*min-1seems to be the minimum speed required for accurate step counting, at least for most waist-mounted activity monitors. Finally, the StepWatch, AG7164, and activPALi were the most accurate devices on the TM | N/N | High |
| 155 | Feito et al. (2015, United States) | Adults (N=24; 23.8±8.8 yrs; NR; NR) | Treadmill (NR) | *ActiGraph GT1M* (hip; ActiLifeMonitoring System version 5.1; 15 sec; NR); *ActiGraph GT3X* (hip; ActiLifeMonitoring System version 5.1; 15 sec; NR); *StepWatch 3* (right ankle; SW analysis software v3.1; 15 sec; NR) | Intensity (Steps) | Observation (Manually counted) | Two-way repeated-measures ANOVA; Paired sample t-test | Turning the LFE on lessens the underestimation of steps recorded at walking speeds ≤54 m*min-1 for both the GT3X and GT1M. | N/NR | High |
| 156 | Feng et al. (2017, United States) | Adults (N=25; 25.96±7.86 yrs; 44% females; NR) | Treadmill (12 minutes) | *APDM Opal* (waist, NR; 1 sec; Piecewise Aggregate Approximation, Symbolic Aggregate approximation; *ActiGraph wGT3X-BT* (left hip, NR; 1 sec; Piecewise Aggregate Approximation, Symbolic Aggregate approximation); *Axivity AX3* (right hip; NR; 1 sec; Piecewise Aggregate Approximation, Symbolic Aggregate approximation) | Intensity (Steps) | Observation (Video) | Percent error; Repeated measure ANOV; Two-way ANOVA; One sample t-test | Results highlight the variation between the performance of devices – the Axivity AX3 provides more accurate step counts than the other two devices. | N/N | Some |
| 157 | Fietze et al. (2015, NR) | Adults with obstructive sleep apnea syndrome (Study 1: N=30; 57±14 yrs; 16.67% females; NR; Study 2: N=20; 60±11 yrs; 30% females; NR) | Sleep (2 nights) | *Somnowatch* (chest; NR; 30 sec; NR) | Biological state (Total sleep time) | Polysomnography | Pearson correlation; Bland–Altman analyses; Sensitivity; Specificity; Accuracy | The study revealed high agreement between polysomnography and single lead EEG in sleep apnea patients. Deviations for REM were slightly higher for the single lead EEG compared to single lead EEG plus EOG/EMG. Both simplified systems proved to be reliable for comfortable out-patient sleep recording. | Y/NR | High |
| 158 | Foerster et al. (1999, Germany) | University students (N=24; 26 yrs; 0% females; NR) | Activities of daily life (approx. 60 minutes), walking stairs (140 seconds) and cycling (40 seconds) | *IC Sensor Model 3031* (sternum, wrist, thigh, lower leg; NR; NR; algorithm reported) | Posture/ Activity type (Lying, sitting, standing, walking, cycling) | Observation (direct) | Accuracy | The findings indicated that the detection of posture and motion based on accelerometry is highly reliable. The correlation between behavior observation and kinematic analysis was satisfactory, although some participants showed discrepancies regarding specific motions. | N/NR | High |
| 159 | Fokkema et al. (2017, Netherlands) | Adults (N=31; 32±12 yrs; 48.4% females; NR) | Treadmill (60 minutes) | *Garmin Vivosmart* (right wrist; NR; NR; NR); *Fitbit Charge HR* (right wrist; NR; NR; NR); *Polar Loop* (right wrist; NR; NR; NR); *Apple Watch Sport* (left wrist; NR; NR; NR); *Pebble Smartwatch* (left wrist; NR; NR; NR); *Samsung Gear S* (left wrist; NR; NR; NR); *Misfit Flash* (right hip; NR; NR; NR), *Jawbone Up Move* (right hip; NR; NR; NR); *Flyfit* (right ankle; NR; NR; NR); | Intensity (Steps) | Observation (Video) | Mean absolute percent error; Paired sample t-tests; Wilcoxon signed-rank tests; Bland–Altman analyses; Intraclass correlation coefficients | Test–retest reliability and validity of activity trackers depends on walking speed. In general, consumer activity trackers perform better at an average and vigorous walking speed than at a slower walking speed. | N/N | High |
| 160 | Fokkenrood et al. (2014, Netherlands) | Adults with peripheral arterial disease (N=21; 67±10 yrs; 38 % females; NR) | Activities of daily life (Range 48-110 minutes) | *Dynaport MoveMonitor* (waist; MoveMonitor analysis software version 2.6; NR; logical algorithms) | Posture/ Activity Type (lying; sitting; standing; locomotion; shuffling; not worn) | Observation (Video) | Intraclass correlation coefficients; Sensitivity; Specificity; Predictive values | The MoveMonitor provides accurate information on a diverse set of postures, daily activities, and number of steps in IC patients. | N/N | Low |
| 161 | Foster et al. (2005, United States) | Adults (N=20; lean group: 30±13 yrs; obese group: 32±7 yrs; 50% females; NR) | Treadmill (15 minutes); Indoor walking (25 minutes) | *Stepwatch* (ankle; NR; NR; NR); *Omron HF-100* (left waist; NR; NR; NR); *Accusplit Eagle Digi-Walker 2* (right waist; NR; NR; NR) | Intensity (Steps; Energy expenditure) | Observation (Manually counted); Indirect Calorimetry | Accuracy; ANOVA; Intraclass correlation coefficients; Paired t-tests; Unpaired t-tests; Regression analysis | The counts from the Stepwatch were virtually identical to the manual counts from a trained investigator and provided a reliable predictor of walking energy expenditure. . | N/N | High |
| 162 | Fridriksdottir & Bonomi (2020, Netherlands) | Adults (N=20; 43±13 yrs; 50% females; NR) | Treadmill, walking and activities of daily life (58 minutes) | *GENEActiv* (trunk; NR; NR; deep neural network, support vector machine) | Posture/Activity types (Lying; upright; walking; wheelchair) | Observation (Video) | Accuracy; Precision; Recall; F1-Score | DNN is capable of recognizing types of physical activity in simulated hospital conditions using data captured by a single tri-axial accelerometer. | N/N | Low |
| 163 | Fruin & Rankin (2004, United States) | Study 1: Adults (N=13; 20.2±1.0 yrs; 0% females; NR); Study 2: Adults (N=20; males: 25.2±3.2 yrs; females: 25.3±3.2 yrs; 50% females; NR) | Study 1: Cycle ergometry (40 minutes); Study 2: Treadmill (30 minutes) | *SenseWear Armband* (upper arm; InnerView Research Software v1.0; NR; NR) | Intensity (Energy expenditure) | Indirect Calorimetry | Two-factor repeated measures ANOVA; Paired t-tests; Pearson correlation; Bland-Altman analyses | The SWA provided valid and reliable estimates of EE at rest and generated similar mean estimates of EE as IC on the ergometer; however, individual error was large. The SWA overestimated the EE of flat walking and underestimated inclined walking EE. | Y/N | High |
| 164 | Fulk et al. (2014, United States) | Adults with stroke and traumatic brain injury (N=50; 52.9±15.1 yrs; 32% females; NR) | Walking (2 minutes) | *Fitbit Ultra* (waist; NR; NR; proprietary algorithms); *Nike+ Fuelband* (wrist; NR; NR; proprietary algorithms); *Yamax Digi-Walker SW-701* (waist; NR; NR; NR); *StepWatch Activity Monitor* (ankle; NR; NR; NR) | Intensity (Steps) | Observation (Video) | Intraclass correlation coefficients; Bland–Altman analyses; Pearson correlation | The Fitbit Ultra may be a low-cost alternative to measure the stepping activity in level, predictable environments of people with stroke and TBI who can walk at speeds ≥0.58 m/s. | N/NR | High |
| 165 | Fuller et al. (2021, Canada) | Adults (N=49; 18-56 yrs; 53% females; NR) | Treadmill (40 minutes) and posture (25 minutes) | *Apple Watch Series 2* (wrist; Apple HealthKit; NR; support vector machines, Random forest, Rotation forest); *Fitbit Charge HR2* (wrist; Fitbit SDK; NR; support vector machines, random forest, rotation forest) | Posture/Activity type (Lying; sitting; walking) | Indirect Calorimetry | Accuracy; Confusion matrices | This preliminary study demonstrated that data from commercial wearable devices could predict movement types with reasonable accuracy. | N/N | High |
| 166 | Furlanetto et al. (2010, Brazil) | Adults with chronic obstructive pulmonary disease (N=30; 67±8 yrs; 43.3% females; NR); Adults (N=30; 68±7 yrs; 50% females; NR) | Treadmill (6 minutes) | *Yamax Digi-Walker SW701* (waist; NR; NR; NR); *SenseWear Armband* (upper arm; NR; NR; NR) | Intensity (Steps; Energy expenditure) | Observation (Video); Indirect Calorimetry | Unpaired t test; One-way ANOVA; Bland-Altman analyses | In both patients with COPD and healthy elderly, the multisensor showed better EE estimates during most walking speeds than the pedometer. Conversely, for step counting, accuracy is observed only with the pedometer during the higher walking speed in both groups. | N/N | High |
| 167 | Gastin et al. (2018, Australia) | Adults (N=26; 21.3±2.4 yrs; 46% females; NR) | Exercise intervals with seated recovery (90 minutes) | *ActiGraph GT3X+* (right hip; Actilife v6.11; 1 minute; Freedson VM3 combination equation); *SenseWear Armband* (right upper arm; Sensewear Innerview Research Software v8.0; 1 minute; Internal proprietary algorithm v5.2) | Intensity (Energy expenditure) | Indirect Calorimetry | Accuracy; Mean bias; Percent difference; Cohen´s d; Root mean square error; One-way ANOVA; Bland Altman analyses | The ActiGraph GT3X+ and BodyMedia SWA do not provide valid EE estimates across a range of exercise modalities and intensities when compared to a criterion measure. | N/NR | Some |
| 168 | Gatti et al. (2015, Canada) | Adults (N=22; 23.86±1.93 yrs; 36.4% females; NR) | Treadmill or cycling (20 minutes) | *ActiGraph GT3X+* (right leg, right trunk, shin, two at thigh, two at waist; Actilife 6; 1 sec; NR) | Intensity (Steps) | Observation (Video) | Pearson correlation; Bland–Altman analyses; One-way ANOVA | GT3X+ can be used for measuring step-count during running and pedal-revolution count during bicycling. Only shank placement is recommended for both activities. | N/N | High |
| 169 | Gaz et al. (2018, United States) | Adults (N=32; 35.8±7.8 yrs; 68.8% females; NR) | Treadmill and walking (approx. 25-30 minutes) | *Fitbit One* (hip; NR; NR; NR); *Fitbit Zip* (hip; NR; NR; NR); *Fitbit Charge HR* (wrist; NR; NR; NR); *Apple Watch (*wrist; NR; NR; NR); *Garmin Vivofit2* (wrist; NR; NR; NR); *Jawbone UP2* (wrist; NR; NR; NR) | Intensity (Steps; Distance) | Observation (Manually counted) | Mixed linear models | Hip-based activity tracking devices varied in accuracy but performed better than their wrist-based counterparts for step accuracy. Distance measurements for both types of devices were more consistent but lacked accuracy. | NR/N | High |
| 170 | Giannakidou et al. (2012, Greece) | Adults (N=42; 22.6±2.8 yrs; 42.9% females; NR) | Treadmill (NR) | *Omron HJ-720* (hip; NR; NR; NR); *Omron HJ-113* (hip; NR; NR; NR); *Yamax Digi-Walker SW-200* (hip; NR; NR; NR) | Intensity (Steps; Energy expenditure) | Observation (Manually counted); Open-circuit spirometry | Root mean square difference; Standard error of measurement; Intraclass correlation; Two-way ANOVA | Omron HJ-720 and HJ-113 pedometers are accurate in the measurement of step-count, they demonstrate limited accuracy in the assessment of traveled distance and energy expenditure in a speed dependent manner. | N/NR | High |
| 171 | Gilgen-Ammann et al. (2019, Switzerland) | Adults (N=30; 29.5±5.1 yrs; 50% females; NR) | Activities of daily life and treadmill (70 minutes) | *Polar Vantage M wristwatch* (non-dominant wrist; NR; NR; NR) | Intensity (Energy expenditure) | Indirect Calorimetry | Mean absolute and percentage errors; Pearson correlation; Bland-Altman analyses | The Polar Vantage has a statistically moderate-to-good accuracy in EE estimation that is activity dependent. | Y/N | High |
| 172 | Gilgen-Ammann et al. (2021, Switzerland) | Adults (N=23; 20.9±2.5 yrs; 0% females; NR) | Military activities (Approx. 50 minutes) | *ActiHeart* (chest; firmware 4.0.109; 30 sec; NR); *Everion* (upper arm; firmware 02.23.00; 30 sec; NR); *Hidalgo EQ02* (chest; NR; 30 sec; algorithm settings Version 1.0); *Garmin Fenix 3* (wrist; firmware 8.3; 30 sec; NR); *PADIS 2.0* (hip; firmware 30.03.2017; 30 sec; NR) | Intensity (Physical activity energy expenditure) | Indirect Calorimetry | Mean absolute error; Mean absolute percentage error; Bland–Altman analyses; Pearson correlation | The present study demonstrated poor to moderate validity in terms of PAEE estimation, but excellent validity in all investigated devices in terms ofHRassessment. | N/N | Some |
| 173 | Gilmore et al. (2020, Australia) | Patients after lumbar fusion surgery (N=40; 65±13.31 yrs; 65% females; NR) | Walking (2 minutes) | *ActivPAL3* (thigh; software provided by PAL Technologies; NR; NR); *Fitbit Flex* (wrist, thigh; NR; NR; NR); *Jawbone UP Move* (wrist, thigh; NR; NR; NR) | Intensity (Steps) | Observation (Video) | Intraclass correlation coefficient; Bland-Altman analyses; Independent t-test; Pearson correlation | The ActivPAL3 activity monitor is a sufficiently valid tool to detect step count immediately after lumbar fusion. | N/N | High |
| 174 | Giurgiu et al. (2020, Germany) | Adults (N=20; 25.68±4.55 yrs; 50% females; NR) | Activities of daily life, walking and postures (74 minutes) | *ActiGraph GT3X+* (right waist; ActiLife v6.13.3; 1 sec; NR); *ActivPAL3 micro* (thigh; PAL connect v8.10.5.55; 1 sec; NR); *Move4* (right hip, thigh; DataAnalyzer v1.13.5; 1 sec; NR) | Posture/Activity type (Sitting, lying, sedentariness) | Observation (Video) | Cohen’s kappa; Sensitivity; Specificity; Accuracy; Youden’s index | Thigh-worn devices, namely the Move and the ActivPAL, achieved up to excellent validity in measuring sitting/lying body positions and sedentary behavior | NR/NR | Some |
| 175 | Glasheen et al. (2020, United States) | Wheelchair users (N=15; 47±12 yrs; 33% females; NR); Able-bodied individuals (N=15; 25±4 yrs; 47% females; NR) | Treadmill (20 minutes); Ergometry (20 minutes); Overground obstacle (50 meters); Figure 8 course (100 meters) | *Apple Watch Series 1* (dominant wrist; watchOS 3.2; NR; NR) | Intensity (Counts) | Observation (Direct) | ANOVA; Paired samples t-tests; Bland-Altman analyses; Mean absolute percentage error; Intraclass correlation coefficient | Apple Watch is suitable for tracking high-frequency standardized (i.e., treadmill) pushing and arm ergometry but not low-frequency pushing or overground tasks. | N/N | High |
| 176 | Goel et al. (2020, United States) | Patients after total hip and knee arthroplasty (N=24; 63.9±8.6 yrs; 41.7% females; NR) | Walking (30 meters) | *Fitbit Charge HR* (ankle, wrist; NR; NR; NR) | Intensity (Steps) | Observation (Manually counted) | Independent samples t-test; Mann-Whitney U analysis; One-way ANOVA; Linear regression; Error rate | In inpatients with TJA, AMDs and SPs have unacceptable variability and limited utility for step counting when using a walker. As gait normalizes and the level of ambulatory assist decreases, AMDs on the contralateral ankle and SPs on the contralateral hip demonstrated low error rates. | N/NR | High |
| 177 | Gould et al. (2021, United States) | Adults (N=24; 19±0.8 yrs; 50% females; African-American (N=12), Caucasian (N=17)) | Treadmill (50 minutes) | *Actical* (left waist; NR; NR; NR); *New Lifestyles* *SW-200* (left waist; NR; NR; NR); *ActiGraph GT3X+* (right hip, non-dominant wrist; NR; NR; NR); *New Lifestyles NL-1000* (right hip; NR; NR; NR); *SenseWear Armband* (right arm; NR; NR; NR); *ActivPAL* (right thigh; NR; NR; NR); *StepWatch* (right ankle; NR; NR; NR) | Intensity (Steps) | Observation (Video) | Mean absolute percentage error; Mean percentage error; Correlation | Reduced performance can be expected at very slow walking speeds (0.8 to 3.2 km/h) for all devices. Ankle-worn and thigh-worn devices demonstrated the highest accuracy. Speed and wear location had a significant effect on accuracy and bias, but not precision. | N/N | High |
| 178 | Grant et al. (2006, United Kingdom) | Staff and students at University (N=10; 43±10.6 yrs; 60% females; NR) | Activities of daily life (Approx. 30 minutes) | *ActivPAL* (thigh; activPAL Professional research edition; 1 sec; proprietary algorithm) | Posture/Activity type (Sitting, upright, standing, walking) | Observation (Video) | Bland-Altman analyses; Agreement; Sensitivity; Specificity; Predictive value | The activPAL activity monitor is a valid and reliable measure of posture and motion during everyday physical activities | NR/NR | High |
| 179 | Grant et al. (2008, United Kingdom) | Adults (N=21; 71.9±5.7 yrs; 50% females; NR) | Treadmill and outdoor walking (25 minutes and 500 meters) | *ActivPAL* (thigh; proprietary software; NR; NR); *Digi-Walker SW-200* (waist; NR; NR; NR); *New-Lifestyles NL-2000* (waist; NR; NR; NR) | Intensity (Steps) | Observation (Video) | Bland-Altman analyses; Percentage error | The activPAL monitor accurately recorded step number and cadence. Combined with its ability to identify primary postures, the activPAL might be a useful and versatile device for measuring activity in older adults. | N/Y | High |
| 180 | Groot & Nieuwenhuizen (2013, Netherlands) | Adults (N=28; 31.5±14.4 yrs; 57.1% females; NR) | Treadmill, cycling and activities of daily life (45 minutes) | *DynaPort MoveMonitor* (lower back; MoveMonitor software; 1 minute; NR) | Intensity (Energy expenditure); Posture/Activity type (Sitting, lying, standing, stairs climbing, cycling) | Indirect Calorimetry | Percentage agreement; Intraclass correlation; Bland-Altman analyses; Standard error of the measurement | In conclusion, most activities were categorized correctly, MI seemed to be valid and reliable but reliability is low for relative time spent in activities and EE cannot be estimated well. | N/N | Low/High |
| 181 | Gruwez et al. (2009, Belgium) | Patients suffering from obstructive sleep apnea (N=22; 53±13 yrs; 22.7% females; NR) | Sleep (1 night) | *Withings Pulse 02* (Non-dominant wrist; NR; NR; NR); *Jawbone UP* (Non-dominant wrist; NR; NR; NR); *SenseWear Pro Armband* (upper arm; NR; NR; NR) | Biological state (Total sleep time) | Polysomnography | Wilcoxon signed rank test; Intraclass correlation coefficient; Bland-Altman analyses | Results confirmed the limited performance of wearable sleep monitors that has been previously observed in healthy subjects. In OSA patients, wearable app-based health technologies provide a good estimation of TIB and light sleep but with very poor ICC. | N/N | High |
| 182 | Gusmer et al. (2014, United States) | Adults (N=32; 22±2 yrs; 78.1% females; White (86%)) | Treadmill (60 minutes) | *ActiGraph™ GT1M* (waist; Actilife 5; 1 minute; Freedson equation); *Fitbit Ultra* (waist; application programm; 1 minute; NR) | Intensity (Energy expenditure) | Indirect Calorimetry | Pearson correlation; Paired t-test; Bland-Altman analyses | Results of this pilot study suggest that the FitBit® and the ActiGraph™ can be used interchangeably to measure steps, but not to measure kilocalories. Furthermore, the FitBit® underestimates energy expenditure, compared to a metabolic cart, as exercise intensity increases. | N/N | High |
| 183 | Hall et al. (2013, United States) | Adults (N=20; 75.0±8.9 yrs; 25% females; NR) | Treadmill and walking (48 minutes) | *ActiGraph GT3X* (right hip; NR; 1 sec; equation reported) | Intensity (Energy expenditure) | Indirect Calorimetry | Repeated-measured ANOVA; Root-mean-square error | This study identifies the need for equations and cut points specific to older adults. | N/N | Some |
| 184 | Harrington et al. (2011, Ireland) | Adults (N=62; 18.5±3.4 yrs; 100% females; NR) | Treadmill (7 minutes) | *ActivPAL* (thigh; NR; 15 sec; equation reported) | Intensity (Energy expenditure; Steps) | Indirect Calorimetry, Wearable (Actigraph GT1M (hip); Observation (Video) | Paired t-tests; Spearman correlation; Intraclass correlation coefficient; Bland-Altman analyses | The ActivPAL step function performs better than the Actigraph at the slowest walking speed under treadmill conditions. | NR/NR | Some |
| 185 | Hart et al. (2011, United States) | Adults (N=29; 28.9±6.2 yrs; 55% female; NR) | Posture and treadmill (25 minutes) | *ActiGraph 7164* (right hip; NR; 1 minute; ≤50 cpm, <100 cpm, ≤259 cpm); *IDEEA* (two at feet, two at thighs, chest; NR; 1 sec; NR); *ActivPAL* (right thigh; NR; 15 sec; NR) | Posture/Activy type (Time spent sedentary); Intensity (Time spent walking) | Observation (Video) | Mean percent error | In a laboratory setting, the utility of all instruments to classify activities into behavioral categories was confirmed. | N/N | High/High |
| 186 | Härtel et al. (2011, Germany) | Rehabilitation patients (N=9; 46.4±10.9 yrs; 0% females; NR) | Activities of daily life (100 minutes) | *kmsMove-sensor* (hip; NR; 1 sec; equiation reported) | Intensity (Energy expenditure | Indirect Calorimetry | Intraclass correlation coefficient; Bland-Altman analyses | Findings indicate that the kmsMovesensor is an appropriate measuring device with relatively good accuracy to assess human energy expenditure in rehabilitation patients. | NR/NR | Some |
| 187 | Hartung et al. (2020, Germany) | Adults (N=18, 28.8±5.0 yrs; 61% females; NR) | Indoor Walking (750 steps; 36 short walking bouts); Activities of daily life (40 movements) | *Fitbit Inspire* (wrist, hip, ankle; NR; NR; NR); *Fitbit Ionic* (wrist; NR; NR; NR); *Garmin vivofit 4* (wrist; NR; NR; NR); *Garmin vivomove* (wrist; NR; NR; NR); *Withings Pulse HR* (wrist; NR; NR; NR); *Withings Steel HR* (wrist; NR; NR; NR); *Xiaomi Mi Band 3* (wrist; NR; NR; NR); *Samsung Galaxy Watch Active* (wrist; NR; NR; NR); *ActiGraph wGT3X-BT* (hip; Actilife v.6.13.4; 1 sec; NR) | Intensity (Steps) | Observation (Manually counted)) | Mean absolute percentage error | Smartphone data collected at the hip, analyzed with a separate algorithm, performed either equally or even superiorly to the research-grade ActiGraph. | N/N | High |
| 188 | Hasson et al. (2009, United States) | Adults (N=92; 29±11 yrs; 52% females; NR) | Treadmill (36 minutes); Walking (19 minutes) | *Omron HJ-112* (hip, pants pocket, chest pocket, around neck; NR; NR; NR); *Yamax Digiwalker SW-701* (hip; NR; NR; NR) | Intensity (Steps) | Observation (Manually counted) | Random error; Percent difference | The Omron HJ-112 pedometer validly assesses steps in different BMI groups during constant- and variable-speed walking; other than that in the pants pocket, placement of the pedometer has little effect on validity. | Y/NR | High |
| 189 | Haymes & Byrnes (1993, United States) | Adults (N=20; NR; 50% females; NR) | Treadmill (36 minutes) | *Caltrac* (hip; NR; NR; NR) | Intensity (Activity counts; Energy expenditure) | Indirect Calorimetry | Three-factor repeated-measures ANOVA; Pearson product correlation correlations, Linear regressions | The Caltrac is a valid indicator of physical activity during walking but does not adequately discriminate between running speeds of 5-8 mph. | NR/NR | High |
| 190 | Hedayatrad et al. (2021, New Zealand) | Adults (N=33; 28-59 yrs; 52% females; NR) | Postures (30 minutes); Treadmill (6 minutes) | *ActiGraph GT3X+* (waist; ActiLife v6.11.9; 5 sec; proprietary inclinometer algorithms, Freedson cut-points, Evenson cut-points); *Axivity AX3* (lower back; OMGUI v 1.0.0.30; 1 sec; Freedson cut-points, Evenson cut-points, activity recognition models) | Intensity (Sedentary; LPA; MPA; VPA); Posture/ Activity type (Sitting; standing; lying) | Observation (Video) | Sensitivity; specifity; balanced accuracy; confusion matrices; Kappa statistics; independent samples t tests; paired t tests | In comparison with the GT3X+ accelerometer, AX3 was able to detect various postures and activity intensities with slightly higher balanced accuracy in children and adults. | NR/NR | High/Some |
| 191 | Hedner et al. (2004, United States, Israel, Sweden) | Adults with and without sleep apnea (N=228; 48.8±14.0 yrs; 28.5% females; NR) | Sleep (1 night | *Watch_PAT100* (non-dominant wrist; sleep/ wakefulness analysis software; 30 sec; sleep/ wake algorithm) | Biological state (Total sleep time) | Polysomnography | Sensitivity; Specificity; Agreement; ANOVA; Paired t-test | This actigraphy algorithm provides a reasonably accurate estimation of sleep and wakefulness in normal subjects and patients with obstructive sleep apnea on an epoch-by-epoch basis. | Y/Y | Some |
| 192 | Heiermann et al. (2011, Germany) | Adults (N=32; 68.6±5.1 yrs; 59% females; NR) | Resting (20 minutes) | *SenseWear Pro 2 Armband* (right upper arm; InnerView Professional v5.0; NR; proprietary algorithm) | Intensity (Resting energy expenditure) | Indirect Calorimetry | Paired sample t-tests; Bland-Altman analyses; Pearson correlation | The SWA provides a reliable estimate of REE in healthy older subjects and has the advantage of easy handling. The 20-min recording time, which was recommended by the manufacturer, can be applied. | N/N | High |
| 193 | Hendelman et al. (2000, United States) | Adults (N=25; 40.8±7.2 yrs; 60% females; NR) | Indoor walking (20 minutes), Golf (2 holes); Activities of daily life (25 minutes) | *CSA accelerometer 7164* (right hip; NR; 1 min; NR); *Tritrac monitor* (left hip; NR; 1 min; NR); *Yamax Digiwalker SW-701* (right hip; NR; NR; NR) | Intensity (Activity counts; Energy expenditure) | Indirect Calorimetry | Pearson correlation coefficients; regression analysis; Two-factor repeated-measures ANOVA | The count versus METs relationship for accelerometry was found to be dependent on the type of activity performed, which may be due to the inability of accelerometers to detect increased energy cost from upper body movement, load carriage, or changes in surface or terrain. This may introduce error in attempts to use accelerometry to assess point estimates of physical activity energy expenditure in free-living situations. | N/N | Some |
| 194 | Hendrikx et al. (2017, Netherlands) | Adults (N=29; 41.2±14.4 yrs; 51.7% females; NR) | Treadmill (6 minutes); Bike ergometer (3 minutes); Cross trainer (3 minutes), Activities of daily life (21 minutes), postures (6 minutes); Outdoor walking (6 minutes), Outdoor cycling (3 minutes) | *Philips Health Watch* (wrist; Companion app; 1 minute; NR) | Intensity (Energy expenditure; Steps); Posture/Activity type (walking; running; cycling; other) | Indirect Calorimetry; Wearable (Fitbit one (waist)); Observation (Direct) | Equivalence tests of paired means; two one-sided tests; Paired sample t tests; Mean percentage errors; Confusion matrix | The health watch can serve its medical purpose of measuring resting heart rate and total energy expenditure over time in an unobtrusive manner, thereby providing valuable data for the prevention and management of lifestyle-related chronic diseases. | Y/Y | High/High |
| 195 | Hergenroeder et al. (2018, United States) | Adults (N=43; 87±5.7 yrs; 81.4% females; White (N=43)) | Walking (200 steps) | *ActiGraph GT3X* (waist; NR; NR; NR); *ActivPAL* (right thigh; NR; NR; NR); *StepWatch Activity Monitor* (ankle; NR; NR; NR) | Intensity (Steps) | Observation (Manually counted) | Accuracy; Kruskal–Wallis test | The StepWatch and ActivPAL monitor were reasonably accurate in measuring steps in older adults who walk slowly and use an assistive device. The Actigraph significantly undercounted steps in those who walk slow or use an assistive device. | N/N | High |
| 196 | Hergenroeder et al. (2019, United States) | Adults (N=43; 87±5.7 yrs; 81.4% females; White (N=43)) | Walking (200 steps) | *Fitbit Charge* (dominant wrist; NR; NR; NR); *Garmin Vivofit* (non-dominant wrist; NR; NR; NR); *Fitbit Zip* (pocket/ neckline, waist; NR; NR; NR); *Yamax SW-200 Digiwalker* (waist; NR; NR; NR); *Accusplit AX2710* (waist; NR; NR; NR); *Yamax EX-510* (waist; NR; NR; NR); *Omron HJ- 321* (waist; NR; NR; NR) | Intensity (Steps) | Observation (Manually counted) | Accuracy | This study identified the limitations of the current commercial activity monitors in both step counting accuracy and usability features for older adults. | N/NR | High |
| 197 | Herkert et al. (2019, Netherlands) | Adults with heart failures and coronary artery disease (N=38; 63.3±6.8 yrs; 28.9% females; NR) | Treadmill (9 minutes); Bike ergometer (9 minutes); Stairs (2 minutes); Activities of daily life (12 minutes); Postures (7 minutes) | *Fitbit Charge 2* (dominant wrist; Fitbit app; NR; NR); *Mio Slice* (non-dominant wrist; Mio app; NR; NR) | Intensity (Energy expenditure) | Indirect Calorimetry | Accuracy; One-sample t-tests; Bland-Altman analyses | Both activity trackers demonstrated low accuracy in estimating EE in cardiac patients and poor performance to detect within-patient changes in the low-to-moderate exercise intensity domain. | N/N | High |
| 198 | Herman Hansen et al. (2014, Norway) | Adults (N=20; 28.2±3.3 yrs; NR; NR) | Treadmill (70 minutes); Bike ergometer (30 minutes) | *ActiGraph GT1M* (right hip, Actilife software v.4.1.1; 10 sec; NR) | Intensity (Activity counts) | Indirect Calorimetry | Linear regression; Generalised linear models; paired t-tests | The GT1M is a valid tool for assessing walking across a wide range of speeds and gradients. However, there is no relationship between activity counts and energy expenditure during cycling and physical activity is underestimated by ≈73% during cycling compared to walking. | NR/NR | High |
| 199 | Hernández-Belmonte et al. (2018, Spain) | Adults (N=10; NR; 0% females; NR) | Walking (483.84 meters) | *WIMU PRO* (upper back; S PRO software; NR; AcelT equation) | Intensity (Steps) | Observation (Video) | Pearson correlation; Intraclass correlation coefficient; Bland-Altman analyses | WIMU PRO is a reliable and valid device to quantify physical activity level through steps measurement. | N/Y | Some |
| 200 | Herrmann et al. (2011, United States) | Adults (N=16; 40.2±12.6 yrs; 50% females; NR) | Treadmill (8 minutes) | *MyWellnessKey* (waist; MyWellness Key web-based software; NR; proprietary algorithm) | Intensity (Energy expenditure) | Wearable (ActiGraph GT1M (waist); Yamax Digiwalker SW-200 (waist)) | Spearman correlation; partial Spearman correlations | The MyWellness Key has a high concurrent validity with the ActiGraph accelerometer to detect PA in both controlled laboratory and free-living settings. | Y/N | High |
| 201 | Hibbing et al. (2018, United States) | Adults (N=30; 23.0±2.3 yrs; 33% females; NR) | Postures (7 minutes); Activities of daily life (21 minutes); outdoor walking (21 minutes); Stairs (7 minutes); Exercise (14 minutes) | *ActiGraph GT9X* (right hip, both ankles, both wrists; NR; 1 sec; two-regression algorithms (ENMO only; ENMO and GVM; ENMO; GVM an direction changes)) | Intensity (Energy expenditure) | Indirect Calorimetry | Root mean square error; Mean absolute percent error; Equivalence testing; two one-sided t-tests | The combined use of gyroscope and accelerometer at the hip and ankles improved individual-level prediction of EE compared with accelerometer only. For the wrists, adding gyroscope produced negligible changes. | N/N | Low |
| 202 | Hickey et al. (2016, United States) | Adults (N=15; 24.9±5.1 yrs; 53.3% females; NR) | Treadmill (20 minutes); Indoor walking (3 minutes); Activities of daily life (23 minutes) | *Omron HJ720-ITC* (waist; NR; NR; NR); *Yamax Digi-Walker SW-200* (waist; NR; NR; NR); *ActiGraph 7164* (waist; NR; NR; NR); *ActiGraph GT3X* (waist; NR; NR; low-frequency extension filter, normal filter); *ActivPAL* (thigh; NR; NR; NR); *StepWatch* (ankle; NR; NR; NR) | Intensity (Steps) | Observation (Video) | Linear mixed model analysis; accuracy; Percent error | This study highlights the need to verify step-counting accuracy of activity monitors with activities that include different movement types/directions. | N/NR | High |
| 203 | Hildebrand et al. (2014, Norway) | Adults (N=30; 34.2±10.7 yrs; 56.6% females; NR) | Postures (10 minutes); Activities of daily life (15 minutes); Treadmill (15 minutes); Stairs (5 minutes) | *ActiGraph GT3X+* (hip, wrist; ActiLife v6.5.2; 1 sec; algorithm reported); *GENEActiv* (hip, wrist; GENEActiv personal computer software v2.2; 1 sec; algorithm reported) | Intensity (Energy expenditure) | Indirect Calorimetry | Repeated-measures ANOVA; Bland-Altman analyses; Linear regression analyses | Accelerometer outputs from AG and GA seem comparable when attached to the same body location in adults, whereas inconsistent differences are apparent between the two brands and placements in children, hence limiting the comparability between brands in this age group. | N/N | Low |
| 204 | Hill et al. (2010, Canada) | Adults with chronic obstructive pulmonary disease (N=26; 67.4±6.8 yrs; 42.3% females; NR) | Postures (18 minutes); Indoor walking (12 minutes) | *SenseWear Armband* (upper right arm; Software v.6.1; NR; proprietary algorithm) | Intensity (Energy expenditure) | Indirect Calorimetry | Repeated-measures ANCOVA; Bland-Altman analyses | EE_SAB_ was sensitive to small but important changes. There was fair agreement between EE_SAB_ and EE_IC_, and measurements of EE_SAB_ were repeatable. These observations suggest that the SAB is useful for the evaluation of EE in patients with COPD who walk without a rollator. | N/N | High |
| 205 | Hiremath et al. (2013, United States) | Manual wheelchair users with spinal cord injury (N=45; 40.2±11.08 yrs; 17.7% females; NR) | Activities of daily life (8 minutes); Wheelchair propulsion (24 minutes), Arm-ergometry (24 minutes) | *SenseWear Armband* (right upper arm; InnerView Research software 7.0; 1 minute; proprietary algorithm) | Intensity (Energy expenditure) | Indirect Calorimetry | Precision; Recall; Specificity; Accuracy; mean absolute error; mean-signed error | The high classification accuracy and low EE estimation errors suggest that the SW can be used by researchers and clinicians to classify and estimate the EE for the four activities tested in this study among MWUs with SCI. | N/Y | Low |
| 206 | Ho et al. (2019, Taiwan) | Adults (N=90; 22.90±4.2 yrs; 45.5% females; NR) | Treadmill (15 minutes) | *ActiGraph GT9X* (wrist, waist; ActiLife v6.12.1; 10 sec; algorithm reported) | Intensity (Energy expenditure) | Indirect Calorimetry | One-way ANOVA; Cohen's d effect size; Mean Absolute Percentage Error; Pearson coefficient of determination; linear regression | The EE estimation equation combining the VM of accelerometer measurements, BW and HRR greatly enhanced the accuracy of EE estimation based on data from accelerometers worn in different positions, particularly from those on the wrist. | N/N | Some |
| 207 | Höchsmann et al. (2018, Switzerland) | Adults (N=20; Older adults: 53 yrs (median); Young adults: 22 yrs (median); 70% females; NR) | Treadmill (20 minutes); Walking (620.6 meters) | *Garmin Vivofit 2* (non-dominant wrist; NR; NR; NR); *ActiGraph wGT3X+* (non-dominant wrist, hip; ActiLife v6.13.3; NR; NR) | Intensity (Steps) | Observation (Video) | Linear mixed effects model; Mean absolute percentage error | The Garmin Vivofit 2 is an accurate tool for step counting in different age groups and during various walking conditions, even during slow walking. | N/N | High |
| 208 | Holbrook et al. (2009, United States) | Adults (N=47; 24±4.4 yrs; 48.9% females; NR) | Outdoor walking (600 meters) | *Omron HJ-151* (right hip, left hip, back; NR; NR; NR); *Omron HJ-720ITC* (right hip, left hip, back, pockets, backpack; NR; NR; NR) | Intensity (Steps) | Observation (Manually counted) | Absolute percent error; repeated-measures ANOVA | The Omron HJ-151 and HJ-720ITC pedometers demonstrated validity and reliability at various mounting positions under prescribed and self-paced walking conditions with both healthy and overweight adults. | NR/NR | High |
| 209 | Horemans et al. (2019, Netherlands) | Adults (N=12; 19-36 yrs; 66.6% females; NR) | Activities of daily life (60 minutes) | *Activ8* (pocket, right thigh; NR; NR; Activ8 algorithm) | Posture/Activity type (Sitting/lying, standing, walking, cycling, running) | Observation (Video) | Agreement; Sensitivity; Positive predictive value; Absolute time difference | The Activ8 is a valid instrument to quantify a defined set of body postures and movements. Because of the smaller time difference, the thigh location is preferred for research purposes. | N/N | High |
| 210 | Horner et al. (2011, United Kingdom) | Adults (N=11; 22±3 yrs; 54.5% females; NR) | Treadmill (32 minutes) | *3DNX model v3* (lower back; dedicated software; 5 sec; proprietary algorithms) | Intensity (Counts) | Indirect Calorimetry | Linear regression analysis; Bland-Altman analyses; Intra-class correlation coefficients | The 3DNX accelerometer is a reliable and valid device for measuring acceleration in a mechanical setting and during human treadmill exercise. | N/Y | High |
| 211 | Horner et al. (2013, United Kingdom) | Adults (N=26; 20.4±1.3 yrs; 100% females; NR) | Treadmill (32 minutes) | *3DNX model v3* (hip, lower back; dedicated software; 5 sec; NR) | Intensity (Counts) | Indirect Calorimetry | Multiple linear regression analysis; ANCOVA; ANOVA | The lower back is a more suitable accelerometer placement for young, active females during treadmill exercise. | NR/NR | High |
| 212 | Horvath et al. (2007, Canada) | Adults (N=20; 27.8±8.8 yrs; 45% females; NR) | Treadmill and walking (NR) | *Yamax Digiwalker SW200* (five at waist; NR; NR; NR) | Intensity (Steps) | Observation (Manually-counted) | Percentage error; Repeated-measures ANOVA | Pedometer position dependent error was demonstrated, with the left mid-axillary position superior to the recommended position of right mid-thigh. The greater accuracy on the left side was wholly explained by gait asymmetry evident in step-induced accelerations recorded at right and left pedometer positions. | N/NR | High |
| 213 | Huang et al. (2016, China) | Adults (N=40; 23.9±2.8 yrs, 25% females; NR) | Walking and treadmill (Approx. 45 minutes) | *Nike+ FuelBand SE* (wrist; NR; NR; NR); *Jawbone UP 24* (non-dominant wrist; NR; NR; NR); *Fitbit One* (waist; NR; NR; NR); *Fitbit Flex* (wrist; NR; NR; NR); *Fitbit Zip* (waist; NR; NR; NR); *Garmin Vivofit* (wrist; NR; NR; NR); *Yamax CW-701* (waist; NR; NR; NR); *Omron HJ-321* (waist; NR; NR; NR) | Intensity (Steps) | Observation (Video) | Percentage error; One-way ANOVA; Bland–Altman analyses | In general, there were not accuracy differences among activity monitors for stair walking. Accuracy did not change between moderate and fast walking speeds, though slow walking increased errors for some activity monitors. Nike+ FuelBand was the least accurate step count estimator during all walking tasks. | N/N | High |
| 214 | Huberty et al. (2018, United States) | Adults (N=22, 30±11 yrs, 77% females, NR) | Yoga session (30 minutes) | *ActiGraph* *GT3X+* (left hip, Actilife software; 1 minute; Freedson algorithm), *GENEActiv* (non-dominant wrist, GENEActiv software; 1 minute; NR) | Intensity (Energy expenditure) | Indirect Calorimetry | Intraclass correlation coefficient, mean difference, mean absolute percent error, root mean square error, Bland-Altman analyses | According to the Oxycon, participation in Vinyasa Flow met the criteria for moderate-intensity physical activity. The AG and GA consistently underestimated EE. More research is needed to determine an accurate measurement for EE during yoga using a wearable device appropriate for free-living environments. | NR/NR | High |
| 215 | Husted & Llewellyn (2017, United States) | Adults (N=12; 20.8±0.94 yrs; 91.67% females; NR) | Treadmill (5 minutes) | *Fitbit Charge* (right wrist; NR; NR; NR); *Smart Health* (left wrist; NR; NR; NR); *Omron HJ-303™* (right hip; NR; NR; NR); *Sportline™* (left hip; NR; NR) | Intensity (Steps) | Observation (Video) | Independent t-tests | These results suggest an inverse relationship between cost and accuracy for the four specific brands tested, and that waist pedometers are more accurate than wrist pedometers. The results concerning the Fitbit are striking considering its high cost and popularity among consumers today. | NR/NR | High |
| 216 | Hustved et al. (2004, Norway) | Adults, Study 1 (N=10; 23.3±2.1 yrs; 60% females; n.a), Study 2 (N=24; 24±2 yrs; 100% females; NR) | Activities of daily life and treadmill (8 hrs 45 minutes) | *ActiReg* (chest, right thigh; ActiCalc; NR; regression equation) | Intensity (Energy expenditure) | Indirect Calorimetry | Bland-Altman analyses; t-test | The objective recording of the time spent in different body positions and at different levels of PA may be useful in studies of PA in different groups and in studies of whether recommendations for PA are being met. The comparative ease of data collection and calculation should make ActiRegw a useful instrument to measure habitual PA level and EE. | NR/NR | Some |
| 217 | Ichinoseki-Sekine et al. (2006, Japan) | Adults from rehabilitation services center (N=49; 80.9±7.7 yrs; 75.4% females; NR) | Walking (20 meters) | *Omron HJ-720IT* (right waist; NR; Fast Fourier transform) | Intensity (Steps) | Observation (Manually counted) | Error; One-way ANOVA; Paired or non-paired t-tests; Correlation | Authors suggest that the FFT method is suitable for estimating the number of steps during walking in this population. | N/NR | High |
| 218 | Imboden et al. (2018, United States) | Adults (N=30; 49.2±19.2 yrs; 50% females; NR) | Activities of daily life, walking and treadmill (80 minutes) | *Fitbit One* (waist; NR; NR; NR); *Fitbit Zip* (waist; NR; NR; NR); *Fitbit Flex* (non-dominant wrist; NR; NR; NR); *Jawbone UP 24* (non-dominant wrist; NR), *ActiGraph GT3X +* (right hip; ActiLife 6; 30 sec; Work-energy theorem and Freedson equation) | Intensity (Energy expenditure; Steps) | Indirect open-circuit Calorimetry; observation (Manually-counted) | Repeated measures ANOVA; Bland-Altman analyses; Correlation; Mean absolute perecentage error | Consumer monitors had similar accuracy for PA assessment as the ActiGraph, which suggests that consumer monitors may serve to track personal PA behaviours and EE. | N/N | Low |
| 219 | Jakicic et al. (2004, United States) | Adults (N=40; 23.3±3.8 yrs; 50% females; NR) | Treadmill (30 minutes); Exercise (60 minutes) | *SenseWear Pro* (right arm; Innerview Research Software v3.3; 1 minute; proprietary algorithms) | Intensity (Energy expenditure) | Indirect Calorimetry | Intraclass correlation coefficients; Two-factor repeated measures ANOVA; dependent t-tests; Bland–Altman analyses | It appears that it is necessary to apply exercise-specific algorithms to the SenseWear Pro Armband™ to enhance the accuracy of estimating energy expenditure during periods of exercise. | Y/NR | High |
| 220 | Jayaraman et al. (2016, United States) | Adults (N=10; 27.1±1.6 yrs; 40% females; NR); Adults with spinal cord injury (N=8; 48.5±3.7 yrs; 13% females, NR) | Posture (2 minutes); Walking (6 minutes + 50 steps) | *ActiGraph GT3X-BT* (right arm, waist, ankle; Actilife software; 10 sec; Choi algorithm, Freedson’s algorithm); *Metria-IH1* (left arm, SenseWear platform; NR; NR) | Intensity (Energy expenditure; Steps) | Indirect Calorimetry; Observation (Manually counted) | One-way ANOVAs | Results suggest that Metria-IH1 out-performed ActiGraph-GT3X in estimating EE during sedentary activity in both groups. The device location and population demographics, significantly affected the accuracy of predicted estimates. | NR/NR | Some |
| 221 | Jayaraman et al. (2018, United States) | Adults (N=10; 27.1±5.1 yrs; 40% females; NR); Adults with spinal cord injury (N=8; 48.5±10.4 yrs; 13% females; NR); Adults with stroke (N=10; 55.6±9.4 yrs; 40% females; NR) | Postures (8 minutes); Walking (27 minutes) | *ActiGraph wGT3X-BT* (right arm, waist, ankle; ActiLIfe software; NR; Choi proprietary algorithm, Freedson proprietary algorithm); *Metria-IH1* (left arm; SenseWear software; NR; proprietary fusion algorithm) | Intensity (Energy expenditure; Steps) | Indirect Calorimetry; Observation (Manually counted) | Epsilon-squared effect size; Kruskal Wallis tests | Sensor type, sensor location, activity characteristics and the population specific condition influences the validity of estimation of physical activity metrics using standard proprietary algorithms. | N/N | Low |
| 222 | Jean-Louis et al. (1996, United States) | Adults (N=20; 29.95±8.98 yrs; 45% females; NR) | Sleep (1 night) | *Gaehwiler Electronic no. CH-8634* (dominant wrist; Actigraph Data Analysis Software; 1 minute; NR) | Biological state (Total sleep time) | Polysomnography | Frequency analysis; Pearson correlation | Overall agreement between actigraphy and PSG of 97%, with a mean discrepancy value of only 13 minutes for the validation sample. | N/NR | High |
| 223 | Jean-Louis et al. (1999, United States) | Adults with insomnia (N=26; 46.46±10.82 yrs; NR; NR) | Sleep (1 night) | *Actigraph* (NR, Actigraph Data Analysis Software; 1 minute; computer algorithms) | Biological state (Total sleep time) | Polysomnography | Frequency analysis; Pearson correlation | A strong correlation coefficient was noted between ACT and PSG for total sleep time, thus suggesting a high degree of accuracy of the actigraph methodology in assessing the sleep/wake profile of insomniacs. | N/NR | High |
| 224 | Jean-Louis et al. (2000, United States) | Adults with major depressive episodes (N=24; 45±9 yrs; 29% females; NR) | Sleep (1 night) | *Gaehwiler Electronic monitor* (wrist; Actigraph Data Analysis Software; 1 minute; two-step algorithm) | Biological state (Total sleep time) | Polysomnography | Bland–Altman analyses | Normative algorithm yielded a correlation oefficient of 0.85 and an average error of 35 min, comparing actigraphic and polysomnographic sleep estimates. These findings suggest that scoring criteria optimized on wrist-activity data of healthy young adults may not produce optimal results for patients characterized with major depressive episodes. | N/NR | Some |
| 225 | Jean-Louis et al. (2001, United States) | Adults (N=11; 35.36±5.66 yrs; 64% females; NR) | Sleep (5 nights) | *Actillume recorder* (wrist; ACTION3 software; 1 minute; three-step algorithm) | Biological state (Total sleep time) | Polysomnography | Agreement; Sensitivity; Specificity; Pearson correlation; Measurement error | Strong correlations and agreements between sleep estimates from Actillume and PSG suggest that the Actillume can reliably monitor sleep and wakefulness in the laboratory | N/NR | Some |
| 226 | Jehn et al. (2010, Switzerland) | Adults with chronic heart failure (N=97; 60.7±13.4 yrs; 23% females; NR) | Treadmill (30 minutes), outdoor walking (30 minutes), Indoor walking (6 minutes) | *Omron HJ-720ITC* (right hip, NR; 1 minute; NR) | Intensity (Steps) | Observation (Manually-counted) | Pearson correlation; Mean difference; Two-way analysis of variance for repeated measurements, ANOVA, unpaired Student’s t-test, Chi^2^ test | The Omron HJ-720ITC pedometer is accurate for monitoring activity in individuals with normal walking behaviour, but seems unsuitable for chronically ill patients characterised by slow walking gaits. | NR/NR | High |
| 227 | Jiang & Larson (2013, United States) | Adults (N=30; 51.6±17.8 yrs.; 53.3% females; Asians (N=15), African Amercians (N=9), Causcasians (N=6)) | Activities of daily life (approx. 4-5 hours) | *IDEEA system* (each thigh, each foot, chest; IDEEA software; NR; NR) | Posture/Activity Type (Sitting; lying; standing) | Observation (Direct) | Pearson correlation; Chi-square test; t-tests | The IDEEA system accurately recognizes sitting and standing positions, but it is less accurate in identifying lying and reclining positions | NR/NR | High |
| 228 | John et al. (2018, United States) | Adults (N=20; 26.7±4.9 yrs.; 40% females, NR) | Treadmill (11 minutes) | *Yamax Digiwalker SW200* (hip; NR; NR; NR); *Omron HJ720ITC* (hip; NR; NR; NR), *ActiGraph GT3X+* (hip, wrist; ActiLife software v6.13.3; NR; NR); *ActiGraph GT9X* (hip, wrist; ActiLife software v6.13.3; NR; NR) | Intensity (Steps) | Observation (Manually counted) | Estimation bias | Three common research-grade physical activity monitors employ different step-detection strategies, which causes variability in step output. | N/N | High |
| 229 | Johnson (2015, United States) | Adults (N=29; 67.70±6.07 yrs.; 41% females; NR) | Indoor walking (200 meters); Treadmill (15 minutes) | *Actical accelerometer* (right hip; manufacturer recommended software; NR; NR); *Yamax SW-200* (right hip; NR; NR; NR); *Omron HJ-112* (right hip; NR; NR; NR); *Walk4Life Elite* (right hip, NR; NR; NR) | Intensity (Steps) | Observation (Manually counted) | One-way repeated measure ANOVA; Mean absolute percent error; Bland-Altman analyses | Activity monitor selection should be deliberate when examining the walking behaviors of community-dwelling older adults, especially for those who walk at a slower pace. | N/N | High |
| 230 | Johnson et al. (2015; United States) | Adults (N=43; 20.98±1.17 yrs.; 53% females; NR) | Indoor walking (200 meters); Treadmill (30 minutes) | *Actical accelerometer* (right hip; manufacturer-recommended software; 30 sec; NR); *Yamax SW-200* (right hip; NR; NR; NR); *Omron HJ-112* (right hip; NR; NR; NR); *Walk4Life Elite* (right hip, NR; NR; NR) | Intensity (Steps; Energy expenditure) | Observation (Manually counted); Indirect Calorimetry | One-way ANOVA; Mean absolute percent error; Bland-Altman analyses; two-way repeated measures ANOVA; paired sample t-test | The Actical provides valid estimates of step counts at self-selected pace and walking at constant speeds of 1.56 and 2.01 m ·s–1. The Actical underestimates EE of walking at constants speeds ≥ 1.38 m · s–1. | NR/NR | High |
| 231 | Jones et al. (2018, Australia) | Adults (N=30; 33±8 yrs; 60% females; NR) | Treadmill (approx. 28 minutes) | *Fitbit Flex* (wrist; web Fitbit interface; 1 minute; NR); *ActiGraph GT3X+* (waist; Actilife v5.10.0; 1 minute; NR) | Intensity (Steps) | Observation (Video) | Correlation coefficient; Mean Absolute Percentage Error | Fitbit Flex and ActiGraph GT3X+ provide a valid account of steps taken at jogging and running speeds up to 14 km/hr, attainable by non-elite runners on a treadmill. | N/N | High |
| 232 | Jung et al. (2020, NR) | Adults (N=32; 26.03±6.59 yrs; 53.13% females; NR) | Treadmill (25 minutes); Activities of daily life (30 minutes); Outdoor walking (10 minutes); Exercise (5 minutes) | *Fitbit Charge HR* (wrist; Fitbit Android application; NR; NR) | Intensity (Steps; Energy expenditure) | Indirect Calorimetry; Observation (Video); Wearable (ActiGraph GT3X; waist) | Repeated measures ANOVAs; Mean difference | The accuracy of Fitbit HR measurements against the criterion measure was moderate. Fitbit HR overestimated the calories regardless of their positions and activity types. | N/N | High |
| 233 | Kahawage et al. (2019, Australia) | Adults with chronic insomnia (N=42; 49±17.54 yrs; 55% females; NR) | Sleep (1 night) | *Actiwatch Spectrum Pro* (non-dominant wrist; Actiware 6.0; 30 sec; NR); *Fitbit Alta HR* (non-dominant-wrist; firmware 26.63.2; 30 sec; NR) | Biological state (Sleep/week algorithm) | Polysomnography | Sensitivity; Specificity; Accuracy; Bland-Altman analyses | Both devices were more accurate in detecting sleep than wake, with equivalent sensitivity, but statistically different specificity. FBA provided equivalent estimates as AWS for all traditional actigraphy sleep parameters. FBA also showed high specificity when identifying N3, and rapid eye movement, though sensitivity was modest. | Y/Y | High |
| 234 | Kamper et al. (2016, United States) | Traumatic brain injury patients (N=50; 37.5±16.3 yrs; 8% females; 80% White) | Sleep (1 night) | *Actiwatch 2, Actiwatch Spectrum* (all non-dominant wrist; Phillips Actiware 6; 1 minute; 40 counts per epoch) | Biological state (Total sleep time) | Polysomnography | Pearson correlation; Bland-Altman analyses; Wilcoxon tests | Actigraphy is a valid proxy for monitoring of sleep in this population across injury severity and common comorbidity groups. | N/N | High |
| 235 | Kanady et al. (2010, United States) | Adults (N=57; (nap group: 20.4±2.3 yrs; 70% females; NR); no-nap group: 20.1±2.8 yrs; 93% females; NR) | Sleep (90 minutes nap) | *Actiwatch-64* (wrist; Respironics Actiware 5.52.0003; 1 minute; automatic minor rest interval algorithm) | Biological state (Total sleep time) | Polysomnography | Linear regressions; Bland-Altman analyses; Accuracy, Sensitivity; Specificity; Kappa statistics | Results suggest that actigraphy can predict TST, SE and SL reliably, depending upon parameter settings, and actigraphy is a highly sensitive but not specific measure for daytime naps. | Y/Y | High |
| 236 | Kanady et al. (2020, United States) | Adults (N=39; 26.8±3.4 yrs; 88.2% females; NR) | Sleep (3 nights) | *Basis B1* (non-dominant wrist; NR; 30 sec; proprietary algorithms); *Micro* *Motionlogger Watch* (non-dominant wrist, NR; NR; Cole-Kripke algorithm) | Biological state (Total sleep time) | Polysomnography | Accuracy; Sensitivity; Specificity; Pearson correlations; Bland-Altman analyses | Basis B1 demonstrated utility for estimates of gross sleep parameters and performed similarly to actigraphy for estimates of total sleep time. Basis B1 specificity was poor, and Basis B1 is not useful for the assessment of wake. Basis B1 accuracy for sleep stages was better than chance but is not a suitable replacement for PSG assessment. | Y/N | Some |
| 237 | Kane et al. (2010, United States) | Adults (N=20; 24.1±4.0 yrs; 45% females; NR) | Treadmill (48 minutes) | *Nike+* (in-shoe, NR; NR; proprietary algorithms) | Intensity (Energy expenditure) | Indirect Calorimetry | Repeated measues ANOVA | Nike + ® in-shoe device provided reasonable estimates of speed and distance during level running at the three speeds tested in this study. However, it overestimated EE during level walking and it did not detect the increased cost of inclined locomotion. | NR/NR | High |
| 238 | Kapella et al. (2017, United States) | Adults wirh mild-to-severe chronic obstructive pulmonary disease (N=50; 63.2±8.4 yrs; 30% females; 56% White, 40% Black, 2% Asian, 2% American Indian or Alaskan) | Sleep (1 night) | *Actiwatch-2* (non-dominant wrist; Actiware 6.0.8; 30 sec; NR) | Biological state (Total sleep time) | Polysomnography | Paired t tests; Bland-Altman analyses; Pitman's test of difference | Results support the conclusion that the default actigraphy settings may not be optimal for people with chronic obstructive pulmonary disease and co-existing insomnia. | N/N | High |
| 239 | Kaplan et al. (2012, United States) | Adults with bipolar disorder (N=27; 33.1±10.3 yrs; 85.2% females; Caucasian (N=19), Non-caucasian (N=8); Healthy adults (N=27; 38.1±13.0 yrs; 70.4% females; Caucasian (N=15), Non-caucasian (N=12)) | Sleep (2 nights) | *Actiwatch-64* (wrist; Actiware 5.57; 30 sec; Actiware low threshold algorithm, Actiware medium threshold algorithm, Actiware high threshold algorithm) | Biological state (Total sleep time) | Polysomnography | Two-way repeated measures ANOVA; Pearson correlation; Bland–Altman analyses | Actigraphy is a valid tool for estimating sleep length and fragmentation in bipolar disorder. | N/N | High |
| 240 | Karabulut et al. (2005, United States) | Adults (N=20; 28±3.7 yrs; 50% females; NR) | Treadmill (18 minutes) | *Yamax Digiwalker SW-701* (right waist; NR; NR); *New-Lifestyles NL-2000* (left waist; NR; NR); *StepWatch 3* (right ankle; NR; 1 minute; NR); *Dynastream* *AMP 331* (left ankle; NR; 1 minute; smart stride detection algorithm) | Intensity (Steps) | Observation (Manually-counted) | One-way repeated measures ANOVA; Modified Bland-Altman analyses | The SW-3Ankle has superior accuracy at slow treadmill walking speeds (although it was also more likely to detect ‘‘fidgeting’’ activities). | Y/N | High |
| 241 | Karaca et al. (2021, Turkey) | Adults (N=29; 26.3±6.2 yrs; 0% females; NR) | Treadmill (8 minutes) | *ActiGraph wGT3X-BT* (each wrist, waist, right ankle, right upper arm; ActiLife 6; 5 sec; NR) | Intensity (Steps) | Observation (Video) | Dependent t-test; Bland-Altman analyses; Mean difference; Pearson correlation; Mean absolute percentage error | The waist and right upper arm are valid for the total step counts during walking and running in the laboratory setting. | N/N | High |
| 242 | Karinharju et al. (2019, Australia, Finland) | Wheelchair users (N=26; 42±13 yrs; 23% females; NR) | Activities of daily life (Approx. 1-2 hours) | *Apple Watch Series 1* (dominant wrist; Apple Activity® Application; NR; NR) | Intensity (Push counts) | Observation (Video) | Intraclass correlation coefficients; Pearson correlation; Bland-Altman analyses; Mean absolute percentage error | Apple Watch® push-count estimates are acceptable for personal, self-monitoring purposes and for research entailing group-level analyses, but less acceptable where accurate push-count measures for an individual is required | N/N | High |
| 243 | Kastelic et al. (2021, Slovenia) | Adults (N=28; 74.3±4.9 yrs; 53.6% females; NR) | Activities of daily life and walking (20 minutes) | *Polar Vantage M* (wrist; Polar Vantage M v.5.0.10; NR; manufacturers’ algorithms); *Garmin Vivoactive 4s* (wrist; Vivoactive 4s v4.70; NR; NR); *Garmin Vivosport* (wrist; Vivoactive 4s v4.20; NR; NR) | Intensity (Steps) | Observation (Video) | Intraclass correlation coefficient; Mean error; Mean percentage error; Mean absolute percentage error; Bland-Altman analyses | Results suggested that all three trackers could be used for measuring sleep time with a high level of accuracy, and both Garmin trackers could also be used for step counts. | N/N | High |
| 244 | Kayes et al. (2009, New Zealand) | Adults with Multiple Sclerosis (N=31; Mean 50 yrs; 68% females; 94% White, 3% Maori, 3% Pacific Islander) | Walking and activities of daily life (NR) | *Actical accelerometer* (waist; Actical manufacturer’s software; 15 sec; NR) | Intensity (Counts) | Wearable (Polar S810i (chest)) | One-way ANOVA; Bland-Altman analyses; Intraclass correlation coefficient | The psychometric problems highlighted here suggest Actical accelerometers should be used with caution in people with MS as a measure of physical activity, particularly when measuring comparatively sedentary or free-living activities | N/N | High |
| 245 | Keating et al. (2018, United States) | Adults (N=17; 20.35±0.86 yrs; 76.5% females; Chinese (N=17)) | Walking (NR) | *Fitbit Charge 2* (non-dominant wrist; FitBit App; NR; NR) | Intensity (Steps) | Wearable (Yamax SW-200 Pedometer (hip)) | Absolute percent error; One sample t-test; Linear mixed model analysis | FC2 revealed promising validity evidence measuring total steps in a controlled setting | NR/NR | High |
| 246 | Kelly et al. (2013, United States) | Adults (N=42; 21.57±2.73 yrs; 45.2% females; NR) | Treadmill (18 minutes) | *ActiGraph GT1M* (right hip; NR; NR; NR); *ActiGraph GT3X* (right hip; NR; NR; NR) | Intensity (Counts) | Indirect Calorimetry | Pearson correlation; Bland-Altman analyses | The present study showed that both the GT1M and the GT3X accurately measure physical activity when compared to oxygen consumption. | N/N | High |
| 247 | Kemp et al. (2020, South Africa) | Adults (N=50; 29.5±18 yrs; 44% females; NR) | Activities of daily life, treadmill, cycling, and stepping (Approx. 60 minutes) | *Actiwatch 2* (non-dominant wrist; Philips Actiware v6.0.2; 15 sec; NR) | Intensity (Energy expenditure (MET); Counts) | Indirect Calorimetry; Wearable (ActiGraph GT3X (waist)) | Spearman correlation; Sensitivity; Specificity; Youden’s J statistic; Bland–Altman analyses | The use of the AW2 in physical activity monitoring looks promising for sedentary behavior, moderate and vigorous activity, however, further validation is needed. | N/N | Some |
| 248 | Kendall et al. (2019, United States) | Adults (N=50; 25.84±8.10 yrs; 50% females; 60% Caucasian) | Treadmill (NR) | *Polar HR Monitor* (chest, NR; NR; NR); *Basis watch* (right wrist, NR; NR; NR); *Fitbit Flex* (left wrist, NR; NR; NR); *Omron HJ-321* (waist; NR; NR; NR); *Jawbone UP move* (waist; NR; NR; NR); *ActiGraph wGT3X-BT* (waist; NR; 10 sec; NR) | Intensity (Energy expenditure; Steps) | Indirect Calorimetry; Observation (Manually-counted) | Intraclass correlation; One-sample t-tests | When comparing low and high fit groups, correlations between AMs and indirect Calorimetry improved for the low fit group, suggesting AMs may be better at measuring EE at lower intensity exercise. | NR/N | High |
| 249 | Keppler et al. (2019, Germany) | Adults (N=20; 75.5±7.82 yrs; 95% females; NR) | Walking (NR) | *Actibelt* (waist, NR; NR; stepslc, stepwave) | Intensity (Steps) | Observation (Video) | Mean deviations; t-test, Bland-Altman analyses | With the described setup, algorithms for step and gait speed detection have successfully been validated in an elderly population and demonstrated to have improved performance versus previously published algorithms. | N/N | High |
| 250 | Kim & Welk (2015, United States) | Adults (N=52; 23.8±5.2 yrs; 36.5% females; NR) | Activities of daily life (75 minutes) | *ActiGraph GT3X+* (right hip; Actilife v6.5.1; 1 minute; Freedson equation); *ActivPAL* (right thigh, Intelligent Activity Classification v7.2.32; 15 sec; NR); *Core Armband Monitor* (non-dominant upper arm; SenseWear Professional v8; NR; proprietary algorithm) | Intensity (Energy expenditure (MET)) | Indirect Calorimetry | Pearson correlation; Mean absolute percent errors; Equivalence testing; Mixed model ANOVA | The CA showed good agreement relative to the OM for the overall group comparison and for moderate and vigorous activities. The AP, in contrast, was the most accurate for sedentary and light activities. The combined use of the CA and AP may yield more accurate estimates of EE than using a single monitor. | Y/NR | Low |
| 251 | Kim et al. (2021, South Korea) | Adults (N=170; 41.6±13.4 yrs; 12.4% females; NR) | Sleep (1 night) | *Mi Band 2* (non-dominant wrist; Mi Fit 4.0.15; NR; NR); Samsung *Gearfit 2* (non-dominant wrist; Samsung Health 6.8.5.009; NR; NR); *Fitbit Alta HR* (non-dominant wrist; Fitbit 6.4.2; NR; NR) | Biological State (Total sleep time) | Polysomnography | Student´s t test; Spearman correlation; Intraclass correlation coefficient; Bland-Altman analyses | Wearable devices mildly correlated with PSG | N/N | High |
| 252 | King et al. (2004, United States) | Adults (N=21; males: 25.2±4.5 yrs; females: 24.7±5.4 yrs; 52.4% females; NR) | Treadmill (70 minutes) | *CSA* (waist; NR; 1 minute; equation reported); *TriTrac-R3D* (waist; NR; NR; NR); *RT3* (waist; NR; NR; proprietary algorithms); *Biotrainer-Pro* (waist; NR; NR; NR); *SenseWear Armband* (upper arm; NR; NR; proprietary algorithms) | Intensity (Energy expenditure) | Indirect Calorimetry | Two-way mixed MANOVA; Pearson correlation; T-tests | The CSA was the best estimate of total EE at walking and jogging speeds, the TriTrac-R3D was the best estimate of total EE at running speeds, and the SenseWear Armband was the best estimate of total EE at most speeds. | N/N | Some |
| 253 | Klassen et al. (2016, Canada) | Post-stroke patients (N=43; 65±10.66 yrs; 30% females; NR) | Walking (120 meters) | *Fitbit One* (waist, ankle; NR; NR; proprietary algorithms) | Intensity (Steps) | Observation (Video) | Linear mixed model; Error rate | Although not recommended by the manufacturer, positioning the accelerometer at the ankle (compared with the waist) may fill a long-standing need for a readily available device that provides accurate feedback for the altered and slow walking patterns that occur with stroke. | N/NR | High |
| 254 | Klassen et al. (2017, Canada) | Stroke rehabilitation patients (N=21; 55±10 yrs; NR; NR) | Physical therapy session (1 hour) | *Fitbit One* (ankle; NR; NR; proprietary algorithms) | Intensity (Steps) | Wearable (StepWatch Activity Monitor (ankle)) | Linear mixed model; Error rate; Bland-Altman analyses | Study provides preliminary evidence that the Fitbit One, when positioned on the nonparetic ankle, can accurately measure walking steps early after stroke during inpatient rehabilitation physical therapy sessions. | N/N | High |
| 255 | Klenk et al. (2016, Germany) | Adults (N=53; 75.3±4.6 yrs; 41.5% females; NR) | Activities of daily life (Approx. 15 minutes) | *ActivPAL 3* (left thigh; activPAL process and presentation software v7.2.32; NR; NR) | Posture/Activity type (Lying/sitting, standing, walking) | Wearable (ActivPAL (thigh)) | Bland-Altman analyses; Percentage agreement | ActivPAL and activPAL3 showed good agreement in older adults. However, if using these devices interchangeably, observed differences might still bias results. | NR/NR | High |
| 256 | Koehler et al. (2011, Germany) | Athletes (N=14; 30.4±6.2 yrs; 0% females; NR) | Treadmill and bicycle ergometer (NR) | *SenseWear Pro3* (right upper arm; SenseWear Professional software v6.1; 1 minute; manufacturer’s algorithm) | Intensity (Exercise energy expenditure) | Indirect Calorimetry | Linear regression; Bland–Altman analyses | The SWA does not provide valid results of TEE and ExEE in endurance athletes because of the underestimation of EE at higher exercise intensities. | N/N | High |
| 257 | Koehler et al. (2013, Germany) | Athletes (N=29; 27±2 yrs; 0% females; NR) | Treadmill and Step-Test (8 minutes) | *SenseWear Pro 3* (upper right arm; SenseWear professional software v6.1; 1 minute; proprietary algorithm); *Actiheart* (chest; standard software by Actiheart; 30 sec; NR) | Intensity (Energy expenditure) | Indirect Calorimetry | Two-sided paired T-test; Linear regression; Bland-Altman analyses | Results show that both devices underestimate EE during high-intensity running. For Actiheart, individual calibration is recommended in order to obtain sufficiently accurate results. For SenseWear, acceleration data could be used to improve EE prediction during high-intensity running. | NR/N | High |
| 258 | Koenders et al. (2018, Netherlands) | Adults (N=31; 18-29 yrs; 0% females; NR) | Posture and walking (30 minutes) | *HealthPatch* (chest; NR; NR; NR) | Posture/Activity Type (Lying, sitting, standing, walking) | Observation (Video) | One sample t-test; Intraclass correlation coefficients; Bland-Altman analyses | Overall, the results show a good validity of the HealthPatch to monitor lying and poor validity to monitor sitting/standing or walking. | N/N | High |
| 259 | Kooiman et al. (2015, Netherlands) | Adults (N=33; Males: 39±13.1 yrs, females: 35±11.2 yrs; 48.5% femals; NR) | Treadmill (60 minutes) | *Lumoback* (lower back; NR; NR; NR); *Fitbit Flex* (non-dominant wrist; NR; NR; NR); *Nike+ Fuelband SE* (non-dominant wrist; NR; NR; NR); *Jawbone Up* (non-dominant wrist; NR; NR; NR); *Misfit Shine* (front pocket; NR; NR; NR); *Withings Pulse* (front pocket; NR; NR; NR); *Fitbit Zip* (front pocket; NR; NR; NR); *Omron HJ-203* (front pocket; NR; NR; NR); *Yamax Digiwalker SW-200* (waist, NR; NR; NR); *ActivPAL* (thigh; NR; NR; NR) | Intensity (Steps) | Device (Optogait system) | Paired samples t-test; Intraclass correlation coefficient; Bland-Altman analyses; Mean absolute percentage errors | The reliability and validity of most trackers for measuring step count is good. The Fitbit Zip is the most valid whereas the reliability and validity of the Nike+ Fuelband is low | N/N | High |
| 260 | Korpan et al. (2015, Canada) | Adults (N=35; 81.5±5.0 yrs; 0% females; NR) | Walking (100 meters) | *ActiGraph GT3X+* (waist, ankle; ActiLife5; 1 sec; Default and LFE algorithm) | Intensity (Steps) | Observation (Manually counted) | Intraclass correlation coefficients; Bland–Altman analyses; One-way Kruskal-Wallis ANOVA | Using the GT3X+ ankle placement and analyzing data with the LFE algorithm resulted in the most accurate step counts in older adults. | N/NR | High |
| 261 | Kosmadopoulos et al. (2014, Australia) | Adults (N=22; 23.9±3.8 yrs; 18% females; NR) | Sleep (2 nights) | *Actiwatch 64* (non-dominant wrist; Actiware v3.4; 30 sec; equation reported); *Actical Z-series* (non-dominant wrist; Actiware v3.4; 30 sec; equation reported) | Biological state (Total sleep time) | Polysonmopgraphy | Agreement; Sensitivity; Specificity; Cohen`s kappa; Positive predictive value; Negative predictive value; Wilcoxon tests; Bland-Altman analyses | Although the partial-PSG system was the most accurate device, both activity monitors were also valid for sleep estimation, provided that appropriate thresholds were selected. | NR/NR | Some |
| 262 | Kossi et al. (2021, France) | Young adults (N=29; 24±4 yrs; 41% females; NR); Older adults (N=27; 71±4 yrs; 74% females; NR) | Cycling and treadmill (15 minutes) | *ActiGraph GT3X+* (wrist, waist, ankle; ActilIfe 5 software v5.7.4; 1 minute; Freedson equation) | Intensity (Energy expenditure) | Indirect Calorimetry | One-way ANOVA; Mean bias of measurement; Intraclass correlation coefficient | The ActiGraph GT3X+ does not provide accurate EE estimates across a range of placement locations during moderate and high-intensity PA. | N/N | Some |
| 263 | Kramer et al. (2018, Australia) | Stroke patients (N=22; Median 78 yrs; 41% females; NR) | Walking and sit-to-stand (12 minutes) | *SenseWear Pro3* (upper arm; NR; 1 minute; proprietary algorithms) | Intensity (Energy expenditure; Steps) | Indirect Calorimetry; Observation (Manually counted) | Intraclass correlation coefficients; Lin’s concordance correlation coefficients; Reduced major axis regression | TheSWA should be used with caution to measure EE of activity of mild to moderate stroke survivors <1 month after stroke. | N/N | High |
| 264 | Kuffel et al. (2011, United States) | Adults (N=30; 28±7.7 yrs; 53% females; NR) | Walking and seated rest (31 minutes) | *ActiGraph GT1M* (right hip, NR; 10 sec; NR; 2006 Crouter 2-Regression Model; Refined Crouter 2-Regression Model) | Intensity (Energy expenditure) | Indirect Calorimetry | Repeated measures ANOVAs; One sample t-tests; Bland-Altman analyses | The 2006 Crouter 2-regression model over-predicts EE at the beginning and end of walking bouts, due to high variability in accelerometer counts during the transitional minutes. | N/N | Low |
| 265 | Kumahara et al. (2004, Switzerland) | Adults (N=79; 39.7±12.4 yrs; 65% females; Japanese (N=79) | Activities of daily life (24 hours) and treadmill (36 minutes) | *Lifecorder* (waist; NR; 4 sec; proprietary algorithm) | Intensity (Energy expenditure) | Indirect room Calorimetry | Linear regression; Bland-Altman analyses; Standard error; Pearson correlation; Paired t tests; One-way ANOVA | Although TEE and PAEE were systematically underestimated during the 24 h period, the accelerometer assessed energy expenditure well during both the exercise period and the non-structured activities. | N/NR | High |
| 266 | Kumahara et al. (2009, Switzerland) | Adults (N=71; 37.9±12.4 yrs; 61% females; Japanese (N=54), Caucasian (N=17)) | Activities of daily life and treadmill (24 hrs) | *ADXL05* (waist; NR; NR; NR); *Lifecorder* (waist; NR; NR; NR) | Intensity (Physical activity energy expenditure) | Indirect room Calorimetry | Linear regression; Pearson correlation | Recording the number of steps per day does not provide accurate information on EE, and at best is only a crude predictor of the general PA in terms of displacement. In contrast, accelerometry signals are considered to be a more meaningful factor in the assessment of EE rather than step counts under sedentary conditions. | N/N | High |
| 267 | Kumahara et al. (2015, Japan) | Adults (N=9; 21.6±0.5 yrs; 100% females; NR) | Treadmill (30 minutes) | *Kenz e-style2, Tanita Calorism Smart, Omron Calori Scan HJA-306* (all chest shirt pocket, Pants pocket, shoulder bag; NR; NR; equation reported) | Intensity (Steps; Energy expenditure) | Observation (Video); MET equations | Absolute error; Two-way repeated-measures ANOVA | Monitors placed at the PP location, especially the Kenz monitor, showed acceptable accuracy for young adult women in real-life settings. In contrast, MVPA indices assessed using these monitors showed limited validity. | N/N | High |
| 268 | Kushida et al. (2001, United States) | Sleep-disordered patients (N=100; 49±14.7 yrs; 31% females; NR) | Sleep (1 night) | *Actiwatch 4* (non-dominant wrist, Actiware-Sleep v. 2.53; 30 sec; equation reported) | Biological State (Total sleep time) | Polysomnography | Sensitivity; Specificity; Accuracy; Paired t-tests; Pearson correlation | Authors recommend the use of subjective data as an adjunct to actigraphic data in estimating total sleep time and sleep efficiency in sleep-disordered patients, especially those with disorders of excessive somnolence. | NR/NR | Some |
| 269 | Kwan et al. (2020, Hong Kong) | Adults (N=31; 66.6±3.5 yrs; 48.4% females; NR) | Treadmill (30 minutes) | *ActiGraph GT3X+* (each wrist, right hip; NR; 1 minute; NR) | Intensity (Energy expenditure (MET); Steps) | Indirect Calorimetry; Observation (Video) | Spearman correlation; Percentage error | A wrist-worn ActiGraph can accurately identify different physical activity intensity levels in older people, but lower cut-off points in older people should be adopted. To measure step rate, a hip-mounted ActiGraph is preferable than a wrist-worn one. | N/N | High |
| 270 | Kwon et al. (2010, United States) | Adults (N=12; Obese group: 42.5±13.0 yrs, Normal-weight: 42.5±11.8 yrs; 100% females; Caucasian (N=12)) | Treadmill and activities of daily life (43 minutes) | *Intelligent Device for Energy Expenditure and Activity* (two at feet, two at thigh, chest; ActView Program; NR; NR) | Posture/Activity type (Sitting, lying, standing, reclining, lean, walk, staris climbing) | Observation (Direct) | Accuracy | The IDEEA monitor is a valid instrument for measuring physical activity and sedentary behavior in extremely obese women, and therefore has potential applications in bariatric surgery both in preoperative evaluation and longterm follow-up. | N/NR | High |
| 271 | Kwon et al. (2021, United States) | Adults (N=20; 32.5±15.1 yrs; 60% females; Tongan Americans (N=10), Samoan Americans (N=10)) | Activities of daily life (60 minutes) | *MotionSense HRV* (wrist; ActiLife 6; 1 minute; NR) | Intensity (Time spent sedentary, LPA and MVPA) | Indirect Calorimetry; Wearable (ActiGraph GT9X Link (wrist, waist) | Pearson correlation; Mean absolute percent errors; Bland–Altman analyses; Equivalence testing | The MotionSense HRV yielded comparable estimates for SED and PA when compared with the GT9X accelerometer under free-living conditions. | N/N | Some |
| 272 | Laakso et al. (2004, Iceland) | Adults with normal sleep (N=10; 28±10 yrs; 30% females; NR); Sleep-disordered patients without motor handicaps (N=13; 38±14 yrs; 61.5% females; NR); Sleep-disordered patients with different motor disabilities (N=16; 36±13 yrs; 31.3% females; NR) | Sleep (1 night) | *Actiwatch* (wrist; Actiwatch Sleep Analysis software v4.15; 1 minute; equation reported) | Biological state (Total sleep time) | Polysomnography | Spearman correlation; Differences with standard deviations | In subjects with rudimentary motor abilities, a standard actigraphy can produce a signal, which is related to the amount of sleep scored in polysomnograms. The sleep parameters obtained by the two methods are not equal, however. | N/NR | High |
| 273 | Ladlow et al. (2019, United Kingdom) | Adults with traumatic lower limb amputation (Unilateral: N=9; 32±5 yrs; 0% females; NR; Bilateral: N=10; 29±4 yrs; 0% females; NR; Controls: N=9; 31±6 yrs; 0% females; NR) | Treadmill (25 minutes) | *ActiGraph GT3X+* (hip; ActiLife; NR; equation reported), *Actiheart* (chest; Actiheart software; 30 sec; proprietary algorithm) | Intensity (Physical activitiy energy expenditure) | Indirect Calorimetry | Multiple linear regression analyses; Pearson correlation; Coefficients of determination; Bland-Altman analyses; One-way ANOVA; Mean absolute percentage error | Statistically derived algorithms (GT3X+HR) provide a more valid estimate of PAEE in individuals with traumatic lower-limb amputation, compared to a proprietary group calibration algorithm (AHR). | N/N | Some |
| 274 | Lai et al. (2020, United States) | Adults with Parkinson disease (N=31; 64.3±6.3 yrs; NR, NR) | Walking and treadmill (12 minutes) | *Garmin Vivosmart 3* (wrist; NR; NR; NR); *Fitbit One* (waist; NR; NR; NR), *Fitbit Charge 2 HR* (wrist; NR; NR; NR) | Intensity (Steps) | Observation (Manually- counted) | Intraclass correlation coefficients; Bland-Altman analyses; Accuracy | The waist-worn sensor (Fitbit One) was accurate and precise in measuring steps with overground and treadmill walking. The wrist-worn sensors were accurate and precise only during overground walking. | N/N | High |
| 275 | Lamont et al. (2018, Australia) | Adults with Parkinson’s disease (N=33; 68.8±8 yrs; 36% females; NR) | Walking (12 minutes indoor and 500 m outdoor) | *Fitbit Charge HR* (each wrist, NR; NR; NR), *Garmin vívosmart HR* (each wrist; NR; NR; NR) | Intensity (Steps, Time spent in different intensities) | Wearable (ActivPAL3 (thigh)); Indirect Calorimetry | Mean difference; Absolute percentage error; Paired t-tests; Bland-Altman analyses; Intraclass correlation coefficient; Repeated-measures ANOVA | The Garmin device was more accurate at reflecting step count across a broader range of walking cadences than the Fitbit, but neither strongly reflected intensity of activity. | N/N | High |
| 276 | Larkin et al. (2016, Ireland) | Adults with rheumatoid arthritis (N=20; 54±14 yrs; 100% females; NR) | Treadmill and activities of daily life (45 minutes) | *ActivPAL* (right thigh; activPAL Professional Research Edition, v7.2.32; 15 sec; NR)) | Intensity (Steps); Posture/Activity Type (Sitting, standing, lying down) | Observation (Video) | Linear regression analysis; Paired t-test; Pearson correlation; Intraclass correlation coefficient | The activPAL activity monitor underestimated step and transition counts and, therefore, is not valid for measuring these outcomes in people with RA. Relative to direct observation, the activPAL activity monitor is valid for measuring time spent in sedentary, standing, and walking behaviors in people with RA. | N/NR | High/High |
| 277 | Lauritzen et al. (2013, Spain) | Rollator dependent elderly with reduced mobility (N=5; 87.6±3.91 yrs; 80% females; NR); Elderly with normal mobility (N=7; 84.14±3.67 yrs; 85.6% females; NR); Healthy adults (N=6; 35.33±6.53 yrs; 0% females; NR) | Walking (20 meters) | *Fitbit Ultra* (dominant hip, dominant wrist; NR; NR; NR) | Intensity (Steps) | Observation (Video) | Kruskal-Wallis analyses; Mann-Whitney U tests; Pearson correlation | Slow walking speed and gait disorders hamper the utility of pedometers for physical activity measurement in rollator dependent elderly, with estimation errors >60%. The tested devices are better suited for use by ostensibly healthy elderly or adult populations. | N/NR | High |
| 278 | Le Masurier & Tudor-Locke (2003, United States) | Adults (N=20; 28.2±4.85 yrs; 35% females; NR) | Treadmill (25 minutes) | *CSA accelerometer* (right hip; NR; 30 sec; NR); *Yamax SW-200* (right hip; NR; 30 sec; NR) | Intensity (Steps) | Observation (Video) | Repeated-measures ANOVA; Students t-test; Intraclass correlation | The magnitude of the error (for either instrument) is not likely an important threat to the assessment of free-living ambulatory populations but may be a problem for pedometers when monitoring frail older adults with slow gaits. | N/N | High |
| 279 | Le Masurier et al. (2004, United States) | Adults (N=12; 29.1±6.45 yrs; 50% females; NR) | Treadmill (25 minutes) | *Yamax SW-200; Omron HJ-105*; *Sportline 330*; *CSA 7164 accelerometer* (all sensors at left or right hip; NR; CSA: 30 sec; NR) | Intensity (Steps) | Observation (Video) | Two-way repeated measures ANOVA; Percent error | Different brands of motion sensors detect steps differently; therefore, caution must be used when comparing step counts between studies that have employed different brands of motion sensors. | N/N | High |
| 280 | Leaf & MacRae (1995; United States) | Adults (N=20; 71.22±4.5; 75% females; NR) | Treadmill and outdoor walking (10 minutes) | *Caltrac* (non-dominant hip; NR; NR; ACSM prediction equations) | Intensity (Energy expenditure) | Indirect Calorimetry | Pearson correlation; Multiple regression analyses | The ACSM predictive equations are suitable for use in elderly individuals, and that the apparent differences in the relationships between treadmill and outdoor walking speeds on EE deserve further investigation.in elderly individuals. | N/NR | High |
| 281 | Lebleu et al. (2020, Belgium) | Adults (N=24; 23.4±1.3 yrs; 45.8% females; NR) | Walking (1570 m) | *Nokia Go* (dominant & non-dominant wrist, dominant waist; NR; NR; NR) | Intensity (Steps) | Wearables (Geonaute OnWalk 50, non-dominant waist & ActiGraph wGT3X-BT, nondominant wrist) | Bias; Intraclass correlation coefficient; Bland-Altman analyses | There are high discrepancies in step count between devices because of the different types of activities in daily life. The Nokia Go may be confidently used for step counting during pure walking tasks, at different locations. | NR/NR | High |
| 282 | Lee & Laurson (2015, United States) | Adults (N=39; 21.65±1.55 yrs; 56.4% females; NR) | Treadmill (15 minutes) | *SenseWear Pro3* (upper arm; NR; 1 minutes; NR); *Yamax Digiwalker SW-701* (right hip; NR; NR; NR) | Intensity (Steps) | Observation (Manually- counted) | Intraclass correlation coefficient; Repeated measure ANOVA; Bland-Altman analyses | The SWA underestimates steps during treadmill walking compared to the DIGI pedometer. | N/N | High |
| 283 | Lee & Tse (2019, China) | University students (N=27; 18-26 yrs; NR) | Treadmill (50 minutes) | *Actiwatch 2* (each wrist; NR; 1 minutes; NR), *ActiGraph wGT3X* (each wrist; NR; 1 minutes; NR) | Intensity (Energy expenditure) | Indirect Calorimetry | Multilevel regression; Bland-Altman analyses, Spearman correlation; Sensitivity; Specificity | Wrist-worn Actiwatch 2 and ActiGraph wGT3X-BT were strongly correlated in PA assessment. | N/N | High |
| 284 | Lee et al. (2011, United States) | Adults (N=46; 24.8±5.6 yrs; 46% females; NR) | Treadmill (16 minutes) | *ePulse Personal Fitness Assistant* (non-dominant forearm; NR; 1 minutes; NR) | Intensity (Energy expenditure) | Indirect Calorimetry | Intraclass correlation coefficient, ANOVA; Bland-Altman analyses | The ePulse Personal Fitness Assistant is a valid device for monitoring heart rate at rest and low-intensity exercise, but becomes less accurate as exercise intensity increases. | NR/NR | High |
| 285 | Lee et al. (2014, United States) | Adults (N=60; 26.4±5.7 yrs; 50% females; NR) | Activities of daily life (69 minutes) | *BodyMedia FIT* (non-dominant arm; ProConnect; NR; NR); *DirectLife monitor* (chest; NR; NR; NR); *Fitbit One* (waist; NR; NR; NR); *Fitbit Zip* (waist; NR; NR; NR); *ActiGraph GT3X+* (waist; NR; NR; Freedson algorithm); *Jawbone Up* (left wrist; NR; NR; NR), *Basis B1 Band* (right wrist; NR; NR; NR); *NikeFuel Band* (left wrist; NR; NR; NR) | Intensity (Energy expenditure) | Indirect Calorimetry | Equivalence testing; Pearson correlation; ANOVA, Bland-Altman analyses; Root mean square error | The indicators of the agreement clearly favored the BodyMedia FIT armband, but promising preliminary findings were also observed with the Fitbit Zip. | N/N | Low |
| 286 | Lee et al. (2015, United States) | Adults (N=43; 20.9±2 yrs; 48.8% females; NR) | Treadmill (15 minutes) | *ActiGraph GT3X+* (right hip; NR; NR; NR); *Polar Active* (left wrist; NR; NR; NR); *Omron HJ-720* (left hip; NR; NR; NR); *Yamax Digiwalker SW-701* (right hip; NR; NR; NR) | Intensity (Steps) | Observation (Manually- counted) | Intraclass correlation coefficient; Lin’s concordance coefficients; Bland–Altman analyses; Repeated-measures ANOVA | The Omron pedometer seems to provide the most reliable and valid estimate of steps taken, as it was the best performer under lab-based conditions. | NR/NR | High |
| 287 | Leicht & Crowther (2007; Australia) | Adults (N=52; 20.9±3.8; 46.2% females; NR) | Outdoor walking (6x 150 meters) | *Yamax SW-700 Digiwalker* (right waist; NR; NR; NR) | Intensity (Steps) | Observation (Manually- counted) | T-test; Two-way repeated-measures ANOVA | Walking on a soft surface such as dry beach sand significantly reduced walking speed and increased pedometer error for females compared with males, possibly by exacerbating hip and walking movements. | NR/NR | High |
| 288 | Leth et al. (2017, Denmark) | Adults (N=22; 31.1±8.03 yrs; 50% females; NR) | Outdoor walking (4 minutes) | *Garmin Vivofit 2* (wrist, NR: NR; NR); *Fitbit Charge HR* (wrist, NR; NR; NR); *Fitbit One* (hip, NR; NR; NR); *Fitbit Zip* (hip, NR; NR; NR) | Intensity (Steps) | Wearable (Shimmer 3, dominant ankle) | Percent error; Bland-Altman analyses | The measured results revealed the current functionality and limitations of the five self-tracking devices, and point towards a need for future research in this area. | N/N | High |
| 289 | Leung et al. (2021, United States) | Adults with Down Syndrome (N=18; 32.56±14.16 yrs; 52.6% females; NR) Adults without Down syndrome (N=19; 31.90±12.8 yrs; 57.9% females; NR) | Indoor walking (45 minutes) | *ActiGraph GT3X+* (right hip, ActiLife v5.4; NR; NR) | Intensity (Counts) | Indirect Calorimetry | Repeated-measure ANOVA; Correlation coefficients; Meng’s z‐test | The findings suggest that when using accelerometers to measure physical activity levels for individuals with DS, triaxial outputs may better predict physical activity levels. | N/N | High |
| 290 | Levine et al. (2000, Netherlands) | Study 1: Adults (N=11; 33±5 yrs; 54.5% females; NR); Study 2: Adults (N=8; 33±9 yrs; 50% females; NR); Study 3: Adults (N=7; 33±11 yrs; 85.7% females; NR); Study 4: Adults (N=8; 33±9 yrs; 50% females, NR) | Standing (20 minutes); Treadmill and walking (132 minutes) | *Tracmor* (lower back; NR; NR; NR) | Intensity (Energy expenditure; Tracmor units) | Indirect Calorimetry | Repeated-measures ANOVA; Two-sided paired t-tests | The Tracmor triaxial accelerometer provides reproducible and reliable data on the body motion associated with walking regardless of whether a subject walks on a treadmill or level ground. Tracmor units can be used to predict the energetic cost of walking provided that separate regression equations are derived for each subject to convert Tracmor output to energy expenditure. | N/NR | Some |
| 291 | Levine et al. (2003, United States) | Adults (N=11; 34±5 yrs; 54.5% females; NR) | Walking and treadmill (40) | *Tracmor* (lower back; NR; NR; NR) | Intensity (Energy expenditure) | Indirect Room Calorimetry | Intraclass correlation coefficient; Linear correlation analysis | When combined with laboratory measures of EE, the Tracmor accelerometer provides useful data on walking-EE and is applicable to free-living individuals. | N/NR | High |
| 292 | Leving et al. (2018, Netherlands) | Wheelchair-dependent adults (N=16; NR; 50% females; NR) | Activities of daily life (16 minutes) | *Activ8* (right forearm, right wheel; NR; 5 sec, Matlab algorithms) | Activity type (Wheelchair propulsion and other non-propulsive wheelchair-related activities) | Observation (Video) | Relative time difference; Overall agreement; Sensitivity; Positive predictive value | Activ8 system proved to be suitable for distinguishing between active wheelchair propulsion and other non-propulsive wheelchair-related activities. | NR/N | Some |
| 293 | Liang & Getchell (2018, United States) | Adults (N=20, 23.8±5.14 yrs, NR, NR) | Activities of daily life (20 minutes) | *Fitbit Zip* (waist; Fitbit Connect software; NR; NR); *Actical* (waist; NR; 15 sec; NR) | Intensity (Steps) | Observation (Video) | Two-way repeated measures ANOVA | The FitBit Zip appears to be satisfactory in its ability to calculate step count in children and in adults, except during light activity. Consumers should not rely on the FitBit Zip when tracking activity intensity levels. | N/N | Some |
| 294 | Lichstein et al. (2006, United States) | Adults (N=57; 21-87 yrs; 55.4% females; African-American (N=9), Caucasian (N=48)) | Sleep (1 night) | *Actiwatch 64* (wrist; Actiware Sleep v3.3; 30 sec; scoring algorithm) | Biological State (Total sleep time) | Polysomnography | One-way repeated-measure ANOVA; Pearson correlation | Actigraphy proved to be a satisfactory objective measure of sleep on 4 of 5 sleep parameters, but these results are specific to this particular instrument using this particular algorithm and should not be construed as a blanket endorsement of actigraphy for measuring insomnia. | Y/Y | Some |
| 295 | Liu et al. (2015, Canada) | Adults (N=40; 59±4.4 yrs; 64% females; NR) | Indoor walking (6 minutes) | *Lifesource XL-18* (front right and left pant pockets; NR; NR; NR) | Intensity (Steps) | Observation (Manually-counted) | Percent error; T-tests; Bland-Altman analyses | XL-18 is suitable for measuring steps in controlled conditions. However, caution may be required when interpreting the steps recorded under slower speeds and free-living conditions. | NR/NR | High |
| 296 | Lopes et al. (2009, Portugal) | Middle-aged to old obese/overweight and Diabetes Mellitus Type 2 adult patients (N=26; 62.6±6.5 yrs; 57.7% females; NR) | Treadmill (NR) | *ActiGraph* (right thigh; NR; 1 minutes; NR) | Intensity (Counts) | Indirect Calorimetry | Intraclass correlation; Concordance correlation coefficient; Sensitivity; Specificity | The ActiGraph is a valid and useful device for the assessment of the amount of time spent in each PA intensity levels in obese/overweight and DM2 middle-aged to old adult patients. | NR/NR | High |
| 297 | Lopez et al. (2018, Denmark) | Adults (N=36; 37.7±9.8 yrs; 44.4% females; NR) | Activities of daily life (40 minutes) | *SenseWear Mini* (non-dominant arm; software v7.0 & software v.8.1; 1 minutes; proprietary algorithms) | Intensity (Energy expenditure) | Indirect Calorimetry; Wearable (ActiGraph GT3X+ (right hip)) | Mean percent error; Bland-Altman analyses; Pearson correlation | SW and AG underestimated energy expenditure. However, both methods demonstrated a proportional bias, with increasing underestimation for increasing energy expenditure level, in addition to the large individual error. | N/N | High |
| 298 | Loprinzi & Edwards (2018, United States) | Adults (N=10; 27.2 yrs; 40% females; 80% Non-Hispanic White, 10% Non-Hispanic Black, 10% Others) | Activities of daily life (27 minutes) | *ActiGraph GT9X* (right hip; ActiLife; 1 minute; NR) | Posture/Activity Type (Sitting, standing, walking) | Observation (Direct) | Percentage | The ActiGraph GT9X accelerometer was inaccurate in detecting a sedentary break when transitioning from a sitting to standing position but was accurate for other transitional shifts. | NR/NR | High |
| 299 | Lötjönen et al. (2003, Finland) | Adults (N=32; 62±19 yrs; 75% females; NR) | Sleep (1 night) | *Vivago WristCare* (non-dominant wrist; NR; 1 minutes; scoring algorithms) | Biological State (Sleep/wake patterns) | Polysomnography; Wearable (Actiwatch (non-dominant wrist)) | Correlation coefficient; Absolute difference; Nonparametric Wilcoxon Signed Ranks Test | The performance of the WristCare can be assumed to be well comparable to actigraphy in sleep/wake studies. The study suggests that the device may be used in long-term monitoring of sleep/wake patterns with similar performance to actigraphy. | N/NR | High |
| 300 | Louter et al. (2014, Netherlands) | Parkinson disease patients (N=45; RBD+: 64.3±9.4 yrs, RBD- 58.1±8.8 yrs; 33.3% females; NR) | Sleep (1 night) | *Actiwatch 4* (wrist; Sleep Analysis 7.23; 15 sec; NR) | Biologial State (Total sleep time) | Video-Polysomnography | Pearson correlation; Multiple regression; Sensitivity; Specificity; Positive predictive value | Parkinson disease patients with sleep behavior disorder showed a significantly higher number of bouts scored as “wake” using actigraphy, compared to patients without RBD. In clinical practice, actigraphy has a high specificity, but low sensitivity in the diagnosis of sleep behavior disorder. | N/N | High |
| 301 | Lowe et al. (2010, United States) | Adults (N=32; 20.3±1.9 yrs; 100% females; NR) | Dance (20 minutes) | *Polar F6* (each wrist; NR; 1 minute; NR) | Intensity (Energy expenditure) | Indirect Calorimetry | One-way repeated measures ANOVA | Even when using actual measures of VO_2max_ and HR_max_, the Polar F6 is inaccurate in estimating EE during aerobic dance bench stepping for college-age females | NR/NR | High |
| 302 | Lützner et al. (2014, Germany) | Adults (Study 1: N=43; 24.3±5.2 yrs; 42% females; NR; Study 2: N=18; 23.6±4.4 yrs; 33% females; NR) | Study 1: Treadmill (30 minutes); Study 2: Treadmill (104 minutes) and stair climbing: 500 steps of a staircase up and down | *ActivPAL* (2x thigh, shank, 2x ankle; activPAL Professional Research Edition; NR; NR) | Intensity (Steps) | Study 1: Observation (Video); Study 2: Observation (Manually- counted) | Relative error; Bland-Altman analyses; Linear mixed effect model | For measuring step number during slow velocities, the alternative position should be favored. Stair climbing was not recorded accurately by any tested placement. | N/N | High |
| 303 | Lynn et al. (2020, United States) | Adults (N=16, 21.4±1.1 yrs, 50% females, NR) | Activities of daily life (18 minutes) | *ActiGraph GT9X Link* (wrist; ActiLife 6; NR; NR*); Fitbit Charge HR 2* (wrist; NR; NR; NR); *Apple Watch 4* (wrist; NR; NR; NR) | Intensity (Steps) | Observation (Video) | Percentage error; One-way ANOVA; Pairwise test; Tukey correction; One sample t-tests | Though wrist-worn, consumer-grade step-counting devices typically undercount steps in general, consumers should be aware that their devices may particularly undercount steps during activities with the hands fixed. This may be especially true with items in contact with the floor. | NR/NR | High |
| 304 | Maddocks et al. (2010, United Kingdom) | Adults (N=40; 28±8 yrs; 62.5% females; NR) | Treadmill (25 minutes) or motor vehicle journey (15 minutes) | *ActivPAL* (thigh; NR; NR; NR); *PALlite* (ankle; NR; NR; NR); *Yamax Digi-Walker SW-401* (waist; NR; NR; NR) | Intensity (Steps) | Observation (Video or motor vehical) | One-way ANOVA; Student t-test | The ActivPAL accelerometer accurately measures step count over a range of walking speeds and, unlike the other accelerometers tested, is not falsely triggered by motor vehicle travel. | NR/N | High |
| 305 | Madigan (2019, United States) | Adults (N=30; 80.6±7.66 yrs; 60% females; White (N=30)) | Indoor walking (74 meters) | *Garmin Vivofit 2* (wrist; NR; NR; proprietary algorithm); *Fitbit Flex* (wrist; NR; NR; proprietary algorithm); *Jawbone Up3* (wrist; NR; NR; proprietary algorithm); *Microsoft Band* (wrist; NR; NR; proprietary algorithm) | Intensity (Steps) | Observation (Video) | ±20 percent accuracy; Bland-Altman analyses | There was no relationship between the step speed and accuracy of the fitness bands. Participants using walkers and walking sticks had none of the bands that met the ±20 percent accuracy. | N/N | High |
| 306 | Maganja et al. (2020, Canada) | Adults (N=36; 71.4±4.7 yrs; 53% females; White 69%) | Indoor walking (15 minutes) | *Fitbit Charge* (wrist; NR; NR; NR); *Fitbit One* (hip; NR; NR; NR); *Garmin vívofit 2* (wrist; NR; NR; NR); *Jawbone UP2* (wrist; NR; NR; NR); *Misfit Shine* (hip; NR; NR; NR); *New-Lifestyle NL-1000* (hip; NR; NR; NR) | Intensity (Steps) | Observation (Manually counted) | Three-way ANOVA; Bland-Altman analyses, Equivalence testing; One-sided t tests | Test-retest reliability and criterion validity of step counting varied across 6 consumer-grade activity monitors worn by older adults with self-reported intact and limited mobility. The hip-worn Fitbit One was the only monitor with high test-retest reliability and criterion validity. | N/N | High |
| 307 | Magistro et al. (2018, Italy) | Adults (N=20; 75±7 yrs; 50% females; NR); Young adults (N=20; 25±5 yrs; 50% females; NR) | Walking (93 meters) and stairs climbing (35 stairs) | *ADAMO Care Watch* (each wrist; NR; NR; step detection algorithm) | Intensity (Steps) | Observation (Manually-counted) | Absolute percentage error; Intraclass correlation coefficient; Bland-Altman analyses; Repeated-measure ANOVA | Findings provide evidence that the ADAMO Care Watch demonstrated highly accurate measurements of the steps count in all activities, particularly walking at normal and slow speeds. | N/N | High |
| 308 | Maglione et al. (2013, United States) | Patients with mild to moderate Parkinson’s Disease (N=61; 67.74±8.88 yrs; 36.7% females; 96.7% Caucasian) | Sleep (1 night) | *Actiwatch-L* (non-dominant wrist; Actiware 5.0 software; 30 sec; computerized scoring algorithms) | Biological state (Total sleep time) | Polysomnography | Spearman correlation; Paired t-tests, Bland-Altman analyses | Results suggest that actigraphy may be useful for measurement of mean TST, SE, and WASO values in groups of patients with mild to moderate Parkinson’s disease. However, there is a significant degree of variability in accuracy among individual patients. | N/Y | High |
| 309 | Mahadevan et al. (2021, United States) | Adult patients with atopic dermatitis (N=33; 31.1±15.8 yrs; 69.7% females; Asian (N=1), African American (N=17), White (N=15)) | Sleep (2 nights) | *GENEActiv* (each wrist; NR; NR; sleep and scratch algorithms) | Biological state (Total sleep time) | Polysomnography | Pearson correlation; Bland-Altman analyses; Accuracy; Sensitivity; Specificity; F1 score | Results support the use of wearable sensors for objective, continuous measurement of nighttime scratching and sleep during daily life | N/N | High |
| 310 | Mahendran et al. (2016, Australia) | Adults with a unilateral stroke (N=15; 63.4±8.3 yrs; 46.7% females; NR) | Indoor walking (6 minutes); treadmill (7 minutes); Outdoor walking (200 meters) | *ActivPAL* (non-paretic thigh; proprietary software; 15 sec; NR); *Sensewear Pro2* (paretic arm; NR; 1 minute; NR); *Garmin Forerunner 405CX* (wrist; Garmin connect; NR; NR) | Intensity (Steps) | Observation (Manually counted) | Intraclass correlation coefficients; ANOVA; Bland-Altman analyses; Absolute percentage error | ActivPAL and Garmin GPS appear valid, reliable and feasible tools for community ambulation measurement after stroke, except for distance. Sensewear demonstrated poor validity and reliability when worn on the paretic arm. | N/N | High |
| 311 | Mammen et al. (2012, Canada) | Adults (N=10; 23.0±1.2 yrs; 50% females; NR) | Treadmill (8 minutes) | *Fitbit* (waist, pants pocket, upper body; NR; NR; NR); *Yamax Digiwalker SW-200* (waist; NR; NR; NR) | Intensity (Steps) | Observation (Video) | One-way repeated measures ANOVA; Mean error scores, percent errors | Our quality-control testing should now enable physical activity practitioners, consumers and researchers alike to make a more informed decision on whether to purchase and utilize the FitBit | NR/NR | High |
| 312 | Mandigout et al. (2017, France) | Adults post stroke (N=24; 68.2±13.9 yrs; 38% females; NR) | Postures (16 minutes); Activities of daily life (>3 minutes); Walking (6 minutes); Stair climbing (56 steps) | *ActiGraph GT3X-BT* (ankle, hip, wrist; NR; NR; NR); *Actical* (ankle, hip, wrist; NR; NR; NR); *Sensewear* (upper-arm; NR; NR; NR); *Geonaute ONStep 400* (neck, hip; NR; NR; NR) | Intensity (Energy expenditure) | Indirect Calorimetry | Kruskal-Wallis test, Wilcoxon signed-rank test; Spearman correlation; Intraclass correlation coefficients; Bland-Altman analyses | The present results suggest that, for a series of everyday tasks, the wearable sensors underestimate the actual energy expenditure values in post-stroke patients in the subacute phase and are therefore not accurate. Several factors are likely to confound the results: types of activity, prediction equations, the position of the sensor and the hemiplegia side. | N/N | High |
| 313 | Manns & Haennel (2012, Canada) | Adults with chronic stroke (N=12; 64.2±10.4 yrs; 42% females; NR) | Indoor walking (12 minutes) | *SenseWear Pro* (arm; software version 6.1; 1 minute; complex pattern recognition algorithms) | Intensity (Energy expenditure; Steps) | Indirect Calorimetry; Wearable (StepWatch Activity Monitor (nonparetic leg)) | Paired t-tests; Intraclass correlation coefficients; Absolute percent differences; Agreement | Results show that, for these moderately impaired persons with stroke, the SWA should be used with caution for the measurement of energy expenditure and should not be used to measure step count. | NR/NR | High |
| 314 | Marino et al. (2013, United States) | Adults (N=77; 35.0±12.5 yrs; 39% females; 74% White, 18.2% Black, 7.8% Other) | Sleep (mean nights 3.2) | *Actiwatch-64* (wrist; Actiware software v3.4/v5.57/ 5.59; 30 sec; Cole-Kripke algorithm); *Actiwatch Spectrum* (wrist; Respironics Actiware 5 v5.57/5.59; 30 sec; Cole-Kripke algorithm) | Biological State (Sleep/wake classification) | Polysomnography | Accuracy; Sensitivity; Specificity; Generalized estimating equation analysis; Spearman correlation; Linear regression; Multivariable linear regression analysis; Bland-Altman analyses | We conclude that actigraphy is overall a useful and valid means for estimating total sleep time and wakefulness after sleep onset in field and workplace studies, with some limitations in specificity. | N/N | Some |
| 315 | Marsh et al. (2007, United States) | Adults at risk for mobility disability (N=29; 75.8±4.2 yrs; 69% females; NR) | Indoor walking (131 meters) | *Accusplit Eagle 120* (right hip; NR; NR; NR); *New Lifestyles-2000* (right hip; NR; NR; NR), *IDEEA* (each foot, each thigh, chest; IDEEA ActView software; NR; NR) | Intensity (Steps) | Observation (Manually counted) | Pearson product-moment correlations; Repeated-measures ANOVA; Paired t-tests; Spearman correlations; Bland-Altman analyses | Our data show that the AE120 is a poor choice for measuring physical activity in older adults at risk for mobility disability. Both the NL-2000 and IDEEA devices have acceptable measurement qualities; however, the NL-2000 is the more practical of the two for use in either research or clinical practice. | N/NR | High |
| 316 | Martien et al. (2015, Belgium) | Adults in nursing homes (N=60; 85.5±5.5 yrs; 78.3% females; NR) | Postures (18 minutes); Walking (4 minutes); Activities of daily life (8 minutes) | *SenseWear Mini* (left upper arm; Innerview Research Software version 7.0; 1 min; proprietary algorithms) | Intensity (Energy expenditure) | Indirect Calorimetry | Unpaired t-test; Percent error; Paired t-tests; Pearson product-moment correlations; Intrclass correlation coefficient; Bland-Altman analyses; Regression analyses | The high percent error scores indicate that the SWMini is of limited value for quantifying EE in the old and old-old. The accuracy could be improved by developing accurate age- and activity-specific algorithms. On the other hand, the SWMini can be used as a suitable device for researchers interested in specific levels and patterns of PA and sedentary behavior. | N/N | Some |
| 317 | Martien et al. (2015, Belgium) | Adults in nursing homes (N=68; 85.8±5.6 yrs; 79.4% females; NR) | Walking (4 minutes); Activities of daily life (26 minutes) | *New Lifestyles 2000* (waist, ankle; NR; NR; NR); *SenseWear Mini* (left upper arm, NR; 1 minute; NR) | Intensity (Steps) | Observation (Manually counted) | Pearson product-moment correlations; Intraclass correlations; Percent error; Bland-Altman analyses; Regression analyses; Receiver operator characteristic curves | The ankle-worn piezoelectric pedometer can be useful for accurate quantification of walking steps in the old and old-old walking faster than 2.35 km/h. | NR/NR | High |
| 318 | Martin et al. (2012, Canada) | Adults (N=18, 63.6 yrs, 67% females, NR) | Indoor walking (640 meters) | *Omron HJ 105* (waist; NR; NR; NR); *Yamax Digiwalker 200* (waist; NR; NR; NR); *SportLine 330* (waist; NR; NR; NR); *New-Lifestyles 2000* (waist; NR; NR; NR); *Actical* (waist; NR; NR; NR) | Intensity (Steps) | Observation (Manually counted) | Absolute percent error; Mean percent errors; Repeated measures ANOVA; Intraclass correlation coefficients | Further research is required to develop pedometer mechanisms that accurately measure steps at slower walking speeds | N/NR | High |
| 319 | Martin et al. (2015, United Kingdom) | Adults (N=15; 18-56 yrs; 100% females; NR) | Postures (30 minutes); Walking (3 minutes); Sitting and rocking (3 minutes); Slow dancing (3 minutes) | *ActivPAL* (left thigh, left shank; NR; NR; NR) | Posture/Activity Type (Lying, sitting, standing, stepping, all fours, other) | Observation (Video) | Confusion matrix; Agreement; Sensitivity; Specificity | This validated system can be used to measure elected activity of laboring women and report on effects of postures on length of first stage, pain experience, birth satisfaction, and neonatal condition | NR/Y | High |
| 320 | Martinato et al. (2021, Italy) | Adults (N=49, 74 yrs, 47% females, NR) | Walking (150 meters) | *Garmin vívoactive HR* (left wrist, NR; NR; NR) | Intensity (Steps) | Observation (Manually counted) | Wilcoxon Kruskal-Wallis test; Pearson chi-square test; Bland-Altman analyses; Intraclass Correlations; Generalized linear model | The level of accuracy of wearable devices in quantifying the PA of elderly people in a real-life setting that was found in this study supports the idea of considering wrist-wearable nonmedical devices (widely available in nonspecialized stores) as reliable tools. Both health care professionals and informal caregivers could monitor the level of PA of their patients | N/N | High |
| 321 | Maskevich et al. (2017, Australia) | Adults with Huntington’s Disease Gene (N=7, 54.14±6.4 yrs; 86% females, 100% Caucasian) | Sleep (1 night) | *Jawbone UP2* (non-dominant wrist; NR; 1 minute; NR), *Fitbit One* (non-dominant wrist; NR; 1 minute; NR); *Actiwatch Spectrum Pro* (non-dominant wrist; NR; 1 minute; NR) | Biological State (Total sleep time) | Polysomnography | Paired t-tests; Bland-Altman analyses; Sensitivity; Specificity; Accuracy; Predicted value; Prevalence and Bias-Adjusted Kappa. | None of the activity monitors are sufficiently accurate to replace polysomnography, although they may be sufficient for estimating overall sleep-wake patterns. | N/N | High |
| 322 | Matthews et al. (2000, United States) | Adults (N=19; 42±11 yrs; 63% females; NR) | Treadmill (NR); Activities of daily life (NR) | *Actillume monitor* (waist; ACTION 3 software; NR; NR) | Intensity (Energy expenditure) | Indirect Calorimetry | Regression; ANOVA | In laboratory testing, the Actillume monitor discriminated between sedentary and moderate intensity activities and was highly correlated with oxygen consumption. | N/NR | High |
| 323 | McClain et al. (2010, United States) | Adults (N=26; 27.3±7.1 yrs, 65.4% females; NR) | Treadmill (10 minutes) | *Omron HJ-151* (waist; NR; 1 minute; NR); *New Lifestyles NL-1000* (waist; NR; 4 sec; NR); *Walk4Life Pro* (waist; NR; 1 sec; NR); *Yamax SW-200* (waist; NR; NR; NR); *ActiGraph GT1M* (waist; NR; 1 minute; Freedson, Matthews, Swartz cut-points) | Intensity (Steps; Time spent in MVPA) | Observation (Manually-counted); Wearable (ActiGraph GT1M (waist)) | Percent error; Absolute percent error; Repeated-measures ANOVA; Agreement | Current findings indicate that the OM, NL, and W4L can provide reasonable estimates of free-living MVPA or activity time in comparison with a range of AG walking and lifestyle cutpoints | NR/NR | High |
| 324 | McCullagh et al. (2017, Ireland) | Adults (N=32; 78.1±7.8 yrs; 47% females; NR) | Walking (60 meters); Activities of daily life (<40 minutes) | *StepWatch Activity Monitor* (dominant ankle; NR; 15 sec; NR); *ActivPAL3* (dominant thigh; NR; 15 sec; NR), *Piezo Step MV* (dominant hip; NR; NR; NR) | Intensity (Steps) | Observation (Video) | Percentage error, Intraclass correlation coefficient; Bland-Altman analyses | The ankle-worn accelerometer gave the most accurate step-count measurement and was most accurate over longer distances. Neither of the other motion sensors had acceptable margins of error. | N/NR | Some |
| 325 | McDevitt et al. (2021, Ireland) | Adults (N=12; Males: 23±3.4 yrs, females: 22±0 yrs; 8% females; NR) | Treadmill (10 minutes); Postures (2 minutes); Indoor walking (6 minutes + 800 meters); Stairs (30 steps) | *Verisense IMU* (non-dominant wrist; Shimmers remote cloud system; 15 sec; algorithm reported) | Intensity (Time spent in sedentary, light, and MVPA) | Wearable (Actiwatch 2 (non-dominant wrist) | Spearman’s rank-order correlation coefficient; Sensitivity; Specificity; Accuracy; Bland-Altman analyses | The results showed moderate-high agreement of Verisense with Actiwatch 2 for assessing epoch-by-epoch physical activity and sleep, but a lack of agreement for activity classifications. | N/N | High |
| 326 | Melanson & Freedson (1995, United States) | Adults (N=28; Males: 21±1 yrs, females: 21±1.1 yrs; 46.4% females; NR) | Treadmill (8 minutes) | *CSA accelerometer* (ankle, hip, wrist; NR; 5 sec; NR); *Caltrac* (hip; NR; 2 minutes; internal algorithm) | Intensity (Energy expenditure) | Indirect Calorimetry | Pearson product-moment correlation; ANOVAs; F-test; paired t-tests | CSA and Caltrac accelerometers have similar validity and either instrument can be used to estimate EE of groups. | NR/NR | High |
| 327 | Melanson et al. (2014, United States) | Adults ((Study 1: N=259; 63 yrs; 58.3% females; NR); (Study 2: N=32; 19-51 yrs; 50% females; NR)) | Treadmill (56 minutes); Indoor walking (300 feet) | *Yamax SW-200* (left hip, NR); *Omron HF-100* (right hip; NR; NR; NR); *Walk-4-Life LS-2500* (foot; NR; NR; NR); *Step Keeper HSB-SKM* (foot; NR; NR; NR) | Intensity (Steps) | Observation (Manually counted) | Paired t-tests; Repeated-measures ANOVA | Accuracy of all pedometers tested exceeded 96% at speeds 3.0 MPH, but decreased at slower walking speeds. | N/NR | High |
| 328 | Miller et al. (2020, Australia) | Adults (N=12; 22.9±3.4 yrs; 50% females; NR) | Sleep (10 nights) | *WHOOP strap* (non-dominant wrist; NR; 30 sec; proprietary algorithms (generation 3)) | Biological state (Total sleep time) | Polysomnography | Linear mixed models; Sensitivity; Specifity; Agreement; Cohen’s kappa; Bland-Altman analyses | In situations where polysomnography is impractical (e.g., field settings), WHOOP is a reasonable method for estimating sleep, particularly for 2-stage categorisation, if accurate bedtimes are manually entered. | N/Y | High |
| 329 | Miller et al. (2021, Australia) | Adults (N=54; 23.0±2.2 yrs; 50% females; NR) | Sleep (9 nights) | *Actical* *Z-series* (non-dominant wrist, Actiware v.3.4; 30 sec; NR), *WHOOP strap* (non-dominant wrist, NR; 30 sec; NR) | Biological state (Total sleep time) | Polysomnography | General Linear mixed models; Bland–Altman analyses; Sensitivity; Specifity; Agreement; Cohen’s kappa; Intraclass correlation coefficients | WHOOPAUTO and WHOOP-MANUAL have a similar sensitivity and specificity to actigraphy for two-stage categorisation of sleep and can be used as a practical alternative to polysomnography for two-stage categorisation of sleep and four-stage categorisation of sleep. | N/Y | High |
| 330 | Modave et al. (2017, United States) | Adults (N=60; 49.5±19.4 yrs; 60% females; NR) | Treadmill (2000 steps) | *Fitbit Surge* (each wrist; NR; NR; NR); *Garmin Vivofit* (each wrist; NR; NR; NR); *Apple watch* (each wrist; NR; NR; NR), *Samsung Gear S2* (each wrist; NR; NR; NR), *Garmin 735XT* (each wrist; NR; NR; NR); *ActiGraph wGT3X-BT* (waist; NR; NR; NR) | Intensity (Steps) | Observation (Manually counted) | Repeated measures mixed-effects model | Analysis shows that apart from the Fitbit Surge, most of the recent mobile devices we tested do not overcount or undercount steps in the 18-39-year-old age group, however some devices undercount steps in older age groups. | N/N | High |
| 331 | Montes et al. (2017, United States) | Adults (N=49; 23.4±6.6 yrs; 47% females; NR) | Treadmill (18 minutes) | *Fitbit Flex* (right wrist; NR; NR; NR) | Intensity (Energy expenditure; Steps) | Indirect Calorimetry; Observation (Manually counted) | Dependent t-test analysis | Based on the results of this study, the FWAT does not accurately measure nor is it consistent in returning energy expenditure or step count measures. | NR/NR | High |
| 332 | Montes et al. (2019, United States) | Adults (N=40; 25.09±7.17 yrs; 50% females; NR) | Indoor walking (20 minutes); Treadmill (20 minutes) | *Samsung Gear 2* (wrist; NR; NR; proprietary algorithm); *Fitbit Surge* (wrist; NR; NR; proprietary algorithm); *Polar A360* (wrist; NR; NR; proprietary algorithm); *Garmin Vivosmart HR+* (wrist; NR; NR; proprietary algorithm); *Leaf Health Tracker* (waist; NR; NR; proprietary algorithm) | Intensity (Steps) | Observation (Manually counted) | 2x6 repeated measures ANOVA | There may be some conditions such as step count measurements taken while jogging/running that may only require treadmill-based validity testing. | N/N | High |
| 333 | Montes et al. (2020, United States) | Adults (N=40; 25±7 yrs; 50% females; NR) | Walking (20 minutes); Treadmill (20 minutes) | *Samsung Gear 2* (wrist; NR; NR; proprietary algorithm); *Fitbit Surge* (wrist; NR; NR; proprietary algorithm); *Polar A360* (wrist; NR; NR; proprietary algorithm); *Garmin Vivosmart HR+* (wrist; NR; NR; proprietary algorithm); *Leaf Health Tracker* (waist; NR; NR; proprietary algorithm) | Intensity (Steps) | Observation (Manually counted) | Pearson correlation; Mean absolute percent error; Bland-Altman analyses | While each device returned some level of consistency and accuracy during either free motion or treadmill exercises, the Garmin Vivosmart HR+ and the Leaf Health Tracker were deemed to be reliable and valid for all conditions tested. | NR/NR | High |
| 334 | Montgomery et al. (2009, Australia) | Adults (N=17; males: 29.8±4.3 yrs, females: 25.6±3.6 yrs; 41% females; NR) | Treadmill (20 minutes) | *Suunto HR monitoring system* (NR; Suunto software; 30 sec; NR) | Intensity (Energy expenditure) | Indirect Calorimetry | Standard error of the estimate; Coefficient of variation; Linear regression; Pearson correlation | Although reliable, basic HR-based estimations of VO2 and energy expenditure from the Suunto system underestimated VO2 and energy expenditure by 6 and 13%, respectively. However, estimation can be improved when maximal HR and VO2 values are added to the software analysis. | N/NR | High |
| 335 | Montgomery-Downs et al. (2012, United States) | Adults (N=24; 26.1 yrs; 40% females; 92% White) | Sleep (1 night) | *Fitbit* (non-dominant wrist; NR; 1 minute; NR) | Biological State (Total sleep time) | Polysomnography; Wearable (Actiwatch-64 (non-dominant wrist)) | Specificity; Sensitivity; Cohen’s d; Repeated-measures ANOVA; Bland-Altman concordance technique; Bland-Altman analyses | Fitbit has the same specificity limitations as actigraphy; both devices consistently misidentify wake as sleep and thus overestimate both sleep time and quality. | N/N | High |
| 336 | Montoye et al. (2016, United States) | Adults (N=39; 22.1±4.3 yrs; 51.3% females; NR) | Indoor walking (3-10 minutes); Stairs (3-10 minutes); Activities of daily life (3-10 minutes); Exercise (3-10 minutes); Cycling (3-10 minutes) | *GENEActiv* (each wrist; NR; NR; artificial neural networks) | Intensity (Energy expenditure) | Indirect Calorimetry | Correlation coefficients; Root mean square error; Repeated-measure ANOVA; Paired t-tests | Highly accurate, wrist-independent EE prediction ANNs were developed by computing absolute values of raw acceleration data prior to ANN development. | N/N | Low |
| 337 | Montoye et al. (2016, United States) | Adults (N=40; 22±4.2 yrs; 52.5% females; NR) | Postures (3-10 minutes); Activities of daily life (15-50 minutes); Exercise (6-20 minutes); Walking (9-30 minutes); Cycling (3-10 minutes); stairs (3-10 minutes) | *ActiGraph GT3X+* (thigh, hip; NR; NR; artificial neural networks); *GENEActiv* (each wrist; NR; NR; artificial neural networks) | Intensity (Time spent in sedentary; LPA; MVPA; Sedentary breaks) | Observation (Direct) | Sensitivity; Specificity; Repeated measures ANOVA; Confusion matrices; Kappa statistics | Coupled with machine learning modeling, the thigh-worn accelerometer should be considered when objectively assessing PA and SB. | N/N | High |
| 338 | Montoye et al. (2017, United States) | Adults (N=41; 22.0±4.2 yrs; 51% females; NR) | Postures (3-10 minutes); Activities of daily life (15-50 minutes); Exercise (6-20 minutes); Walking (9-30 minutes); Cycling (3-10 minutes); Stairs (3-10 minutes) | *ActivPAL 3* (right thigh; activPAL Research Edition 6.4.1 software; 15 sec; proprietary algorithm, artificial neural network) | Intensity (Energy expenditure; Time spent in sedentary; light; MVPA) | Indirect Calorimetry | Repeated measures ANOVA; Correlations; Bias; Root mean square error; paired t-test; Bland-Altman analyses | The ANN had higher accuracy for estimation of EE and PA than the activPAL software in this semi-structured laboratory setting, indicating potential for the ANN to be used in PA assessment. | N/NR | Some |
| 339 | Montoye et al. (2017, United States) | Adults (N=32; 23.5±1.3 yrs; 43.8% females; NR) | Postures (15 minutes); Treadmill (35 minutes); Cycling (5 minutes); Indoor walking (600 meters) | *Fitbit Charge HR* (non-dominant wrist; NR; NR; proprietary algorithms) | Intensity (Steps; Energy expenditure) | Indirect Calorimetry; Wearable (Omron HJ 323u; hip)) | Repeated-measures ANOVA; Mean absolute percent error; Paired t-tests; Bland-Altman analyses | The Fitbit has utility for measurement of some, but not all, physical activity and physiologic variables which it measures | N/N | High |
| 340 | Montoye et al. (2019, United States) | Adults that are pregnant (N=40; 28.6± 3.8 yrs; 100% females; NR) | Treadmill (10 minutes) | *ActiGraph GT9X Link* (right hip; ActiLIfe v6.13.3; NR; proprietary algorithms, Freedson 1998 equation, Freedson 2011 equation); *Omron HJ-720* (left hip; NR; NR; proprietary algorithms); *New Lifestyles NL 2000* (thigh; NR; NR; proprietary algorithms); *Fitbit Flex* (non-dominant wrist; NR; NR; proprietary algorithms); *StepWatch* (right ankle; software v3.4; NR; proprietary algorithms) | Intensity (Steps; Energy expenditure) | Observation (Manually counted); Indirect Calorimetry | Mean absolute percent error; repeated-measures ANOVA; Paired-samples t-tests; Bland-Altman analyses; Two one-sided test method | The ankle-worn SW and hip-worn OM had high accuracy for measuring step counts at all treadmill walking speeds, whereas the NL had high accuracy for speeds ≥2.0 miles/hour. Conversely, the monitors tested for Calorie expenditure have poor accuracy and should be interpreted cautiously for walking behavior. | N/N | Some |
| 341 | Moreno et al. (2020, United States) | Adults and adults in wheelchairs (N=30; 35.7±14.0 yrs; 40% females; NR) | Treadmill (20 minutes); Cycle ergometry (20 minutes) | *Apple Watch Series 1* (dominant wrist; NR; NR; NR) | Intensity (Energy expenditure) | Indirect Calorimetry | ANOVAs; Pairwise T-tests; Intraclass correlation coefficients; Bland-Altman analyses | The smartwatch is currently not well suited to calculate caloric expenditure when performing exercise tasks on a wheelchair treadmill and arm cycle ergometry. | N/N | High |
| 342 | Moreno-Pino et al. (2019, Spain) | Adults with obstructive sleep apnea (N=65; 58.84±13.84 yrs; 35% females; NR) | Sleep (1 night) | *Fitbit Charge 2* (wrist; Fitbit application; NR; proprietary algorithm); *Fitbit Alta HR* (wrist; Fitbit application; NR; proprietary algorithm) | Biological state (Total sleep time) | Polysomnography | Paired t tests; Bland-Altman analyses; Sensitivity; Specificity | Fitbit wearable devices showed an acceptable sensitivity but poor specificity. Consumer sleep trackers still have insufficient accuracy for clinical settings, especially in clinical populations. | N/NR | High |
| 343 | Morris et al. (2019, United States) | Adults (N=47; 28.5±11.6 yrs; 53% females; NR) | High intensity functional training (15 minutes) | *ActiGraph GT3X* (waist; NR; NR; NR); *Nike Fuelband* (wrist; NR; NR; NR); *Fitbit One* (hip; NR; NR; NR); *Fitbit Charge HR* (wrist; NR; NR; NR); *Jawbone UP Move* (hip; NR; NR; NR) | Intensity (Energy expenditure) | Indirect Calorimetry | Mixed effects model intraclass correlation coefficient; Repeated-measures ANOVA; equivalence testing; Pearson correlation; Mean absolute percentage errors | The wrist- and hip-mounted activity trackers did not accurately assess energy expenditure during HIFT exercise. With the exception of the ActiGraph GT3X, the remaining four activity trackers showed inaccurate estimates of the amount of kilocalories expended during the HIFT exercise bout compared to the PMA. | N/NR | High |
| 344 | Motl et al. (2005, United States) | Adults with multiple sclerosis (N=23; 40.3±8.6 yrs; 91% females; NR) | Treadmill (25 minutes) | *Yamax SW-200* (non-dominant hip; NR; NR; NR); *Yamax SW-401* (non-dominant hip; NR; NR; NR) | Intensity (Steps) | Observation (Video) | Repeated-measures ANOVA | Those results support the quantification of physical activity using pedometers among those with MS who are ambulatory without an aide. | N/NR | High |
| 345 | Motl et al. (2011, United States) | Adults with and without multiple sclerosis (N=48; with MS: 43.5±12.2 yrs; Healthy: 40.9±11.4 yrs; 83% females; NR) | Treadmill (18 minutes) | *ActiGraph 7164* (right hip; NR; 30 sec; NR) | Intensity (Steps) | Observation (Manually counted) | Percentage error; Mixed-model ANOVA; Pearson product moment correlations; Spearman rho correlations | Findings support the accuracy of a waist worn ActiGraph accelerometer for the measurement of steps in persons with MS and control subjects. | N/NR | High |
| 346 | Mudge et al. (2007, New Zealand) | Adults with chronic stroke (N=25; median 69 yrs; 32% females; NR) | Indoor Walking (36 meters); Outdoor walking (216 meters) | *StepWatch 3* (ankle; NR; 3 sec; NR) | Intensity (Steps) | 3-dimensional gait analysis (3-DGA); footswitches | Bland-Altman analyses; Pearson’s correlation coefficients; Percentage error | Criterion validity of the SAM to measure steps in both clinical and natural environments has been established when used on the nonparetic limb. | N/N | High |
| 347 | Murakami et al. (2019, Japan) | Adults (N=19; 32.3±9.6 yrs; 53% females; NR) | Activities of daily life (805 minutes); Treadmill (120 minutes); Postures (60 minutes); Sleep (480 minutes) | *Fitbit Flex* (non-dominant wrist; NR; NR; NR); *Jawbone UP24* (non-dominant wrist; NR; NR; NR); *Misfit Shine* (non-dominant wrist; NR; NR; NR); *EPSON PULSENSE* (non-dominant wrist; NR; NR; NR); *Garmin Vivofit* (non-dominant wrist; NR; NR; NR); *TANITA AM-160* (pocket; NR; NR; NR); *Omron CaloriScan* (pocket; NR; NR; NR); *Withings Pulse O2* (waist; NR; NR; NR); *Omron Active style Pro* (waist; NR; NR; NR); *Panasonic Actimarker* (waist; NR; NR; NR); *SUZUKEN Lifecorder EX* (waist; NR; NR; NR); *ActiGraph GT3X* (waist; NR; NR; NR) | Intensity (Energy expenditure) | Indirect Room Calorimetry | Mean absolute percent errors; Pearson correlations; Spearman correlations; Bland-Altman analyses | Most wearable devices do not provide comparable PAEE estimates when using gold standard methods during 1 standardized day. | N/Y | High |
| 348 | Nakazaki et al. (2014, Japan) | Adults (N=34; 21.9±1.7 yrs; 26% females; NR) | Sleep (1 night) | *FS-750* (waist; NR; 2 minutes; algorithm reported) | Biological state (Total sleep time) | Polysomnography | Unpaired t-tests; Paired t-tests, Sensitivity; Specificity, Agreement; Intraclass correlation coefficient | The developed algorithm could determine sleep/wake states from activity intensity data obtained with the FS-750 with sensitivity and specificity equivalent to that determined with conventional actigraphs. | N/N | High |
| 349 | Navalta et al. (2018, United States) | Adults (N=20; 22.2±5.8 yrs; 40%, females; NR) | Outdoor hiking (10 minutes); Outdoor running (10 minutes) | *Fitbit Surge 2* (wrist; NR; NR; NR); *Garmin Vivosmart HR+* (wrist; NR; NR; NR), *Leaf Health Tracker* (waist; NR; NR; NR); *Polar A360* (wrist, NR; NR; NR); *Samsung Gear 2* (wrist; NR; NR; NR); *Spire Activity Tracker* (waist, NR; NR; NR); *Stryd Power Meter* (shoes; NR; NR; NR) | Intensity (Steps) | Observation (Manually counted) | Intraclass correlation coefficient; Bland-Altman analyses; Mean average percentage error | As only certain devices returned valid step measurements, continued testing in applied environments are needed to have confidence in utilizing technology to track health and activity goals. | NR/NR | High |
| 350 | Nazarahari & Rouhani (2018, Canada) | Adults (N=10; 27±12 yrs; 0% females; NR) | Postures and indoor walking (NR) | *Physilog* (chest; NR; NR; NR) | Posture/Activity type (Lying; sitting, standing; walking) | Observation (Video) | Accuracy; Sensitivity; Specificity; Correlation coefficients | The proposed method enabled detection and classification of postural transitions and walking modalities with high sensitivity and specificity using only one chest-mounted accelerometer. | N/N | Some |
| 351 | Nelson et al. (2016, United States) | Adults (N=30; 48.9±19.4 yrs; 50% females; NR) | Postures (20 minutes); Activities of daily life (20 minutes); Exercise (20 minutes) | *Fitbit One* (hip; Fitbit Connect Application; NR; NR); *Fitbit Zip* (hip; Fitbit Connect Application; NR; NR); *Fitbit Flex* (non-dominant wrist; Fitbit Connect Application; NR; NR); *Jawbone UP24* (non-dominant wrist; Jawbone Application; NR; NR); *Omron HJ-720IT* (hip, NR; NR; NR) | Intensity (Energy expenditure; Steps) | Indirect Calorimetry; Observation (Manually counted) | Friedman Test; Dunn’ s test; Mean absolute error; Mean absolute percentage error; Root mean square error | Consumer based PA monitors should be used cautiously for estimating EE, although they provide accurate measures of steps for structured ambulatory activity, similar to validated pedometers. | N/N | Some |
| 352 | Ng et al. (2012, Australia) | Adults with chronic obstructive pulmonary disease (N=20; 73±8.5 yrs; 60% females; NR) | Indoor walking (20 minutes) | *Stepwatch activity monitor* (right ankle; NR; 1 minute; NR); *ActivPAL* (thigh, NR; 15 sec; NR) | Intensity (Steps) | Observation (Manually counted) | Repeated measures ANOVA; Bland- Altman analyses | StepWatch activity monitor can be used to detect steps in people who walk very slowly including those who use a rollator. Both devices were sensitive to small changes | N/N | High |
| 353 | Nguyen et al. (2013, Switzerland) | Adults (N=24; 25 ±4 yrs; 50% females; NR) | Walking (34 minutes); Cycling (8-12 minutes) | *Lifecorder Kenz EX* (waist; NR; NR; algorithm reported); *Stepwatch 3 Activity Monitor* (ankle; NR; 6 sec; algorithm reported) | Intensity (Energy expenditure) | Indirect Calorimetry | Univariate linear regression; Pearson correlation; Bland-Altman analyses, 2-sample paired t test | Performed PA types could be recognized with little error. | N/NR | Some |
| 354 | Nichols et al. (1999, United States) | Adults (N=60; 23.4±2.9 yrs; 50% females; NR) | Treadmill (20 minutes) | *Tritrac R3D activity monitor* (each hip; NR; 1 minute; algorithm reported) | Intensity (Energy expenditure) | Indirect Calorimetry | Intraclass correlation coefficients; Paired t tests; ANOVA with repeated measures; Linear regression analysis | These data indicate that the Tritrac is highly reliable from day to day and is sensitive to changes in speed but not grade. Furthermore, the Tritrac accurately distinguishes various intensities of walking and jogging on level ground. With limitations, these cut-points can be used to categorize light, moderate, and vigorous physical activity and to estimate EE. | NR/NR | Some |
| 355 | Nichols et al. (2000, United States) | Adults (N=60; Males:23.0±2.9 yrs; Females: 22.9±2.9 yrs; 50% females; NR) | Treadmill (20 minutes); Outdoor walking (15 minutes) | *ActiGraph 7164* (each hip; NR; 1 minute; NR; NR) | Intensity (Energy expenditure) | Indirect Calorimetry | Intraclass correlation coefficients; T tests; Repeated-measures ANOVA; Regression analysis; Paired t-tests | We conclude that the CSA can be used to quantify walking and jogging outdoors on level ground; however; laboratory equations may not be appropriate for use in field settings, particularly for light and vigorous activity | NR/NR | High |
| 356 | Nielson et al. (2011, United States) | Adults (N=100; 23.3±3.9 yrs; 33.3% females; Caucasian (N=100)) | Treadmill (10 minutes) | *Walk4Life Elite pedometer* (waist; NR; NR; NR) | Intensity (Steps; Energy expenditure) | Observation (Manually counted); Indirect Calorimetry | Linear mixed model regression analysis | The pedometers’ inability to accurately estimate energy expenditure cannot be attributed to stride length entered into the pedometer or its ability to measure step counts. | Y/NR | High |
| 357 | Nightingale et al. (2014, United Kingdom) | Adults using a manual wheelchair (N=15; 36±11 yrs; NR, NR) | Activities of daily life (6 minutes); Outdoor wheelchair propulsion (24 minutes) | *ActiGraph GT3X+* (right wrist, upper arm, waist; NR; 1 minute; proprietary digital filtering algorithms) | Intensity (Energy expenditure) | Indirect Calorimetry | Pearson correlation; Coefficients of determination; Linear regressions; Bland–Altman analyses | The ActiGraph GT3X+ is a reliable tool for determining mechanical movements within the physiological range of human movement. Of the three anatomical locations considered, a wrist-mounted accelerometer explains more of the variance and results in the lowest random error when predicting physical activity energy expenditure in manual wheelchair users. | N/N | High |
| 358 | Nightingale et al. (2015, United Kingdom) | Adults using a manual wheelchair (N=17; 36±10 yrs; 0% females; NR) | Postures (6 minutes); Activities of daily life (6 minutes); Treadmill (48 minutes) | *ActiGraph GT3X+* (right wrist, upper arm; ActiLife software; 1 minute; proprietary digital filtering algorithms); *GENEActiv* (right wrist, upper arm; GENEActiv PC software v1.2.1; 1 minute; NR) | Intensity (Energy expenditure) | Indirect Calorimetry | Linear regression analyses; Pearson correlation; Coefficients of determination; Mean absolute error; Mean signed error; Bland-Altman analyses; Two-way mixed model ANOVA | The results indicate that the GENEActiv device worn on either the upper arm or wrist provides the most valid prediction of PAEE in MWUs. Variation in error statistics between the two devices is a result of inherent differences in internal components, on-board filtering processes and outputs of each device. | N/N | High |
| 359 | Noah et al. (2013, United states) | Adults (N=23; 26.65±7.55 yrs; 43% females; NR) | Postures (6 minutes); Treadmill (18 minutes); Stair climbing (6 minutes) | *Fitbit* (waist; NR; 1 min; proprietary algorithms); *Fitbit Ultra* (waist; NR; 1 min; proprietary algorithms), *Actical* (waist; NR; 1 min; proprietary algorithms) | Intensity (Steps; Energy expenditure) | Indirect Calorimetry | Intraclass correlation coefficients; ANOVA | Results indicate the Fitbit and Fitbit Ultra are reliable and valid for activity monitoring (step counts) and determining energy expenditure while walking and jogging without an incline. The Fitbit and standard accelerometers under-estimated energy expenditure compared to indirect Calorimetry for inclined activities. | N/N | High |
| 360 | Nuss et al. (2019, United States) | Adults (N=30; 23.5±2.96 yrs; 50% females; NR) | Postures (5 minutes); Treadmill (NR) | *Apple Watch* (right wrist; NR; NR; NR); *Fitbit Charge HR 2* (left wrist, NR; NR; NR) | Intensity (Energy expenditure) | Indirect Calorimetry | Relative error rates; Paired t-tests; Regression scatterplots; Concordance correlation coefficients | Neither device showed accurate results compared with EE measured by a MetCart. | N/N | High |
| 361 | Nuss et al. (2020, United States) | Adults (N=30; 22.93±3.30 yrs; 50% females; NR) | Postures (5 minutes); Treadmill (49 minutes) | *ActiGraph wGT3X* (each wrist, each hip; ActiLife software version 6.13.3; 10 sec; Freedson VM3 combination algorithm) | Intensity (Energy expenditure; Steps) | Indirect Calorimetry, Observation (Video) | Interclass correlation coefficient; One-sided tests; Mean absolute percent error; Cohen’s d effect sizes; Lin’s concordance correlation coefficients; Bland-Altman analyses; Linear regressions | Neither hip nor wrist placements assess EE accurately. For step counts, both dominant and nondominant hip placements, but not wrist placements, lead to accurate results for both men and women. | NR/NR | Some |
| 362 | O’Brien et al. (2021, Canada) | Adults (N=20; 25±6 yrs; 65% females; NR) | Treadmill (42 minutes) | *ActivPAL 3* (right thigh; PAL Software Suite v7; 15 sec; algorithm reported) | Intensity (Energy expenditure) | Indirect Calorimetry | Repeated-measure ANOVA; Friedman’s ANOVA; Absolute error; Equivalence testing; Bland-Altman analyses | The absolute percent error of the counts-METs model in the laboratory cross-validation was 18±13%, with equivalence testing determinining equivalent MET values to indirect Calorimetry during the slowest (1.5 mph) and fastest (4.0-4.5 mph) stages. | N/N | High |
| 363 | O’Brien et al. (2018, Canada) | Adults (N=43; 39.4±15.2 yrs; 85% females; NR) | Treadmill (36 minutes) | *PiezoRx* (left waist; NR; NR; algorithm reported); *Omron HJ-320* (right waist; NR; NR; NR); *ActiGraph GT3X* (left waist; NR; 15 sec; NR) | Intensity (Steps, Time spent in MVPA) | Observation (Video); Indirect Calorimetry | Intraclass correlation coefficients; Lin’s concordance coefficients; Bland-Altman analyses; repeated-measures ANOVA | The PiezoRx PA monitor appears to be a valid and reliable measure of step count and MVPA in this diverse sample of adults. | Y/NR | Some |
| 364 | O’Brien et al. (2020, United Kingdom) | Adults with rheumatoid arthritis (N=19; 53.7±12.5 yrs; 86% females; NR) | Activities of daily life (66 minutes) | *ActiGraph GT3X+* (right hip; Actilife; 1 minute; NR); *ActivPAL3* (right thigh; PAL Connect; 15 sec; NR) | Intensity (Energy expenditure, Steps); Posture/Activity Type (Sitting/lying, standing, stepping) | Indirect Calorimetry; Observation (Video) | Bland-Altman analyses; Accuracy | In conclusion, the activPAL3™ accurately quantifies sedentary, standing and stepping time in RA. The RA-specific cut-points offer a validated measure of sedentary time, light-intensity PA and moderate-intensity PA in these patients, and demonstrated superior accuracy for estimating free-living sedentary time, compared to non-RA cut-points. | N/N | High/High |
| 365 | O’Connell et al. (2016, Ireland) | Adults (N=15; 21.1±1.1 yrs; 53% females; NR) | Walking (NR) | *Garmin Vivofit* (non-dominant wrist; NR; NR; NR); *NL-2000* *pedometer* (waist; NR; NR; NR); *Withings Pulse O2* (waist; NR; NR; NR); *Fitbit One* (chest; NR; NR; NR) | Intensity (Steps) | Observation (Video); Wearable (activPAL micro (thigh)) | Repeated measures ANOVA; Bland-Altman analyses | All activity monitors tested were accurate in their step detection sensitivity and are valid monitors for physical activity quantification over the variety of different surfaces tested, when wearing both running shoes and hard-soled dress shoes, and over a timeframe necessary for accumulating the recommended daily step count of 10,000 steps. | N/N | High |
| 366 | O’Connell et al. (2017, Ireland) | Adults (N=37; 39±13.9 yrs; 67.6% females; NR) | Activities of daily life (NR) | *ActivPAL micro* (right thigh; ActivPAL™ software; NR; NR); *NL-2000 pedometer* (left hip; NR; NR; NR); *Withings Pulse O2* (right hip; NR; NR; NR); *Fitbit One* (chest; NR; NR; NR); *Jawbone UP* (right wrist; NR; NR; NR) | Intensity (Steps) | Observation (Video) | Negative binomial regression analysis; Simple comparison | The Withings™ activity monitor performed best with regard to specificity during the activities of daily living tested. | N/N | High |
| 367 | O’Driscoll et al. (2013, Australia) | Adults (N=50; 45.5±2 yrs; 32% females, NR) | Sleep (1 night) | *SenseWear Pro3 Armband* (upper right arm; SenseWear Professional Software, v6.1, 30 sec, NR) | Biological state (Total sleep time) | Polysomnography | Linear regression analyses; Wilcoxon signed-rank tests; Cohen’s Kappa; Agreement; Sensitivity; Specificity; Predictive value for sleep; Bland-Altman analyses | The SenseWear armband provides a reasonable estimation of sleep but a poor estimation of wake. | N/N | High |
| 368 | O’Driscoll et al. (2020; United Kingdom) | Adults (N=59; 44.4±14.1 yrs; 69.5% females; NR) | Activities of daily life (50 minutes) | *Fitbit Charge 2* (wrist; NR; NR; random forest); *SenseWear Armband Mini* (non-dominant upper arm; NR; NR; random forest); *ActiGraph GT3X* (non-dominant wrist; NR; NR; random forest) | Intensity (Energy expenditure) | Indirect Calorimetry | Pearson correlation; Root mean squared error; Mean absolute percentage error; Equivalence tests; Repeated-measures ANOVA; Pairwise t-tests | A high degree of accuracy in EE estimation was achieved by applying non-linear models to wearable devices which may offer a means to capture the energy cost of free-living activities. | N/N | Some |
| 369 | O’Hare et al. (2014, Germany) | Adults (N=20; 30±6 yrs; 45% females; NR) | Sleep (1 night) | *Actiwatch 2* (non-dominant wrist; NR; 30 sec; NR) | Biological state (Total sleep time) | Polysomnography | Bland-Altman analyses; Pearson correlation; One-way ANOVA; Pairwise t-tests | The radio-frequency biomotion sensors provided similar accuracies for sleep/wake determination in normal subjects as the actigraph used in this study and slightly improved estimates of TST, SOL, and WASO | N/N | High |
| 370 | Ohkawara et al. (2011, Japan) | Adults (N=66; 42.4±13.5 yrs; 53% females; NR) | Activities of daily life (4.5 hours) | *Micro Electro Mechanical Systems LIS3LV02DQ* (waist; NR; 10 sec; algorithm reported) | Intensity (Energy expenditure) | Indirect Calorimetry | Pearson correlation; Linear and non-linear regression models; One-way ANOVA; Paired t test; Bland–Altman analyses | The use of a triaxial accelerometer in combination with a GRPACA permits more accurate and immediate estimation of daily physical activity intensities, compared with previously reported cut-off classification models. | N/N | Some |
| 371 | Oomen et al. (2018, Netherlands) | Adults (N=40; Substudy 1: 24.2±5.8 yrs; Substudy 2: 28.8±11.1 yrs; Substudy 3: 22.7 ±7 yrs; 32.5% females; NR) | Substudy 1: Treadmill (30 minutes); Substudy 2 and 3: Treadmill (36 minutes) | *Activ8* (thigh; NR; NR; NR) | Intensity (Energy expenditure) | Indirect Calorimetry | Correlation; Two-way repeated measures ANOVA | The results of this study show an improved correlation between EE measured by indirect Calorimetry and the Activ8 activity device (R2 from 0.91 to 0.95); a decrease in differences between substudy A and substudy B considering EE measured (indirect Calorimetry) and calculated (Activ8 calculation) was observed. | NR/NR | High |
| 372 | Osawa et al. (2013, Japan) | Adults (N=20; 32.5±15.3 yrs; 20% females; NR) | Walking (18 minutes) | *Acos FS500* (waist; NR; NR; NR); *Omron HJ301* (waist; NR; NR; NR); *Yamax EX700* (waist; NR; NR; NR); *Tanita FB727* (waist; NR; NR; NR); *Citizen TW600* (waist; NR; NR; NR) | Intensity (Steps; Energy expenditure) | Observation (Manually counted); Indirect Calorimetry | Correlation; Two-way repeated measures ANOVA; Paired t-tests | Feedback about energy expenditure is somewhat inaccurate. In contrast, step counts are very accurate, and thus pedometers are useful tools with which to indicate daily exercise levels. | N/N | High |
| 373 | Pallin et al. (2014, Ireland) | Adults with obstructive sleep apnea syndrome (N=103; 54.8±13.9 yrs; 18% females; NR) | Sleep (1 night) | *Actiwatch-4* (non-dominant-wrist; Actiwatch Sleep Score 5.32; 30 sec; NR) | Biological State (Total sleep time) | Polysomnography | Paired t-test; Wilcoxon matched pair-tests; Bland-Altman analyses; Accuracy; Specificity; Sensitivity | We conclude that the biomotion sensor provides a viable alternative to actigraphy for sleep estimation in the assessment of obstructive sleep apnoea syndrome. | N/N | High |
| 374 | Pambianco et al. (1990, United States) | Overweight and normal weight adults (N=20; 20-35 yrs; 50% females; NR) | Treadmill (15 minutes) | *Caltrac* (hip; NR; NR; equation reported) | Intensity (Energy expenditure) | Indirect Calorimetry | Repeated measure ANOVA; Pearson correlation | The Caltracs seem well suited for studies of activity level of groups, but may be less useful in estimating an indvidual´s activity. | N/NR | High |
| 375 | Papazoglou et al. (2006, Italy) | Obese adults (N=142; 46.9±14.2 yrs; 74% females; NR) and lean and overweight adults (N=25; 36.5±14.7 yrs; 80% females; NR) | Treadmill; Activities of daily life (45 minutes) | *SenseWear Pro 2* (right upper arm; InnerView Research Software v4.0; NR; Harris-Benedict (H-B) equations) | Intensity (Energy expenditure) | Indirect Calorimetry | Univariate and multivariate linear regression analyses; Bland-Altman analyses | The SWA is an easy to handle, practical, new portable device for measuring energy expenditure. The accuracy of the SWA appeared to be poor in the obese subjects we examined, especially those with high REE both in rest and exercise. | N/NR | Low |
| 376 | Paquet et al. (2007, Canada) | Adults (N=15, 39.3±15.1 yrs, 53% females, NR) | Sleep (1 night and 2 daytime recovery sleeps) | *Actiwatch-L* (non-dominant wrist, Actiware 5.0; 1 minute; Algorithms reported) | Biological State (Total sleep time) | Polysomnography | Two-way repeated measures ANOVAs; Simple effect analyses; Post hoc Tukey HSD test; Huynh-Feldt correction for sphericity; Epsilon values; Original degrees of freedom; Bland-Altman analyses; Sensitivity; Specificity; Accuracy | The very low ability of actigraphy to detect wakefulness casts doubt on its validity to measure sleep quality in clinical populations with fragmented sleep or in situations where the sleep-wake cycle is challenged, such as jet lag and shift work. | N/N | Some |
| 377 | Paradiso et al. (2020, Canada) | Adults (N=14; 23±4.2 yrs; 57% females; NR) | Walking, treadmill and exercise (18 minutes) | *Mi Band 2* (each wrist; NR; NR; NR) | Intensity (Steps) | Observation (Video) | Percentage error; Sample t-tests; Bland-Altman analyses | The Mi Band significantly underestimated heart rate during exercise. Overall, caution is required when interpreting the steps recorded (at slower speeds) and heart rate measurements. | NR/N | High |
| 378 | Parak et al. (2017, Finland) | Adults (N=24; 36.2±8.2 yrs; 46% females; NR) | Treadmill (NR) | PulseOn (wrist; NR; NR; Artifact correction algorithm) | Intensity (Energy expenditure) | Indirect Calorimetry; Chest strap HR device (RS800CX) | Mean absolute error; Mean absolute percentage error; Pearson or Spearman correlation; Bland-Altman analyses | When combined with physiological modeling, wrist-worn OHR may be used for an estimation of EE, especially during higher intensity running, and VO_2Max_, even during submaximal self-paced outdoor recreational running. | NR/Y | Some |
| 379 | Park et al. (2011, Japan) | Adults (N=18; 29.6±5.5 yrs; 50% females; NR) | Treadmill (16 minutes) | *Kenz Lifecorder EX* (waist; Physical Activity Analysis Software; NR; NR); *Actimarker* (waist; NR; NR; NR); *Omron* *Active Style Pro* (waist; NR; NR; NR) | Intensity (Energy expenditure; Steps) | Indirect Calorimetry; Observation (Video) | Percentage error; One-way repeated measures ANOVA; Two-way repeated measures ANOVA; Multiple stepwise regression analysis | These results suggest that LC and AM can cause errors in step-count functions at a low walking speed. Furthermore, LC may show low accuracy of the METs measurement during walking altered according to step frequency and speed, whereas AM and ASP, which are tri-axial accelerometers, are more accurate but the degree of the percentage error is affected by step frequency. | N/NR | High |
| 380 | Park et al. (2016, Japan) | Adults (N=18; 76.9±3.7 yrs; 67% females; NR); Frail adults (N=16; 74.5±7.4 yrs; 63% females; NR); Adults walking with cane (N=7; 83.1±8.2 yrs; 57% females; NR); Adults in wheelchairs (N=8; 86.5±5.3 yrs; 100% females; NR) | Walking (9 minutes) | *Kenz Lifecorder EX* (waist; Physical Activity Analysis Software v1.0; NR; NR); *Actimarker* (waist; NR; NR; NR); *Omron* *Active Style Pro* (waist; NR; NR; NR) | Intensity (Energy expenditure; Steps) | Indirect Calorimetry; Observation (Manually- counted) | Absolute percentage error; Three-way ANOVA; One-way ANOVA, Two-way ANOVA, Bland-Altman analyses | Among frail older people with assistive devices, the gait intensity error was smaller than for step count error. To accurately assess the steps walked or the gait intensity among frail older people using assistive devices, more study is needed on these groups of participants. | Y/NR | High |
| 381 | Passler et al. (2019, Germany) | Adults (N=24; 23±2.1 yrs; 46% females; NR) | Running and treadmill (20 minutes) | *Polar V800* (wrist; Polar Flow App; NR; NR); *Garmin Forerunner 920XT* (wrist; Garmin Connect App; NR; NR); *Garmin Vivosmart HR* (wrist; Garmin Connect App; NR; NR); *TomTom Touch* (wrist; TomTom My Sports; NR; NR); *Withings Pulse O_X_* (wrist; Nokia Health Mate App; NR; NR) | Intensity (Energy expenditure) | Indirect Calorimetry | Paired sample t-tests; Mean absolute percentage errors; Intraclass correlation coefficient; Bland-Altman analyses | The tested devices did not show valid results concerning the estimation of VO2max and EE. Hence, the current wrist-worn activity trackers are most likely not accurate enough to be used for neither purposes in sports, nor in health care applications. | N/N | High |
| 382 | Patel et al. (2007, United States) | Adults with mild-to-severe chronic obstructive pulmonary disease (N=8; 61.5±4.3 yrs; 50% females; NR) | Walking (12 minutes) | *SenseWear Pro* (right upper arm; InnerView Reasearch Software v2.2; NR; proprietary multiple non-linear regression equation) | Intensity (Energy expenditure) | Indirect Calorimetry | Intraclass correlation coefficient; Pearson correlation; Mixed model analyses; Bland-Altman Analyses | This physiologic activity monitor provides a valid and reproducible estimate of energy expenditure during slow to moderate paced walking in a laboratory setting and represents an objective method to assess activity in COPD subjects. | NR/NR | High |
| 383 | Peiris et al., (2017, Australia) | Adolescents and young adults with Down syndrome and mild to moderate intellectual disability (N=10; 22±2 yrs; 50% females; NR) | Activities of daily life (2x 60 minutes) | *SenseWear* (each upper arm; NR; NR; proprietary algorithm); *RT3* (right hip; NR; NR; proprietary algorithm) | Intensity (Energy expenditure) | Indirect Calorimetry | Paired t-tests; Pearson correlation; Coefficient of determination; Bland-Altman analyses | SenseWear steps and RT3 activity count thresholds can be used to monitor physical activity in young people with Down syndrome, though energy expenditure estimates should be used with caution in this population. | NR/NR | High |
| 384 | Petrucci et al. (2018, United States) | Adults (N=21, 24.8±3.57 yrs, 46% females, NR) | Treadmill (NR) | *Misfit Shine* (dominant wrist, right hip; NR; NR; proprietary algorithms) | Intensity (Steps; Physical activity counts) | Observation (Video); Wearable (ActiGraph GT3X+ (dominant wrist, right hip)) | Paired t-tests; Linear mixed effects models; Percent difference | Although there were systematic errors in step estimates from the MS, it was sensitive to changes during LAB and FL, and may be a useful tool for interventionists where tracking changes in PA is an important exposure or outcome variable. | N/NR | High |
| 385 | Pigeon et al. (2018, United States) | Adults (N=20; 30.1±13.1 yrs; 35% females; NR) | Sleep (1 night) | *myCanadian watch* (non-dominant wrist; CURA System; 30 sec; proprietary algorithm); *Actiwatch 2* (non-dominant wrist; Actiware v6.0.2; 30 sec; proprietary algorithm) | Biological State (Total sleep time) | Polysomnography | Kappa statistics; Agreement; Positive predictive value | The kappa statistic for MC is consistent with a high level of agreement with PSG. | NR/N | High |
| 386 | Pino-Ortega et al. (Spain, 2021) | Adults (N=26; 71.2±3.2 yrs; 50% females; NR) | Outdoor walking (20 minutes) | *Xiaomi Mi Band versions 2.0., 3.0. and 4.0* (all wrist; NR; NR; NR) | Intensity (Steps) | Wearable (Inertial devices WIMU PRO) | Bland-Altman analyses; Intraclass correlation coefficient | The accuracy of Mi Band Xiaomi 2.0., 3.0. and 4.0. may be considered as good to count the number of steps for physical activity monitoring, whereas distance estimation is considered questionable. | N/N | High |
| 387 | Pitchford & Yun (2010, United States) | Adults with Down Syndrome (N=20; 29.25±12.45 yrs; 60% females; NR) and adults without disability (N=24; 32.08±13.10 yrs; 58% females; NR) | Walking (6 minutes) | *Omron HJ-112* (waist, NR; NR; NR); *Yamax Digiwalker SW-200* (waist; NR; NR; NR) | Intensity (Steps) | Observation (Manually counted) | Absolute error score; Intraclass correlation coefficients; Independent t-tests; Repeated-measures ANOVA | The study concludes that pedometer measurement error is significantly different for adults with DS but also that piezoelectric pedometers can be used in the future to measure walking activity for adults with and without DS. | NR/NR | High |
| 388 | Pollak et al. (2001, United States) | Adults (N=14; 21-72 yrs; 50% females; NR) | Sleep (7 nights) | *CSA* (non-dominant wrist; NR; 30 sec; NR); *ActiTrac* (non-dominant wrist; NR; 30 sec; NR) | Biological State (Sleep-wake state) | Polysomnography | Predictive values; Agreement; MANOVA; Paired or unpaired t-tests | Low PV’s and overestimation of sleep currently disqualify actigraphy as an accurate sleep-wake indicator. | N/NR | High |
| 389 | Pomeroy et al. (2011, United States) | Adults (N=54; 20-34 yrs; 50% females; ≥50% American Indian ancestry) | Walking (540 meters) | *Accusplit AX120* (hip; NR; NR; NR); *ActiGraph 7164* (hip; NR; NR; NR); *Dynastream AMP-331* (ankle; NR; NR; NR) | Intensity (Steps) | Observation (Manually- counted) | Accuracy; Spearman correlation; Equality of the correlations; Generalized linear models; Bland-Altman analyses | The AMP was the most accurate of the instruments compared with observed steps in the laboratory-based walk test followed by the MTI and the AX120. | N/N | High |
| 390 | Pope et al. (2019, United States) | College students (N=21; 24.5±3.5 yrs; 66.7% females; Non-Hispanic White (N=12), Asian (N=7), Hispanic (N=1), Middle-Eastern (N=1)) | Exergaming (20 minutes) | *Apple Watch* (wrist; NR; NR; NR); *Fitbit Surge HR* (wrist; NR; NR; NR); *TomTom Multisport Cardio Watch* (wrist; NR; NR; NR); *Microsoft Band* (wrist; NR; NR; NR) | Intensity (Energy expenditure) | Wearable (ActiGraph GT3X+-BT (hip)) | Intraclass correlation coefficient; Repeated-measures ANOVA; Bland-Altman analyses; Linear regression | Smartwatch EE measurements were less valid. | N/N | High |
| 391 | Pope et al. (2019, United States) | Adults (N=25; 23.52±1.04 yrs; 52% females; NR) | Treadmill and resting (80 minutes) | *Microsoft Band* (wrist; NR; NR; NR); *Fitbit Surge HR* (wrist; NR; NR; NR); *TomTom Cardio Watch* (wrist; NR; NR; NR); *Apple Watch* (wrist; NR; NR; NR) | Intensity (Energy expenditure) | Indirect Calorimetry | Pearson correlation; Mean absolute percent error; Equivalence testing | MB and AW appear most accurate for EE estimation. However, smartwatch manufacturers may consider concentrating most on improving EE estimate accuracy during MPA. | N/N | High |
| 392 | Powell et al. (2016, Ireland) | University staff and students (N=56; 39.9±11.5 yrs; 55% females; NR) | Activities of daily life (56 minutes) | *SenseWear Pro3* (right upper arm; SenseWear Professional v 6.1; NR; NR); *ActivPAL3 Micro* (right thigh; v 7.2.32; NR; NR) | Intensity (Energy expenditure) | Indirect Calorimetry | Paired samples t-tests; Bland-Altman analyses | All of the activities (excluding SWP3 sweeping) were significantly different from the criterion measure. Although the SWP3 predicted METs are more accurate than their aP3M equivalent, the predicted MET values from both devices are significantly different from the criterion measure for the majority of activities. | N/N | High |
| 393 | Pribyslavska et al. (2018, United States) | Adults (N=34; 25.8±4.9 yrs; 32% females; NR) | Treadmill and cycling (40 minutes) | *Fitbit Surge* (wrist; NR; NR; NR); *Garmin vívofit* (wrist; NR; NR; NR); *SenseWear Mini* (upper left arm; NR; NR; proprietary algorithm) | Intensity (Energy expenditure) | Indirect Calorimetry | Percent error; One-sample t-test; Mean absolute percentage error; Paired-sample t-test; Wilcoxon signed ranks test | The activity monitors tended to underestimate EE during moderate and vigorous treadmill and cycling activities. The EE estimates from the activity monitors did not account for the energy cost met by anaerobic means during activity, as suggested by the higher EPOC-adjusted EE error rates. | NR/N | High |
| 394 | Price et al. (2017, Australia) | Adults (N=14; 23.0±6.0 yrs; 21% females; NR) | Treadmill (24 minutes) | *Fitbit One* (right hip, NR; NR; proprietary algorithm); *Garmin Vivofit* (right wrist; NR; NR proprietary algorithm); *Jawbone UP* (left wrist; NR; NR; proprietary algorithm) | Intensity (Energy expenditure) | Indirect Calorimetry | Bland-Altman analyses; Pearson correlation; Paired t-tests; Absolute difference; Mean percentage difference, Regression | Energy expenditure reported by the devices distinguished between walking and running, with a general increase as exercise intensity increased. However, the reported energy expenditure from these devices should be interpreted with caution, given their potential bias and error. | NR/N | High |
| 395 | Prieto-Centurion et al. (2016, United States) | Adults (N=12; <40 yrs; 58% females, NR); Patients recovering from COPD exacerbations (N=4; 69±10 yrs; 0% females; NR) | Activities of daily life (15 minutes); Indoor walking (6 minutes) | *Fitbit Zip* (waist; NR; NR; NR); *Fitbit Force* (non-dominant wrist; NR; NR; NR); *ActiGraph wGT3X-BT* (hip, wrist; NR; NR; NR) | Intensity (Steps) | Observation (Manually counted) | Bland-Altman analyses; Paired t-tests | The accuracy of commercially available pedometers in healthy volunteers is highly variable. The top-performing pedometer in our study, the Fitbit Zip, ® accurately measures step counts in both healthy volunteers and patients recovering from COPD exacerbations. | NR/N | High |
| 396 | Radtke et al. (2021, Switzerland) | Adults (N=20; 38±11 yrs; 65% females; NR) | Activities of daily life (approx. 60 minutes) | *ActivPAL micro* (thigh; activPAL Research Edition v7.2.32; 15 sec; proprietary algorithm); *ActiGraph wGT3X-BT* (thigh; Actilife v6.13.3; 15 sec; inclinometer algorithm) | Poster/Activity Type (Sitting, standing, stepping, transition) | Observation (Direct) | Accuracy; McNemar test | The ActiGraph appears to be slightly more sensitive than the activPAL with respect to the measurement of sitting and postural transitions of short duration, whereas the activPAL seems to be slightly more accurate in capturing standing postures. | N/N | High |
| 397 | Rampichini et al. (2016, Italy) | Adults (N=23; 48.0±27.3 yrs; 57% females; NR) | Indoor cycling and outdoor walking protocol (20 minutes) | *Actiheart* (chest; NR; NR; Schofield equation) | Intensity (Energy expenditure) | Indirect Calorimetry | One-way ANOVA; Two-way ANOVA; Pearson correlation; Bland-Altman analyses | Without individual calibration, WMS underestimated EE measured by IC during all physical activities, with the lower correlation coefficient in institutionalized individuals. An individual calibration of WMS seems to be mandatory to obtain accurate estimations of EE, especially in institutionalized elderly people. | N/N | High |
| 398 | Ray et al. (2014, United States) | Healthy adults and adults with sleep disorders (N=54; 51±12 yrs; 54% females; NR) | Sleep (1 night) | *ActiGraph MTI 7164* (non-dominant wrist, hip; MTI ActiLife 5; 1 minute; Cole–Kripke algorithm) | Biological State (Total sleep time) | Polysomnography | Wilcoxon test; Spearman correlation; Agreement; Sensitivity; Specificity; Bland-Altman analyses | The original wrist actigraphy data showed modest correspondence with PSG, and much less correspondence was found between hip actigraphy and PSG. | N/NR | Some |
| 399 | Raymond et al. (2015, Australia) | Older inpatients (N=12; 79.8±7.26 yrs; NR; NR) | Activities of daily life (min. 33 minutes) | *Positional Activity Logger 2* (thigh; PALCalcs 2009; NR; NR) | Posture/Activity Type (Upright, sitting, lying, walking) | Observation (Video) | Paired t-tests; Bland-Altman analyses; Pearson correlation | The PAL2 is a valid tool for quantifying activity levels, position transitions, and within-person changes in gait speed in older inpatients. | NR/NR | High |
| 400 | Razjouyan et al. (2017, United States) | Adults with self-reported sleep problems (N=21; 50.8±12.8 yrs; 48% females; NR) | Sleep (1 night | *Actiwatch-L* (dominant wrist; Action4; 1 minutes; Cole-Kripke algorithm), *BioPatch ZephyrLife* (chest, MATLAB software; NR; NR) | Biological State (Total sleep time) | Polysomnography | Sensitivity; Specificity; Accuracy; Student t tests; Mann-Whitney U test; Pearson and Spearman correlations; Bland-Altman analyses; Binary logistics regression model | Combination of sleep postural/position changes and body acceleration improved detection of sleep/wake epochs compared to wrist acceleration alone. The chest sensors also improved estimation of sleep parameters of interest with stronger agreement with PSG. | N/N | High |
| 401 | Reddy et al. (2018, United States) | Adults (N=20; 27.5±6.0 yrs; 55% females, NR) | Treadmill and activities of daily life (Approx. 4 hrs) | *Fitbit Charge 2* (wrist; Fitabase; NR; NR); *Garmin vívosmart HR+* (wrist, Garmin-Connect App v 3.17; NR; NR) | Intensity (Energy expenditure) | Indirect Calorimetry | Mean absolute percentage error; Pearson correlation; Bland-Altman analyses; Student t test; Matched paired t tests; One-way analysis ANOVA; Concordance class correlation | Two common wrist-worn devices (Fitbit Charge 2 and Garmin vívosmart HR+) show good HR accuracy, with a small negative bias, and reasonable EE estimates during low to moderate-intensity exercise and during a variety of common daily activities and exercise. | N/N | High |
| 402 | Redfield et al. (2013, United States) | Adults with trans-tibial amputations (N=8; 53±11.6 yrs; 25% females; NR) | Activites of daily life (NR) | *ActiGraph GT3X+* (ankle (prosthesis), thigh; NR; NR; Binary decision tree (BDT) algorithm) | Posture/Activity Type (Doffed, sitting, standing, active) | Observation (Direct) | Sensitivity; Accuracy; Confusion matrix | The classifier achieved a mean accuracy of 96.6% (SD=3.0%). | N/NR | High |
| 403 | Reece et al. (2015; United States) | Adults (N=22; 45±11.7 yrs; 50% females; NR) | Activities of daily life (30 minutes) | *SenseWear* (upper left arm; software 7.0; 1 minutes; NR) | Intensity (Energy expenditure) | Indirect Calorimetry | Pearson correlation; Intraclass correlation coefficient; Bland-Altman analyses; two-way repeated-measures ANOVA; Simple effect tests; Paired samples t-tests | The SWA and IC EE rates were strongly correlated during sedentary and light activity office behaviors. However, the SWA may under predict EE during office work (standing or sitting) and when standing motionless, making it slightly less sensitive than IC. | NR/NR | High |
| 404 | Reeve et al. (2014, Australia) | Participants with strength training experience (N=18, 22.7±4.5 yrs; 38% females; NR) | Resistance training (NR) | *SenseWear Mini* (arm; Software v7; NR; NR); *BodyMedia FIT* (arm; NR; NR; NR) | Intensity (Total Energy expenditure) | Indirect Calorimetry | Pearson correlation; Percent mean change | The SenseWear Armband Mini and BodyMedia FIT provide a valid and reliable measure of energy expenditure during resistance training. There was no significant difference in validity or reliability observed between the SenseWear Armband Mini and BodyMedia FIT. | N/NR | High |
| 405 | Reid & Dawson (1999, Australia) | Adults (N=32; Group 1: 21.2±2.7 yrs; Group 2: 43.9±6.8 yrs; 21.9% females; NR) | Sleep (1 night) | *Z80-32k V1* (non-dominant wrist; NR; 30 sec; NR) | Biological State (Sleep-wake state) | Polysomnograhy | Accuracy; Sensitivity | Results suggest that wrist activity monitoring is a valid measure of sleep/wake activity and sleep duration, in a simulated shiftwork environment | N/NR | High |
| 406 | Renfrew et al. (2020, United Kingdom) | Adults (N=16; 34.9±3.36 yrs; 44% females; NR) | Treadmill (15 minutes) | *PALlite 3c* (dominant leg; PALanalysis; NR; NR) | Intensity (Steps) | Observation (Video) | One-way ANOVA; Bland-Altman analyses | Clinicians could consider both devices to objectively measure step count with people who are prescribed foot drop orthoses, thus quantifying orthotic use. | N/N | High |
| 407 | Rennie et al. (2000, United Kingdom) | Adults (N=8; mean 31.3 yrs; 38% females; NR) | Activities of daily life (24 hours) | *HR + M instrument* (chest; NR; NR; NR) | Intensity (Energy expenditure) | Whole-body Calorimeter | Percentage error | This preliminary test of HR + M demonstrates its ability to estimate EE and the pattern of EE and activity throughout the day. | NR/NR | High |
| 408 | Riel et al. (2016, Denmark) | Adults (N=30; 27.9±4.2 yrs; 50% females; NR) | Treadmill (6 minutes) | *Mother* (hip; Senseboard software; NR; NR); *ActiGraph wGT3X-BT* (hip; ActiLife Pro 6 software; NR; NR) | Intensity (Steps) | Observation (Video) | Wilcoxon’s signed ranks test; Two-way random effects model; Absolute agreement; Intraclass correlation coefficient; Root Mean Square Error; Bland-Altman analyses; Percent differences | Mother provides valid measures of steps at walking speeds of 3.2, 4.8, and 6.4 km/h with clinically irrelevant deviations compared to a hand tally while ActiGraph only provides valid measurements at 6.4 km/h based on the 3% criterion. These results have significant potential for valid objective measurements of low walking speeds. | N/N | High |
| 409 | Robert-Lewis et al. (2021, United Kingdom) | Adults with progressive muscle diseases (N=20; NR; NR; NR) | Posture, walking and cycle ergometer (40 minutes) | *Fitbit Charge 2* (wrist; Fitabase; NR; NR) | Intensity (Steps; Energy expenditure (MET)) | Observation (Video; Direct) | Kappa statistics; Absolute measurement error; Spearman correlation; Bland-Altman analyses | Fitbit had satisfactory measurement properties for monitoring physical activity in adults with progressive muscle diseases. However, Fitbit should not be considered an exact step counter, heart rate monitor or calorimeter and Fitbit active minutes are not synonymous with MVPA time. | N/N | High |
| 410 | Roberts et al. (2020, United States) | Adults (N=8; 40.75±4.84 yrs; 63% females; NR) | Sleep protocol (4 nights) | *Apple Watch* (wrist of non-dominant hand; NR; NR; NR); *Oura Ring* (finger; NR; 30 sec; NR); *ActiGraph GT9X Link* (wrist of dominant hand; NR; NR; NR); *Actiwatch Spectrum Plus* (wrist of non-dominant hand; NR; 30 sec; NR) | Biological State (Total sleep time) | Polysomnography | Accuracy; Sensitivity; Specificity; Precision; Cohen’s kappa (κ); Signal detection theory d-prime (d′) | Data from multisensor consumer wearables are strongly correlated with reference devices at the epoch level and can be used to develop epoch-by-epoch models of sleep–wake rivaling existing research devices. | N/NR | High |
| 411 | Roos et al. (2017, Switzerland) | Adults (N=20; 23.90±1.92 yrs; 40% females; NR) | Treadmill (33 minutes) | *Suunto Ambit2* (wrist; Suunto Movescount; NR; NR); *Garmin Forerunner920XT* (wrist; Garmin Connect; NR; NR), *Polar V800* (wrist, Polar Flow; NR; NR) | Intensity (Energy expenditure) | Indirect Calorimetry | Repeated-measures ANOVA; Pearson correlation; Mean absolute error; Mean absolute percentage error; Root mean square error, Bland-Altman analyses | To estimate energy expenditure during aerobic running, the Polar V800 is recommended. By contrast, the other two watches either significantly overestimated or underestimated energy expenditure during most running intensities. The energy expenditure estimations generated during anaerobic exercises revealed large measurement errors in all tested sport watches | N/N | High |
| 412 | Rothney et al. (2010, United States) | Adults (N=34; 40.1±12.2 yrs; 68% females; NR) | Activities of daily life (24 hours) | *ActiGraph GT1M* (hip; Actilife v.4.3.0; 1 sec; two-regression model) | Intensity (Energy expenditure; Time spent in sedentary, LPA, MVPA) | Room Calorimetry | Absolute percent difference; Mean absolute error; Mean squared error; Paired t-tests; Bland-Altman analyses | The two-regression model with LPF showed good agreement with total EE measured using room calorimeter and DLW. However, the individual variability in assessing time spent in sedentary, low, and moderate PA intensities and related EE remains significant. | N/N | High |
| 413 | Rousset et al. (2015; France) | Adults (N=49; males: 44.2±4.6 yrs, females: 46.2±5.6 yrs; 53% females; NR) | Activities of daily life (17 hours) | *Actiheart* (chest; Finder2E software; 1 minute; Schofield equation); *SenseWear Pro-3* (right upper arm; Finder2E software; NR; NR) | Intensity (Total Energy expenditure) | Indirect Calorimetry | T-tests; Paired t-tests; Bland-Altman analyses | Our results show that both monitors are appropriate for estimating TEE. Armband is more effective than Actiheart at the individual level for daily light-intensity activities | N/NR | Some |
| 414 | Rowlands et al. (2016, United Kingdom) | Adults (N=34; 27.2±5.9 yrs; 58.8% females; NR) | Activities of daily life (120 minutes) | *GENEActive* (non-dominant wrist; NR; 15 sec; NR); *ActiGraph GT3X+* (non-dominant wrist; NR; 15 sec; NR) | Posture/Activity Type (Lying, sitting, upright) | Observation (Direct) | Accuracy | Data support the efficacy of the Sedentary Sphere for classification of posture from a wrist-worn accelerometer in adults. Importantly, the approach is equally valid with data from both the GENEActiv and ActiGraph accelerometers. | N/Y | High |
| 415 | Rowlands et al. (2004, United Kingdom) | Adults (N=15, 20.7±1.4 yrs, 0% females, NR) | Treadmill, posture and exercise (34 minutes) | *RT3 triaxial accelerometer* (right hip, NR; 1 minutes; NR); *Tritrac T303A* (hip; NR; 1 minute; NR) | Intensity (Counts) | Indirect Calorimetry | Pearson correlation; Two-way mixed model ANOVA | The RT3 accelerometer is a good measure of physical activity for boys and men. However, moderate and vigorous intensity count thresholds differ for boys and men when the predominant activities are walking and running. RT3 counts are significantly higher than Tritrac counts for a number of activities. | NR/NR | High |
| 416 | Rüdiger et al. (2019, Germany) | Adults (N=22; 74.8±5.9 yrs; 81.81% females; NR | Walking (200 meters) | *Polar M400* (non-dominant arm; NR; NR; NR) | Intensity (Steps) | Observation (Direct); Wearable (Omron Walking Style Pro HJ-720IT-E2 (waist)) | Kruskal-Wallis test; Lin´s Concordance correlation coefficient; Bland-Altman analyses | The Polar M400 activity tracker accurately assesses steps during walking in older adults. Nevertheless, a slight overestimation compared to the pedometer was observed, which should be considered when using the activity tracker for tracking steps over a longer period of time. | N/N | High |
| 417 | Rupp & Balkin (2011, United States) | Adults (N=29; 24.3±5.4 yrs; 31% females; NR) | Sleep (1 night) | *Motionlogger Watch* (non-dominant wrist; Action-W Version 2; 30 sec; Cole–Kripke algorithm); *Actiwatch-64* (non-dominant wrist; Actiware-Sleep, Version 3.4; 30 sec; NR) | Biological State (Sleep/wake identification; Total sleep time) | Polysomnography | Repeated measures ANOVAs; Sensitivity: Specificity; Agreement | It was concluded that the Motionlogger provided nominally better agreement with PSG, and that actigraphy generally constitutes a reasonably reliable tool for producing objective measurements of sleep/ wake, but that users should remain mindful of its limitations. | N/NR | Some |
| 418 | Ryan & Gormley (2013, Ireland) | Adults (N=26; 24.7±4.4 yrs; 57.7% females; NR) | Treadmill (20 minutes) | *Intelligent Device for Energy Expenditure and Activity* (each thigh, each foot, chest; NR; NR; proprietary algorithm); *Sensewear Pro* (upper arm; NR; NR; new algorithm); *RT3* (hip; NR; NR; proprietary algorithm) | Intensity (Energy expenditure) | Indirect Calorimetry | Bland-Altman analyses; Pearson or Spearman correlation | Although the RT3 provided the best estimate of resting EE in adults and children, the SWA provided the most accurate estimate of EE across a range of physical activity intensities | N/NR | High |
| 419 | Ryan et al. (2006, United Kingdom) | Adults (N=20, 34.5±6.9 yrs, 60% females, NR) | Treadmill (25 minutes) and outdoor walking (1500 m) | *ActivPAL* (thigh; activPAL Professional Research Edition; NR; proprietary algorithm); *Yamax Digiwalker SW-200* (waist; NR; NR; NR), *Omron HU-109-E* (waist, NR; NR; NR) | Intensity (Steps) | Observation (Video) | Bland-Altman analyses; Intraclass correlation coefficient; Absolute percentage error | The activPAL monitor is a valid and reliable measure of walking in healthy adults. Its accuracy is not influenced by walking speed. The activPAL may be a useful device in sports medicine. | N/Y | High |
| 420 | Ryan et al. (2008, United Kingdom) | Adults with chonic low back pain (N=10, 51±10 yrs; 90% females; NR) | Activities of daily life (NR) | *ActivPAL* (right thigh; activPAL Professional Research Edition; NR; NR) | Posture/Activity Type (Sitting/lying, standing, walking); Intensity (Steps) | Observation (Video) | Bland-Altman analyses; Percentage agreement; Sensitivity; Positive predictive values | The activPAL activity monitor is a valid device for measuring postural physical activity, step count and cadence in people with CLBP in a semi-constrained environment. | N/Y | High/High |
| 421 | Ryde et al. (2012, Australia) | Adults (N=13, 30±6.5 yrs, 69% females, NR) | Posture (approx. 5 minutes) | *ActivPAL3* (NR; NR; NR; NR), *ActiGraph GT3X+* (NR; NR; NR; NR) | Posture/Activity type (Sitting time; sit-to-stand transitions) | Observation (Video) | Intraclass correlation coefficient; Mean differences | Findings indicated that the SP and AP3 provided highly accurate measures of desk based sitting time and transitions in both the prescribed and free-living protocols. In contrast, the AG inclinometer was unable to accurately measure sitting time or transitions during either protocol. | NR/NR | High |
| 422 | Sadeh et al. (1994, United States) | Adults (N=20; 22.6±1.7 yrs; 55% females; NR) | Sleep (2 nights) | *Actigraph AMA-32* (wrist; NR; 1 minute; algorithm reported) | Biological state (Sleep/wake scores) | Polysomnography | Repeated measures ANOVA | Results obtained with the same algorithm for the dominant-wrist data were within the same range. Agreement for sleep scoring was consistently higher than for wake scoring. | N/NR | High |
| 423 | Sánchez-Trigo et al. (2020, Spain) | Premenopausal women (N=18; 44±5 yrs; 100% females; NR) | Jump and treadmill (12 minutes) | *Muvone* (hip, wrist; NR; NR; NR) | Intensity (Acceleration (g)) | Wearable (ActiGraph GT3X+ (hip, wrist)) | Pearson correlation; Dependent t-test; Bland-Altman analyses | The validity of Muvon to quantify acceleration has been tested at both the wrist and hip and could be used to assess mechanical loading during physical activities for the implementation of population-wide osteoporosis prevention programs. | N/N | High |
| 424 | Sandroff et al. (2012, United States) | Adults with multiple sclerosis (N=41; 47.2±9.1 yrs; 88.4% females; NR); Healthy adults (N=41; 46.5±10.0 yrs; 88.4% females; NR) | Treadmill (Approx. 45 minutes) | *ActiGraph 7164* (non-dominant hip; NR; 15 sec; NR) *ActiGraph GT3X* (non-dominant hip; NR; 15 sec; NR) | Intensity (Counts; Energy expenditure) | Indirect Calorimetry | Multiple correlation; Independent t-tests | The strong linear relationship between activity counts and energy expenditure and cut-points for quantifying time spent in MVPA should allow for better understanding of physical activity and examination of its predictors and consequences when using accelerometers in MS. | N/N | High |
| 425 | Sandroff et al. (2014, United States) | Adults with multiple sclerosis (N=54; 50.9±9.2 yrs; 83.3% females; NR) | Walking (18 minutes) | *ActiGraph GT3X+* (hip; ActiLife 6; NR; NR) | Intensity (Counts; Energy expenditure) | Indirect Calorimetry | Multiple correlation; Mixed-model ANOVA | We believe that this research will facilitate a better understanding of time spent in MVPA across a broad range of MS disability. | N/N | High |
| 426 | Santos-Lozano et al. (2013, Spain) | Adults (N=31; 47.1±3.5 yrs; 48.4% females; NR); Older adults (N=35; 71.9±5.4 yrs; 62.9% females; NR) | Treadmill (40 minutes) | *ActiGraph GT3X* (right hip; Actilife; 1 sec; NR) | Intensity (Counts; Energy expenditure) | Indirect Calorimetry | Repeated-measures ANOVA; Bland-Altman analyses | Activity counts derived from the VM yielded a more accurate EE estimation than those derived from the Y-axis. The GT3X represents a step forward in triaxial technology estimating EE. | N/N | High |
| 427 | Santos-Lozano et al. (2017, Spain) | Adults (N=23; 48.0±3.4 yrs; 56.5% females; NR) | Treadmill (30 minutes) | *SenseWear Armband* (upper arm; NR; NR; NR) | Intensity (Energy expenditure) | Indirect Calorimetry | Repeated-measures ANOVA; Bland-Altman analyses; Regression analyses; Sensitivity; Specificity | The SWA is not as precise in estimating EE as IC, but it could be a useful tool to determine levels of EE at low intensities. | N/N | High |
| 428 | Sargent et al. (2018, Australia) | Soccer players (N=12; 18.3±1.0 yrs; NR; NR) | Sleep (3 nights) | *Fitbit Charge HR* (non-dominant wrist; NR; NR; NR) | Biological State (Sleep time) | Polysomnography | Paired samples t-tests; Bland-Altmann analyses | Compared with polysomnography, the FitBit overestimated total sleep time by an average of 52 ± 152 min for night-time sleep periods, and by 4 ± 8 min for daytime naps. It is important for athletes and practitioners to be aware of the limitations of wearable devices that automatically detect sleep duration. | N/N | High |
| 429 | Sasaki et al. (2015, United States) | Adults (N=20; 24.1±4.5 yrs; 50% females; NR) | Treadmill and activities of daily life (Approx. 41 minutes) | *Fitbit* (waist; NR; NR; proprietary algorithm) | Intensity (Energy expenditure) | Indirect Calorimetry | Pearson correlation; Bland-Altman analyses; Paired t-tests | The Fitbit worn on the hip significantly underestimates EE of activities. The variability in underestimation of EE for the different activities may be problematic for weight loss management applications since accurate EE estimates are important for tracking/monitoring energy deficit. | NR/NR | High |
| 430 | Sasaki et al. (2016, United States) | Adults (N=35; 70.6±5.0 yrs; 60% females; NR) | Posture (1.5 minutes) and activities of daily life (35 minutes) | *ActiGraph GT3X+* (each wrist, hip; ActiLife 5; 20 sec; algorithms reported) | Posture/Activity Type (Standing, sedentary, locomotion, household, recreational) | Observation (Direct) | Agreement; Kappa statistics; Confusion matrix | Algorithms developed on free-living accelerometer data were more accurate in classifying the activity type in free-living older adults than those on our algorithms developed on laboratory accelerometer data. | N/N | High |
| 431 | Schaffer et al. (2017, United States) | Adults with stroke-induced hemiparesis (N=24; 54±13.4 yrs; 41.67% females; NR) | Indoor walking (6 minutes) | *Garmin Vivofit* (each wrist; NR; NR; NR); *Fitbit Zip* (waist; NR; NR; NR) | Intensity (Steps) | Observation (Video) | One-sample t-test; Bland–Altman analyses; Regression analysis; Mean absolute percent error; Independent sample t-tests; Pearson Chi-square tests; Pearson correlation | Fitbit Zip was more accurate and reliable for persons with stroke than Garmin Vivofit, but slower walking speeds were associated with greater undercounting of steps for both devices. The Fitbit Zip is appropriate for counting steps in adults poststroke who range from household to community ambulators. | N/N | High |
| 432 | Schneider et al. (2003, United States) | Adults (N=20; males: 34.7±12.6 yrs, females: 43.1±19.9 yrs; 50% females; NR) | Outdoor walking (400 m) | *Freestyle Pacer Pro*; *Kenz Lifecorder*; *New Lifestyles NL-2000*; *Omron HJ-105*; *Oregon Scientific PE316CA*; *Sportline 330 and 345*; *Walk4Life LS 2525*; *Yamax Skeletone EM-180*; *Yamax Digi-Walker SW-701* (all waist; NR; NR; NR) | Intensity (Steps) | Observation (Manually- counted) | Two-way repeated measures ANOVA; Paired t-tests; Bland-Altman analyses | Due to the variation that exists among models in regard to the internal mechanism and sensitivity, not all pedometers count steps accurately. Thus, it is important for researchers who use pedometers to assess physical activity to be aware of their accuracy and reliability. | N/NR | High |
| 433 | Schneller et al. (2015, Denmark) | Adults (N=14, 27.7±3.3 yrs; 57% females, NR) | Posture, treadmill, walking and ergometer (82 minutes) | *ActivPAL* (thigh; ActivPAL3 software; NR; NR), *Actiheart* (chest; Actiheart 4 software; 1 minute; branched equation model), *ActiGraph GT3X+* (hip, thigh, ActiLife 6; 10 sec; Crouter adult algorithm) | Intensity (Energy expenditure) | Indirect Calorimetry | Linear regression; Pearson’s coefficient of determination; Paired t tests; Mean square error; Bland-Altman analyses | Combining accelerometer data from a thigh-worn ActiGraph GT3X+ with activity type recognition improved the accuracy of activity specific EE estimation against indirect calorimetry in semi-standardized settings compared to previously validated methods using CPM only. | N/N | High |
| 434 | Scott et al. (2021, Australia) | Adults (N=20; 23.22±5.01 yrs; 70% fermales; NR) | Sleep (1 night) | *THIM* (finger; smartphone app v 1.0.1; 30 sec; sleep tracking algorithm); *Actiwatch-2* (wrist; Actiware Sleep software v6.0.0; 30 sec; default software algorithm); *Fitbit Flex* (wrist; smartphone app v3.3.1; 1 minute; proprietary Fitbit algorithm) | Biological State (Total sleep time) | Polysomnography | Sensitivity; Specificity; Accuracy; Linear Mixed Modelling; Bland-Altman analyses | Together, these studies suggest that THIM is capable of monitoring sleep and wake overnight in good and poor sleepers to a similar degree of accuracy as two of the most popular actigraphy devices available. | Y/N | High |
| 435 | Sears et al. (2017, United States) | Adults (N=10; 23.3±5.23 yrs; 50% females; NR) | Treadmill (25 minutes) | *Fitbit Charge HR* (wrist; NR; NR; NR); *Garmin Vivosmart HR* (wrist; NR; NR; NR); *Apple Watch* (wrist; NR; NR; NR); *Jawbone UP3* (wrist; NR; NR; NR); *Yamax* *Digi-Walker* (hip; NR; NR; NR) | Intensity (Steps) | Observation (Manually- counted) | Pearson correlation; One-way MANOVA | All wrist-worn devices tested tended to underestimate steps. These data indicate that wrist-worn pedometers are inaccurate even with a specific designed purpose: count steps in a controlled manner. Because these devices are inaccurate in this setting, they remain highly questionable for accuracy in a real-world setting in which the definition of a “step” becomes less finite. | NR/NR | High |
| 436 | Sellers et al. (2016, United Kingdom) | Adults (N=20; Median: 27.6 yrs, 55% females; NR) | Treadmill, outdoor walking and activities of daily life (Approx. 80 minutes) | *ActivPAL3* (thigh; activPAL software version 7.1.18; NR; proprietary analysis algorithms) | Posture/Activity Type (Sitting, lying, upright); Intensity (Steps) | Observation (Video) | Modified Bland-Altman analyses; Agreement; Sensitivity; Positive predictive value | In general, the detection of posture and purposeful stepping with the activPAL3 was excellent indicating that it is a suitable monitor for characterising free-living posture and purposeful stepping activity in healthy adults. | Y/N | Some/Some |
| 437 | Serra et al. (2017, United States) | Adults with chronic hemiparetic gait (N=28; 60.4±1.6 yrs; 35.71% females; 43% Caucasian, 56% African American) | Treadmill (6 minutes) and activites of daily life (40 minutes) | *Actical* (non-paretic hip; NR; 1 minute; NR) | Intensity (Activity counts, Energy expenditure) | Indirect Calorimetry | Regression analysis | Our revised cut-points better reflect activity levels after stroke and suggest significantly lower thresholds relative to those observed for the general population of healthy individuals. We conclude that the standard, commonly applied Actical thresholds are inappropriate for this unique population. | N/N | High |
| 438 | Sharif & BaHammam (2013, Saudi Arabia) | Adults with obstructive sleep apnea (Patients: N=107; 45.2±14.3 yrs; 38.32% females; NR; Control: N=30; 42.6±14.0 yrs; 43.33% females; NR) | Sleep (1 night) | *SenseWear* (right arm; Body Media InnerView Research Software v5.1; 1 minute; proprietary algorithms) | Biological State (Total sleep time) | Polysomnography | Paired sample t‑test; Wilcoxon rank test; Chi‑squared (χ2) test; Paired sample correlation; Bland‑Altman analyses; Intraclass correlation coefficients | The current data suggest that BSA is a reliable method for determining sleep in patients with OSA when compared against the gold standard test (PSG). BSA can be a useful tool in determining sleep in patients with OSA and can be combined with portable sleep studies to determine TST. | N/N | Some |
| 439 | Shcherbina et al. (2017, United States) | Adults (N=60; males: 40±11.48 yrs, females: 37±9.77 yrs; 51.67% females; NR) | Treadmill and cycle ergometer (35 minutes) | *Apple Watch* (wrist; Apple Health app; NR; NR); *Basis Peak* (wrist; Basis app; NR; NR); *Fitbit Surge* (wrist; Fitbit Developer API; NR; NR); *Microsoft Band* (wrist; mitmproxy software; NR; NR); *PulseOn* (wrist; SQLite3 database; NR; NR) | Intensity (Energy expenditure) | Indirect Calorimetry | Two-way ANOVA; Pearson correlation; General estimating equation; Root mean square error; Bland-Altman analyses | In conclusion, most wrist-worn devices adequately measure HR in laboratory-based activities, but poorly estimate EE, suggesting caution in the use of EE measurements as part of health improvement programs. | N/N | High |
| 440 | Shepherd et al. (1999, United States) | Adults (N=29; 42.3±15.3 yrs; 72.41% females; NR) | Outdoor walking (400m) | *Stepwatch* (ankle; NR; NR; NR) | Intensity (Steps) | Wearable (Sportline (hip)); Observation (Manually- counted) | Percentage error; Student's t test; Univariate regression analysis; Pearson correlation | The accuracy and additional capabilities, including a real-time memory record of activity, of the step activity monitor make it well suited for objectively quantifying ambulatory activity, especially for obese subjects. | N/NR | High |
| 441 | Shimizu et al. (2018, Japan) | Adults (N=10; 27.6±5.6 yrs; 50% females; NR); Stroke patients (N=10; 57.5±16.2 yrs; 50% females; NR) | Walking (6 minutes) | *Omron Active Style Pro HJA350-IT* (waist; HMS-HJA-IC01 J; 10 sec; NR) | Intensity (Energy expenditure) | MET-values (Compendium of physical activities by ACSM) | One sample t-test | The estimated metabolic equivalents using accelerometer may be suitable to assess movement activity rather than motionless activity, and accelerometer demonstrated acceptable validity in people with subacute stroke. | N/N | High |
| 442 | Shin et al. (2015, Australia) | Adults (N=9; 23.3±4.1 yrs; 33.33% females; NR) | Sleep (9 nights) | *Actiwatch 2* (non-dominant wirst; Respironics Actiware v5.59.0015; 30 sec; NR); *Sense-Wear Pro3* (non-dominant arm; SenseWear professional 7.0 software; NR; proprietary algorithms) | Biological State (Total sleep time) | Polysomnography | Bland–Altman analyses; Linear regression; Kappa statistic; Agreement Sensitivity; Specificity | Actiwatch2 showed small biases for most of sleep variables at all temperature conditions, except for WASO. Sense-Wear armband Pro3 is reliable for measures of TST, WASO and SE at 17–22 °C but not at 29 °C, and SOL approximates that of PSG only at 29 °C, thus caution is needed when monitoring sleep at different temperatures, especially in home sleep studies, in which temperature conditions are more variable. | N/N | High |
| 443 | Simonsen et al. (2020, Denmark) | Adults (N=30; 28.2±4.33 yrs; 13.3% females; NR) | Treadmill (9 minutes) | *Polar M200* (left arm; NR; NR; NR); *Polar A300* (left arm; NR; NR; NR); *Dunlop pedometer* (right hip; NR; NR; NR) | Intensity (Steps) | Observation (Video) | Relative error; Spearman correlation; Bland-Altman analyses | The results of this study show that step counting validity and error obtained during treadmill walking is not similar to a field test. | N/N | High |
| 444 | Simpson et al. (2015, Canada) | Adults (N=42; 73±6.9 yrs; 73.81% females; NR) | Indoor walking (Approx. 120 meters) | *Fitbit One* (waist, ankle; NR; NR; proprietary algorithms) | Intensity (Steps) | Observation (Video) | Percentage error; Bland-Altman analyses; Paired t-tests | The Fitbit One can accurately capture steps at slow speeds when placed at the ankle and thus may be appropriate for capturing physical activity in slow-walking older adults. | N/N | High |
| 445 | Sirichana et al. (2017, United States) | Adults (N=20; 21±1 yrs; 50% females; NR) | Activities of daily life and treadmill (76 minutes) | *GENEActive* (each wrist; NR; 1 minute; Sum of vector magnitudes with gravity subtracted (SVMgs)) | Intensity (Counts) | Indirect Calorimetry | Linear regression | Wrist-worn triaxial accelerometry reliably predicted energy expenditure during common physical activities <6 METs. More consistent correlations were found when the accelerometer was worn on the non-dominant wrist rather than the dominant wrist. | N/NR | High |
| 446 | Sivertsen et al. (2006, Norway) | Adults with chronic primary insomnia (N=34; 60.5±4.5 yrs; 50% females; NR) | Sleep (1 night) | *Actiwatch Plus* (wrist; Actiwatch Sleep Analysis 2001 software v1.19; 30 sec; NR) | Biological State (Total sleep time) | Polysomnography | Agreement; Sensitivity; Specificity; Pearson correlations; Wilcoxon rank tests | The present findings suggest that the clinical utility of actigraphy is still suboptimal in older adults treated for chronic primary insomnia and should, hence, be used in this clinical setting with the concurrent use of supplementary assessment methods. | NR/NR | High |
| 447 | Sjöberg et al. (2021, Sweden) | Adults with chronic pain (N=42; 43.8±11.8 yrs; 76.19% females; NR) | Treadmill (18 minutes) | *Fitbit Versa* (wrist; NR; NR; NR) | Intensity (Energy expenditure; Steps) | Indirect Calorimetry; Wearable (ActiGraph GT3X (hip)) | Intraclass correlation; ANOVA; Spearman correlation; Bland-Altman analyses; Mean absolute percentage error | The wrist-worn device systematically overestimated energy expenditure and showed poor agreement and correlation compared to the criterion standard (Jaeger Oxycon Pro) and the relative criterion standard (ActiGraph GT3X), which needs to be considered when used clinically. | N/N | High |
| 448 | Skipworth et al. (2011, Norway, Switzerland, Germany) | Adults with advanced cancer (N=45; 64.8± 12.5 yrs; 51.1% females; NR) | Activities of daily life (30 minutes) | *ActivPAL* (thigh; NR; NR; NR) | Intensity (Energy expenditure; Steps) | Observation (Video) | Student’s Independent; Sample t-test; Bland-Altman analyses; Pearson correlation | AM-systems provide valid estimates of body positions and transfers, but not step count, especially in non-self caring patients. ActivPAL can derive estimates of EE but there is considerable variability in results, which is consistent, in part, with the inaccuracy in step count. | N/N | Some |
| 449 | Skotte et al. (2014, Denmark) | Adults (N=17; 34±11 yrs; 58.8% females; NR) | Activities of daily life (30 minutes) | *ActiGraph GT3X+* (hip, thigh; ActiLife v5.5; NR; NR) | Posture/Activity Type (Move, sit, stand, walk, run, cycle) | Observation (Direct) | Sensitivity; Specificity | The developed method for detecting physical activity types showed a high sensitivity and specificity for sitting, standing, walking, running, walking stairs, and cycling in a standardized setting and for sitting posture during free living. | N/NR | High |
| 450 | Slater et al. (2014, Australia) | Adults (N=108; 22.7±0.2 yrs; 47.22% females; NR) | Sleep (1 night) | *ActiGraph GT3X+* (non-dominant wrist, hip; ActiLife 6.8; 1 minute; Sadeh’s algorithm) | Biological State (Total sleep time) | Polysomnography | Sensitivity; Specificity; Accuracy; Repeated measures ANOVA; Bland-Altman analyses; Intraclass correlation coefficients; Paired t-tests | This study showed that using existing algorithms, a GTX3+ Actigraph worn on the hip does not provide valid or accurate measures of sleep, mainly due to poor wake detection. Relative to the hip, a wrist worn GTX3+ Actigraph provided more valid measures of sleep, but with only moderate capability to detect periods of wake during the sleep period. | N/N | Some |
| 451 | Slootmaker et al. (2009, Netherlands) | Adults (N=32; 29.4±7.3 yrs; 56.25% females; NR) | Treadmill and walking (15 minutes) | *Personal Activity Monitor* (waist; NR; 1 sec; regression equations); *MTI Actigraph* (waist; NR; 1 sec; regression equations) | Intensity (Energy expenditure) | Indirect Calorimetry | Generalized estimating equations; Intraclass correlation coefficients; Paired t-test; One-way ANOVA; Coefficient of variation | The PAM and Actigraph accelerometer are comparable in assessing bodily movement during treadmill and stair walking. The PAM is a valid device to rank subjects in EE and can be useful in collecting objective data to monitor habitual physical activity. | N/N | High |
| 452 | Smith & Schroeder (2008, United States) | Adults (N=96; 22.4±1.8 yrs; 51% females; NR) | Walking, skipping, galloping, sliding, hopping (10 minutes) | *Walk4Life LS-7010* (right and left thigh; NR; NR; NR) | Intensity (Steps) | Observation (Manually- counted) | ANOVA; Dependent t-tests; Chronbach’s Alpha; Intraclass correlation coefficients; Bland-Altman analyses | The pedometer may not consistently register the vertical force produced by the trail foot contact, the lead foot contact, or a combination of the two while skipping, galloping, and sliding. | N/NR | High |
| 453 | Smith et al (2012, United States) | Pregnant women (N=30; 29±4.3 yrs; 100% females; 93% Caucasian) | Treadmill and activities of daily life (Approx. 28 minutes) | *SenseWear Mini* (left upper arm; SenseWear Software v7.0, v2.2; 1 minute; proprietary algorithms) | Intensity (Energy expenditure) | Indirect Calorimetry | Three-way mixed model ANOVA; Pearson correlation; Bland–Altman analyses | Overall, the SWA correlated well with IC; however, EE was significantly overestimated during most activities. | N/N | Some |
| 454 | Smith et al. (2017, United States) | Adults (N=19; 18-22 yrs; NR; NR) | Indoor walking (1.609 meters) | *Omron HJ 720-ITC* (right hip, right front pocket, middle of the chest, middle of the back; NR; NR; NR) | Intensity (Steps; Energy expenditure) | Indirect Calorimetry; Observation (Manually-counted) | Intraclass correlation coefficient; Standard error of the measurement; One-way ANOVAs; Accuracy | Although the Omron pedometer produced reliable results regardless of placement on the body, it did not produce valid estimates of kcal expenditure. | N/N | High |
| 455 | Smith et al. (2019, United States) | Adults with lower-limb prosthetics (N=32; 49.7±14.0 yrs; 34.38% females; NR) | Indoor walking (140 meters) | *Polar Loop*, *Fitbit Flex*, *MOVEBAND*, *Garmin Vivofit*, *Fitbit Charge* (all right and left wrists; NR; NR; NR); *Omron HJ-113* (right and left hip; NR; NR; NR) | Intensity (Steps) | Observation (Manually- counted) | Repeated-measures ANOVA; Intraclass correlation coefficients; Percentage error; Bland–Altman analyses; Pearson correlation | When considering the use of consumer-grade wrist-worn activity monitors for assessing step counts in persons using lower-limb prostheses, the Garmin Vivofit seems to be the best option followed by Fitbit Charge. | N/N | High |
| 456 | Soric et al (2012, Croatia) | Adults (N=19; 28±6 yrs; 58% females; NR) | Inline skating (6.360 meters) | *Sensewear* 3 (upper right arm; SenseWear Professional software v7.0; 1 minute; proprietary algorithms) | Intensity (Energy expenditure) | Indirect Calorimetry | Paired t-tests; Simple linear regression; Bland–Altman analyses | The results of the present study indicate that the SWA is not able to overcome the drawbacks of accelerometry in assessing activities with limited vertical movement. | N/N | High |
| 457 | Spielmanns et al. (2019, Germany) | Adults (N=26; 54.8±14.4 yrs; 35% females; NR) | Sleep (2 nights) | *Polar A300* (wrist; NR; NR; NR) | Biological State (Total sleep time) | Polysomnography | Bland-Altman analyses; Spearman correlation | The sleep efficiency and TST measured with the PAM sufficiently reflect the PSG sleep parameters and the subjects’ subjective feelings. | N/N | High |
| 458 | Stanish (2004, Canada) | Adults with mild mental retardation (N=20; 19-65 yrs; 60% females; NR) | Walking (1.6 kilommeters) | *Yamax Digiwalker SW 500* (right and left waist; NR; NR; NR) | Intensity (Steps) | Observation (Manually- counted) | Intraclass correlation coefficients; Independent samples t-test; 2 x 2 ANOVAs | Pedometer counts were highly consistent with actual step counts during normal and fast paced walking on two ground surfaces. | NR/NR | High |
| 459 | Stansfield et al. (2015, United Kingdom) | Adults (N=20; 36±10 yrs; 50% females; NR) | Treadmill (25 minutes) | *ActivPAL3* (right and left thigh; activPAL3 software v 7.1.18; NR; NR) | Intensity (Steps) | Observation (Video) | Percentage steps detection; Wilcoxon Signed Rank Test; Paired sample correlations | When examining the stepping activity of groups with limited stepping cadence the above thresholds of performance should be considered to ensure that outcomes are not misinterpreted and important very slow stepping activity missed. | N/N | High |
| 460 | Steeves et al. (2011, United States) | Adults (N=80; Study 1: 35±12.8 yrs; 48% females; NR; Study 2: 22±3.15 yrs; 50% females; NR) | Treadmill and activities of daily life (NR) | *Omron HJ-303* (pants pocket, waist, backpack; NR; NR; NR); *Sportline Traq* (pants pocket; NR; NR; NR); *Yamax Digi-Walker SW200* (waist; NR; NR; NR) | Intensity (Steps) | Observation (Manually-counted) | Accuracy; Repeated-measures ANOVA | The Omron HJ-303, worn on the waist, appeared to be the most valid of the 3 pedometers. | Y/NR | High |
| 461 | Stenbäck et al. (2021, Finland) | Adults (N=19; 33.5±8.3 yrs; 36.8% females; NR) | Treadmill (20 minutes) | *ActiGraph GT3X+* (hip; ActiLife v6.13.4; 1 s; Freedson equation); *Sartorio Xelometer* (hip; Sartorio v18 software; NR; detection algorithms); *ActivPAL* (left thigh; PALconnect v8.10.8.76, PALanalysis v8.11.2.54; equation reported) | Intensity (Steps; Energy expenditure) | Observation (Video); Indirect Calorimetry | Mean absolute percentage error; Bland-Altman analyses; | The Xelometer is a valid device for assessing step counts at different gait speeds. MAPE is different at different speeds, which is of importance when assessing the PA in obese subjects and elderly. EE estimates of all three devices were found to be inaccurate when compared with indirect calorimetry. | N/Y | High |
| 462 | Stewart et al. (2018, New Zealand) | Adults (N=33; 42.3±9.9 yrs; 51.5% females; NR) | Activities of daily life and treadmill (1 hour) | *Axivity AX3* (thigh, lower back; NR; NR; NR) | Posture/Activity Type (Sitting, lying, standing, walking, running) | Observation (Video) | Sensitivity, Specificity, Accuracy | When previous wear time compliance results are taken together with our findings, it represents a promising step forward for monitoring and understanding 24-h time-use behaviors. | N/N | Low |
| 463 | Storm et al. (2015; United Kingdom | Adults (N=16; 28.87±2.65 yrs; 38% females; NR) | Walking and activities of daily life (20 minutes) | *DynaPort Movemonitor* (lower back; Dyrector v1.0.7.17; 1 sec; NR); *Jawbone Up* (right wrist; UP v2.8.8.3.7.1; 1 minute; NR); *Fitbit One* (left waist; Connect v1.0.0.4022; 1 minute; NR); *ActivPAL* (right shank; ActivPAL v7.1.18; 1 sec; NR); *Nike+ Fuelband* (left wrist; Nike+ Connect v3.8; 1 minute; NR); *Tractivity* (right ankle; Connect v2.12; 1 minute; NR); *Sensewear Armband Mini* (upper left arm; Sensewear v7.0.0.2378; 1 minute; NR) | Intensity (Steps); Posture/Activity Type (Lying, sitting, standing, locomotion, shuffling) | Wireless inertial measurement units | Mean absolute percentage error; mixed-model ANOVA; Bland-Altman analyses | The Movemonitor, One, ActivPAL, Nike+ Fuelband and Sensewear Armband Mini underestimated the number of steps in all the observed walking speeds, whereas the Tractivity significantly overestimated step count. The Movemonitor was the best performing sensor, with an error lower than 2% at all speeds and the smallest error obtained in the outdoor walking. The activity recognition protocol showed that the Movemonitor performed best in the walking recognition, but had difficulty in discriminating between standing and sitting. | N/N | High/High |
| 464 | Storti et al. (2008, United States) | Adults (N=34, 79.2±6.0 yrs; 38% females; NR) | Walking (100 step walking test) | *ActiGraph* (hip; NR; 3 sec; NR), *StepWatch* (ankle; NR; 3 sec; NR), *Yamax DigiWalker* (dominant hip; NR; NR; NR) | Intensity (Steps) | Observation (Manually-counted) | ANOVA; Chi-square tests; Spearman correlations; One-way ANOVA | All three objective activity monitors performed well at moderate and higher walking speeds, but at decreased gait speeds, the SAM seemed to be the most accurate. | N/NR | High |
| 465 | Strath et al. (2001, United States) | Adults (N=30; 32.5±12.7; 46.7% females; 80% Caucasian, 17% African American, 3% Hispanic) | Activities of daily life and treadmill (60 minutes) | *Polar Vantage XL* (wrist; NR; NR; NR); *CSA* (dominant wrist, hip, right thigh; NR; 1 minute; NR); *Yamax SW-701* (hip; NR; NR; NR) | Intensity (Energy expenditure (MET)) | Indirect Calorimetry | Error score; Bland-Altman analyses; Linear regression | The simultaneous HR-motion sensor technique is a good predictor of EE during selected lifestyle activities, and allows researchers to more accurately quantify free-living PA. | N/NR | High |
| 466 | Strath et al. (2015, United States) | Adults (N=99; 49.3±17.4 yrs; 52% females; NR) | Treadmill and activities of daily life (78 minutes) | *ActiGraph GT3X* (non-dominant hip, non-dominant wrist, ankle; NR; 1 sec; Ngram time series model) | Intensity (Energy expenditure (MET)); Posture/Activity Type (Slow walking, moderate walking, fast walking) | Indirect Calorimetry | Jackknife leave-one-out cross validation; Bias; Root mean squared error; Accuracy | Specific age group models on average performed better than when all age groups were combined. A time series computation show promising results for predicting energy cost and activity type. | N/N | High/High |
| 467 | Sugino et al. (2011, Japan) | Adults with COPD (N=14, 74.3**±**6.2 yrs, 0% females, NR) | Walking and cycling (NR) | *Actimarker* (waist; NR; NR; NR) | Intensity (Energy expenditure) | Wearable (DynaPort Activity Monitor (waist, leg)) | Regression analyses; Wilcoxon test; Intraclass correlation coefficient; Bland-Altman analyses | The validity of the Actimarker was confirmed, and repeatability was obtained when the data from at least 3 non-rainy weekdays were analyzed. Actimarker appears to be useful as a simplified method to evaluate the physical activity of COPD patients. | NR/NR | High |
| 468 | Sushames et al. (2016, Australia) | Adults (N=25; 23.7±5.8 yrs; 48% females; NR) | Treadmill (24 minutes) | *Fitbit Flex* (wrist; NR; 1 minute; NR); *ActiGraph GT3X+* (hip; ActiLife v6.2; 1 minute; Freedson equation) | Intensity (Steps) | Observation (Video) | Coefficients of variation; Absolute differences and absolute proportional differences; Paired samples t-tests; Intraclass correlation coefficients; Bland-Altman analyses | The Fitbit Flex has moderate validity for measuring physical activity relative to direct observation and the Actigraph. | N/N | High |
| 469 | Svarre et al. (2020, Denmark) | Adults (N=30; 26.6±6.2 yrs; 60% females; NR) | Treadmill (40 minutes) | *Garmin Vivosmart HR* (wrist; Garmin Express v. 4.1.19.0 software; NR; NR); *StepWatch 3* (each ankle; StepWatch v.3.4; NR; NR) | Intensity (Steps) | Observation (Video) | Bland–Altman analyses; Mean absolute percentage error | Garmin Vivosmart HR tended to undercount steps compared with the manual step count, and StepWatch 3 slightly overcounted steps compared with the manual step count. Both the consumer-graded activity tracker (Garmin Vivosmart HR) and the research-graded (StepWatch 3) are valid in detecting steps at selected walking speeds in healthy adults under controlled conditions. | N/N | High |
| 470 | Swan et al. (1997, United States) | Adults (N=31; 22.6±0.8 yrs; NR; NR) | Treadmill (40 minutes) | *Caltrac* (hip; NR; NR; NR) | Intensity (Energy expenditure) | Indirect Calorimetry | ANOVA; Dependent t-tests; Pearson correlation; Linear Regression | The Caltrac is a reliable instrument but it did not accurately distinguish EE in running, race walking, or stepping in a group of young women. | N/NR | High |
| 471 | Swartz et al. (2003, United States) | Adults (N=66; 28.6±10 yrs; 47% females; NR) | Treadmill (9 minutes) | *Yamax SW-200* (right waist, thigh; NR; NR; NR) | Intensity (Steps) | Observation (Manually- counted) | Repeated measures ANOVA | The placement of the pedometer on the front, side, or back of the waistband did not affect accuracy of the pedometer for counting steps. | NR/NR | High |
| 472 | Swartz et al. (2009, United States) | Adults (N=48; 33.0±10.7 yrs; 58.3% females; NR) | Treadmill (30 minutes) | *Kenz Lifecorder EX* (hip; NR); NR; NR; *Omron HJ-700IT* (hip; NR; NR; NR); *Sportbrain iStep X1* (hip; NR; NR; NR) | Intensity (Energy expenditure) | Indirect Calorimetry | ANOVA | Study demonstrates that these devices do not offer the accuracy needed to provide precise feedback on EE for individuals with varying BMI levels. | N/NR | High |
| 473 | Syed et al. (2020, Canada) | Adults with chronic obstructive pulmonary disease (N=9; 72±16 yrs; 44% females; NR) | Sleep (1 night) | *Vibe Actigraph* (each wrist; NR; NR; proprietary algorithms) | Biological State (Total sleep time) | Polysomnography | Spearman correlation; Agreement; Cohen´s kappa; Repeated measures ANOVA; Sensitivity; Specificity; Bland-Altman analyses | This study demonstrated that, under controlled laboratory conditions, the Vibe actigraph in its default settings is a promising tool for the detection of sleep-wake parameters in a small number of ambulatory patients with COPD. | Y/Y | High |
| 474 | Taibi et al. (2013, United States) | Adults with insomnia (N=16; 69.4±8.1 yrs; 100% females; White/Caucasian: 94%) | Sleep (9 nights) | *Actiwatch-64* (wrist, Actiware 5.57 software; 30 sec; scoring algorithm (low, medium, high)) | Biological state (Total sleep time) | Polysomnography | Wilcoxon signed-ranks test; Generalized linear model analysis; Sensitivity; Specificity; Accuracy; Predictive values; Bland-Altman analyses | Actigraphy offers a relatively inexpensive and unobtrusive method for measuring sleep, but it appears to underestimate sleep disturbance, particularly at sleep efficiency levels below 73%, in older women with insomnia. | N/N | Some |
| 475 | Takacs et al. (2013, Canada) | Adults (N=30; 29.6±5.7 yrs; 50% females; NR) | Treadmill (25 minutes) | *Fitbit One* (two at hips pocket; NR; NR; proprietary algorithms) | Intensity (Steps) | Observation (Video) | Two-way repeated measures ANOVA, Concordance correlation coefficients; Bland-Altman analyses; Intraclass correlation coefficients | The Fitbit One activity monitors are valid and reliable devices for measuring step counts in healthy young adults. The distance output of the monitors is inaccurate and should be noted with caution. | N/NR | High |
| 476 | Tam & Cheung (2018, Hong-Kong) | Adults (N=30; 32.1±8.66 yrs; 50% females; NR) | Treadmill (30 minutes) | *Fitbit Charge HR* (dominant wrist; NR; NR; proprietary algorithm); *Mi Band 2* (dominant wrist; NR; NR; proprietary algorithm) | Intensity (Steps) | Observation (Video) | Percent relative error; Paired sample t test; Pearson correlation; Bland-Altman analyses | Both Fitbit Charge HR and Mi Band 2 provided accurate step count measurement in the treadmill walking test. | N/N | High |
| 477 | Taraldsen et al. (2011, Norway) | Adults with impairment function ((Test group: N=36; 79.7±7.3 yrs; 61% females; NR) Reference group: N=10; 46.3±9.0 yrs; 100% females; NR) | Activities of daily life (20-60 minutes) | *ActivPAL* (each thigh, chest; activPAL Professional Research Edition; NR; proprietary algorithm) | Posture/Activity Type (Sitting, lying, upright); Intensity (Steps) | Observation (Video) | Bland Altman analyses; Absolute percent error | The activPAL sensor system provides valid measures of postures and transitions in older people with impaired walking ability. Step counting needs to be improved for the sensor system to be acceptable for this population, especially at slow walking speeds. | N/NR | Some/Some |
| 478 | Taylor et al. (2014, New Zealand) | Adults (N=22; 88.1±5 yrs; 82% females; NR) | Activities of daily life (21 minutes) | *DynaPort MoveMonitor* (waist; NR; NR; NR) | Posture/Activity Type (Sitting, lying, standing, locomotion) | Observation (Video) | Bland-Altman analyses; Sensitivity; Specificity; Percentage overall agreement | This single-device accelerometer provides a valid measure of lying and locomotion in people aged >80 years. There is an error of approximately 25% when discriminating sitting from standing postures, which needs to be taken into account when monitoring longer-term habitual activity in this age group. | N/N | Some |
| 479 | Taylor et al. (2018, United States) | Female Adults (N=16; 18.9±1.1 yrs.; 19. ±1.2 yrs.; 100% females, NR) | Walking and Running (20m Shuttle Run); Basketball training (30 minutes) | *SenseWear Mini Armband* (wrist, BodyMedia's InnerView® Research Software v7.0; NR; proprietary algorithm) | Intensity (Energy expenditure) | Indirect Calorimetry | Two-way ANOVA, t-test, Pearson correlation | Due to the underestimation of EE by the Mini, the development of exercise specific algorithms to improve the estimation of EE during intermittent exercise in basketball players is warranted. | NR/NR | High |
| 480 | Te Lindert et al. (2020, Netherlands) | Adults with insomnia disorder (N=58; 47.8±14.0 yrs; 75.9% females; NR); Adults without sleep difficulties (N=56; 69.6% females; 43.2±15.0 yrs; NR) | Sleep (2 nights) | *GENEActiv* (wrist; GENEActiv PC 1.0; 30 sec; immobile–mobile algorithm) | Biological state (Total sleep time) | Polysomnography | Bland-Altman analyses; Accuracy, Sensitivity, Specificity, Positive predictive value; Negative predictive value; Kappa statistics | This systematic evaluation shows that actigraphic sleep feature estimation can be improved by using uncommon parameter settings. One specific parameter setting provides (near-)optimal estimation of sleep onset and nocturnal sleep across ID and controls | N/N | Some |
| 481 | Tedesco et al. (2019, Irland) | Adults (N=18; 69.35±2.8 yrs; 61% females; NR) | Treadmill (9 minutes); Activities of daily life (28 minutes) | *Fitbit Charge 2* (wrist; NR; NR; NR); *Garmin VivoSmart HR+* (wrist; NR; NR; NR); *Philips Health Watch* (wrist; NR; NR; NR); *Withings Pulse Ox* (wrist; NR; NR; NR); *ActiGraph GT9X-BT* (ankle, hip, ActiLife software; NR; publicly available validated algorithm); *Omron HJ-720ITC* (hip; NR; NR; NR); *Polar H7* (chest; NR; NR; NR) | Intensity (Steps) | Observation (Video) | Mean Absolute Percentage Error; Intraclass correlation; Bland-Altman analyses; Wilcoxon signed-rank test; T-test | This study showed a number of limitations of consumer-level wrist-based activity trackers for older adults. Therefore, caution is required when used, in healthcare or in research settings, to measure activity in older adults. | N/N | High |
| 482 | Thiebaud et al. (2018, United States) | Adults (N=22; 22±3 yrs; 9% females; NR) | Treadmill (15 minutes) | *TomTom Cardio* (wrist; NR; NR; NR); *Microsoft Band* (wrist; NR; NR; NR); *Fitbit Surge* (wrist; NR; NR; NR) | Intensity (Energy expenditure) | Indirect Calorimetry | Pearson correlations; Spearman rank correlation; Mean percentage error | Data from these devices may be useful in obtaining an estimate of heart rate for everyday activities and general exercise, but energy expenditure from these devices may be significantly over- or underestimated. | N/N | High |
| 483 | Thompson et al. (2006, United Kingdom) | Adults (N=20, 25±5 yrs, 50% females, NR) | Activities of daily life (104 minutes) | *Actiheart* (chest, NR; NR equation provided by the manufacture), *Yamax DW 351* (right hip, NR; NR; NR) | Intensity (Energy expenditure) | Indirect Calorimetry | Bland-Altman analyses; Agreement analysis | AHR provides an accurate estimate of criterion energy expenditure whereas a simple motion sensor (pedometer) does not. | NR/NR | High |
| 484 | Thorup et al. (2017, Denmark) | Adults (N=20; 39±13.79 yrs; 50% females; NR) | Treadmill (30 minutes) | *Fitbit Zip* (two at chest, two at waist; NR; NR; NR) | Intensity (Steps) | Wearable (Shimmer3 (ankle)) | Relative error; Interclass correlation coefficient | A speed of 3.6 km/hour or higher is required to expect acceptable accuracy in step measurement using a Zip, on a treadmill and in real life. Inaccuracies are directly related to slow speeds, which might be a problem for patients with cardiac disease who walk at a slow pace. | N/N | High |
| 485 | Tierney et al. (2013, Ireland) | Adults with Rheumatoid Arthritis (N=14; 64.43±6.80 yrs; 43% females; NR) | Activities of daily life (115 minutes) | *SenseWear Armband* (right upper arm; Research Software v6.1; NR; proprietary algorithm) | Intensity (Energy expenditure; Steps) | Indirect Calorimetry; Observation (Video) | Bland-Altman analyses; Intraclass correlation coefficient; Pearson correlation | The SWA can be considered a valid tool to estimate energy expenditure during ADL in the RA population; however, attention should be paid to its tendency to overestimate energy expenditure. | NR/NR | High |
| 486 | Tophoi et al (2018, Denmark) | Adults (N=20; 25.6±2 yrs; 50% females; NR) | Treadmill (8 minutes) | *Fitbit Surge* (nondominant wrist; Fitbit for iOS and android; NR; NR); *Fitbit Charge HR* (nondominant wrist; Fitbit for iOS and android; NR; NR); *Microsoft Band 2* (nondominant wrist; Microsoft Health v 1.3.21021.1; NR; NR); *A&D 101NFC Activity Monitor* (hip; NR; NR; NR) | Intensity (Steps) | Observation (Manually- counted) | Mean average percentage error; Bland–Altman analyses | The four evaluated activity trackers are not useful for patient groups walking at lower speeds during rehabilitation, nor for counting indoor walking. | N/N | High |
| 487 | Toth et al. (2017, United States) | Adults (N=25; 26±8 yrs; NR, NR) | Treadmill (20 minutes); Activities of daily life (NR) | *StepWatch 3* (two at ankles; NR; NR; NR) | Intensitiy (Steps) | Observation (Manually- counted) | Error scores; One-way repeated measures ANOVA; One sample t-tests | The preprogrammed “quick start” StepWatch settings should be used with individuals who do not engage in running and vigorous sports. However, for individuals who engage in running and tennis, use of modified settings may result in improved step counting accuracy. | N/N | High |
| 488 | Treacy et al. (2017, Australia) | Rehabilitation inpatients (N=166; 80±11 yrs; 45% females; NR) | Walking (6 minutes) | *Garmin Vivofit* (wrist, NR; NR; NR); *Fitbit Charge* (wrist; NR; NR; NR); *Fitbit One* (hip, ankle; NR; NR; NR); *G-Sensor 2026 Accelerometer Pedometer* (hip; NR; NR; NR); *ActiGraph GT3X+* (hip, ActiLife 6 software; 1 sec; NR); *ActivPAL*(thigh, ActivPAL v7.2.32 software; NR; NR); *StepWatch Activity Monitor* (ankle; Modus Trex software; 3 sec; NR) | Intensity (Steps) | Observation (Manually- counted) | Intraclass correlation coefficient; Percentage agreement; Average absolute error | The StepWatch showed the highest accuracy and closest agreement with observed step count. This device can be confidently used by researchers for accurate measurement of step counts in inpatient rehabilitation in individuals who walk slowly. | N/N | High |
| 489 | Tripette et al. (2014, Japan) | Adults (N=22; 19.7*±*1.8 yrs; 45% females; NR) | Active video games (NR) | *Omron Activity Style Pro HJA-350IT* (waist; NR; 10 sec; equation reported) | Intensity (Energy expenditure; MET-values) | Indirect Calorimetry | Paired t-test; Two-way repeated measure ANOVA; Bland-Altman analyses | Results point out the potenial bias of accelerometry measurements for evaluating AVG intensities. Beacause average AVG intensity lays at the boundary between LPA and MVPA classes, misclassifications can frequently occur. Accelerometry data should be interpreted with caution in intervention studies using AVG. | N/N | Some |
| 490 | Tsang et al. (2015, United States) | Adults with spinal cord injury (N=45, 41.0±12.6 yrs, 13% females, NR) | Activities of daily life (120 minutes) | *SenseWear* (right triceps; NR; NR; NR) | Intensity (Energy expenditure) | Indirect Calorimetry | Mean signed percent error, mean absolute percent error, intraclass correlation coefficient, Bland-Altman analyses | The custom models for the SenseWear armband significantly improved the EE estimation accuracy for MWUs with SCI. | N/N | Some |
| 491 | Tucker et al. (2015, United States) | Adults (N=24; 28.4±7.8 yrs; 54% females; NR) | Activities of daily life (120 minutes) | *Nike + Fuelband* (left wrist; Nike + Software; 1 minute; proprietary algorithm); *SenseWear armband* (left upper arm; SenseWear Professional Software 7.0; 1 minute; proprietary algorithm) | Intensity (Physical activity energy expenditure) | Indirect Calorimetry | Intraclass correlation coefficient; Bland-Altman analyses; Mean absolute percent error; Pearson correlations; Paired T-test | The Nike + Fuelband provided valid and reliable estimates of PAEE, that are similar to the previously validated SWA, during a routine that included approximately equal amounts of sedentary/light-, moderate- and vigorous-intensity physical activity. | N/Y | Some |
| 492 | Tudor Locke et al. (2015, United States) | Adults (N=15; 27.5±2.5 yrs; 67% females; NR) | Treadmill protocol (3 hrs) | *ActiGraph GT3X+* (right hip, nondominant wrist; ActiLife software v6.0; 1 minute; proprietary algorithm) | Intensity (Steps) | Observation (Video) | Paired sample t-tests | The wrist attachment site detected consistently fewer visually counted steps than the waist attachment site at most treadmill speeds during laboratory testing. | N/N | High |
| 493 | Tudor-Locke et al. (2006, Canada) | Adults (N=9, 25-40 yrs, 100% females, NR) | Treadmill protocol (NR) | *Kellog’s Special K step counter* (waist, NR; NR; NR); *Yamax pedometer* (waist, NR; NR; NR) | Intensity (Steps) | Observation (Video) | Absolute percent error, intraclass correlation coefficient | K pedometers are unacceptably inaccurate. | NR/NR | High |
| 494 | Turner et al. (2012, United Kingdom) | Patients with Chronic Respiratory Disease (N=48; 71.25±9.64 yrs; 54.2% females; NR) | Walking (20 minutes) | *Yamax CW-700 Digiwalker* (waist; NR; NR; NR); *SenseWear Pro3* (upper arm; NR; NR; NR) | Intensity (Steps) | Observation (Manually- counted) | Kruskal-Wallis test; Friedman ANOVA; Wilcoxon signed rank test | Pedometers are an inexpensive and simple alternative t activity monitors and appear to be reliable during faster walking in both healthy subjects and patients with chronic respiratory disease However, caution should be applied during slow walking speed because of undercounting steps, causing misleading data that could become demotivating when used as a training adjunct. | N/N | High |
| 495 | Tweedy & Trost (2005, Australia) | Community-dwelling adults (N=14; 32±8 yrs; 21% females; NR) | Posture (5 minutes); Walking (18 minutes) | *MTI ActiGraph* (waist, NR; 1 sec; .NR) | Intensity (Steps) | Indirect Calorimetry | One-way /two way repeated-measures ANOVA, Pearson correlation; Bland–Altman analyses | Actigraph counts provide a valid index of activity across the intensities investigated in this study. For light to moderate activity, Actigraph-based estimates of METs are acceptable for group-level analysis and are a valid means of classifying activity intensity. | N/N | High |
| 496 | Uchimura et al. (2019, United States) | Adults (N=38; 23 yrs; 23.7 % females; NR) | Treadmill (25 minutes) | *Fitbit One* (waist; NR; NR; NR); *Fitbit Surge* (wrist; NR; NR; NR); *Jawbone UP3* (wrist; NR; NR; NR); *Apple watch* (wrist; NR; NR; NR); *Withings Pulse Ox* (waist; NR; NR; NR); *Health Patch* (chest; NR; NR; NR) | Intensity (Steps) | Observation (Video) | Accuracy; Mean absolute percentage error; Bland-Altman analyses | The use of consumer-marketed health-monitoring devices for clinical or medical purposes should be undertaken with caution, especially in the absence of food and drug administration or comparable clearance | N/NR | High |
| 497 | Ummels et al. (2018, Netherlands) | Patients with chronic diseases (N=130; 61.5±11.1 yrs; 56.4% females; NR) | Activities of daily life and walking (19-33 minutes) | *Activ8* (trouser pocket; NR; NR; propretary algorithm); *Digi-Walker CW-700* (wrist; NR; NR; NR); *Fitbit Flex* (wrist; NR; NR; propretary algorithm); *Fitbit one* (waist; NR; NR; propretary algorithm); *Lumoback* (wrist; NR; NR; propretary algorithm); *Jawbone UP24* (wrist; NR; propretary algorithm); *Walking Style X* (waist; NR; NR; NR) | Intensity (Steps, Time spent active) | Observation (Video) | Pearson correlation coefficients; Paired samples t tests; Bland-Altman analyses | Validity of commercially available activity trackers is low measuring steps while individuals with chronic diseases receiving physiotherapy engage in activities of daily living. | N/N | High |
| 498 | Ummels et al. (2020, Netherlands) | Adults (N=20; 74.5 yrs; 50% females; NR) | Activities of daily life (NR) | *MOX Activity Logger* (front trouser pocket; NR; NR; activity classification algorithm); *ActivPAL3* (thigh; PAL Software Suite v7.2.32; NR; NR); *Fitbit Alta HR* (wrist; corresponding Fitbit app; NR; NR) | Intensity (Steps); Posture/Activity Type (Sedentary, standing, dynamic) | Observation (Video) | Bland-Altman analyses; Percentage error; Absolute percentage error; Pearson correlation coefficient; Paired sample t-test; Sensitivity; Specificity; Accuracy | Optimized algorithm parameter settings can more validly estimate step count and physical behavior in older adults wearing an activity tracker in the trouser pocket during ADL compared to reference applications. | N/N | High/High |
| 499 | Valkenet & Veenhof (2019, Netherlands) | Adults (N=12; 49.5±21.5 yrs; 25% females; NR) | Activities of daily life (27 minutes) | *ActiGraph GT9X Link* (hip; NR; 1 sec; NR); *Activ8* (thigh; NR; 5 sec; NR), *Dynaport MoveMonitor* (waist; NR; 1 sec; NR) | Posture/Activity type (Lying, sitting, standing, walking) | Observation (Video) | Sensitivity, Specificity; Positive predictive values; Negative predictive values | The validity outcomes for the categories lying, sitting, standing and walking vary between the investigated accelerometers. All three accelerometers scored good to excellent in identifying walking. None of the accelerometers were able to identify all categories validly. | N/N | High |
| 500 | Vallières & Morin (2003, Canada) | Adults with insomnia (N=17; 41.6±5.7 yrs; 58.8% females; NR) | Sleep (4 nights) | *Actitrac* (wrist; software v.3.15a; NR; IM System algorithm) | Biological state (Total sleep time) | Polysomnography | Means of relative and absolute differences; Accuracy; ANOVA; Cohen´s d effect sizes; Spearman correlation | These results suggest that actigraphy is a useful device for measuring treatment response and that it should be used as a complement to sleep-diary evaluation. | N/NR | Some |
| 501 | Van der Kooi et al. (2013, Netherlands) | Adults in intensive care unit (N=7; 65 yrs (median); 14% females; NR) | Sleep (1 night) | *Actiwatch* (wrist; Actiwatch Sleep Analysis program v1.16; 30 sec; NR) | Biological state (Total sleep time) | Polysomnography | Sensitivity; Specificity; Correlation | The result showed that actigraphy underestimated the amount of wake time and overestimated the amount of sleep. The median specificity for actigraphy was always less than 19% and sensitivity more than 94%. Therefore, actigraphy is not reliable for sleep monitoring in short-stay ICU patients. | N/N | High |
| 502 | Van Hees et al. (2018, United Kingdom, United States) | Adults (Group 1, adults with sleep disorder: N=28; 45±15 yrs; 39% females; NR; Group 2, healthy adults: N=22; 22.8±4.5 yrs; 68% females; NR) | Sleep (1 night) | *GENEActiv* (non-dominant wrist; NR; NR; algorithm cited); *Axivity* (non-dominant wrist; NR; NR; NR) | Biological State (Sleep period time window, sleep episodes) | Group 1: Diary; Group 3: Polysomnography | Multi-level regression; Accuracy; Area under the curve, T-test; Mean absolute error; Bland-Altman analyses | The accuracy of the algorithm to detect the sleep period time window. | N/N | Some |
| 503 | Van Hees et al. (2009, Netherlands) | Adults (N=15, 22±2 yrs; 100 % females; NR) | Walking and activities of daily life (70 minutes) | *DynaPort MiniMod* (lower back; NR: NR; algorithm reported) | Intensity (Energy expenditure) | Direct Calorimetry | Pearson correlation; Standard error of the estimate; Paired sample t-test | The explained variation in AEE by the model was higher than the explained variation by MI alone. This shows that a tri-axial seismic accelerometer is a valid tool for estimating AEE under sedentary conditions. | Y/Y | High |
| 504 | Van Hoye et al. (2014, Belgium) | Adults (N=44; 21.1±1.4 yrs; 45.5% females; NR) | Treadmill (incremental maximal treadmill test) | *SenseWear Pro3* (right arm; Innerview Professional Research Software Version 5.2; NR; algorithm reported) | Intensity (Total energy expenditure) | Indirect Calorimetry | 2-sided paired T-test; Intraclass correlation Coefficients; Bland-Altman analyses | Compared with the IC, the SWA showed no significant difference when walking but significantly underestimated EE at higher speeds starting from a jogging speed for men and from a running speed for women. The underestimation increased significantly with increasing intensity in both male and female participants. | N/NR | Some |
| 505 | Van Laarhoven et al. (2016, Netherlands) | Adults with crutch-support (N=40; 65±9 yrs; 55 % females; NR); Healthy adults (N=8; 49±20 yrs; 37.5% females; NR) | Activities of daily life (NR) | *USB accelerometer X16-mini* (non-affected thigh; MATLAB; NR; algorithm reported) | Posture/Activity type (Walking, standing, resting, stair climbing) | Observation (Video) | Sensitivity; Predictive value; Mean percentage difference; Bland-Altman analyses | The AM was a valid tool for measuring physical activity in these patients. | N/N | Low |
| 506 | Van Remoortel et al. (2012, Greece, Belgium, United Kingdom) | Adults with chronic obstructive pulmonary disease (N=39; 67.9±7.4 yrs; 36% females; NR) | Activities of daily life (59 minutes) | *Kenz Lifecorder* (left waist; Physical Activity Analysis Software; 1 minute; NR); *Actiwatch* (left waist; Respironics Actiware 5; 1 minute; NR); *RT3* (right waist; Stayhealthy RT3 Assist v1.0.7; 1 minute; NR); *ActiGraph GT3X* (right waist; Actilife 5; 1 minute; NR); *Dynaport MiniMod* (waist; NR; 1 minute; NR); *SenseWear Armband* (upper left arm; SenseWear Professional 6.0; 1 minute; NR) | Intensity (Energy expenditure) | Indirect Calorimetry | Pearson correlation; linear regression | The Dynaport MiniMod, Actigraph GT3X and SenseWear Armband (all triaxial monitors) are the most valid monitors during standardized physical activities. The Dynaport MiniMod and Actigraph GT3X discriminate best between different walking speeds. | N/N | Some |
| 507 | Van Rooij et al. (2020, Netherlands) | Adults with a unilateral lower-limb amputation (N=30; 61.8 yrs; 13% females; NR) | Activities of daily life (60 minutes) | *Activ8* (thigh and pocket; NR; 5 sec; NR) | Posture/Activity Type (Sitting, standing, lying, walking, cycling) | Observation (Video) | Bland-Altman analyses | The Activ8 activity monitor has acceptable validity to measure physical activity and sedentary behavior in people with a unilateral lower-limb amputation. | N/N | Some |
| 508 | Vanhelst et al. (2012, France) | Adults (N=21; 29.3±5.1 yrs; 47.6% females; NR) | Treadmill and activities of daily life (60 minutes) | *Vivago* (wrist; NR; 1 min; algorithm reported) | Intensity (Counts; Energy expenditure) | Indirect Calorimetry | Repeated-measure ANOVA; Bland-Altman analyses | Results of the study suggest that the Vivago wrist-worn accelerometer is a valid measure of PA at varying levels of intensity. | N/N | Low |
| 509 | Vanroy et al. (2013, Belgium) | Adults with stroke (N=15; 60.40±10.26 yrs; 40 %, females; NR); Healthy adults (N=15; 58.07±10.37 yrs; 66.7% females; NR) | Treadmill, walking and ergometer cycling (12 minutes and 120m free-living walking). | *Yamax Digi-Walker SW-200* (hip, knee; NR; NR; NR); *SenseWear Pro2* (upper arm; Sense Wear 6.1; NR; algorithm reported) | Intensity (Steps; Energy expenditure) | Observation (Video); Indirect Calorimetry | Spearman correlation; Bland–Altman analyses | YDWP and SWP2A are both reliable. Only knee-worn YDWP is a valid device to measure steps except high intensity walking in stroke. YDWP systematically undercounts steps during other activities of short duration. This study could not demonstrate valid measurement of steps/EE in stroke using SWP2A | N/N | Some |
| 510 | Veerabhadrappa et al. (2018, United States) | Adults (N=71; 18-55 yrs; 33.8% females; NR) | Treadmill (24 minutes) | *Apple Watch* (wrist; NR; 1 minute; NR) | Intensity (Steps) | Observation (Video) | Bland-Altman analyses; Lin’s concordance correlation coefficient | Our study is one of the initial studies to objectively validate the accuracy of the step counts obtained from Apple watch at different walking speeds. Apple Watch tested to be an extremely accurate device for measuring daily step counts for adults. | N/N | High |
| 511 | Vernillo et al. (2014, Italy) | Adults (N=20; 30.1±7.2 yrs; 40 % females; NR) | Treadmill (70 minutes) | *SenseWear Mini* (upper left arm; software V.7.0; NR; NR) | Intensity (Energy expenditure) | Indirect Calorimetry | Two-way mixed model ANOVA; Pearsons correlation; Bland-Altman analyses | The present data suggest that the armband is not accurate to correctly detect and estimate the energy expenditure during pole walking activities. | N/N | High |
| 512 | Vernillo et al. (2015, Italy) | Adults (N=20; 30.1±7.1 yrs; 40 % females; NR) | Treadmill (40 minutes) | *SenseWear Armband* (right upper arm; software V.6.1; NA; proprietary algorithm); *SenseWear Mini Armband* (left upper arm; software.7.0; NA; algorithm V.2.2.3) | Intensity (Energy expenditure) | Indirect Calorimetry | Two-way mixed model ANOVA; Pearsons correlation; Bland-Altman analyses | Both the armbands produced similar EE values and seem to be nor accurate in estimation EE during activities involving uphill and downhill walking. | N/NR | High |
| 513 | Vetrovsky et al. (2019, Czech Republic) | Adults (N=20; 34.3±11.6 yrs; 25% females; NR) | Treadmill (12 minutes) | *Withings Go* (non-dominant wrist, NR; NR; NR); *Fitbit Charge 2* (dominant wrist; NR; NR; NR); *Garmin vivofit* (wrist; NR; NR; NR); *Garmin vivofit 3* (wrist; NR; NR; NR); *Omron HJ-322U-E* (waist; NR; NR; NR); *SmartLAB walk+* (neck; NR; NR; NR) | Intensity (Steps) | Observation (Manually-counted) | Concordance correlation coefficients; Mean absolute percentage errors; Bland-Altman analyses | Even though none of the tested activity monitors fall within arbitrary thresholds for validity, most of them perform reasonably well enough to be useful tools that clinicians can use to simply motivate chronic heart failure patients to walk more. | N/N | High |
| 514 | Vieira Costa et al. (2019, Brazil) | Adults with chronicity after stroke (N=55; 62.5±14.9 yrs; 45.5% females; NR) | Walking (2 minutes) | *Fitbit Ultra* (NR; NR; NR; NR); *Google fit* (front pocket; NR; NR; NR) | Intensity (Steps) | Observation (Video) | Pearsons correlation coefficients; Intra-class correlation coefficient; Bland-Altman analyses | mHealth devices (Pacer–iphone, Fitbit Ultra, Google Fit, and Pacer–Android) are valid and reliable for step counting in chronic stroke survivors. Body location (paretic or non-paretic side) does not affect validity or reliability of the step count metric. | N/N | High |
| 515 | Wahl et al. (2017, Germany) | Adults (N=20; Males: 26±2.8 yrs, females: 24.2±1.9 yrs; 50% females; NR) | Treadmill (20 minutes) and outdoor walking (2.4 km) | *Sensewear* (upper arm; NR); *Withings Pulse O_x_* (upper arm, hip; NR; NR; NR); *Beurer AS80, Polar Loop, Garmin Vivofit, Garmin Vivosmart, Garmin Vivoactive, Garmin Forerunner 920XT, Fitbit Charge HR* (all wrist; NR; NR; NR) | Intensity (Steps; Energy expenditure) | Optogait system and Observation (Manually-counted); Indirect Calorimetry | Mean absolute percentage error; Intraclass Correlation Coefficient; Typical error; Bland-Altman analyses | Most Wearables provide an acceptable level of validity for step counts at different constant and intermittent running velocities reflecting; sports conditions. However, the covered distance, as well as the EE could not be assessed validly with the investigated Wearables. Consequently, covered distance and EE should not be monitored with the presented Wearables, in sport specific conditions. | N/N | High |
| 516 | Walch et al. (2019, United States) | Adults (N=39; 29.42±8.52 yrs; 68% females; NR) | Sleep (7- to 14 nights) | *Apple Watch Series 2 and 3* (wrist; Mobile Application developed by OW; 30 sec; classification algorithms) | Biological state (Total sleep time) | Polysomnography | Accuracy; Sensitivity; Specificity; Kappa statistics | This study demonstrates, for the first time, the ability to analyze raw acceleration and heart rate data from a ubiquitous wearable device with accepted, disclosed mathematical methods to improve accuracy of sleep and sleep stage prediction. | N/N | High |
| 517 | Wallmann-Sperlich et al. (2014, Germany) | Adults (N=30; 24±3; 50% females; NR) | Treadmill (55 minutes) | *Omron Walking Style HJ-304* (both hips, both pockets, backpack; NR; NR; Weir formula), *Yamax Digiwalker SW-700* (both hips; NR; NR; Weir formula) | Intensity (Steps, Energy expenditure) | Indirect Calorimetry | Absolute percent error | The Omron pedometer provides accurate step counts when worn on the hip and backpack at all tested speeds and inclinations. | Y/N | High |
| 518 | Wang et al. (2008, Australia) | Adults with and without obstructive sleep apnea (N=21; 38.9±13.0 yrs; NR; NR) | Sleep (1 night) | *Actiwatch 64* (nondominant wrist; Actiware 5.0; 30 sec; standard factory-default algorithm) | Biological state (Total sleep time) | Polysomnography | Agreement; Sensitivity; Specificity; Cohen’s kappa statistic; Intraclass correlation coefficients; Paired t-test; Pearson correlation | Contrary to prior reports, epoch-by-epoch comparison of sleep/wake scoring showed similar fair agreement between actimetry and PSG in subjects with or without OSA. | N/N | Some |
| 519 | Wang et al. (2017, China) | Adults (N=9; 22±1 yrs; 44% females; NR) | Walking (6*400 meters), treadmill (5 minutes) | *Huawei B1, Mi Band, Fitbit Charge, Polar Loop, Garmin Vivofit2, Misfit Shine, Jawbone Up* (all wrist; Firmware each; NR; shank IMU-based algorithm) | Intensity (Steps) | Wearable (MATLAB algorithm based on angular velocity from an IMU (shank)) | Absolute percentage error; Meane absolute percentage error; Bland-Altman analyses; ANOVA | When comparing these monitors performance of the entire experiments, Garmin Vivofit2 showed the best accuracy (average MAPE of 3.51%) and Misfit Shine showed the worst (average MAPE of 12.63%). | NR/NR | High |
| 520 | Warms & Belza (2004, United States) | Adult wheelchair users (N=6; 37.3±8.3 yrs; 33.3% females; NR) | Activities of daily lives (approx. 20 minutes.) | *Actiwatch* (non-dominant wrist; actiware software; 15 sec; NR) | Intensity (Activity counts) | A self-report physical activity record | Pearson Correlation; T-Tests | Actigraphy is suitable as a measurement of activity for people with spinal cord injury. This initial investigation suggests that it has concurrent validity with a selfreport measure of activity intensity and frequency | N/NR | High |
| 521 | Webber & John (2016, Canada) | Geriatric rehabilitation patients (N=38; 83.2±7.1 yrs; 89.5% females; NR) | Walking (NR) | *ActiGraph GT3X+* (right hip, left ankle; Actilife 6; 1 sec; default analysis, algorithm; LFE algorithm); *StepWatch* (right ankle; StepWatch software 3.1b; 3 sec; NR) | Intensity (Steps) | Observation (Manually- counted) | Intraclass correlation coefficients; Bland-Altman analyse; Kruskal-Wallis ANOVA | Although these finding suggest the GT3X+ (ankle, LFE) functions as well as the StepWatch in detecting steps during walking in older adults with slow gait speeds, further research is needed to determine whether the GT3X+ is also able to disregard other body movements (e.g., fidgeting) that occur when full day monitoring is utilized. | N/NR | High |
| 522 | Webber et al. (2014, Canada) | Adults (N=35; 81.5±5.0 yrs; NR; NR) | Walking (100 meters) | *SC-StepMX* (waist; NR; NR; NR); *Yamax SW200 Digi-Walker* (waist; NR; NR; NR); *ActiGraph GT3X+* (waist; ActiLife5; 1 sec; analysis algorithms) | Intensity (Steps) | Observation (Manually- counted) | Percentage error; Kruskal-Wallis ANOVA; Bland-Altman analyses; Spearman correlation | These results support using a piezoelectric pedometer for measuring steps in older adults who use walking aids and who walk slowly. | N/N | High |
| 523 | Welk et al. (2003, United States) | Adults (N=181; 32.7±10.3 yrs; 46.4% females; NR) | Treadmill (18 minutes) | *Biotrainer* (hip; NR; NR; NR); *Actitrac* (hip; NR; NR; NR) | Intensity (Counts; Energy expenditure) | Indirect Calorimetry | Accuracy; Bland-Altman analyses | The results support the validity of Biotrainer and Actitrac monitors for estimating energy expenditure under controlled conditions. | NR/NR | High |
| 524 | Welk et al. (2017, United States) | Adults (N=27; 27.6±9.2 yrs; 44.4% females; NR) | Treadmill (15 minutes) | *Metria IH1* (left arm; NR; NR; proprietary algorithms) *Sensewear* (left arm; NR; NR; proprietary algorithms) | Intensity (Energy expenditure) | Indirect Calorimetry | Pearson correlation; Formalized equivalence testing procedures; Mean absolute percent error | The disposable nature of the adhesive Metria IH1 monitor offers promise for clinical evaluation of physical activity behavior in patients. Additional research is needed to test utility for counseling and behavior applications. | Y/N | High |
| 525 | Wellons et al. (2019, United States) | Adults with vestibular disorders (N=26; 61.5±12.4 yrs; 65.4% females; NR) | Walking (2 minutes) | *StepWatch* (ankle; NR; NR; NR) | Intensity (Steps) | Observation (Video) | One sample t-test; Bland-Altman analyses; Linear regression | Results indicate that the SAM accurately measures step count in individuals with vestibular disorders. | N/N | High |
| 526 | Wendel et al. (2018, United States) | Adults with Parkinson Disease (N=33; 65.48±9.39 yrs; 42% females; NR) | Walking (2 hrs) | *Fitbit Zip* (waist; NR; NR; proprietary algorithm); *Fitbit Surge* (wrist; NR; NR; proprietary algorithm); *Jawbone Up 2* (wrist; NR; NR; proprietary algorithm); *Jawbone Up Move* (waist; NR; NR; proprietary algorithm) | Intensity (Steps) | Observation (Video) | Bland-Altmann analyses; Intraclass correlation; Mean absolute percent error | In persons with mild-to-moderate PD, waist-worn activity trackers may be prescribed to monitor bouts of continuous walking with reasonable accuracy; however, activity trackers have little utility in monitoring discontinuous walking common in household settings. | N/N | High |
| 527 | Wetten et al. (2014, Australia) | Adults (N=23; 25.3±6.3 yrs; 52.3% females; NR) | Exercise and sedentary activities (4 hrs) | *ActiGraph GT1M* (hip; Actilife v.4.1.1; 10 sec; Work-Energy Theorem and Freedson Equation); *RT3* (hip; Assist v1.0.7; 1 min; proprietary manufacturer equations); *SenseWear* (upper arm; SenseWear Professional 3 v6.1.0; 1 min; proprietary manufacturer equations) | Intensity (Energy expenditure) | Indirect room Calorimetry | Repeated measures MANOVA; Bland-Altman analyses; Percentage difference | The Actigraph and SWA are both valid tools for quantifying EE during light-intensity stepping. These results provide further valuable information on how accelerometer devices may be appropriately used. | N/N | Some |
| 528 | Wetzler et al. (2003, France) | Adults (N=26; 50±20 yrs; 42.3% females; NR) | Activities of daily life (approx. 30 minutes) | *Quattrolter* (waist, thigh; NR; NR; NR) | Posture/Activity type (Standing, sitting, lying) | Observation (Direct) | Error in transition time; Accuracy | The combination of an accelerometer placed on the subject’s thigh and a position sensor located at the subject’s waist appeared to be a suitable system for position/activity monitoring during ambulatory ECG and blood pressure monitoring | NR/NR | High |
| 529 | Whithrow et al. (2019, United States) | Adults with insomnia (N=30; 45.1 yrs; 83.3% females; Caucasian (N=10), African-American (N=17), Asian (N=3)) | Sleep (1 night) | *Actiwatch Spectrum Plus* (non-dominant wrist, Philips Actiware v6; 30 sec; NR) | Biological State (Total sleep time) | Polysomnography | Bland-Altman analyses; Paired t-test | Differences between the PSG night and the following night at home were found, with better sleep on the first night home. | N/N | High |
| 530 | Whybrow et al. (2013, United Kingdom) | Adults (N=14; 38.9±10.4 yrs; 50% females; NR) | Activities of daily life (24 hrs) | *Intelligent Device for Energy Expenditure and Activity* (two at feet, two at thigh, one at chest; IDEEA software; NR; NR) | Intensity (Energy expenditure) | Indirect Calorimetry | Student’s t tests; Pearson correlation coefficient; Lin’s Concordance coefficient; Bland–Altman plots; ANOVA | IDEEA methods overestimated EE compared to the calorimeter. | N/N | High |
| 531 | Wong et al. (2017, United States) | Adults (N=25; 25.96±7.86 yrs; 52% females; NR) | Treadmill (12 minutes) | *FitBit Ultra (*waist; NR; 1 minute; NR) | Intensity (Steps) | Observation (Video) | ANOVAs | This study highlights problems with using the FitBit Ultra by slow-walking populations, and recommends that researchers and clinicians should carefully consider the trade-off between accuracy and convenience when using commercial activity trackers with slow-walking populations. | N/N | High |
| 532 | Woodman et al. (2017, United States) | Adults (N=28; 25.5±3.7 yrs; 29% females; NR) | Activities of daily life (40 minutes); Treadmill (5 minutes); Ergometer (5 minutes) | *Basis Peak* (non-dominant wrist; NR; NR; NR); *Garmin Vivofit* (non-dominant wrist; NR; NR; NR); *Withings Pulse* (right hip, shirt collar, dominant wrist; NR; NR; Benedict equation) | Intensity (Energy expenditure); Posture/Activity Type (Activity types) | Indirect Calorimetry | ANOVA; Bland-Altman analyses | The Basis Peak was the only device that did not significantly differ from measured EE; however, it also had the largest individual errors. Additionally, the Basis Peak accurately predicted minutes spent walking and running, but not cycling. | N/N | High/High |
| 533 | Wu et al. (2021, Canada) | Normotensive adults (N=40; 25±6 yrs; 60% females; NR) | Treadmill (42 minutes) | *ActivPAL* (each thigh, PAL Software Suite; NR; activPAL-linear equation) | Intensity (Energy expenditure) | Indirect Calorimetry | Shapiro-Wilk test, Mauchly’s test, Greenhouse-Geisser correction factor, Bonferroni post-hoc testing, Bland-Altman method | The activPAL-curvilinear estimated values statistically equivalent to indirect calorimetry for treadmill stages 1–6 (1.5–4.0 miles•hour^−1^) | N/N | High |
| 534 | Xie et al. (2018, China) | Adults (N=44; Males: 22.2±2.2 yrs, females: 22.5±2.1 yrs; 47.7% females; NR) | Walking (800 meters) and cycling (3 routes) | *Apple Watch 2, Samsung Gear S3, Jawbone UP3, Fitbit Surge, Huawei Talk Band* *B3, Xiaomi Mi Band 2* (All wrist; NR; NR; NR) | Intensity (Steps; Energy expenditure) | Observation (Video); Indirect Calorimetry | Mean absolute percentage errors; Spearman correlation; Pairwise t-test | At present, mainstream devices are able to reliably measure heart rate, number of steps, distance, and sleep duration, which can be used as effective health evaluation indicators, but the measurement accuracy of energy consumption is still inadequate. | N/N | High |
| 535 | Yang et al. (2018, China) | Adults (N=19; 28.2±3.8 yrs; 47% females; NR) | Activites of daily life (12 hours) | *Fibion* (thigh, pocket of the trousers; NR; 1 minute; NR | Intensity (Energy expenditure; Time spent in LPA, MVPA); Posture/Activity type (Sitting, lying, standing, walking, cycling) | Observation (direct); Indirect Calorimetry | Student’s t-tests; Wilcoxon signed-rank test; Intraclass correlation coefficient; Bland-Altman analyses | Low correlations between subsequent measurements of both devices indicated large random measurement errors, which were somewhat diminished during the simulated 12 h real-life test. | N/Y | High/High |
| 536 | Yokoyama et al. (2002, Japan) | Students (N=39; 20-24 yrs; 36% females; NR); White-collar workers (N=6; 39-44 yrs; 0 % females; NR) | Treadmill (25 minutes); Ergometer (5 minutes) | *Calorie Counter Select 2* (right waist; NR; 4 sec; equation reported) | Intensity (Energy expenditure) | Indirect Calorimetry | Student’s t-test; coefficient of variance; Pearson correlation | Accelerometry is a reasonably accurate and feasible method for evaluating the physical activities of non-athletes. | NR/NR | High |
| 537 | Yoon et al. (2018, South Korea) | Adults: Group 1: low respiratory disturbance index (N=15; 40.73±11.42 yrs; 53.3% females; NR), Gorup 2: high rdi (N=15; 48.80±7.94 yrs; 47% females; NR) | Sleep (1 night) | *T-REX* (chest, NR; 30 sec; algorithm reported) | Biological state (Total sleep time) | Polysomnography | Kappa statistics; Accuracy; Sensitivity; Specificity; Bland–Altman analyses | Wakefulness-related information was successfully provided using data from the patch-type device. | N/N | High |
| 538 | Zambotti et al. (2015, United States) | Adults (N=28; 50.1±3.9 yrs; 100% females; NR) | Sleep (1 or 2 nights) | *Jawbone UP* (wrist; Jawbone UP mobile app; 1 minute; proprietary algorithm) | Biological state (Total sleep time) | Polysomnography | Sensitivity; specificity; Bland-Altman analyses; Wilcoxon signed-rank tests; Mann–Whitney U tests | Data suggest that Jawbone UP provides acceptable levels of agreement with PSG measures, when the overall night is considered, and thus may be a feasible alternative for ecologically monitoring sleep-wake rhythms over several days | N/N | High |
| 539 | Zambotti et al. (2018, United States) | Adults (N=44; main sample: 35±12 yrs; Periodic limb movement of sleep: 42±15 yrs; 59% females; 57% Caucasian) | Sleep (1 night) | *Fitbit Charge 2* (non-dominant wrist; Fitbit App; 30 sec; NR) | Biological state (Total sleep time) | Polysomnography | Paired t-tests; Bland–Altman analyses; Sensitivity; Specificity; Accuracy | Fitbit Charge 2 shows promise in detecting sleep-wake states and sleep stage composition relative to gold standard PSG, particularly in the estimation of REM sleep, but with limitations in N3 detection. | Y/NR | High |
| 540 | Zanetti et al. (2014, Australia) | Adults (N=14; 22±4 yrs; 0% females; NR) | Exercise (42 minutes) | *SenseWear Mini Armband* (left upper arm; SenseWear Professional v7.0; NR; proprietary algorithm) | Intensity (Energy expenditure) | Indirect Calorimetry | Pearson correlation; coefficient; Cohen effect size scores | The SWA did not provide a valid measure of energy expenditure during rugby-specific intermittent exercise or 10-minute postexercise recovery. | NR/NR | High |
| 541 | Zhang et al. (2003, United States) | Adults (N=76; 36.3±14.9 yrs; 56.6% females; NR) | Posture (220 seconds); limb movements (50 seconds); walking (180m); Stair climbing (48 steps) | *IDEEA* (chest, each thigh, each foot; NR; NR; NR) | Intensity (Energy expenditure); Posture/ Activity type (Lying; reclining; sitting; standing; leaning; jumping; walking; running; up stairs; down stairs; biking) | Observation (Direct) | Intraclass correlation; Accuracy; Pooled correlation | IDEEA accurately measured duration, frequency, type, and intensity of a variety of daily PAs. | N/NR | High |
| 542 | Zhang et al. (2016, Hong Kong) | Adults (N=45; 20.0±4.5 yrs; 48.9% females; NR) | Treadmill (32 minutes) | *ActiGraph GT3X+* (chest, wrist; Actilife v6; 1 sec; NR) | Intensity (Counts) | Wearable (ActiGraph GT3X+ (waist)) | Dependent t-tests; Cohen effect sizes; Pearson correlation; Absolute percentage errors; Bland-Altman analyses | PA measurements recorded by a Chest worn GT3X+ more closely resembled PA measurements recorded at the traditional Waist site than when compared to the Wrist site. | N/N | High |
| 543 | Zhang et al. (2018, United States) | Adults (N=30; 20.2±1.7 yrs; 43% females; NR) | Treadmill (60 minutes) | *Apple Watch series 1* (wrist; workout app; NR; NR) | Intensity (Energy expenditure) | Indirect Calorimetry | Two-way repeated measure ANOVA; Pearson correlation; Regression analysis | Apple Watch demonstrated a low to moderate validity and reliability on measuring EE. | N/NR | High |
| 544 | Zhu & Lee (2010, United States) | Adults (N=40; 43.55±11.68 yrs; 50% females; Caucasians (N=32), African Americans (N=3), from other minority groups (N=5)) | Outdoor walking (1000 steps, 1253 meters); Stair climbing (80 steps) | *BI pedometer* (waist, pant’s front pockets, shirt pocket, bag carried on the left side, neck, backpack; NR; NR; proprietary algorithm); *Yamax Digi-Walker SW-200* (waist; NR; NR; NR); *Dynastream AMP 331* (ankle; NR; NR; NR) | Intensity (Steps) | Observation (Manually counted) | Absolute percentage error; Repeated ANOVA; Intraclass correlation coefficient; Accuracy | The Omron BI pedometer can accurately count steps when worn at various locations on the body in free-living conditions except for front pant pocket locations, especially when climbing stairs. | Y/NR | High |
| 545 | Zorrilla-Revilla et al. (2017, Spain) | Adults (N=41; males: 33.26±7.25 yrs; females: 28.79±6.44 yrs; 34% females; NR) | Treadmill (44 minutes) | *SenseWear mini* (dominant upper arm; BodyMedia Professional Software v7.0; NR; proprietary algorithms) | Intensity (Energy expenditure) | Indirect Calorimetry | Mean absolute percentage error; T-tests; Simple regressions; Bland–Altman analyses; One-way ANOVA; Pearson correlation | The SWA mini is not a valid device for estimating energy expenditure in brief light- or moderate activities. | N/N | High |
| ^1^ NR=Not reported; ^2^ N= No; ^3^Y=Yes | | | | | | | | | |  |
