# Supplementary material for: Assessment of 24-hour physical behaviour in adults via wearables: a systematic review of validation studies under laboratory conditions
Source: Int J Behav Nutr Phys Act. 2023 Jun 8;20:68. doi: 10.1186/s12966-023-01473-7 (PMC10249261; doi:10.1186/s12966-023-01473-7)
Supplement: Supplementary file 6 — Additional file 6 [file 12966_2023_1473_MOESM6_ESM.docx]

**Additional file 6.** Overview of wearables used in validation studies.

| **Model (Manufacturer)** | **Type** | **Memory/Battery life** | **Feedback display (yes/no)** | **Epoch-length** | **Dynamic range [g]** | **Sampling frequency [Hz]** | **Dimensions (W x D x H) [mm]** | **Weight [g]** | **Used in No. Studies** |
| --- | --- | --- | --- | --- | --- | --- | --- | --- | --- |
| **3dNX accelerometer model v3** (BioTel Ltd., Bristol, UK) | Research-grade triaxial accelerometer | Approx. 21 days | No | 5-60 sec | ± 5.5 | 100 | 54 x 54 x 18 | 70 | 2 |
| **Accusplit-AX120** (Accusplit Inc., Livermore, CA, USA) | Commercial-grade pedometer | NR | Yes | NR | NR | NR | NR | 90.8 | 1 |
| **Accusplit AX2710** (Accusplit, Inc., Livermore, CA) | Commercial-grade pedometer | NR/ >1 year | Yes | NR | NR | NR | NR | NR | 1 |
| **Accusplit Eagle 120** (Accusplit, San Jose, CA) | Commercial-grade pedometer | NR | Yes | NR | NR | NR | NR | NR | 1 |
| **Accusplit Eagle Digi-Walker 2** (AST, San Jose, California) | Commercial-grade pedometer | NR | Yes | NR | NR | NR | NR | NR | 1 |
| **Acos FS500** (Acos Co. Ltd., Iida, Japan; AC) | Commercial-grade pedometer | NR | NR | NR | NR | NR | NR | NR | 1 |
| **Actibelt** (Trium Analysis Online GmbH) | Research-grade triaxial accelerometer | 512MB/ 2 months | No | NR | ± 6 | 100 | NR | NR | 1 |
| **Actical accelerometer** (Philips Respironics, Inc., Murrysville PA, USA) | Research-grade uniaxial accelerometer | NR | No | 1-5 sec | NR | 32 | 28 x 27 x 10 | 17 | 16 |
| **Actical Z series** (Mini-Mitter Philips Respironics, Inc., Bend, OR, USA) | Research-grade triaxial accelerometer | NR | No | 1- 60 sec | 0.05-2 | 32 | NR | NR | 2 |
| **ActiGraph AM7164** (MTI Health Services, Florida, USA) | Research-grade uniaxial accelerometer | 32 MB/4 days | No | 10 sec | 0.05-2.13 | 10 | 51 x 41 x 15 | 37.8 | 18 |
| **Actigraph AMA-32** (Ambulatory Monitoring, Inc., Ardsley, NY, USA) | Research-grade accelerometer | 32KB/ NR | NR | 1 min | NR | 0.1-10 | 40 x 31 x 10 | 57 | 1 |
| **ActiGraph GT1M** (ActiGraph, LLC, Pensacola, FL, USA) | Research-grade uniaxial accelerometer | 1 MB/14 days | No | 5 sec | 0.05-2 | 30 | 38 x 37 x 18 | 27 | 17 |
| **ActiGraph GT3X** (ActiGraph, LLC, Pensacola, FL, USA) | Research-grade triaxial accelerometer | 2 GB/31 days | No | 1-60 sec | 0.05-2 | 30 – 100 | 38 x 37 x 18 | 27 | 40 |
| **ActiGraph GT3X+** (ActiGraph, LLC, Pensacola, FL, USA) | Research-grade triaxial accelerometer | 2 GB/31 days | No | 1-60 sec | ± 6 | 30 – 100 | 46 x 33 x 15 | 19 | 55 |
| **ActiGraph GT9X** (ActiGraph Inc, Pensacola, FL, USA) | Research-grade triaxial accelerometer | 4 GB/16 days | Yes | 1-60 sec | ± 8 | 30 - 100 | 35 x 35 x 10 | 14 | 13 |
| **Actiheart** (CamNtech, Cambridge, UK) | Research-grade triaxial accelerometer | 1 GB/21 days | No | 15, 30, 60 sec | ± 2.5 | 100 | 39.7 x 30.2 x 9.25 | 10.5 | 10 |
| **ActiPed** (FitLinxx Inc., Shelton, CT, USA) | Uniaxial accelerometer | 30 days/6 months | No | NR | NR | NR | 35 x 27 x 11 | 15 | 1 |
| **ActiReg** (PreMed AS, Oslo, Norway) | NR | 30 days/ | NR | NR | NR | NR | 85 x 45 x 15 | 60 | 2 |
| **Activ4Life Pro** (Activ4LifeHealthcare Technologies Ltd, Boroughbridge, UK) | Triaxial accelerometer | NR | NR | NR | NR | NR | 38 x 32 x 10 | NR | 1 |
| **Actillume recorder** (Ambulatory Monitoring, Inc., Ardsley, NY) | Research-grade triaxial accelerometer | 32 K RAM/ 7 days | NR | 60 sec | 0.003 | 20 | 10 x 30 x 60 | 85 | 2 |
| **Activ8** (Remedy Distribution Ltd., Valkenswaard, The Netherlands) | Commercial-grade triaxial accelerometer | NR/ >30 days | No | 5 sec – 5 min | ± 4 | 12.5 | 34 x 30 x 10 | 20 | 8 |
| **ActivPAL** (PAL Technologies Limited, Glasgow, UK) | Research-grade triaxial accelerometer | 16 MB/10 days | No | 15 sec | ± 2 | 20 | 50 x 35 x 7 | 15 | 32 |
| **ActivPAL micro** (PAL Technologies Ltd, Glasgow, Scotland, UK) | Research-grade triaxial accelerometer | NR | No | NR | NR | 20/80 | 23.5 x 43 x 5 | 9.5 | 2 |
| **ActivPAL3** (PAL Technologies Limited, Glasgow, UK) | Research-grade triaxial accelerometer | 16 MB/7 days | No | 1 sec | ± 2 | 20/80 | 53 × 35 × 7 | 15 | 16 |
| **ActivPAL micro 3** (PAL Technologies Ltd, Glasgow, Scotland**)** | Research-grade triaxial accelerometer | 7 days | No | 15 sec | ± 2 | 20 | 23.5 x 43 x 5 | 9.5 | 2 |
| **ActivPAL micro 4** (PAL Technologies Ltd, Glasgow, Scotland**)** | Research-grade triaxial accelerometer | NR | No | 15 sec | ± 2-4 | 20 | 23.5 x 43 x 5 | NR | 1 |
| **Actimarker** (Panasonic Electronic Works, Ltd., Osaka, Japan) | Research-grade triaxial accelerometer | NR | NR | 12 sec | NR | 0.3-100 | 60 x 35 x 12 | 30 | 4 |
| **Actitrac** (IM Systems, Inc., Baltimore, Maryland) | Research-grade biaxial accelerometer | NR | NR | NR | NR | 40 | NR | NR | 3 |
| **Actiwatch activity monitor** (Mini Mitter Company, Inc., Sunriver, OR, USA) | Research-grade uniaxial accelerometer | NR | NR | NR | NR | 32 | 27 x 26 x 9 | NR | 5 |
| **Actiwatch-2** (Philips Respironics Inc., Murrysville, PA, USA) | Research-grade biaxial accelerometer | 1 MB/30 days | No | NR | 0.5-2 | 32 | 43 x 23 x 10 | 16 | 14 |
| **Actiwatch 4 (**Cambridge Neurotechnology Ltd, Cambridgeshire, United Kingdom) | Uniaxial accelerometer | 64 KB | NR | 2 sec - 15 min | 0.05 | 32 | 37 x 29 x 10 | 16 | 3 |
| **Actiwatch 64** (Mini-Mitter, Inc., Bend, Ore, USA) | Research-grade uniaxial accelerometer | 64 KB/180 days | Yes | 15 sec - 15 min | NR | 32 | 29 x 37 x 12 | 16 | 9 |
| **Actiwatch-L** (Mini Mitter Co., Inc., Respironics, Inc., Bend, OR) | Research-grade uniaxial accelerometer | 64 KB/180 days | Yes | 15 sec - 15 min | NR | 32 | 29 x 37 x 11 | 16 | 5 |
| **Actiwatch Spectrum** **Plus** (Philips Healthcare, Andover, MA, USA) | Research-grade uniaxial accelerometer | 1 MB/8 months | Yes | 30 sec | 0.5-2 | 32 | 48 x 37 x 14 | 30 | 10 |
| **A&D 101NFC Activity Monitor** (A&D Company Ltd., Tokyo, Japan) | Research-grade triaxial accelerometer | 14 days/ >1 year | Yes | NR | NR | NR | 75 x 34 x 11 | 25 | 1 |
| **ADAMO Care Watch** (Analog Devices, Norwood, MA, USA) | Research-grade triaxial accelerometer | NR | Yes | NR | NR | 50 | NR | NR | 1 |
| **ADXL05** (Analog Devices, Norwood, MA, USA) | Research-grade uniaxial accelerometer | NR | NR | NR | ± 5 | 40 | NR | NR | 1 |
| **ADXL202** (Analog Devices BV Ltd, Limerick, Ireland) | Research-grade biaxial accelerometer | NR | NR | NR | ± 2 | 0.01-5000 | 5 x 5 x 2 | 5 | 1 |
| **APDM Opal** (APDM Wearable Technologies Inc., Portland, OR, USA) | Research-grade triaxial acclerometer | >30 days/ 16 hours | No | NR | NR | NR | NR | NR | 1 |
| **Apple Watch** (Apple Inc., Los Altos, CA; USA) | Commercial-grade triaxial accelerometer | NR | Yes | NR | NR | NR | 38.6 x 33.3 x 10.5 | 25 | 21 |
| **Apple Watch 2** (Apple Inc., Los Altos, CA; USA) | Commercial-grade triaxial accelerometer | <18 Std. | Yes | NR | NR | NR | 38.6 x 33.3 x 11.4 | 28.2 | 4 |
| **Apple Watch 3** (Apple Inc., Los Altos, CA; USA) | Commercial-grade triaxial accelerometer | 8 GB/ NR | Yes | NR | NR | NR | 38.6 x 33.3 x 11.4 | 27 | 1 |
| **Apple Watch 4** (Apple Inc., Los Altos, CA; USA) | Commercial-grade triaxial accelerometer | 16 GB/ <18 hours | Yes | NR | <32 | NR | 40 x 34 x 10.7 | 30 | 1 |
| **Axivity AX3** (Axivity Ltd., York, UK) | Research-grade triaxial accelerometer | 512 MB/30 days | No | NR | ± 16 | 12.5 - 3200 | 23 x 32.5 x 8.9 | 11 | 6 |
| **B3** (Huawei Technologies Co Ltd, Longgang District Shenzhen, China) | Commercial-grade accelerometer | 512 KB/ NR | Yes | NR | NR | NR | 210 x 21.7 x 12.2 | 95.8 | 1 |
| **Basis B1 Band** (Basis Science, Inc., San Francisco, CA, USA) | Commercial-grade triaxial accelerometer | 4 days | Yes | 30 sec | NR | NR | 36 x 273 x 27 | 44 | 5 |
| **Basis Peak** (Intel, Santa Clara, CA, USA) | Commercial-grade triaxial accelerometer | 4 days | Yes | NR | NR | NR | 36 x 273 x 27 | 24 | 2 |
| **Beurer AS80** (Beurer GmbH, Ulm, Germany) | Commercial-grad triaxial accelerometer | 30 days/ NR | Yes | NR | NR | NR | 254 x 18 x 11 | 17 | 1 |
| **BioPatch ZephyrLife** (BioPatch ZephyrLife; Annapolis, Maryland, United States) | Research-grade triaxial accelerometer | NR | Yes | 30 sec - 1 min | NR | 100 | NR | NR | 1 |
| **Biotrainer** (IM Systems, Baltimore, MD, USA) | Research-grade uniaxial accelerometer | NR | NR | 1 min | NR | NR | NR | NR | 1 |
| **Biotrainer Pro** (IM Systems, Inc., Baltimore, MD, USA) | Research-grade uniaxial accelerometer | 112 days/ NR | NR | 15 sec – 5 min | NR | 10 | NR | NR | 1 |
| **BodyMedia FIT** (BodyMedia Inc., Pittsburgh, PA) | Commercial-grade triaxial accelerometer | 2 weeks/ 7 days | No | 1 min | NR | 1 | 55 x 62 x 13 | 45.4 | 2 |
| **Calorie Counter Select 2** (Suzuken Co Ltd, Japan) | Research-grade accelerometer | NR | NR | 4 sec | NR | NR | NR | NR | 1 |
| **Caltrac** (Muscle Dynamics Fitness network, Torrance, CA, USA) | Research-grade uniaxial accelerometer | NR | No | NR | NR | NR | 70 x 70 x 20 | 78 | 7 |
| **CAM** (Maastricht Instruments BV, Maastricht, Netherlands) | Research-grade triaxial accelerometer | 2 GB/ NR | NR | 1 sec | ±4 | 25 | 63 x 45 x 18 | 100 | 2 |
| **Citizen TW600** (Citizen Systems Japan Co., Ltd., Tokyo, Japan; CTZ) | Commercial-grade pedometer | 14 days/ 6 months | Yes | NR | NR | NR | 73 x 31 x 10 | 25 | 1 |
| **Core Armband Monitor** (BodyMedia, Inc. Pittsburgh, PA, USA) | Commercial-grade triaxial accelerometer | NR/ 14 days | No | NR | NR | NR | NR | 16 | 1 |
| **DirectLife monitor** (DirectLife, Philips Lifestyle Incubator, Amsterdam, The Netherlands) | Commercial-grade triaxial accelerometer | 22 weeks/ 3 weeks | No | NR | NR | NR | 32 x 32x 5 | 12.5 | 2 |
| **Dunlop pedometer** (Dunlop Sport, Surrey, United Kingdom) | Commercial-grade pedometer | NR | Yes | NR | NR | NR | NR | NR | 1 |
| **Dynastream AMP-331** (Dynastream Innovations, Inc., Alberta, Canada) | Research-grade triaxial accelerometer | 7 days | Yes | NR | NR | NR | NR | NR | 3 |
| **Dynaport ADL Monitor** (McRoberts, Den Haag, Netherlands) | Research-grade triaxial accelerometer | 10 MB/1 day | No | 1-60 sec | 14 | 32 | 125 x 95 x 34 | 295 | 5 |
| **Dynaport MoveMonitor MicroMod** (Mc Roberts BV, Den Haag, Netherlands) | Research-grade triaxial accelerometer | 64 MB/ 72 hours | NR | NR | NR | 100 | 83 x 51 x 8 | 40 | 1 |
| **DynaPort MiniMod** (McRoberts, TheHague, Netherlands) | Research-grade triaxial accelerometer | NR/ 72 hours | No | NR | NR | 100 | 84 x 50 x 8 | 44.5 | 4 |
| **Dynaport MoveMonitor** (Mc Roberts BV, Den Haag, Netherlands) | Research-grade triaxial accelerometer | 7 days | NR | NR | NR | NR | 84 x 50 x 8 | 70 | 1 |
| **Epson PULSENSE** (Seiko Epson, Suwa, Japan) | Commercial-grade triaxial accelerometer | NR/ 36 hours | NR | NR | NR | NR | NR | NR | 1 |
| **ePulse Personal Fitness Assistant** (Impact Sports Technologies, San Diego, CA) | Commerical-grade accelerometer | NR | Yes | 60 sec | NR | NR | NR | NR | 1 |
| **Empatica E4 wristband** (Empatica, Milan, Italy) | Research-grade triaxial accelerometer | < 60 hours/ > 32 hours | NR | NR | ± 2 | 32 | 44 x 40 x 16 | 25 | 1 |
| **Everion** (Biovotion, Zurich, Switzerland) | Research-grade accelerometer | 4 days/ NR | NR | NR | NR | NR | NR | NR | 1 |
| **Fatigue Science Readiband** (Fatigue Science; Vancouver, BC, Canada) | Commercial-grade accelerometer | NR/ 30 days | Yes | 60 sec | NR | NR | NR | NR | 1 |
| **Fibion** (Fibion Inc, Jyväskylä, Finland) | Research-grade triaxial accelerometer | NR | No | NR | NR | NR | 30 x 32 x 10 | 20 | 1 |
| **Fitbit Alta** (Fitbit Inc., San Francisco, CA, USA) | Commercial-grade triaxial accelerometer | < 30 days/ 5 days | Yes | NR | NR | NR | NR | 32 | 7 |
| **Fitbit Blaze** (Fitbit Inc, San Francisco, CA, USA) | Commercial-grade triaxial accelerometer | 7 days/5 days | Yes | 1 sec | NR | NR | 40.13 x 25.4 x 19.05 | 40 | 1 |
| **Fitbit Charge HR** (Fitbit Inc, San Francisco, CA, USA) | Research-grade triaxial accelerometer | 30 days/5 days | Yes | NR | NR | NR | 157.5-193-21 | 30 | 27 |
| **Fitbit Charge 2 (**Fitbit Inc., San Francisco, CA, USA) | Commercial-grade triaxial accelerometer | < 30 days/ 5 days | Yes | 30 sec | NR | NR | 103 x 5 x 225 | 150 | 16 |
| **Fitbit Charge 3 (**Fitbit Inc., San Francisco, CA, USA) | Commercial-grade triaxial accelerometer | < 30 days/ 7 days | Yes | 1 sec, 5 sec | NR | NR | 19.9 x 34.5 x 22.7 | 32 | 1 |
| **Fitbit Flex** (Fitbit Inc, San Francisco, CA, USA) | Commercial-grade triaxial accelerometer | 5 days/7 days | No | NR | NR | NR | 32 × 12 × 10 | 14.6 | 28 |
| **Fitbit Flex 2 (**Fitbit Inc., San Francisco, CA, USA) | Commercial-grade triaxial accelerometer | < 30 days/ 5 days | No | NR | NR | 100 | 31 x 9 x 7 | 23.5 | 1 |
| **Fitbit Force** (Fitbit Inc, San Francisco, CA, USA) | Commercial-grade triaxial accelerometer | NR/ 5 days | Yes | NR | NR | NR | NR | NR | 2 |
| **Fitbit Inspire** (Fitbit Inc, San Francisco, CA, USA) | Commercial-grade triaxial accelerometer | NR / 5 days | Yes | NR | NR | NR | NR | NR | 1 |
| **Fitbit Ionic** (Fitbit Inc, San Francisco, CA, USA) | Commercial-grade triaxial accelerometer | NR / 5 days | Yes | NR | NR | NR | NR | NR | 1 |
| **Fitbit One (**Fitbit Inc., San Francisco, CA, USA) | Commercial-grade triaxial accelerometer | < 23 days/ 10 days | Yes | 60 sec | NR | NR | 19.3 x 48 x 9.65 | 8 | 28 |
| **Fitbit Surge (**Fitbit Inc., San Francisco, CA, USA) | Commercial-grade triaxial accelerometer | < 30 days/ 7 days | Yes | 1 sec, 5 sec | NR | NR | NR | 52 | 13 |
| **Fitbit Surge 2** (Fitbit Inc, San Francisco, CA, USA) | Commercial-grade triaxial accelerometer | NR | NR | NR | NR | NR | NR | NR | 1 |
| **Fitbit Ultra (**Fitbit Inc., San Francisco, CA, USA) | Commercial-grade triaxial accelerometer | 4 days | Yes | NR | NR | NR | 50.8 x 12.7 x 9.5 | 45.36 | 6 |
| **Fitbit Versa (**Fitbit Inc., San Francisco, CA, USA) | Commercial-grade triaxial accelerometer | 2.5 GB/ 4 days | Yes | NR | NR | NR | 39 x 39 x 11 | 38 | 1 |
| **Fitbit Zip** (Fitbit Inc, San Francisco, CA, USA) | Commercial-grade triaxial accelerometer | 7 days/6 Months | Yes | NR | NR | NR | 28 x 35.5 x 9.65 | 8 | 20 |
| **Flyfit** (Flyfit Inc., San Francisco, CA, USA) | NR | NR / 5-7 days | No | NR | NR | NR | 31 x 19 x 10 | < 100 | 1 |
| **Freestyle Pacer Pro** ((Freestyle Brands, Carrollton, TX, USA) | Commercial-grade pedometer | NR | Yes | NR | NR | NR | NR | NR | 2 |
| **FS-750** (Estera Corporation, Saitama, Japan) | Research-grade triaxial accelerometer | 40 days / 6 months | NR | 2 min | NR | NR | 75 x 33.5 x 10,8 | 26 | 1 |
| **G-Sensor 2026** (Perth, Australia) | Commercial-grade pedometer | NR | Yes | NR | NR | NR | NR | NR | 1 |
| **Gaehwiler Electronic** (Gaehwiler Electronic, Hombrechtikon, Switzerland) | Research-grade triaxial accelerometer | NR | NR | 60 sec | NR | NR | NR | NR | 2 |
| **Garmin 235** (Garmin, Schaffhausen, Switzerland) | Commercial-grade triaxial accelerometer | NR / 9 days | Yes | NR | NR | NR | 40 x 45 x 11.7 | 41.3 | 1 |
| **Garmin Fenix 3** (Garmin, Schaffhausen, Switzerland) | Commercial-grade triaxial accelerometer | 26 MB / 50 hours – 6 weeks | Yes | NR | NR | NR | 51 x 51 x 15.5 | 82 | 1 |
| **Garmin Fenix 5S** (Garmin, Schaffhausen, Switzerland) | Commercial-grade triaxial accelerometer | 64 MB / 9 days | Yes | 60 sec | NR | NR | 42 x 42 x 15 | 67 | 1 |
| **Garmin Forerunner 225** (Garmin, Schaffhausen, Switzerland) | Commercial-grade triaxial accelerometer | NR / 10 days – 4 weeks | Yes | NR | NR | NR | 287 x 48 x 16 | 54 | 1 |
| **Garmin Forerunner 405CX** (Garmin, Schaffhausen, Switzerland) | Commercial-grade triaxial accelerometer | NR / 8 hours – 2 weeks | Yes | NR | NR | NR | 48 x 71 x 16 | 60 | 1 |
| **Garmin Forerunner 735XT** (Garmin, Schaffhausen, Switzerland) | Commercial-grade triaxial accelerometer | NR / 14 hours – 11 days | Yes | NR | NR | NR | 44.5 x 44.5 x 11.9 | 40.2 | 1 |
| **Garmin Forerunner 920XT** (Garmin, Schaffhausen, Switzerland) | Commercial-grade triaxial accelerometer | 32 MB / 24 hours – 4 months | Yes | NR | NR | NR | 44 x 55 x 12.7 | 61 | 3 |
| **Garmin Vovoactive** (Garmin, Schaffhausen, Switzerland) | Commercial-grade triaxial accelerometer | 7-14 days/7 hours with GPS, 3 weeks without | Yes | NR | NR | NR | 43.8 x 38.5 x 8 | 38 | 2 |
| **Garmin Vovoactive 4s** (Garmin, Schaffhausen, Switzerland) | Commercial-grade triaxial accelerometer | NR/7 days | Yes | NR | NR | NR | 40 x 40 x 12.7 | 40 | 1 |
| **Garmin Vivofit 1** (Garmin, Schaffhausen, Switzerland) | Commercial-grade triaxial accelerometer | > 1 year | Yes | NR | NR | NR | 25.5 x 10 | 25.5 | 19 |
| **Garmin Vivofit 2** (Garmin, Schaffhausen, Switzerland) | Commercial grade triaxial accelerometer | NR/1 year | Yes | NR | NR | NR | 21 x 10.5 x 120 | 25.5 | 6 |
| **Garmin Vivofit 3** (Garmin, Schaffhausen, Switzerland) | Commercial-grade triaxial accelerometer | 1 year | Yes | NR | NR | NR | 10 x 10 | 26-28 | 2 |
| **Garmin Vivofit 4** (Garmin, Schaffhausen, Switzerland) | Commercial-grade triaxial accelerometer | 4 weeks/>1 year | Yes | NR | NR | NR | 19 x 9.4 | 25 – 25.5 | 1 |
| **Garmin Vivomove HR** (Garmin, Schaffhausen, Switzerland) | Commercial-grade triaxial accelerometer | 14 days/5-14 days | Yes | NR | NR | NR | 43 x 43 x 11.6 | 40.8 – 56.5 | 1 |
| **Garmin Vivosmart** (Garmin, Schaffhausen, Switzerland) | Commercial grade triaxial accelerometer | 7 days | Yes | NR | NR | NR | 140 –200 | 19 | 7 |
| **Garmin Vivosmart HR+** (Garmin, Schaffhausen, Switzerland) | Commercial-grade triaxial accelerometer | 7 days | Yes | NR | NR | NR | 21 x 15 | NR | 7 |
| **Garmin Vivosmart 3** (Garmin, Schaffhausen, Switzerland) | Commercial-grade triaxial accelerometer | 14 days /5 days | Yes | NR | NR | NR | 197 x 18.5 x 9.8 | 20.4 | 2 |
| **Garmin Vivosport** (Garmin, Schaffhausen, Switzerland) | Commercial-grade triaxial accelerometer | 7-14 days/8 hours with GPS, 7 days without | Yes | NR | NR | NR | 122 x 21 x 10.9 | 24.1 | 1 |
| **GENEActiv** (ActivInsights Ltd., Cambridgeshire, United Kingdom) | Commercial-grade triaxial accelerometer | 500 MB/45 days (at 10 Hz) | No | NR | ± 8 | 1000 | 43 x 40 x 13 | 16 | 14 |
| **Geonaute ONStep 400** (Decathlon France S.A.S, Villeneuve d’Ascq, France) | Commercial-grade pedometer | 7 days/NR | Yes | NR | NR | NR | NR | NR | 2 |
| **Gopher FITStep Pro** (Gopher, Ovatonna, MN, USA) | Commercial-grade pedometer | NR | Yes | NR | NR | NR | NR | NR | 1 |
| **Health Patch** (Vital Connect, CA, USA) | Commercial-grade device | NR | No | NR | NR | NR | NR | NR | 1 |
| **Hidalgo EQ02** (Equivital, Cambridge, UK) | Research-grade triaxial accelerometer | 8 GB/48 hours | No | NR | NR | 256 | NR | NR | 1 |
| **HR + M instrument** (NR) | NR | 8 KB/6 months | NR | 1 min | NR | NR | 145 x 30 x 8 | 100 | 1 |
| **Huami Arc** (Huami Inc, Hefei, China) | Commercial-grade accelerometer | 8 MB/20 days | Yes | NR | NR | NR | 245 x 19 x 11 | 20 | 1 |
| **IC Sensor Model 3031** (NR) | Research-grade accelerometer | NR | No | NR | 1 | 16 | 20 x 20 x 2 | 4 | 1 |
| **Intelligent Device for Energy Expenditure and Activity (IDEEA)** (Minisun LLC, Fresno, CA, USA) | Research-grade device | 200 Mb/48 hours | No | NR | NR | NR | 70 x 44 x 18 | 59 | 9 |
| **iHealth activity monitor** (iHealth Labs Inc, Mountain View, CA, USA) | Commercial-grade pedometer | NR/7 days | Yes | NR | NR | NR | NR | NR | 2 |
| **ikcal** (Teltronic AG, Biberist, Switzerland) | Research-grade biaxial accelerometer | NR/NR | NR | NR | NR | NR | NR | NR | 1 |
| **Jabra Sport Pulse Wireless Earbuds** (GN Audio, Copenhagen, Denmark) | Commercial-grade device | NR | NR | NR | NR | NR | 18 x 21 x 32 | 16 | 1 |
| **Jawbone UP** (Jawbone, San Francisco, California, USA) | Commercial-grade triaxial accelerometer | 9 month/10 days | N | NR | NR | NR | 14.0 x 15.5 (small) | 19-23 | 10 |
| **Jawbone UP2** (Jawbone, San Francisco, California, USA) | Commercial-grade triaxial accelerometer | NR/10 days | NR | 60 sec | NR | NR | NR | NR | 5 |
| **Jawbone UP3** (Jawbone, San Francisco, California, USA) | Commercial-grade triaxial accelerometer | /7 days | No | 30 sec | NR | NR | 140 x 90 | 29 | 7 |
| **Jawbone UP24** (Jawbone, San Francisco, California, USA) | Commercial-grade triaxial accelerometer | 7 days | No |  |  |  | 66–81 × 50–56 | 19-23 | 9 |
| **Jawbone UP Move** (Jawbone, San Francisco, California, USA) | Commercial-grade triaxial accelerometer | NR/6 months | No | NR | NR | NR | 28 x 28 x 10 | 6.8 | 6 |
| **Kellogg's* Special K* Step Counters** (Kellogg´s, Battle Creek, MI, USA) | Commercial-grade pedometer | NR | Yes | NR | NR | NR | 64 x 48 x 22 | 21 | 1 |
| **Kenz e-style2** (Suzuken, Co., Ltd., Nagoya, Japan) | Research-grade triaxial accelerometer | NR | NR | NR | NR | NR | 63 x 36 x 14 | 22 | 2 |
| **Kenz Lifecorder** **EX** (Suzuken, Co., Ltd., Nagoya, Japan) | Research-grade triaxial accelerometer | 200 days/6 months | Yes | 5 sec - 10 min | 0.06-1.94 | 32 | 72.5 x 41.5 x 27.5 | 60 | 13 |
| **Leaf Health Tracker** (Bellabeat, San Francisco, CA, USA) | Commercial-grade triaxial accelerometer | NR | No | NR | NR | NR | NR | 16.5 | 3 |
| **Lifecorder PLUS** (Suzuken, Co., Ltd., Nagoya, Japan) | Research-grade uniaxial accelerometer | 7 days/2 months | Yes | NR | NR | NR | 75 x 42 x 29.1 | 48 | 1 |
| **Lifesource XL-18 (**A&D Medical, Toronto, ON, Canada**)** | Commercial-grade triaxial accelerometer | 14 days/1 year | Yes | NR | NR | NR | 76 x 34 x 11 | 28.35 | 1 |
| **LIS3LV02DQ** (ST-Microelectronics, Geneva, Switzerland) | Research-grade triaxial accelerometer | 4 GB/NR | No | 10 sec | ±6 | 32 | 80 x 50 x 20 | 60 | 1 |
| **Lumoback** (Lumo Bodytech Inc., Mountain View, CA, USA) | Commercial-grade triaxial accelerometer | NR/120-168 h | No | NR | NR | NR | 415 x 100 x 8 | 25 | 2 |
| **Metria IH1 (**Vandrico Inc., Vancouver, Canada) | Commercial grade triaxial accelerometer | 28 days/1 week | No | NR | NR | NR | NR | NR | 3 |
| **Microsoft Band** (Microsoft, Inc., Redmond, WA, USA) | Commercial-grade triaxial accelerometer | 64 MB/48 hours | Yes | NR | NR | NR | NR | 60 | 6 |
| **Microsoft Band 2** (Microsoft, Inc., Redmond, WA, USA) | Commercial-grade triaxial accelerometer | NR/48 hours | Yes | NR | NR | NR | NR | NR | 1 |
| **Mio FUSE** (Mio, Vancouver, Canada) | Commercial-grade triaxial accelerometer | NR/24 hours | Yes | NR | NR | NR | 30 x 259 x 16 | 39.7 | 1 |
| **Mio Slice** (Mio, Vancouver, Canada) | Commercial-grade biaxial accelerometer | 7 days / 5 days | Yes | NR | NR | NR | 13.5 x 20.8 x 24 | 29 | 1 |
| **Misfit Flash** (Misfit Wearables, Burlingame, CA, USA) | Commercial-grade triaxial accelerometer | NR / 6 months | No | NR | NR | NR | 28.5 x 8.0 x 28.5 | 6 | 1 |
| **Misfit Shine (**Fossil Group, Richardson, TX, USA) | Commercial-grade triaxial accelerometer | 4 months | Yes | NR | NR | NR | 27.5 x 3.3 x 27.5 | 9.4 | 10 |
| **Motionlogger Microwatch Actigraph** (Ambulatory Monitoring Inc., Ardsley, New York, USA) | Research-grade triaxial accelerometer | NR | Yes | 30 sec | NR | NR | NR | NR | 2 |
| **MotionSense HRV** (MD2K Center, Memphis, USA) | Research-grade triaxial accelerometer | NR | No | NR | 2 | 16 | NR | NR | 1 |
| **MotionWatch 8** (CamNTech, Cambridge, UK) | Research-grade triaxial accelerometer | 4 Mbits/3 months | No | 1, 2, 5, 15, 30, 60 sec | 0.01-8 | 3-11 | 28.2 x 9.4 x 36 | 9.1 | 1 |
| **Mother** (Sen.se, Paris, France) | Research-grade triaxial accelerometer | NR | No | NR | 2 | 25 | 50 x 22 x 4 | 6 | 1 |
| **Moto 360** (Motorola, Chicago, IL, USA) | Commercial-grade trixaialer accelerometer | 8 GB/< 1 day | Yes | NR | NR | NR | 42.8 x 42.8 x 11.7 | 52 | 2 |
| **MovBand Model 2** (Movband, LLC, Brecksville, OH, USA) | Commercial-grade triaxial accelerometer | 40 days | Yes | NR | 2-8 | NR | NR | NR | 1 |
| **MOVEBAND** (DHS Group, Houston, TX, USA) | Commercial-grade device | NR | Yes | NR | NR | NR | NR | NR | 1 |
| **Move II (movisens GmbH, Karlsruhe, Germany)** | Research-grade triaxial accelerometer | 2 months/7 days | No | 10, 30, 60 sec | 8 | 128 | 53 x 30 x 20 | 16,1 | 2 |
| **Move 3 (movisens GmbH, Karlsruhe, Germany)** | Research-grade triaxial accelerometer | 2 months/7 days | No | 10, 30, 60 sec | 8 | 64 | 62.3 x 38.6 x 11.5 | 25 | 1 |
| **Move 4 (movisens GmbH, Karlsruhe, Germany)** | Research-grade triaxial accelerometer | 4 GB/7 days | No | 10, 30, 60 sec | 16 | 64 | 62.3 x 38.6 x 11.5 | 25 | 1 |
| **MOX Activity Logger** (Maastricht Instruments, Maastricht, NL) | Research-grade triaxial accelerometer | 1.5 GB/7 days | No | 10, 30, 60 sec | 8 | 25-100 | 35 x 35 x 10 | 11 | 2 |
| **Multi-sensor board** (NR) | Research-grade triaxial accelerometer | 12 hours/8 hours | No | NR | NR | ~550 | 62 x 79.7 x 21.5 | 25 | 1 |
| **Muvone** (Secmotic, Seville, Spain) | Commercial-grade triaxial accelerometer | NR | NR | NR | NR | 40 | 32 x 32 x 11 | 9 | 1 |
| **myCanadian watch** (CurAegis Technologies, Rochester, NY, USA) | Commercial-grade triaxial accelerometer | NR | Yes | 30 sec | 4 | 25 | NR | NR | 1 |
| **MyWellness Key** (Technogym, Gambettola, Italy) | Commercial-grade uniaxial accelerometer | 49-59 days | Yes | NR | 0.06 - 12 | 16 | 85 x 20 x 7 | 18.7 | 2 |
| **New Lifestyle NL-800** (New Lifestyles, Inc., Lee´s Summit, MO, USA) | Commercial-grade uniaxial accelerometer | 7 days/18 months | Yes | NR | NR | NR | 63.5 x 38.1 x 22.2 | 31.2 | 1 |
| **New Lifestyle NL-1000** (New Lifestyles, Inc., Lee´s Summit, MO, USA) | Commercial-grade uniaxial accelerometer | 7 days/18 months | Yes | 4 sec | NR | NR | 63.5 x 38.1 x 22.2 | 31.2 | 7 |
| **New Lifestyle NL-2000** (New Lifestyles, Inc., Lee´s Summit, MO, USA) | Commercial-grade triaxial accelerometer | 7-14 days/ g months | Yes | NR | NR | NR | 63.5 x 38.1 x 12.7 | 25.5 | 14 |
| **Nike + Fuelband SE** (Nike Inc., Beaverton, OR, USA) | Commercial-grade triaxial accelerometer | 4 days | Yes | NR | NR | NR | 147–197 x 19 | 27-32 | 13 |
| **Nokia GO** (Nokia Corporation, Espoo, Finland) | Commercial-grade triaxial accelerometer | 8 months | Yes | NR | NR | NR | 34.5 x 9.4 | 9 | 2 |
| **Omron Active Style Pro** (Omron Healthcare, Inc., Bannockburn, IL, USA) | Commercial-grade triaxial accelerometer | NR | Yes | NR | 3 | 32 | 80 x 20 x 50 | 61 | 3 |
| **Omron B1** (Omron Healthcare, Inc., Bannockburn, IL, USA) | Commercial-grade device | NR | NR | NR | NR | NR | NR | NR | 1 |
| **Omron CaloriScan** (Omron Healthcare, Inc., Bannockburn, IL, USA) | Commercial-grade pedometer | 7 days/3 months | Yes | NR | NR | NR | 33 x 7 | 27 | 1 |
| **Omron HF-100** (Omron Healthcare, Inc., Bannockburn, IL, USA) | Commercial-grade pedometer | NR | Yes | NR | NR | NR | NR | NR | 2 |
| **Omron HJ-105** (Omron Healthcare, Inc., Vernon Hills, IL, USA) | Commercial-grade device | 7 days/ 3 months | Yes | NR | NR | NR | 63.5 x 38.1 x 25.4 | 24 | 4 |
| **Omron HJ-109** (Omron Healthcare, Inc., Bannockburn, IL, USA) | Commercial-grade pedometer | 7 days/1 year | Yes | NR | NR | NR | 63.5 x 36.3 x 23 | 24 | 1 |
| **Omron HJ-112** (Omron Healthcare, Inc., Bannockburn, IL, USA) | Commercial-grade pedometer | 7 days/6 months | Yes | NR | NR | NR | 47 x 66 x 16 | NR | 5 |
| **Omron HJ-113** (Omron Healthcare, Inc., Bannockburn, IL, USA) | Commercial-grade pedometer | 7 days/6 months | Yes | NR | NR | NR | 47 x 73 x 16 | 37 | 3 |
| **Omron HJ-151** (Omron Healthcare, Inc., Bannockburn, IL, USA) | Commercial-grade uniaxial accelerometer | 7 days (on instrument) /1 year | Yes | NR | NR | NR | 53 x 25.8 | 35 | 2 |
| **Omron HJ-203** (Omron Healthcare, Inc., Vernon Hills, IL, USA) | Commercial-grade biaxial accelerometer | 7 days/ 1.5 yrs | Yes | NR | NR | NR | 35.5 x 68.5 x 11.0 | 19 | 2 |
| **Omron HJ-301** (Omron Healthcare, Inc., Bannockburn, IL, USA) | Commercial-grade accelerometer | 7 days/6 months | Yes | NR | NR | NR | 75.5 x 33.5 x 11.8 | 28 | 1 |
| **Omron HJ-303** (Omron Healthcare, Inc., Bannockburn, IL, USA) | Commercial-grade triaxial accelerometer | 7 days/6 months | Yes | NR | NR | NR | 75.5 x 33.5 x 11.8 | 28 | 2 |
| **Omron HJ-304** (Omron Healthcare, Inc., Bannockburn, IL, USA) | Commercial-grade pedometer | 7 days/6 months | Yes | NR | NR | NR | 75.5 x 33.5 x 11.8 | 28 | 1 |
| **Omron HJ-320** (Omron Healthcare, Inc., Bannockburn, IL, USA) | Commercial-grade triaxial accelerometer | 7 days/6 months | Yes | NR | NR | NR | 78 x 33 x 10 | 25 | 1 |
| **Omron HJ-321** (Omron Healthcare, Inc., Bannockburn, IL, USA) | Commercial-grade triaxial accelerometer | 7 days/6 months | Yes | NR | NR | NR | 75 x 32 x 8 | NR | 4 |
| **Omron HJ-322** (Omron Healthcare, Inc., Bannockburn, IL, USA) | Commercial-grade pedometer | 7 days/6 months | Yes | NR | NR | NR | NR | 27 | 1 |
| **Omron Active Style Pro HJA-350IT** (Omron Healthcare, Inc., Bannockburn, IL, USA) | Commercial-grade triaxial accelerometer | 4 GB/ NR | Yes | 10-60 sec | ± 6 | 32 | 74x 46 x 34 | 60 | 2 |
| **Omron Walking Style Pro HJ-700IT** (Omron Healthcare, Inc., Bannockburn, IL, USA) | Commercial-grade pedometer | NR/6 months | Yes | NR | NR | NR | 47 x 16 x 73 | 37 | 1 |
| **Omron Walking Style Pro HJ-701IT** (Omron Healthcare, Inc., Bannockburn, IL, USA) | Commercial-grade pedometer | NR/NR | Yes | NR | NR | NR | 52 x 74 x 15 | 50 | 1 |
| **Omron Walking Style Pro HJ-720IT** (Omron Healthcare, Inc., Bannockburn, IL, USA) | Commercial-grade biaxial accelerometer | 35 days/ 6 months | Yes | NR | NR | NR | 47 x 20 x 71 | NR | 16 |
| **Omron CaloriScan HJ-306** (Omron Healthcare, Inc., Vernon Hills, IL, USA) | Commercial-grade triaxial accelerometer | 7 days/ 6 months | Yes | NR | NR | NR | 78 x 33 x 10 | 25 | 1 |
| **Oregon Scientific PE316CA** (Oregon Scientific, Tualatin, OR, USA) | Commercial-grade pedometer | NR | Yes | NR | NR | NR | 32 x 41 x 63 | 28 | 2 |
| **Össur patient activity monitor** (Össur, Reykjavík, Iceland) | Commercial-grade biaxial accelerometer | 7 days/NR | NR | NR | NR | NR | 85 x 38 x 32 | 50 | 1 |
| **Oura ring** (Oura Health Ltd., Oulu, Finland) | Commercial-grade triaxial accelerometer | 3 weeks/4-7 days | No | NR | NR | NR | 7.9 x 2.55 | 4-6 | 1 |
| **PADIS 2.0** (Axiamo, Biel/Bienne, Switzerland) | Commercial-grade accelerometer | NR/7 days | No | 2-60 sec | NR | 64 | NR | NR | 1 |
| **PALlite** (PAL Technologies Limited, Glasgow, UK) | Research-grade accelerometer | 10 days/NR | NR | NR | NR | NR | 50 x 35 x 7 | 20 | 1 |
| **PALlite 3c** (PAL Technologies Limited, Glasgow, UK) | Research-grade accelerometer | NR | NR | NR | NR | NR | NR | NR | 1 |
| **Pebble Steel Watch** (Pebble Technology Corp., Redwood City, CA, USA) | Commercial-grade triaxial accelerometer | NR/3-6 days | Yes | NR | NR | NR | 34 x 46 x 10.5 | 56 | 3 |
| **Personal Activity Monitor** (PAM B.V. Doorwerth, Netherlands) | Commercial-grade uniaxial accelerometer | 3 months/NR | Yes | NR | NR | NR | 58 x 42 x 13 | 28 | 1 |
| **Philips Health Watch** (Philips, Stamford, CT, USA) | Commercial-grade triaxial accelerometer | 4 days/ 7 days | Yes | NR | NR | NR | 21 x 15 | 31/33 | 2 |
| **Physilog** (GaitUp, Switzerland) | Research-grade triaxaial accelerometer | NR/NR | No | NR | ± 11 | 100 | NR | NR | 2 |
| **PiezoRx** (StepsCount, Ontario, Canada) | Commercial-grade pedometer | 33 days | Yes | NR | NR | NR | NR | NR | 2 |
| **Piezo Step MV** (StepsCount, Ontario, Canada) | Commercial-grade pedometer | NR | NR | NR | NR | NR | 56 x 32 x 10 | 20 | 1 |
| **Polar A300** (Polar Electro Oy, Kimpel, Finland) | Commercial-grade triaxial accelerometer | 60 hours/200 hours | Yes | NR | NR | NR | 115 x 95 x 74.9 | 48 | 2 |
| **Polar A360** (Polar Electro Oy, Kimpel, Finland) | Commercial-grade triaxial accelerometer | 60 hours/NR | Yes | NR | NR | NR | NR | 37.3 | 5 |
| **Polar F6** (Polar Electro Oy, Kimpel, Finland) | Commercial-grade-device | 3 months/1.5 years | Yes | NR | NR | NR | NR | NR | 1 |
| **Polar FT7** (Polar Electro Oy, Kimpel, Finland) | Commercial-grade-device | NR | Yes | NR | NR | NR | NR | NR | 1 |
| **Polar H7** (Polar Electro Oy, Kimpel, Finland) | Commercial accelerometer | 150 hours/NA | No | NR | NR | NR | 35 x 10 x 64 | 25 | 2 |
| **Polar Loop** (Polar Electro Oy, Kimpel, Finland) | Commercial-grade triaxial accelerometer | 4 Mb/6 days | Yes | NR | NR | NR | 145–240 × 20 | 38 | 6 |
| **Polar M200** (Polar Electro Oy, Kimpel, Finland) | Commercial-grade traxial accelerometer | NR/8 h with GPS, 24 days without | Yes | NR | NR | NR | NR | 40 | 1 |
| **Polar M400** (Polar Electro Oy, Kimpel, Finland) | Commercial-grade triaxial accelerometer | NR/8 h with GPS, 24 days without | Yes | NR | NR | NR | 30 x 12 x 35 | 110 | 1 |
| **Polar S410** (Polar Electro Oy, Kimpel, Finland) | Commercial-grade triaxial accelerometer | NR | Yes | NR | NR | NR | NR | 31.8 | 1 |
| **Polar S810i** (Polar Electro Oy, Kimpel, Finland) | Commercial-grade accelerometer | NR | Yes | 5; 15; 60 sec | NR | NR | NR | NR | 1 |
| **Polar V800** (Polar Electro Oy, Kimpel, Finland) | Commercial-grade triaxial accelerometer | 60 hours/30 days | Yes | NR | NR | NR | 37 x 56 x 12.7 | 79 | 2 |
| **Polar Vantage M** (Polar Electro Oy, Kimpel, Finland) | Commercial-grade triaxial accelerometer | 32 MB/7 days | Yes | NR | NR | NR | NR | 45 | 2 |
| **Polar Vantage XL** (Polar Electro Oy, Kimpel, Finland) | Commercial-grade triaxial accelerometer | NR | Yes | NR | NR | NR | NR | NR | 1 |
| **Positional Activity Logger 2** (Gorman ProMed, Melbourne, Australia) | Research-grade biaxial accelerometer | NR | No | 3 sec | NR | 10 | 80 x 40 x 15 | NR | 1 |
| **Prosthetic Activity Monitor** (Össur, Reykjavík, Iceland) | Research-grade biaxial accelerometer | 3 days/NR | Yes | 60 sec | NR | NR | NR | NR | 1 |
| **PulseOn** (Oy, Espoo, Finland) | Commercial-grade device | NR | No | NR | NR | NR | NR | 29 | 2 |
| **Qualcomm Toq** (Qualcomm, San Diego, California, USA) | Commercial-grade accelerometer | NR / 5 days | Yes | NR | NR | NR | NR | 91 | 1 |
| **Quattrolter** (Novacor, France) | Commerical-grade biaxial accelerometer | NR | Yes | NR | NR | NR | NR | NR | 1 |
| **RT 6** (Stayhealthy, Inc., Monrovia, CA, USA) | Research-grade triaxial accelerometer | NR | NR | 1 sec | ± 4 | 80 | NR | NR | 1 |
| **RT3** (Stayhealthy, Inc., Monrovia, CA, USA) | Research-grade triaxial accelerometer | 21 days/ 8.5 days | No | 1 - 60 sec | ± 250 | 0.7 - 5000 | 71 x 56 x 28 | 65.2 | 7 |
| **Samsung Galaxy Watch Active** (Samsung Electro-Mechanics, Seoul, South Korea) | Commercial-grade accelerometer | NR/45 hours | Yes | NR | NR | NR | 39.5 x 39.5 x 10.5 | 25 | 1 |
| **Samsung Gear 1** (Samsung Electro-Mechanics, Seoul, South Korea) | Commercial-grade accelerometer | 4 GB/<1 day | Yes | NR | NR | NR | NR | NR | 1 |
| **Samsung Gear 2** (Samsung Electro-Mechanics, Seoul, South Korea) | Commercial-grade accelerometer | 4 GB/<1 day | Yes | NR | NR | NR | 36.9 x 58.4 x 10 | 68 | 5 |
| **Samsung Gear Fit** (Samsung Electro-Mechanics, Seoul, South Korea) | Commercial-grade accelerometer | NR/1-2 days | Yes | NR | NR | NR | 23.4 × 57.4 × 11.95 | 27 | 1 |
| **Samsung Gear Fit2** (Samsung Group, Seoul, South Korea) | Commercial-grade triaxial accelerometer | 32 MB/ 15 days | Yes | NR | NR | NR | 46.6 x 1.6 x 11.1 | 21 | 1 |
| **Samsung Gear S** (Samsung Electro-Mechanics, Seoul, South Korea) | Commercial-grade triaxial accelerometer | 4 GB/< 1 day | Yes | NR | ± 2 | 100 | 58 x 40 x 12 | 67 | 3 |
| **Samsung Gear S3** (Samsung Electro-Mechanics, Seoul, South Korea) | Commercial-grade triaxial accelerometer | 4 GB/39h | Yes | NR | NR | NR | 12.9 x 49.1 x 46 | 63 | 1 |
| **Sartorio Xelometer** (Sartorio OY) | Research-grade triaxial accelerometer | NR/21 days | NR | NR | ± 8 | 100 | NR | NR | 1 |
| **SENS Motion System** (SENS Innovation ApS, Copenhagen, Denmark**)** | Commercial-grade triaxial accelerometer | 14 days/20 weeks | No | NR | ± 4 | 12.5 | 50 x 21 x 5 | 8 | 1 |
| **SenseWear Pro** (BodyMedia, Inc., Pittsburgh, PA) | Commercial-grade biaxial accelerometer | 5 days /4 days | No | NR | NR | 32 | 85.3 x 53.4 x 19.5 | 85 | 37 |
| **SenseWear Pro 2** (BodyMedia, Inc., Pittsburgh, PA) | Commercial-grade biaxial accelerometer | NR/2 weeks | No | 60 sec | NR | 32 | NR | NR | 7 |
| **SenseWear Pro 3** (BodyMedia, Pittsburgh, PA, USA) | Commercial-grade biaxial accelerometer | NR/2 weeks | No | NR | ± 2 | NR | 85 x 53 x 19 | 79 | 18 |
| **SenseWear Mini** (BodyMedia, Pittsburgh, PA, USA) | Commercial-grade triaxial accelerometer | NR | No | NR | ± 1 | 1 | 86 x 60 x 27 | 45.36 | 16 |
| **Silva pedometer** (Silva, Sweden) | Commercial-grade pedometer | NR/1 year | Yes | NR | NR | NR | NR | NR | 1 |
| **SmartHealth** (Smart Health, Fremont, CA, USA) | Commercial-grade pedometer | 1 day/NR | Yes | NR | NR | NR | NR | NR | 1 |
| **SmartLAB walk +** (HMM Diagnostics GmbH, Dossenheim, Germany) | Commercial-grade triaxial accelerometer | 7 days memory | Yes | NR | NR | NR | 70 x 37 x 10.5 | 30 | 1 |
| **SOMNOwatch** (Somnomedics, Germany) | Research-grade triaxial acclerometer | 8 MB/NR | No | 1 – 120 sec | 8.7 | 256 | NR | NR | 2 |
| **Sony SmartBand** (Sony Computer Entertainment Inc., San Mateo, CA, USA) | Commercial-grade accelerometer | 1 GB/2–4days | No | NR | NR | NR | NR | 60 | 1 |
| **Spire Activity Tracker** (Spire, San Francisco, CA) | Commercial-grade device | NR/7 days | No | NR | NR | NR | 32 x 44 x 14 | NR | 1 |
| **Sportline 330** (E&B Giftware LLC, Hazleton, PA, USA) | Commercial-grade pedometer | NR/NR | Yes | NR | NR | NR | 19.7 x 5.9 x 27.6 | 90.7 | 4 |
| **Sportline 340 Strider** (E&B Giftware LLC, Hazleton, PA, USA) | Commercial-grade pedometer | NR/NR | Yes | NR | NR | NR | 127 x 25 x 216 | 45 | 1 |
| **Sportline 345** (E&B Giftware LLC, Hazleton, PA, USA) | Commercial-grade pedometer | NR/NR | Yes | NR | NR | NR | 78 x 43 x 17 | 45 | 2 |
| **Sportline Traq** (E&B Giftware LLC, Hazleton, PA, USA) | Commercial-grade pedometer | 7 days/NR | Yes | NR | NR | NR | NR | NR | 1 |
| **SportBrain iStep X1 (**Sportbrain Holdings Inc., Naples, FL, USA) | Commercial-grade pedometer | NR/NR | Yes | 60 sec | NR | NR | 226 x 17 x 23 | NR | 1 |
| **Step-Keeper HSB-SKM** (NR, Japan) | Commercial-grade pedometer | NR/NR | No | NR | NR | NR | NR | NR | 1 |
| **Stepcount StepMX** (StepsCount, Deep River, ON, USA) | Commercial-grade pedometer | NR/NR | NR | NR | NR | NR | 56 x 32 x 10 | 20 | 2 |
| **Step-N-Tune** (Kinergy Electronics Co., Ltd, Shenzhen, Hong Kong, China) | Commercial-grade pedometer | NR/NR | Yes | NR | NR | NR | NR | NR | 1 |
| **Stepwatch Activity Monitor** (Cyma Corp., Mountlake Terrace, WA, USA) | Research-grade biaxial accelerometer | NR/NR | No | NR | NR | NR | 50 x 15 x 65 | 65 | 21 |
| **Stepwatch 3 Activity Monitor** (Cyma Corp., Mountlake Terrace, WA, USA) | Research-grade triaxial accelerometer | 32 KB/7 years | No | NR | NR | NR | 75 x 50 x 20 | 38 | 7 |
| **Stryd Power Meter** (Stryd, Boulder, CO, USA) | Commercial-grade device | NR/1 month | No | NR | NR | NR | NR | 10 | 1 |
| **Suunto Ambit2** (Suunto Oy, Vantaa, Finland) | Commercial-grade device | NR/30 days | Yes | 1, 10 sec | NR | NR | 50 x 50 x 18.1 | 89 | 1 |
| **Suunto HR** (Suunto Oy, Vantaa, Finland) | Commercial-grade device | NR/NR | No | NR | NR | NR | NR |  | 1 |
| **Suunto Sport** (Suunto Oy, Vantaa, Finland) | Commercial-grade accelerometer | NR/10-80 hours | Yes | 1, 10 sec | NR | NR | 50 x 50 x 16.8 | 75 | 1 |
| **Suunto Trainer** (Suunto Oy, Vantaa, Finland) | Commercial-grade accelerometer | NR/10-30 hours | Yes | NR | NR | NR | 46 x 46 x 15.7 | 56 | 1 |
| **Tanita AM-160** (Tanita Europe BV, Amsterdam, Netherlands) | Commercial-grade triaxial accelerometer | 7 days/NR | Yes | NR | NR | NR | 75 x 14 x 35 | 26 | 1 |
| **Tanita Calorism Smart** (Tanita Europe BV, Amsterdam, Netherlands) | Commercial-grade triaxial accelerometer | NR/NR | Yes | NR | NR | NR | 55 x 11.8 x 29 | 23 | 1 |
| **Tanita FB727** (Tanita Europe BV, Amsterdam, Netherlands) | Commercial-grade pedometer | NR/NR | NR | NR | NR | NR | NR | NR | 1 |
| **THIM** (Re-Timer Pty Ltd, Lonsdale, Australia) | Commercial-grade triaxial accelerometer | NR | No | 30 sec | NR | NR | NR | NR | 1 |
| **Tom Tom Cardio** (TomTom, Amsterdam, Netherlands) | Commercial-grade accelerometer | NR/10 hours | Yes | NR | NR | NR | NR | 63 | 3 |
| **Tom Tom Touch** (TomTom, Amsterdam, Netherlands) | Commercial-grade accelerometer | 4 MB/5 days | Yes | NR | NR | NR | NR | 10 | 2 |
| **Tom Tom Spark 3** (TomTom, Amsterdam, Netherlands) | Commercial-grade accelerometer | 3 GB/NR | Yes | NR | NR | NR | 22 x 25 x 13.7 | 49 | 1 |
| **Tracmor** (ICSensors 3031–010, Druck Nederland, The Netherlands) | Research-grade triaxial accelerometer | NR | NR | NR | NR | NR | 50 x 30 x 8 | 16 | 2 |
| **Tractivity** (Kineteks Corp., Vancouver, Canada) | Commercial-grade uniaxial accelerometer | NR/NR | No | 60 sec | NR | NR | NR | NR | 1 |
| **T-REX** (Taewoong Medical, Co., Ltd, Gyeonggido, Korea) | Research-grade triaxial accelerometer | NR/NR | No | NR | 6 | 32 | NR | NR | 1 |
| **Tritrac-R3D** (Professional Products, Madison, WI, USA) | Research-grade triaxial accelerometer | 14 days/ NR | No | 1 - 15 min | 0.05 - 6.3 | 0.1 - 3.0 | 120 x 65 x 22 | 170 | 6 |
| **Tritrac T303A** (Professional Products, Madison, WI, USA) | Research-grade triaxial accelerometer | NR | No | 1 min | NR | NR | 108 x 68 x 33 | 170.4 | 1 |
| **USB accelerometer X16-mini** (Gulf Coast Data Concepts, Waveland, MS, USA) | Commercial-grade accelerometer | NR | No | NR | 2 | 40 | 64 x 25 x 13 | 18 | 1 |
| **Verisense** (Shimmer Research Ltd., Dublin, Ireland) | Research-grade triaxial accelerometer | NR/6 months | No | NR | NR | 12.5-1600 | 43 x 35 x 12 | 29.6 | 1 |
| **Vibe Actigraph** (Agartee Technology Inc., Vancouver, Canada) | Commercial-grade accelerometer | NR/NR | Yes | NR | NR | NR | NR | NR | 1 |
| **Vitabit** (VitaBit Software International B.V., Eindhoven, The Netherlands) | Research-grade triaxial accelerometer | NR/30 days | No | 30 sec. | 16 | 33 | 39 x 14 x 8.5 | 4.8 | 1 |
| **Vivago** (Vivago Wellness, Paris, France) | Commercial-grade uniaxial accelerometer | NR | Yes | 60 sec | 4 | 0.5-10 | NR | 24 | 1 |
| **Vivago WristCare** (IST International Security Technology Oy) | Commercial-grade device | NR/2-4 months | No | 10 sec | NR | NR | NR | NR | 1 |
| **Walk4Life Elite** (Walk4Life, Inc., Plainfield, IL, USA) | Commercial-grade pedometer | NR | Yes | NR | NR | NR | NR | NR | 3 |
| **Walk4Life LS 2500** (Walk4Life, Inc., Plainfield, IL, USA) | Commercial-grade pedometer | NR/NR | Yes | NR | NR | NR | NR | NR | 1 |
| **Walk4Life LS 2525** (Walk4Life, Inc., Plainfield, IL, USA) | Commercial-grade pedometer | NR/NR | Yes | NR | NR | NR | NR | NR | 2 |
| **Walk4Life LS 7010** (Walk4Life, Inc., Plainfield, IL, USA) | Commercial-grade pedometer | None/NR | Yes | NR | NR | NR | 57 x 32 x 13 | NR | 1 |
| **Walk4Life Pro** (Walk4Life, Inc., Plainfield, IL, USA) | Commercial-grade pedometer | 7 days/NR | Yes | 1 sec | NR | NR | 52 x 39 x 19 | NR | 1 |
| **Walking Style X** (Omron Healthcare Europe BV, Netherlands) | Commercial-grade pedometer | 7 days/6 months | Yes | NR | NR | NR | 75.5 x 33.5 x 11.8 | 28 | 1 |
| **Watch_PAT100** (AMI, Ambulatory Monitoring Inc, New York, USA) | Commercial-grade uniaxial accelerometer | NR | NR | 30 sec | NR | 100 | NR | NR | 1 |
| **WHOOP Strap** (CB Rank, Greater Boston, New England, USA) | Commercial-grade accelerometer | NR/48 hours | No | 30 sec | NR | NR | NR | 18.4 | 2 |
| **WIMU Pro** (RealTrack Systems, Almeira, Spain) | Commerical accelerometer | 2 GB/4 hours | No | NR | 2-400 | 10-100 | 81 x 45 x 16 | 70 | 1 |
| **Withings Go** (Withings SA, Issy les Moulineaux, France) | Commerical-grade triaxial accelerometer | NR/8 months | Yes | NR | NR | NR | NR | NR | 1 |
| **Withings Pulse O2** (Withings SA, Issy les Moulineaux, France) | Commercial-grade triaxial accelerometer | NR/14 days | Yes | NR | NR | NR | 22 x 8 x 43 | 8 | 9 |
| **Withings Pulse Ox** (Withings SA, Issy les Moulineaux, France) | Commercial-grade triaxial accelerometer | NR/14 days | Yes | NR | NR | NR | 22 x 8 x 43 | 8 | 4 |
| **Withings Steel** (Withings SA, Issy les Moulineaux, France) | Commercial-grade triaxial accelerometer | NR/25 days | Yes | NR | NR | NR | 36 x 18 x 13 | 39 | 1 |
| **Xiaomi Mi Band** (Xiaomi, Beijing, China) | Commercial-grade triaxial accelerometer | 16 MB/60 days | Yes | NR | NR | NR | 37 x 13.6 x 9.9 | 13 | 1 |
| **Xiaomi Mi Band 2** (Xiaomi, Beijing, China) | Commercial-grade triaxial accelerometer | 20 days | Yes | NR | NR | NR | 15.7 x 40.3 x 10.5 | 19 | 6 |
| **Xiaomi Mi Band 3** (Xiaomi, Beijing, China) | Commercial-grade triaxial accelerometer | NR | Yes | NR | NR | NR | 46.9 x 17.9 x 12 | 20 | 3 |
| **Xiaomi Mi Band 4** (Xiaomi, Beijing, China) | Commercial-grade triaxial accelerometer | 16 MB/20 days | Yes | NR | NR | NR | 47 x 21.6 x 10.8 | 22.1 | 1 |
| **Yamax Digiwalker** (Yamasa Tokei Keiki Co., Ltd., Tokyo, Japan) | Commercial-grade pedometer | NR | NR | NR | NR | NR | NR | NR | 5 |
| **Yamax Digiwalker CW-700** (Yamax Corp., Tokyo, Japan) | Commercial-grade pedometer | 2 weeks/3 years | Yes | NR | NR | NR | 61 x 19 x 46 | 38 | 1 |
| **Yamax Digiwalker CW-701** (Yamax Corp., Tokyo, Japan) | Commercial-grade pedometer | 2 weeks/3 years | Yes | NR | NR | NR | 61 x 19 x 46 | 38 | 1 |
| **Yamax Digiwalker DW-200** (Yamasa Tokei Keiki Co., Ltd., Tokyo, Japan) | Commercial-grade pedometer | NR/NR | Yes | NR | NR | NR | NR | NR | 2 |
| **Yamax Digiwalker DW-351** (Yamasa Tokei Keiki Co., Ltd., Tokyo, Japan) | Commercial-grade pedometer | NR/NR | Yes | NR | NR | NR | NR | NR | 1 |
| **Yamax Digiwalker DW-800** (Yamasa Tokei Keiki Co., Ltd., Tokyo, Japan) | Commercial-grade pedometer | NR/NR | Yes | NR | NR | NR | NR | NR | 1 |
| **Yamax Digiwalker SW-200** (Yamasa Tokei Keiki Co., Ltd., Tokyo, Japan) | Commercial-grade pedometer | None/ 3 yrs | Yes | 1, 2, 5 sec | 0.35-0.50 | NR | 50 x 38 x 14 | 21 | 30 |
| **Yamax Digiwalker SW-401** (Yamasa Tokei Keiki Co., Ltd., Tokyo, Japan) | Commercial-grade pedometer | None/ 3 yrs | Yes | NR | NR | NR | 50 x 35 x 15 | 22.7 | 2 |
| **Yamax Digiwalker SW-500** (Yamasa Tokei Keiki Co., Ltd., Tokyo, Japan) | Commercial-grade pedometer | NR/3 yrs | Yes | 1 sec | NR | NR | 50 x 38 x 14 | 21 | 1 |
| **Yamax Digiwalker SW-700** (Yamasa Tokei Keiki Co., Ltd., Tokyo, Japan) | Commercial-grade pedometer | None/3 yrs | Yes | NR | NR | NR | 50 x 38 x 14 | 21 | 4 |
| **Yamax Digiwalker SW-701** (Yamasa Tokei Keiki Co., Ltd., Tokyo, Japan) | Commercial-grade pedometer | None/3 yrs | Yes | NR | 0.35-0.50 | NR | 50 x 38 x 14 | 21 | 13 |
| **Yamax EX510** (Yamasa Watch & Clock Co., Ltd., Japan) | Commercial-grade triaxial accelerometer | 30 days/1 year | Yes | NR | NR | NR | 76 x 33.5 x 10 | 24 | 2 |
| **Yamax EX700** (Yamasa Watch & Clock Co., Ltd., Japan) | Commercial-grade pedometer | NR/NR | Yes | NR | NR | NR | NR | NR | 1 |
| **Yamasa Skeletone** (Yamax Corp., Tokyo, Japan) | Commercial-grade pedometer | NR/NR | Yes | NR | NR | NR | NR | NR | 1 |
| **Yamax Skeletone EM-180** (Yamax Corp., Tokyo, Japan) | Commercial-grade pedometer | NR/ 3 yrs | Yes | NR | NR | NR | 48 x 36 x 12.3 | 16 | 1 |
| **Z80-32k V1** (Gaehwiler Electronic, Hombrechtikon, Switzerland) | uniaxial accelerometer | NR | NR | 30 sec | NR | 8 | NR | NR | 1 |
| **Zulu watch** (Institutes for Behavior Resources, Baltimore, MD, USA) | Commercial-grade triaxial accelerometer | 80 sleep intervals/ 1 year | NR | 2 min | NR | NR | NR | NR | 1 |
| ^1^NR: Not reported | | | | | | | | | |
