# Supplementary material for: Assessment of 24-hour physical behaviour in adults via wearables: a systematic review of validation studies under laboratory conditions
Source: Int J Behav Nutr Phys Act. 2023 Jun 8;20:68. doi: 10.1186/s12966-023-01473-7 (PMC10249261; doi:10.1186/s12966-023-01473-7)
Supplement: Supplementary file 7 — Additional file 7 [file 12966_2023_1473_MOESM7_ESM.docx]

**Additional file 7.** Risk of bias for the included studies.

| **Nr.** | **Author** | **Patient Selection/Study design** | **Index measure** | **Criterion measure** | **Flow & Timing** |
| --- | --- | --- | --- | --- | --- |
| 1 | Abel et al., 2008 | HIGH | LOW | LOW | HIGH |
| 2 | Albaum et al., 2019 | HIGH | HIGH | HIGH | LOW |
| 3 | Alberto et al., 2017 | HIGH | LOW | LOW | HIGH |
| 4 | Ali et al., 2018 | HIGH | HIGH | LOW | HIGH |
| 5 | Alinia et al., 2017 | HIGH | HIGH | LOW | HIGH |
| 6 | Almeida et al., 2015 | LOW | LOW | LOW | LOW |
| 7 | Alsubheen et al., 2016 | HIGH | HIGH | LOW | HIGH |
| 8 | Ameen et al., 2019 | HIGH | HIGH | LOW | LOW |
| 9 | Aminian et al., 1999 | HIGH | LOW | LOW | LOW |
| 10 | An et al., 2017 | LOW | LOW | HIGH | HIGH |
| 11 | An et al., 2017 | HIGH | HIGH | HIGH | LOW |
| 12 | Anastasopoulou et al., 2014 | HIGH | LOW | LOW | UNCLEAR |
| 13 | Anderson et al., 2019 | HIGH | HIGH | HIGH | LOW |
| 14^a^ | Andersson et al., 2014 | HIGH | HIGH | LOW | HIGH |
| 14^b^ | Andersson et al., 2014 | HIGH | HIGH | LOW | HIGH |
| 15^a^ | Anens et al., 2021 | LOW | LOW | LOW | HIGH |
| 15^b^ | Anens et al., 2021 | LOW | HIGH | LOW | HIGH |
| 16 | Annegarn et al., 2011 | HIGH | LOW | LOW | HIGH |
| 17 | Arch et al., 2018 | HIGH | HIGH | HIGH | HIGH |
| 18 | Ayabe et al., 2008 | HIGH | LOW | LOW | UNCLEAR |
| 19 | Backhouse et al., 2013 | HIGH | HIGH | LOW | HIGH |
| 20 | Bai et al., 2018 | HIGH | HIGH | LOW | HIGH |
| 21 | Bai et al., 2016b | LOW | LOW | LOW | UNCLEAR |
| 22 | Bajaj et al., 2018 | HIGH | HIGH | HIGH | HIGH |
| 23 | Balogun et al., 1989 | HIGH | HIGH | LOW | HIGH |
| 24 | Balto et al., 2016 | HIGH | HIGH | HIGH | HIGH |
| 25^a^ | Bania, 2014 | HIGH | HIGH | LOW | HIGH |
| 25^b^ | Bania, 2014 | HIGH | HIGH | LOW | HIGH |
| 26 | Barkley et al., 2019 | HIGH | HIGH | LOW | HIGH |
| 27 | Barouni et al., 2020 | HIGH | LOW | LOW | HIGH |
| 28 | Bartholdy et al., 2018 | LOW | LOW | HIGH | HIGH |
| 29 | Bassett et al., 2000 | LOW | LOW | LOW | UNCLEAR |
| 30 | Bassett et al., 2014 | HIGH | HIGH | HIGH | LOW |
| 31 | Battenberg et al., 2017 | HIGH | HIGH | HIGH | UNCLEAR |
| 32 | Beecroft et al., 2008 | HIGH | HIGH | LOW | LOW |
| 33 | Beevi et al., 2016 | HIGH | HIGH | HIGH | HIGH |
| 34 | Bélanger et al., 2014 | HIGH | HIGH | LOW | LOW |
| 35 | Benito et al., 2012 | HIGH | HIGH | LOW | HIGH |
| 36 | Berendsen et al., 2014 | HIGH | HIGH | HIGH | HIGH |
| 37 | Bergamin et a., 2012 | HIGH | HIGH | LOW | HIGH |
| 38 | Bergman et al., 2008 | HIGH | HIGH | HIGH | HIGH |
| 39 | Berninger et al., 2018 | HIGH | HIGH | HIGH | LOW |
| 40 | Berntsen et al., 2010 | LOW | HIGH | LOW | LOW |
| 41 | Berntsen et al., 2011 | LOW | HIGH | LOW | LOW |
| 42 | Bertapelli et al., 2019 | HIGH | HIGH | HIGH | HIGH |
| 43 | Bezuidenhout et al., 2021 | HIGH | LOW | HIGH | UNCLEAR |
| 44 | Bhammar et al., 2016 | LOW | HIGH | LOW | HIGH |
| 45 | Bigué et al., 2020 | HIGH | LOW | LOW | HIGH |
| 46 | Bijnens et al., 2019 | LOW | LOW | LOW | LOW |
| 47 | Boolani et al., 2011 | HIGH | HIGH | LOW | LOW |
| 48 | Boudreaux et al., 2018 | HIGH | HIGH | LOW | UNCLEAR |
| 49 | Bourke et al., 2016 | HIGH | HIGH | LOW | LOW |
| 50^a^ | Bourke et al., 2019 | HIGH | HIGH | LOW | LOW |
| 50^b^ | Bourke et al., 2019 | HIGH | LOW | LOW | UNCLEAR |
| 51 | Bowden & Behrman, 2007 | HIGH | HIGH | HIGH | HIGH |
| 52 | Brazeau et al., 2011 | HIGH | HIGH | LOW | HIGH |
| 53 | Brazeau et al., 2014 | HIGH | HIGH | LOW | HIGH |
| 54 | Brazeau et al., 2016 | HIGH | HIGH | LOW | HIGH |
| 55 | Brian & Haegele, 2017 | HIGH | HIGH | HIGH | HIGH |
| 56 | Briseno & Smith, 2014 | HIGH | HIGH | HIGH | HIGH |
| 57 | Brown et al., 2013 | HIGH | HIGH | LOW | LOW |
| 58 | Bunn et al., 2018 | HIGH | HIGH | LOW | LOW |
| 59 | Burton et al., 2018 | HIGH | HIGH | LOW | HIGH |
| 60 | Busse et al., 2009 | HIGH | HIGH | LOW | HIGH |
| 61^a^ | Bussmann et al., 2004 | HIGH | LOW | LOW | LOW |
| 61^b^ | Bussmann et al., 2004 | HIGH | LOW | LOW | LOW |
| 62 | Cakmak et al., 2020 | HIGH | LOW | LOW | LOW |
| 63 | Calabro et al., 2014 | HIGH | LOW | LOW | LOW |
| 64 | Carroll et al., 2012 | HIGH | HIGH | LOW | HIGH |
| 65 | Casiraghi et al., 2013 | HIGH | HIGH | LOW | HIGH |
| 66 | Cavalheri et al., 2011 | LOW | HIGH | LOW | LOW |
| 67 | Cederberg et al., 2021 | HIGH | HIGH | HIGH | HIGH |
| 68 | Cellini et al., 2013 | HIGH | LOW | LOW | LOW |
| 69 | Cereda et al., 2007 | HIGH | HIGH | LOW | HIGH |
| 70 | Chakar et al., 2017 | HIGH | HIGH | LOW | LOW |
| 71 | Chandrasekar et al., 2018 | HIGH | HIGH | LOW | HIGH |
| 72 | Chang et al., 2020 | HIGH | LOW | LOW | LOW |
| 73 | Chen & Sun, 1997 | LOW | LOW | LOW | LOW |
| 74 | Chen et al., 2003 | LOW | LOW | LOW | LOW |
| 75 | Chen et al., 2016 | LOW | HIGH | LOW | LOW |
| 76 | Cheung et al., 2020 | HIGH | LOW | LOW | LOW |
| 77 | Chinoy et al., 2020 | HIGH | HIGH | LOW | UNCLEAR |
| 78 | Choi et al., 2017 | HIGH | HIGH | LOW | HIGH |
| 79 | Choi et al., 2019 | HIGH | HIGH | LOW | UNCLEAR |
| 80 | Choi et al., 2020 | HIGH | HIGH | LOW | UNCLEAR |
| 81 | Chou et al., 2009 | HIGH | HIGH | HIGH | LOW |
| 82 | Chow et al., 2017 | HIGH | HIGH | LOW | HIGH |
| 83 | Chowdhury et al., 2017 | LOW | HIGH | LOW | UNCLEAR |
| 84 | Claridge et al., 2019 | HIGH | HIGH | LOW | LOW |
| 85 | Clay et al., 2019 | HIGH | HIGH | LOW | HIGH |
| 86 | Clemens et al., 2009 | HIGH | HIGH | LOW | UNCLEAR |
| 87 | Colley et al., 2012 | HIGH | HIGH | HIGH | HIGH |
| 88 | Compagnat et al., 2018 | LOW | HIGH | LOW | LOW |
| 89 | Compagnat et al., 2019 | HIGH | HIGH | LOW | HIGH |
| 90 | Connolly et al., 2010 | HIGH | HIGH | HIGH | HIGH |
| 91 | Connolly et al., 2020 | HIGH | HIGH | HIGH | HIGH |
| 92 | Conway et al., 2018 | LOW | HIGH | LOW | UNCLEAR |
| 93 | Cook et al., 2017 | HIGH | HIGH | LOW | LOW |
| 94 | Cook et al., 2019 | HIGH | HIGH | LOW | LOW |
| 95 | Cook et al., 2018 | HIGH | HIGH | LOW | LOW |
| 96 | Coote & O’Dwyer, 2012 | LOW | HIGH | LOW | LOW |
| 97^a^ | Coulter et al., 2017 | HIGH | HIGH | LOW | HIGH |
| 97^b^ | Coulter et al., 2017 | HIGH | HIGH | LOW | HIGH |
| 98 | Cox et al.,2014 | LOW | HIGH | LOW | HIGH |
| 99 | Crisafulli et al., 2011 | HIGH | HIGH | LOW | HIGH |
| 100 | Crouter et al., 2003 | HIGH | HIGH | LOW | HIGH |
| 101 | Crouter et al., 2004 | HIGH | HIGH | LOW | HIGH |
| 102 | Crouter et al., 2008 | LOW | LOW | LOW | LOW |
| 103 | Crowley et al., 2019 | LOW | LOW | LOW | HIGH |
| 104 | Cruz et al., 2017 | HIGH | HIGH | LOW | UNCLEAR |
| 105 | Culhane et al., 2004 | HIGH | HIGH | LOW | LOW |
| 106 | Curran et al., 2021 | HIGH | HIGH | LOW | HIGH |
| 107 | Cyarto et al., 2004 | HIGH | HIGH | LOW | HIGH |
| 108 | Daligadu et al., 2018 | HIGH | HIGH | LOW | HIGH |
| 109 | Dannecker et al., 2013 | HIGH | LOW | LOW | LOW |
| 110 | Danzig et al., 2019 | HIGH | HIGH | LOW | HIGH |
| 111^a^ | Davoudi et al., 2019 | LOW | HIGH | LOW | UNCLEAR |
| 111^b^ | Davoudi et al., 2019 | LOW | HIGH | HIGH | UNCLEAR |
| 112 | De Cocker et al., 2012 | HIGH | HIGH | LOW | HIGH |
| 113 | De Man et al., 2016 | HIGH | HIGH | HIGH | LOW |
| 114 | De Ridder & De Blaiser, 2019 | HIGH | HIGH | HIGH | HIGH |
| 115^a^ | Deans et al., 2020 | HIGH | HIGH | LOW | HIGH |
| 115^b^ | Deans et al., 2020 | HIGH | HIGH | LOW | HIGH |
| 116 | Delaney et al., 2021 | HIGH | HIGH | LOW | LOW |
| 117 | Devine et al., 2021 | HIGH | HIGH | LOW | HIGH |
| 118 | DeVoe et al., 2003 | HIGH | HIGH | LOW | UNCLEAR |
| 119 | Diaz et al., 2016 | HIGH | HIGH | LOW | LOW |
| 120 | Dick et al., 2010 | HIGH | LOW | LOW | UNCLEAR |
| 121 | Dijkstra et al., 2008 | HIGH | HIGH | LOW | HIGH |
| 122 | Dijkstra et al., 2010 | LOW | HIGH | LOW | LOW |
| 123 | Domene & Easton 2014 | HIGH | LOW | LOW | HIGH |
| 124 | Dondzila & Garner, 2016 | HIGH | HIGH | LOW | HIGH |
| 125 | Dondzila et al., 2012 | HIGH | HIGH | HIGH | HIGH |
| 126 | Dondzila et al., 2018 | HIGH | HIGH | HIGH | HIGH |
| 127 | Dooley et al., 2017 | HIGH | HIGH | LOW | HIGH |
| 128 | Dorn et al., 2019 | HIGH | HIGH | HIGH | HIGH |
| 129 | Downs et al., 2015 | HIGH | HIGH | LOW | HIGH |
| 130 | Duclos et al., 2019 | HIGH | HIGH | LOW | HIGH |
| 131 | Dudek et al., 2008 | LOW | HIGH | LOW | HIGH |
| 132^a^ | Duncan et al., 2011 | HIGH | HIGH | LOW | HIGH |
| 132^b^ | Duncan et al., 2011 | HIGH | HIGH | HIGH | HIGH |
| 133 | Durkalec-Michalski et al., 2013 | HIGH | LOW | LOW | HIGH |
| 134 | Dutta et al., 2018 | HIGH | LOW | LOW | UNCLEAR |
| 135 | Dwyer et al., 2009 | HIGH | HIGH | LOW | HIGH |
| 136 | Edwardson et al., 2016 | LOW | LOW | HIGH | LOW |
| 137 | Ehrler et al., 2016 | HIGH | HIGH | LOW | LOW |
| 138 | Ehrlich et al., 2021 | HIGH | HIGH | HIGH | HIGH |
| 139 | Ekelund et al., 2002 | HIGH | LOW | LOW | LOW |
| 140 | El-Amrawy & Nounou, 2015 | HIGH | HIGH | HIGH | UNCLEAR |
| 141 | Ellender et al., 2021 | HIGH | HIGH | LOW | LOW |
| 142^a^ | Ellingson et al., 2016 | LOW | LOW | LOW | LOW |
| 142^b^ | Ellingson et al., 2016 | LOW | LOW | HIGH | LOW |
| 143 | Elsworth et al., 2009 | HIGH | HIGH | HIGH | HIGH |
| 144 | Enomoto et al., 2009 | HIGH | LOW | LOW | HIGH |
| 145 | Erdogan et al., 2010 | HIGH | HIGH | LOW | LOW |
| 146 | Esliger et al., 2007 | HIGH | HIGH | HIGH | LOW |
| 147 | Eyre et al., 2019 | HIGH | HIGH | LOW | UNCLEAR |
| 148 | Falter et al., 2019 | HIGH | HIGH | LOW | HIGH |
| 149 | Fanchamps et al., 2018 | LOW | HIGH | LOW | LOW |
| 150 | Farabi et al., 2017 | HIGH | HIGH | LOW | LOW |
| 151 | Faria et al., 2019 | HIGH | LOW | LOW | HIGH |
| 152 | Farmer et al., 2022 | HIGH | HIGH | HIGH | HIGH |
| 153 | Feehan et al., 2016 | LOW | HIGH | HIGH | HIGH |
| 154 | Feito et al., 2012 | HIGH | HIGH | HIGH | LOW |
| 155 | Feito et al., 2015 | HIGH | HIGH | HIGH | HIGH |
| 156 | Feng et al., 2017 | HIGH | LOW | LOW | UNCLEAR |
| 157 | Fietze et al., 2015 | HIGH | HIGH | LOW | UNCLEAR |
| 158 | Foerster et al, 1999 | HIGH | LOW | HIGH | UNCLEAR |
| 159 | Fokkeman et al., 2017 | HIGH | HIGH | LOW | UNCLEAR |
| 160 | Fokkenrood et al., 2014 | LOW | LOW | LOW | LOW |
| 161 | Foster et al., 2005 | HIGH | HIGH | LOW | HIGH |
| 162 | Fridriksdottir & Bonomi, 2020 | LOW | LOW | LOW | LOW |
| 163 | Fruin & Rankin, 2004 | HIGH | HIGH | LOW | LOW |
| 164 | Fulk et al., 2014 | HIGH | HIGH | LOW | HIGH |
| 165 | Fuller et al., 2021 | LOW | LOW | HIGH | HIGH |
| 166 | Furlanetto et al., 2010 | HIGH | HIGH | LOW | LOW |
| 167 | Gastin et al., 2018 | LOW | LOW | LOW | HIGH |
| 168 | Gatti et al., 2015 | HIGH | HIGH | LOW | LOW |
| 169 | Gaz et al., 2018 | HIGH | HIGH | HIGH | HIGH |
| 170 | Giannakidou et al., 2012 | HIGH | HIGH | LOW | HIGH |
| 171 | Gilgen-Ammann et al., 2019 | HIGH | HIGH | LOW | HIGH |
| 172 | Gilgen-Ammann et al., 2021 | LOW | LOW | LOW | HIGH |
| 173 | Gilmore et al., 2020 | HIGH | HIGH | LOW | LOW |
| 174 | Giurgiu et al., 2020 | LOW | HIGH | LOW | LOW |
| 175 | Glasheen et al., 2020 | HIGH | HIGH | HIGH | LOW |
| 176 | Goel et al., 2020 | HIGH | HIGH | HIGH | HIGH |
| 177 | Gould et al., 2021 | HIGH | HIGH | LOW | LOW |
| 178 | Grant et al., 2006 | HIGH | HIGH | LOW | LOW |
| 179 | Grant et al., 2008 | HIGH | HIGH | LOW | LOW |
| 180^a^ | Groot & Nieuwenhuizen, 2013 | LOW | LOW | LOW | UNCLEAR |
| 180^b^ | Groot & Nieuwenhuizen, 2013 | LOW | LOW | HIGH | UNCLEAR |
| 181 | Gruwez et al., 2017 | HIGH | HIGH | LOW | HIGH |
| 182 | Gusmer et al., 2014 | HIGH | LOW | LOW | HIGH |
| 183 | Hall et al., 2013 | HIGH | LOW | LOW | LOW |
| 184 | Harrington et al., 2011 | HIGH | LOW | LOW | LOW |
| 185^a^ | Hart et al., 2011 | HIGH | HIGH | LOW | LOW |
| 185^b^ | Hart et al., 2011 | HIGH | HIGH | LOW | LOW |
| 186 | Härtel et al., 2011 | HIGH | LOW | LOW | LOW |
| 187 | Hartung et al., 2020 | HIGH | HIGH | HIGH | HIGH |
| 188 | Hasson et al., 2009 | HIGH | HIGH | HIGH | UNCLEAR |
| 189 | Haymes & Byrnes, 1993 | HIGH | HIGH | LOW | HIGH |
| 190^a^ | Hedayatrad et al., 2021 | HIGH | LOW | HIGH | LOW |
| 190^b^ | Hedayatrad et al., 2021 | HIGH | LOW | LOW | LOW |
| 191 | Hedner et al., 2004 | HIGH | LOW | LOW | LOW |
| 192 | Heiermann et al., 2011 | HIGH | HIGH | LOW | HIGH |
| 193 | Hendelman et al., 2000 | LOW | HIGH | LOW | LOW |
| 194^a^ | Hendrikx et al., 2017 | LOW | HIGH | LOW | HIGH |
| 194^b^ | Hendrikx et al., 2017 | LOW | HIGH | HIGH | HIGH |
| 195 | Hergenroeder et al., 2018 | HIGH | HIGH | HIGH | HIGH |
| 196 | Hergenroeder et al., 2019 | HIGH | HIGH | HIGH | HIGH |
| 197 | Herkert et al., 2019 | LOW | HIGH | LOW | HIGH |
| 198 | Herman Hansen et al., 2014 | HIGH | HIGH | LOW | UNCLEAR |
| 199 | Hernández Belmonte et al., 2018 | HIGH | LOW | LOW | LOW |
| 200 | Herrmann et al., 2011 | HIGH | LOW | HIGH | HIGH |
| 201 | Hibbing et al., 2018 | LOW | LOW | LOW | LOW |
| 202 | Hickey et al., 2016 | HIGH | HIGH | LOW | HIGH |
| 203 | Hildebrand et al., 2014 | LOW | LOW | LOW | LOW |
| 204 | Hill et al., 2010 | LOW | HIGH | LOW | HIGH |
| 205 | Hiremath et al., 2013 | LOW | LOW | LOW | LOW |
| 206 | Ho et al., 2019 | HIGH | LOW | LOW | LOW |
| 207 | Höchsmann et al., 2018 | HIGH | HIGH | LOW | HIGH |
| 208 | Holbrook et al., 2009 | HIGH | HIGH | HIGH | HIGH |
| 209 | Horemans et al., 2019 | HIGH | HIGH | LOW | LOW |
| 210 | Horner et al., 2011 | HIGH | HIGH | LOW | HIGH |
| 211 | Horner et al., 2013 | HIGH | HIGH | LOW | HIGH |
| 212 | Horvath et al., 2007 | HIGH | HIGH | HIGH | UNCLEAR |
| 213 | Huang et al., 2016 | HIGH | HIGH | LOW | HIGH |
| 214 | Huberty et al., 2021 | HIGH | LOW | LOW | HIGH |
| 215 | Husted & Llewellyn 2017 | HIGH | HIGH | LOW | HIGH |
| 216 | Hustved et al., 2004 | LOW | LOW | LOW | HIGH |
| 217 | Ichinoseki-Sekine et al., 2006 | HIGH | LOW | HIGH | HIGH |
| 218 | Imboden et al., 2018 | LOW | LOW | LOW | UNCLEAR |
| 219 | Jakicic et al., 2004 | HIGH | HIGH | LOW | HIGH |
| 220 | Jayaraman et al., 2016 | HIGH | LOW | LOW | LOW |
| 221 | Jayaraman et al., 2018 | LOW | LOW | LOW | LOW |
| 222 | Jean-Louis et al., 1996 | HIGH | HIGH | LOW | LOW |
| 223 | Jean-Louis et al., 1998 | HIGH | HIGH | LOW | HIGH |
| 224 | Jean-Louis et al., 2000 | HIGH | LOW | LOW | LOW |
| 225 | Jean-Louis et al., 2001 | HIGH | LOW | LOW | LOW |
| 226 | Jehn et al., 2010 | HIGH | HIGH | HIGH | HIGH |
| 227 | Jiang & Larson, 2013 | LOW | HIGH | HIGH | HIGH |
| 228 | John et al., 2018 | HIGH | HIGH | HIGH | HIGH |
| 229 | Johnson, 2015 | HIGH | HIGH | HIGH | HIGH |
| 230 | Johnson et al., 2015 | HIGH | HIGH | LOW | HIGH |
| 231 | Jones et al., 2018 | HIGH | HIGH | LOW | HIGH |
| 232 | Jung et al., 2020 | LOW | HIGH | LOW | HIGH |
| 233 | Kahawage et al., 2019 | HIGH | HIGH | LOW | UNCLEAR |
| 234 | Kamper et al., 2016 | HIGH | LOW | LOW | HIGH |
| 235 | Kanady et al., 2010 | HIGH | HIGH | LOW | LOW |
| 236 | Kanady et al., 2020 | HIGH | LOW | LOW | LOW |
| 237 | Kane et al., 2010 | HIGH | HIGH | LOW | HIGH |
| 238 | Kapella et al., 2017 | HIGH | HIGH | LOW | HIGH |
| 239 | Kaplan et al., 2012 | HIGH | LOW | LOW | HIGH |
| 240 | Karabulut et al., 2005 | HIGH | LOW | HIGH | HIGH |
| 241 | Karaca et al., 2021 | HIGH | HIGH | LOW | UNCLEAR |
| 242 | Karinharju et al., 2019 | LOW | HIGH | LOW | HIGH |
| 243 | Kastelic et al., 2021 | LOW | HIGH | LOW | HIGH |
| 244 | Kayes et al., 2009 | LOW | HIGH | HIGH | HIGH |
| 245 | Keating et al., 2018 | HIGH | HIGH | HIGH | UNCLEAR |
| 246 | Kelly et al., 2013 | HIGH | HIGH | LOW | HIGH |
| 247 | Kemp et al., 2020 | LOW | HIGH | LOW | LOW |
| 248 | Kendall et al., 2019 | HIGH | HIGH | LOW | LOW |
| 249 | Keppler et al., 2019 | HIGH | LOW | LOW | HIGH |
| 250 | Kim & Welk, 2015 | LOW | LOW | LOW | UNCLEAR |
| 251 | Kim et al., 2021 | HIGH | HIGH | LOW | LOW |
| 252 | King et al., 2004 | HIGH | LOW | LOW | LOW |
| 253 | Klaasen et al., 2016 | HIGH | HIGH | LOW | HIGH |
| 254 | Klassen et al., 2017 | LOW | HIGH | HIGH | LOW |
| 255 | Klenk et al., 2016 | LOW | HIGH | HIGH | LOW |
| 256 | Koehler et al., 2011 | HIGH | HIGH | LOW | HIGH |
| 257 | Koehler et al., 2013 | HIGH | HIGH | LOW | LOW |
| 258 | Koenders et al., 2018 | LOW | HIGH | LOW | HIGH |
| 259 | Kooiman et al., 2015 | HIGH | HIGH | HIGH | HIGH |
| 260 | Korpan et al., 2015 | HIGH | HIGH | HIGH | HIGH |
| 261 | Kosmadopoulos et al., 2014 | HIGH | LOW | LOW | LOW |
| 262 | Kossi et al., 2021 | HIGH | LOW | LOW | LOW |
| 263 | Kramer et al., 2018 | HIGH | HIGH | LOW | LOW |
| 264 | Kuffel et al., 2011 | LOW | LOW | LOW | LOW |
| 265 | Kumahara et al., 2004 | LOW | HIGH | LOW | HIGH |
| 266 | Kumahara et al., 2009 | HIGH | HIGH | LOW | HIGH |
| 267 | Kumahara et al., 2015 | HIGH | HIGH | LOW | UNCLEAR |
| 268 | Kushida et al., 2001 | HIGH | LOW | LOW | UNCLEAR |
| 269 | Kwan et al., 2020 | HIGH | HIGH | LOW | HIGH |
| 270 | Kwon et al., 2010 | HIGH | HIGH | HIGH | UNCLEAR |
| 271 | Kwon et al., 2021 | LOW | LOW | LOW | HIGH |
| 272 | Laakso et al., 2004 | HIGH | LOW | LOW | HIGH |
| 273 | Ladlow et al., 2019 | HIGH | LOW | LOW | LOW |
| 274 | Lai et al., 2020 | HIGH | HIGH | HIGH | LOW |
| 275 | Lamont et al., 2018 | HIGH | HIGH | LOW | HIGH |
| 276^a^ | Larkin et al., 2016 | LOW | HIGH | LOW | HIGH |
| 276^b^ | Larkin et al., 2016 | LOW | HIGH | LOW | HIGH |
| 277 | Lauritzen et al., 2013 | HIGH | HIGH | LOW | HIGH |
| 278 | Le Masurier & Tudor-Locke, 2003 | HIGH | HIGH | LOW | LOW |
| 279 | Le Masurier et al., 2004 | HIGH | HIGH | LOW | LOW |
| 280 | Leaf & MacRae, 1995 | HIGH | LOW | LOW | HIGH |
| 281 | Lebleu et al., 2020 | HIGH | HIGH | HIGH | HIGH |
| 282 | Lee & Laurson, 2015a | HIGH | HIGH | HIGH | HIGH |
| 283 | Lee & Tse, 2019 | HIGH | HIGH | LOW | LOW |
| 284 | Lee et al., 2011 | HIGH | HIGH | LOW | HIGH |
| 285 | Lee et al., 2014 | LOW | LOW | LOW | LOW |
| 286 | Lee et al., 2015b | HIGH | HIGH | HIGH | HIGH |
| 287 | Leicht & Crowther, 2007 | HIGH | HIGH | HIGH | HIGH |
| 288 | Leth et al., 2017 | HIGH | HIGH | HIGH | UNCLEAR |
| 289 | Leung et al., 2021 | HIGH | HIGH | LOW | HIGH |
| 290 | Levine et al., 2000 | LOW | HIGH | LOW | UNCLEAR |
| 291 | Levine et al., 2003 | HIGH | HIGH | LOW | UNCLEAR |
| 292 | Leving et al., 2018 | HIGH | LOW | LOW | LOW |
| 293 | Liang & Getchell, 2018 | LOW | HIGH | LOW | LOW |
| 294 | Lichstein et al., 2006 | HIGH | LOW | LOW | UNCLEAR |
| 295 | Liu et al., 2015 | HIGH | HIGH | HIGH | HIGH |
| 296 | Lopes et al., 2009 | HIGH | HIGH | LOW | HIGH |
| 297 | Lopez et al., 2018 | HIGH | HIGH | LOW | LOW |
| 298 | Loprinzi & Edwards, 2018 | HIGH | HIGH | HIGH | HIGH |
| 299 | Lötjönen et al., 2003 | HIGH | LOW | LOW | HIGH |
| 300 | Louter et al., 2014 | HIGH | HIGH | LOW | LOW |
| 301 | Lowe et al., 2010 | HIGH | HIGH | LOW | HIGH |
| 302 | Lützner et al., 2014 | HIGH | HIGH | LOW | LOW |
| 303 | Lynn et al., 2020 | HIGH | HIGH | LOW | HIGH |
| 304 | Maddocks et al., 2010 | HIGH | HIGH | LOW | UNCLEAR |
| 305 | Madigan, 2019 | HIGH | HIGH | LOW | HIGH |
| 306 | Maganja et al., 2020 | HIGH | HIGH | HIGH | HIGH |
| 307 | Magistro et al., 2018 | HIGH | LOW | HIGH | HIGH |
| 308 | Maglione et al., 2013 | HIGH | HIGH | LOW | LOW |
| 309 | Mahadevan et al., 2021 | HIGH | LOW | LOW | HIGH |
| 310 | Mahendran et al., 2016 | HIGH | HIGH | HIGH | HIGH |
| 311 | Mammen et al., 2012 | HIGH | HIGH | LOW | HIGH |
| 312 | Mandigout et al., 2017 | LOW | HIGH | LOW | HIGH |
| 313 | Manns & Haennel, 2012 | HIGH | HIGH | LOW | LOW |
| 314 | Marino et al., 2013 | HIGH | LOW | LOW | LOW |
| 315 | Marsh et al., 2007 | HIGH | HIGH | HIGH | HIGH |
| 316 | Martien et al., 2015 | LOW | HIGH | LOW | LOW |
| 317 | Martien et al., 2015 | LOW | HIGH | HIGH | HIGH |
| 318 | Martin et al., 2012 | HIGH | HIGH | HIGH | HIGH |
| 319 | Martin et al., 2015 | HIGH | HIGH | LOW | UNCLEAR |
| 320 | Martinato et al., 2021 | HIGH | HIGH | HIGH | HIGH |
| 321 | Maskevich et al., 2017 | HIGH | HIGH | LOW | UNCLEAR |
| 322 | Matthews et al., 2000 | HIGH | HIGH | LOW | UNCLEAR |
| 323 | McClain et al., 2010 | HIGH | HIGH | HIGH | UNCLEAR |
| 324 | McCullagh et al., 2017 | LOW | HIGH | LOW | LOW |
| 325 | McDevitt et al., 2021 | HIGH | LOW | HIGH | HIGH |
| 326 | Melanson & Freedson, 1995 | HIGH | HIGH | LOW | LOW |
| 327 | Melanson et al., 2004 | HIGH | HIGH | HIGH | HIGH |
| 328 | Miller et al., 2020 | HIGH | HIGH | LOW | LOW |
| 329 | Miller et al., 2021 | HIGH | HIGH | LOW | LOW |
| 330 | Modave et al., 2017 | HIGH | HIGH | HIGH | HIGH |
| 331 | Montes et al., 2017 | HIGH | HIGH | LOW | UNCLEAR |
| 332 | Montes et al., 2019 | HIGH | HIGH | HIGH | LOW |
| 333 | Montes et al., 2020 | HIGH | HIGH | HIGH | HIGH |
| 334 | Montgomery et al., 2019 | HIGH | HIGH | LOW | HIGH |
| 335 | Montgomery-Downs et al., 2012 | HIGH | HIGH | LOW | LOW |
| 336 | Montoye et al., 2016 | LOW | LOW | LOW | LOW |
| 337 | Montoye et al., 2016 | LOW | LOW | HIGH | LOW |
| 338 | Montoye et al., 2017 | LOW | LOW | LOW | HIGH |
| 339 | Montoye et al., 2017 | HIGH | HIGH | LOW | HIGH |
| 340 | Montoye et al., 2019 | HIGH | LOW | LOW | LOW |
| 341 | Moreno et al., 2020 | HIGH | HIGH | LOW | HIGH |
| 342 | Moreno-Pino et al., 2019 | HIGH | HIGH | LOW | HIGH |
| 343 | Morris et al., 2019 | HIGH | HIGH | LOW | UNCLEAR |
| 344 | Motl et al., 2005 | HIGH | HIGH | LOW | HIGH |
| 345 | Motl et al., 2011 | HIGH | HIGH | HIGH | HIGH |
| 346 | Mudge et al., 2007 | HIGH | HIGH | HIGH | HIGH |
| 347 | Murakami et al., 2019 | HIGH | HIGH | LOW | HIGH |
| 348 | Nakazaki et al., 2014 | HIGH | LOW | LOW | HIGH |
| 349 | Navalta et al., 2018 | HIGH | HIGH | HIGH | LOW |
| 350 | Nazarahari &Rouhani, 2018 | HIGH | LOW | LOW | LOW |
| 351 | Nelson et al., 2016 | LOW | HIGH | LOW | LOW |
| 352 | Ng et al., 2012 | HIGH | HIGH | HIGH | HIGH |
| 353 | Nguyen et al., 2013 | HIGH | LOW | LOW | UNCLEAR |
| 354 | Nichols et al., 1999 | HIGH | LOW | LOW | UNCLEAR |
| 355 | Nichols et al., 2000 | HIGH | HIGH | LOW | LOW |
| 356 | Nielson et al., 2011 | HIGH | HIGH | LOW | HIGH |
| 357 | Nightingale et al., 2014 | HIGH | HIGH | LOW | UNCLEAR |
| 358 | Nightingale et al., 2015 | HIGH | HIGH | LOW | LOW |
| 359 | Noah et al., 2013 | HIGH | HIGH | LOW | HIGH |
| 360 | Nuss et al., 2019 | HIGH | HIGH | LOW | LOW |
| 361 | Nuss et al., 2020 | HIGH | LOW | LOW | LOW |
| 362 | O`Brien et al., 2021 | HIGH | HIGH | LOW | HIGH |
| 363 | O`Brien et al., 2018a | HIGH | LOW | LOW | UNCLEAR |
| 364^a^ | O`Brien et al., 2020 | HIGH | HIGH | LOW | LOW |
| 354^b^ | O`Brien et al., 2020 | HIGH | HIGH | LOW | LOW |
| 365 | O`Connell et al., 2017 | HIGH | HIGH | LOW | LOW |
| 366 | O`Connell et al., 2016 | HIGH | HIGH | LOW | UNCLEAR |
| 367 | O`Driscoll et al., 2013 | HIGH | HIGH | LOW | LOW |
| 368 | O`Driscoll et al., 2020a | LOW | LOW | LOW | HIGH |
| 369 | O`Hare et al., 2014 | HIGH | HIGH | LOW | LOW |
| 370 | Ohkawara et al., 2011 | LOW | LOW | LOW | HIGH |
| 371 | Oomen et al., 2018 | HIGH | LOW | LOW | HIGH |
| 372 | Osawa et al., 2013 | HIGH | HIGH | LOW | HIGH |
| 373 | Pallin et al., 2014 | HIGH | HIGH | LOW | LOW |
| 374 | Pambianco et al., 1990 | HIGH | LOW | LOW | HIGH |
| 375 | Papazoglou et al., 2006 | LOW | LOW | LOW | LOW |
| 376 | Paquet et al., 2007 | HIGH | LOW | LOW | LOW |
| 377 | Paradiso et al., 2020 | HIGH | HIGH | LOW | HIGH |
| 378 | Parak et al., 2017 | HIGH | LOW | LOW | LOW |
| 379 | Park et al., 2011 | HIGH | HIGH | LOW | HIGH |
| 380 | Park et al., 2016 | HIGH | HIGH | LOW | HIGH |
| 381 | Passler et al., 2019 | HIGH | HIGH | LOW | HIGH |
| 382 | Patel et al., 2007 | HIGH | HIGH | LOW | HIGH |
| 383 | Peiris et al., 2017 | HIGH | HIGH | LOW | HIGH |
| 384 | Petrucci et al., 2018 | HIGH | HIGH | LOW | HIGH |
| 385 | Pigeon et al., 2018 | HIGH | HIGH | LOW | LOW |
| 386 | Pino-Ortega et al., 2021 | HIGH | HIGH | HIGH | UNCLEAR |
| 387 | Pitchford & Yun, 2010 | HIGH | HIGH | HIGH | HIGH |
| 388 | Pollak et al., 2001 | HIGH | HIGH | LOW | LOW |
| 389 | Pomeroy et al., 2011 | HIGH | HIGH | HIGH | UNCLEAR |
| 390 | Pope et al., 2019 | HIGH | HIGH | HIGH | HIGH |
| 391 | Pope et al., 2019 | LOW | HIGH | LOW | HIGH |
| 392 | Powell et al., 2016 | LOW | HIGH | LOW | HIGH |
| 393 | Pribyslavska et al., 2018 | HIGH | HIGH | LOW | HIGH |
| 394 | Price et al., 2017 | HIGH | HIGH | LOW | HIGH |
| 395 | Prieto-Centurion et al., 2016 | HIGH | HIGH | HIGH | HIGH |
| 396 | Radtke et al., 2021 | LOW | HIGH | HIGH | LOW |
| 397 | Rampichini et al., 2016 | HIGH | LOW | LOW | HIGH |
| 398 | Ray et al., 2014 | HIGH | LOW | LOW | LOW |
| 399 | Raymond et al., 2015 | HIGH | HIGH | LOW | LOW |
| 400 | Razjouyan et al., 2017 | HIGH | LOW | LOW | HIGH |
| 401 | Reddy et al., 2018 | HIGH | HIGH | LOW | LOW |
| 402 | Redfield et al., 2013 | HIGH | LOW | HIGH | HIGH |
| 403 | Reece et al., 2015 | LOW | HIGH | LOW | UNCLEAR |
| 404 | Reeve et al., 2014 | HIGH | HIGH | LOW | HIGH |
| 405 | Reid & Dawson, 1999 | HIGH | HIGH | LOW | LOW |
| 406 | Renfrew et al., 2020 | HIGH | HIGH | LOW | HIGH |
| 407 | Rennie et al., 2000 | HIGH | HIGH | LOW | HIGH |
| 408 | Riel et al., 2016 | HIGH | HIGH | LOW | LOW |
| 409 | Robert-Lewis et al., 2021 | LOW | HIGH | LOW | HIGH |
| 410 | Roberts et al., 2020 | HIGH | HIGH | LOW | LOW |
| 411 | Roos et al., 2017 | HIGH | HIGH | LOW | LOW |
| 412 | Rothney et al., 2010 | LOW | HIGH | LOW | HIGH |
| 413 | Rousset et al., 2015 | LOW | LOW | LOW | HIGH |
| 414 | Rowlands et al., 2004 | HIGH | HIGH | LOW | UNCLEAR |
| 415 | Rowlands et al., 2016b | HIGH | LOW | HIGH | HIGH |
| 416 | Rüdiger et al., 2019 | HIGH | HIGH | HIGH | UNCLEAR |
| 417 | Rupp & Balkin, 2011 | HIGH | LOW | LOW | LOW |
| 418 | Ryan & Gormley, 2013 | HIGH | HIGH | LOW | LOW |
| 419 | Ryan et al., 2006 | HIGH | HIGH | LOW | LOW |
| 420^a^ | Ryan et al., 2008 | HIGH | HIGH | LOW | HIGH |
| 420^b^ | Ryan et al., 2008 | HIGH | HIGH | LOW | HIGH |
| 421 | Ryde et al., 2011 | HIGH | HIGH | LOW | HIGH |
| 422 | Sadeh et al., 1994 | HIGH | LOW | LOW | HIGH |
| 423 | Sanchez-Triago, 2020 | HIGH | HIGH | HIGH | HIGH |
| 424 | Sandroff et al., 2012 | HIGH | HIGH | LOW | HIGH |
| 425 | Sandroff et al., 2014 | HIGH | HIGH | LOW | HIGH |
| 426 | Santos-Lozano et al., 2013 | HIGH | HIGH | LOW | HIGH |
| 427 | Santos-Lozano et al., 2017 | HIGH | HIGH | LOW | UNCLEAR |
| 428 | Sargent et al., 2018 | HIGH | HIGH | LOW | LOW |
| 429 | Sasaki et al., 2015 | HIGH | HIGH | LOW | UNCLEAR |
| 430 | Sasaki et al., 2016 | LOW | LOW | HIGH | HIGH |
| 431 | Schaffer et al., 2017 | HIGH | HIGH | LOW | HIGH |
| 432 | Schneider et al., 2003 | HIGH | HIGH | HIGH | UNCLEAR |
| 433 | Schneller et al., 2015 | HIGH | LOW | LOW | HIGH |
| 434 | Scott et al., 2021 | HIGH | LOW | LOW | HIGH |
| 435 | Sears et al., 2017 | HIGH | HIGH | HIGH | UNCLEAR |
| 436^a^ | Sellers et al., 2016 | LOW | HIGH | LOW | LOW |
| 436^b^ | Sellers et al., 2016 | LOW | HIGH | LOW | LOW |
| 437 | Serra et al., 2017 | LOW | HIGH | LOW | HIGH |
| 438 | Sharif & BaHammam, 2013 | HIGH | LOW | LOW | LOW |
| 439 | Shcherbina et al., 2017 | HIGH | HIGH | LOW | UNCLEAR |
| 440 | Shepherd et al., 1999 | HIGH | HIGH | HIGH | HIGH |
| 441 | Shimizu et al., 2018 | HIGH | HIGH | HIGH | HIGH |
| 442 | Shin et al., 2015 | HIGH | HIGH | LOW | HIGH |
| 443 | Simonsen et al., 2020 | HIGH | HIGH | LOW | UNCLEAR |
| 444 | Simpson et al., 2015 | HIGH | HIGH | LOW | HIGH |
| 445 | Sirichana et al., 2017 | HIGH | LOW | LOW | HIGH |
| 446 | Sivertsen et al., 2006 | HIGH | HIGH | LOW | HIGH |
| 447 | Sjöberg et al., 2021 | HIGH | HIGH | LOW | HIGH |
| 448 | Skipworth et al., 2011 | LOW | HIGH | LOW | LOW |
| 449 | Skotte et al., 2014 | HIGH | HIGH | HIGH | UNCLEAR |
| 450 | Slater et al., 2014 | HIGH | LOW | LOW | UNCLEAR |
| 451 | Slootmaker et al., 2009 | HIGH | LOW | LOW | HIGH |
| 452 | Smith & Schroeder, 2008 | HIGH | HIGH | HIGH | HIGH |
| 453 | Smith et al., 2012 | LOW | HIGH | LOW | LOW |
| 454 | Smith et al., 2017 | HIGH | HIGH | LOW | UNCLEAR |
| 455 | Smith et al., 2019 | HIGH | HIGH | HIGH | LOW |
| 456 | Soric et al., 2012 | HIGH | HIGH | LOW | HIGH |
| 457 | Spielmanns et al., 2019 | HIGH | HIGH | LOW | HIGH |
| 458 | Stanish et al., 2004 | HIGH | HIGH | HIGH | UNCLEAR |
| 459 | Stansfield et al., 2015 | HIGH | HIGH | LOW | LOW |
| 460 | Steeves et al., 2011 | HIGH | HIGH | HIGH | UNCLEAR |
| 461 | Stenbäck et al., 2021 | HIGH | LOW | LOW | HIGH |
| 462 | Stewart et al., 2018 | LOW | LOW | LOW | LOW |
| 463^a^ | Storm et al., 2015 | HIGH | HIGH | HIGH | UNCLEAR |
| 463^b^ | Storm et al., 2015 | HIGH | HIGH | HIGH | UNCLEAR |
| 464 | Storti et al., 2008 | HIGH | HIGH | HIGH | HIGH |
| 465 | Strath et al., 2001 | HIGH | HIGH | LOW | HIGH |
| 466^a^ | Strath et al., 2015 | HIGH | LOW | LOW | HIGH |
| 466^b^ | Strath et al., 2015 | HIGH | LOW | HIGH | HIGH |
| 467 | Sugino et al., 2011 | HIGH | HIGH | HIGH | HIGH |
| 468 | Sushames et al., 2016 | HIGH | LOW | LOW | HIGH |
| 469 | Svarre et al., 2020 | HIGH | HIGH | LOW | UNCLEAR |
| 470 | Swan et al., 1997 | HIGH | HIGH | LOW | HIGH |
| 471 | Swartz et al., 2003 | HIGH | HIGH | HIGH | UNCLEAR |
| 472 | Swartz et al., 2009 | HIGH | HIGH | LOW | HIGH |
| 473 | Syed et al., 2020 | HIGH | HIGH | LOW | LOW |
| 474 | Taibi et al., 2013 | HIGH | LOW | LOW | UNCLEAR |
| 475 | Takacs et al., 2013 | HIGH | HIGH | LOW | LOW |
| 476 | Tam & Cheung, 2018 | HIGH | HIGH | LOW | HIGH |
| 477^a^ | Taraldsen et al., 2011 | LOW | HIGH | LOW | LOW |
| 477^b^ | Taraldsen et al., 2011 | LOW | HIGH | LOW | LOW |
| 478 | Taylor et al., 2014 | LOW | HIGH | LOW | LOW |
| 479 | Taylor et al., 2018 | HIGH | HIGH | LOW | HIGH |
| 480 | Te Lindert et al., 2020 | HIGH | LOW | LOW | LOW |
| 481 | Tedesco et al., 2019 | HIGH | HIGH | LOW | HIGH |
| 482 | Thiebaud et al., 2018 | HIGH | HIGH | LOW | HIGH |
| 483 | Thompson et al., 2006 | HIGH | LOW | LOW | HIGH |
| 484 | Thorup et al., 2017 | HIGH | HIGH | HIGH | HIGH |
| 485 | Tierney et al., 2013 | HIGH | HIGH | LOW | LOW |
| 486 | Topshoi et al., 2018 | HIGH | HIGH | HIGH | HIGH |
| 487 | Toth et al., 2017 | LOW | HIGH | HIGH | HIGH |
| 488 | Treacy et al., 2017 | HIGH | HIGH | HIGH | HIGH |
| 489 | Tripette et al., 2014 | HIGH | LOW | LOW | UNCLEAR |
| 490 | Tsang et al., 2015 | LOW | HIGH | LOW | LOW |
| 491 | Tucker et al., 2015 | LOW | HIGH | LOW | LOW |
| 492 | Tudor Locke et al., 2015 | HIGH | HIGH | LOW | UNCLEAR |
| 493 | Tudor-Locke et al., 2006 | HIGH | HIGH | LOW | UNCLEAR |
| 494 | Turner et al., 2012 | HIGH | HIGH | HIGH | HIGH |
| 495 | Tweedy & Trost, 2005 | HIGH | HIGH | LOW | LOW |
| 496 | Uchimura et al, 2019 | HIGH | HIGH | LOW | HIGH |
| 497 | Ummels et al., 2018 | LOW | HIGH | LOW | HIGH |
| 498^a^ | Ummels et al., 2020 | LOW | HIGH | LOW | HIGH |
| 498^b^ | Ummels et al., 2020 | LOW | HIGH | LOW | HIGH |
| 499 | Valkenet & Veenhof, 2019 | HIGH | HIGH | LOW | LOW |
| 500 | Vallieres & Morin, 2003 | HIGH | LOW | LOW | LOW |
| 501 | Van der Kooi et al., 2013 | HIGH | HIGH | LOW | LOW |
| 502 | Van Hees et al., 2009 | HIGH | LOW | LOW | UNCLEAR |
| 503 | Van Hees et al., 2018 | HIGH | LOW | LOW | HIGH |
| 504 | Van Hoye et al., 2014 | HIGH | LOW | LOW | LOW |
| 505 | Van Laarhoven et al., 2016 | LOW | LOW | LOW | LOW |
| 506 | Van Remoortel et al., 2012 | LOW | HIGH | LOW | LOW |
| 507 | Van Rooij et al., 2020 | LOW | HIGH | LOW | LOW |
| 508 | Vanhelst et al., 2012 | LOW | LOW | LOW | LOW |
| 509 | Vanroy et al., 2013 | HIGH | LOW | LOW | LOW |
| 510 | Veerabhadrappa et al., 2018 | HIGH | HIGH | LOW | LOW |
| 511 | Vernillo et al., 2015 | HIGH | HIGH | LOW | LOW |
| 512 | Vernillo, 2014 | HIGH | HIGH | LOW | LOW |
| 513 | Vetrovsky et al., 2019 | HIGH | HIGH | HIGH | LOW |
| 514 | Vieira Costa et al., 2019 | HIGH | HIGH | LOW | HIGH |
| 515 | Wahl et al., 2017 | HIGH | HIGH | LOW | UNCLEAR |
| 516 | Walch et al., 2019 | HIGH | HIGH | LOW | HIGH |
| 517 | Wallmann-Sperlich et al., 2014 | HIGH | HIGH | LOW | UNCLEAR |
| 518 | Wang, et al., 2008 | HIGH | LOW | LOW | LOW |
| 519 | Wang, et al., 2017 | HIGH | HIGH | HIGH | HIGH |
| 520 | Warms & Belza, 2004 | HIGH | HIGH | HIGH | HIGH |
| 521 | Webber & John, 2016 | HIGH | LOW | HIGH | HIGH |
| 522 | Webber et al., 2014 | HIGH | HIGH | HIGH | LOW |
| 523 | Welk et al., 2017 | HIGH | HIGH | LOW | HIGH |
| 524 | Welk et al., 2003 | HIGH | HIGH | LOW | HIGH |
| 525 | Wellons et al., 2019 | HIGH | HIGH | LOW | HIGH |
| 526 | Wendel et al., 2019 | HIGH | HIGH | LOW | HIGH |
| 527 | Wetten et al., 2014 | LOW | LOW | LOW | HIGH |
| 528 | Wetzler et al., 2003 | HIGH | LOW | HIGH | HIGH |
| 529 | Whitrow et al., 2019 | HIGH | HIGH | LOW | HIGH |
| 530 | Whybrow et al., 2013 | HIGH | HIGH | LOW | HIGH |
| 531 | Wong, et al., 2017 | HIGH | HIGH | LOW | HIGH |
| 532^a^ | Woodman et al., 2017 | LOW | HIGH | LOW | HIGH |
| 532^b^ | Woodman et al., 2017 | LOW | HIGH | HIGH | HIGH |
| 533 | Wu et al., 2021 | HIGH | HIGH | LOW | HIGH |
| 534 | Xie et al., 2018 | HIGH | HIGH | LOW | LOW |
| 535^a^ | Yang et al., 2018 | HIGH | HIGH | LOW | UNCLEAR |
| 535^b^ | Yang et al., 2018 | HIGH | HIGH | HIGH | UNCLEAR |
| 536 | Yokoyama et al., 2002 | HIGH | LOW | LOW | HIGH |
| 537 | Yoon et al., 2018 | HIGH | LOW | LOW | HIGH |
| 538 | Zambotti et al., 2015 | HIGH | HIGH | LOW | LOW |
| 539 | Zambotti et al., 2018 | HIGH | HIGH | LOW | LOW |
| 540 | Zanetti et al., 2014 | HIGH | HIGH | LOW | UNCLEAR |
| 541^a^ | Zhang et al, 2003 | HIGH | HIGH | HIGH | HIGH |
| 541^b^ | Zhang et al, 2003 | HIGH | HIGH | HIGH | HIGH |
| 542 | Zhang et al., 2016 | HIGH | HIGH | HIGH | LOW |
| 543 | Zhang et al., 2018 | HIGH | HIGH | LOW | LOW |
| 544 | Zhu & Lee, 2010 | HIGH | HIGH | HIGH | UNCLEAR |
| 545 | Zorrilla-Revilla et al., 2017 | HIGH | HIGH | LOW | UNCLEAR |
| ^a^ Intensity outcome; ^b^ Posture/Activity type outcome | | | | | |
