# Supplementary material for: Assessment of 24-hour physical behaviour in adults via wearables: a systematic review of validation studies under laboratory conditions
Source: Int J Behav Nutr Phys Act. 2023 Jun 8;20:68. doi: 10.1186/s12966-023-01473-7 (PMC10249261; doi:10.1186/s12966-023-01473-7)
Supplement: Supplementary file 8 — Additional file 8 [file 12966_2023_1473_MOESM8_ESM.docx]

**Additional file 8.** Validity of wearables seperated by dimensions and age groups.

| **Wearable** | **Younger aged adults (18-40 yrs)** | | | **Middle aged adults (41-64 yrs)** | | | **Older aged adults (≥ 65 yrs)** | | |
| --- | --- | --- | --- | --- | --- | --- | --- | --- | --- |
|  | **Bio.^1^** | **Pos./AT^2^** | **Int.^3^** | **Bio.^1^** | **Pos./AT^2^** | **Int.^3^** | **Bio.^1^** | **Pos./AT^2^** | **Int.^3^** |
| **3DNX model v3** |  |  | **↑↔** |  |  |  |  |  |  |
| **Accusplit AX120** |  |  | **↓** |  |  |  |  |  |  |
| **Accusplit AX2710** |  |  |  |  |  |  |  |  | **↑** |
| **Accusplit Eagle 120** |  |  |  |  |  |  |  |  | **↓** |
| **Accusplit Eagle Digi-Walker 2** |  |  | **↔**^4^ |  |  |  |  |  |  |
| **Acos FS500** |  |  | **↔** |  |  |  |  |  |  |
| **Actibelt** |  |  |  |  |  |  |  |  | **↑** |
| **Actical** |  |  | **↔↔↔↔↔↔↔↓↓↔↔** |  |  | **↓↔↓** |  |  | **↔↓** |
| **Actical Z-Series** | **↑↔** |  |  |  |  |  |  |  |  |
| **ActiGraph AM7164** | **↔** |  | **↔↔↑↔↔↓↔↔↔↔↔↓↓↔↔↑** | **↔** |  | **↔↑↑** | **↔** |  |  |
| **Actigraph AMA-32** | **↑** |  |  | **↑** |  |  |  |  |  |
| **ActiGraph GT1M** |  |  | **↓↔↔↔↑↔↔↔↔↓↔↔** |  |  | **↔** |  |  | **↔** |
| **ActiGraph GT3X** |  | **↓↔** | **↓↓↔↔↔↔↔↑↔↔↔↓↑↓↔↔↔↔↔↓↓↓↔↓↔** |  | **↑** | **↔↔↔↔↑↑↔↓↔↔↔** |  |  | **↓↓↔↑↓↔↓↔** |
| **ActiGraph GT3X+** | **↔↔** | **↔↔↔↑↔↑↑↔↔** | **↔↓↓↑↔↑↔↓↔↔↔↔↓↔↔↑↑↓↔↔↔↔↑↔↔↑↔** |  | **↔↑↑** | **↑↔↓↔↔↔↔↔↔↔↑** |  | **↔↔** | **↔↓↔↓↔↓↔↔** |
| **ActiGraph GT9X** | **↔** | **↔** | **↑↑↔↔↑↔↔↓** |  | **↓↔** | **↑** |  |  | **↔** |
| **Actiheart** |  |  | **↔↔↑↔↔↔↔↑** |  |  | **↓↔** |  |  |  |
| **ActiPed** |  |  | **↔** |  |  |  |  |  |  |
| **ActiReg** |  |  | **↓↔** |  |  |  |  |  |  |
| **Activ4Life Pro** |  |  |  |  |  | **↔** |  |  |  |
| **Actillume recorder** | **↑** |  |  |  |  | **↔** |  |  |  |
| **Activ8** |  | **↔↔↔**^5^ | **↔** |  | **↑↔↔**^5^ | **↓** |  | **↔↔**^5^ |  |
| **ActivPAL** |  | **↔↑↔↑↑↔↑↔↑** | **↓↔↑↔↑↑↑↓↔↔↑↔↓↔↑↔↔↑** |  | **↑↑↑↑↑** | **↔↓↔↑↔↔** |  | **↑** | **↔↑↓↔↔** |
| **ActivPAL micro** |  |  | **↔** |  |  |  |  |  |  |
| **ActivPAL3** |  | **↑↔↑↑** | **↑↑↔↔↑** |  | **↑↑** | **↑↔** |  | **↔↑↔** | **↔↑↔↓** |
| **ActivPAL3 micro** |  |  | **↓** |  |  |  |  |  |  |
| **ActivPAL micro4** |  | **↑** |  |  |  |  |  |  |  |
| **Actimarker** |  |  | **↔↔** |  |  |  |  |  | **↔↔** |
| **Actitrac** | **↔** |  | **↑** |  |  |  | **↔** |  |  |
| **Actiwatch** | **↔** |  | **↔↔** |  |  |  | **↓** |  | **↔** |
| **Actiwatch-2** | **↔↔↔↔↔↔↔↔** |  | **↑↑** | **↑↔↔↓** |  |  |  |  |  |
| **Actiwatch 4** |  |  |  | **↔↔↑** |  |  |  |  |  |
| **Actiwatch 64** | **↔↑↑↔↔↔↔** |  |  | **↔** |  |  | **↔↓** |  |  |
| **Actiwatch-L** | **↔↔** |  |  | **↔** |  |  | **↔** |  |  |
| **Actiwatch Spectrum Pro** | **↔↔↔** |  |  | **↔↑↔↔↔↔** |  |  | **↔** |  |  |
| **A&D 101NFC Activity Monitor** |  |  | **↔** |  |  |  |  |  |  |
| **ADAMO Care Watch** |  |  |  |  |  |  |  |  | **↔** |
| **ADXL05** |  |  | **↔** |  |  |  |  |  |  |
| **ADXL202** |  |  |  |  |  |  |  | **↔** |  |
| **APDM Opal** |  |  | **↔** |  |  |  |  |  |  |
| **Apple Watch** |  |  | **↔↓↔↔↔↑↔↓↔↓↔↓↔↔↑↓↓** |  |  | **↓↑↔↔↔** |  |  |  |
| **Apple Watch 2** | **↔↔** | **↔** | **↔↓** |  |  |  |  |  |  |
| **Apple Watch 3** | **↔** |  |  |  |  |  |  |  |  |
| **Apple Watch 4** |  |  | **↔** |  |  |  |  |  |  |
| **Axivity AX3** | **↔** | **↔↑** | **↑↔** | **↔** | **↔↑** | **↔** |  |  |  |
| **B3** |  |  | **↔** |  |  |  |  |  |  |
| **Basis B1** | **↓** |  | **↔↓↓↔↑** |  |  |  |  |  |  |
| **Basis Peak** |  | **↔** | **↓↔** |  |  |  |  |  |  |
| **Beurer AS80** |  |  | **↓** |  |  |  |  |  |  |
| **BioPatch ZephyrLife** |  |  |  | **↔** |  |  |  |  |  |
| **Biotrainer** |  |  | **↑** |  |  |  |  |  |  |
| **Biotrainer Pro** |  |  | **↔** |  |  |  |  |  |  |
| **BodyMedia FIT** |  |  | **↔↔** |  |  |  |  |  |  |
| **Calorie Counter Select 2** |  |  | **↔** |  |  | **↔** |  |  |  |
| **Caltrac** |  |  | **↓↔↓↑↔↔**^5^ |  |  | **↔**^5^ |  |  | **↔↔**^5^ |
| **CAM** |  | **↑↑** |  |  | **↑** |  |  |  |  |
| **Citizen TW600** |  |  | **↔** |  |  |  |  |  |  |
| **Core Armband Monitor** |  |  | **↔** |  |  |  |  |  |  |
| **DirectLife monitor** |  |  | **↓↔** |  |  |  |  |  |  |
| **Dunlop pedometer** |  |  | **↔** |  |  |  |  |  |  |
| **Dynastream AMP-331** |  |  | **↑↔** |  |  | **↔** |  |  |  |
| **DynaPort ADL** |  |  |  |  | **↔** | **↑** |  |  |  |
| **DynaPort MicroMod** |  |  |  |  |  |  |  |  | **↔** |
| **DynaPort MiniMod** |  |  | **↑** |  | **↔** | **↑** |  | **↔** | **↑** |
| **DynaPort MoveMonitor** |  | **↓ ↔** | **↔↑** |  | **↔** |  |  | **↔↔** |  |
| **Epson Pulsesense** |  |  | **↓** |  |  |  |  |  |  |
| **ePulse Personal Fitness Assistant** |  |  | **↓** |  |  |  |  |  |  |
| **Empatica E4** |  |  |  |  |  |  | **↔** |  |  |
| **Everion** |  |  | **↔** |  |  |  |  |  |  |
| **Fatigue Science Readiband** | **↔** |  |  |  |  |  |  |  |  |
| **Fibion** |  | **↔** | **↔** |  |  |  |  |  |  |
| **Fitbit** | **↔** |  | **↓↔↔** |  |  | **↔** |  |  |  |
| **Fitbit Alta HR** | **↔↔↔** |  | **↔** | **↔↔** |  |  |  |  | **↔** |
| **Fitbit Blaze** |  |  | **↓** |  |  |  |  |  |  |
| **Fitbit Charge HR** | **↓** |  | **↓↔↑↔↓↓↓↔↔↔↔↓↓↔↔↔↑↔↓** |  |  | **↓↑** |  |  | **↓↓↔↓↔** |
| **Fitbit Charge 2** | **↔↔** | **↑** | **↓↔↑↔↓↓↔**^5^ | **↔** |  | **↑↓↔**^5^ |  |  | **↔↓↔**^5^ |
| **Fitbit Charge 3** |  |  | **↑** |  |  |  |  |  |  |
| **Fitbit Flex** | **↔↓** |  | **↔↔↔↓↔↔↑↔↔↔↔↓↔↔↓↓↓** |  |  | **↓↔↓↔↓↓** |  |  | **↓↓↓** |
| **Fitbit Flex 2** |  |  | **↔** |  |  |  |  |  |  |
| **Fitbit Force** |  |  | **↔↔** |  |  |  |  |  | **↔** |
| **Fitbit Inspire** |  |  | **↔** |  |  |  |  |  |  |
| **Fitbit Ionic** |  |  | **↔** |  |  |  |  |  |  |
| **Fitbit One** |  |  | **↓↔↑↑↔↔↑↑↔↔↑↑↑↓↓** | **↔** |  | **↔↔↔↓↑↑↔** |  |  | **↔↑↔↑↑** |
| **Fitbit Surge** |  |  | **↓↔↓↔↓↔↔↔↔↓↓** |  |  | **↓** |  |  | **↔** |
| **Fitbit Surge 2** |  |  | **↔** |  |  |  |  |  |  |
| **Fitbit Ultra** |  |  | **↔↓↓** |  |  | **↔↔↔** |  |  | **↓** |
| **Fitbit Versa** |  |  |  |  |  | **↓** |  |  |  |
| **Fitbit Zip** |  |  | **↔↑↑↑↔↑↔↓↓↔↔↑** |  |  | **↔↓↔** |  |  | **↔↔↔↔↔↑** |
| **Flyfit** |  |  | **↓** |  |  |  |  |  |  |
| **Freestyle Pacer Pro** |  |  | **↔↔** |  |  |  |  |  |  |
| **FS-750** | **↑** |  |  |  |  |  |  |  |  |
| **G-Sensor 2026** |  |  |  |  |  |  |  |  | **↓** |
| **Gaehwiler Electronic** | **↑** |  |  | **↔** |  |  |  |  |  |
| **Garmin 235** |  |  | **↑** |  |  |  |  |  |  |
| **Garmin Fenix 3** |  |  | **↓** |  |  |  |  |  |  |
| **Garmin Fenix 5S** | **↔** |  |  |  |  |  |  |  |  |
| **Garmin Forerunner 225** |  |  | **↓** |  |  |  |  |  |  |
| **Garmin Forerunner 405CX** |  |  |  |  |  | **↔** |  |  |  |
| **Garmin Forerunner 735XT** |  |  |  |  |  | **↔** |  |  |  |
| **Garmin Forerunner 920XT** |  |  | **↓↔↑** |  |  |  |  |  |  |
| **Garmin Vovoactive** |  |  | **↔** |  |  |  |  |  | **↑** |
| **Garmin Vovoactive 4s** |  |  |  |  |  |  |  |  | **↑** |
| **Garmin Vivofit 1** |  |  | **↔↔↓↔↑↔↑↔↔↓↔↓↔↓** |  |  | **↓↑↔** |  |  | **↓↓** |
| **Garmin Vivofit 2** |  |  | **↔↔↑↑** |  |  | **↔** |  |  | **↓↔** |
| **Garmin Vivofit 3** |  |  | **↔↔** |  |  |  |  |  |  |
| **Garmin Vivomove HR** |  |  | **↔** |  |  |  |  |  |  |
| **Garmin Vivosmart** |  |  | **↔↔↔↔↓↔↔** |  |  |  |  |  | **↔↔** |
| **Garmin Vivosmart HR+** |  |  | **↑↓↑↑↓** |  |  |  |  |  | **↔** |
| **Garmin Vivosmart 3** | **↔** |  |  |  |  |  |  |  |  |
| **Garmin Vivosport** |  |  |  |  |  |  |  |  | **↑** |
| **GENEActiv** | **↔↔** | **↑↔↑** | **↑↔↓↔↑↔** | **↔↔** | **↑** | **↑** |  | **↑** |  |
| **Geonaute ONStep 400** |  |  |  |  |  | **↑** |  |  |  |
| **Gopher FITStep Pro** |  |  | **↔** |  |  |  |  |  |  |
| **Health Patch** |  | **↔** | **↓** |  |  |  |  |  |  |
| **Hidalgo EQ02** |  |  | **↓** |  |  |  |  |  |  |
| **HR + M instrument** |  |  | **↑** |  |  |  |  |  |  |
| **Huami Arc** |  |  |  | **↔** |  |  |  |  |  |
| **IC Sensor Model 3031** |  | **↔** |  |  |  |  |  |  |  |
| **IDEEA** |  | **↑↑** | **↑↔↔↔** |  | **↑↔** | **↔** |  |  | **↑** |
| **iHealth activity monitor** |  |  | **↓↓** |  |  |  |  |  |  |
| **Ikcal** |  |  | **↔** |  |  |  |  |  |  |
| **Jabra Sport Pulse Wireless Earbuds** |  |  | **↓** |  |  |  |  |  |  |
| **Jawbone UP** |  |  | **↑↓↔↔↓↔↔↓↓** | **↔↔** |  | **↔** |  |  |  |
| **Jawbone UP2** |  |  | **↔** | **↔** |  | **↑** |  |  | **↓** |
| **Jawbone UP3** | **↔** |  | **↓↔↔** | **↔↓** |  |  |  |  | **↔↓** |
| **Jawbone UP24** |  |  | **↔↑↓↔↓** |  |  | **↓↔↓** |  |  | **↔** |
| **Jawbone UP Move** |  |  | **↓↓** |  |  |  |  |  | **↓** |
| **Kellog’s Special K** |  |  | **↓** |  |  |  |  |  |  |
| **Kenz e-Style2** |  |  | **↔** |  |  |  |  |  |  |
| **Kenz Lifecorder EX** |  |  | **↑↔↑↑↔↑↓↔↔↔↑** |  |  |  |  |  | **↔↔↔** |
| **Kenz select 2** |  |  | **↓** |  |  |  |  |  |  |
| **Leaf Health Tracker** |  |  | **↑↑↑** |  |  |  |  |  |  |
| **Lifecorder PLUS** | **↔** |  |  |  |  |  |  |  |  |
| **Liefsource XL-18** |  |  |  |  |  | **↔** |  |  |  |
| **LIS3LV02DQ** |  |  |  |  |  | **↑** |  |  |  |
| **Lumoback** |  |  | **↑** |  |  | **↓** |  |  |  |
| **Metria-IH1** |  |  | **↔↔↑** |  |  | **↔↔** |  |  |  |
| **Microsoft Band** |  |  | **↑↓↓↓↓** |  |  |  |  |  | **↓** |
| **Microsoft Band 2** |  |  | **↔** |  |  |  |  |  |  |
| **Mio FUSE** |  |  | **↔** |  |  |  |  |  |  |
| **Mio Slice** |  |  |  |  |  | **↓** |  |  |  |
| **Misfit Flash** |  |  | **↔** |  |  |  |  |  |  |
| **Misfit Shine** |  |  | **↔↓↑↑↔↔↔↓↔** |  |  |  |  |  | **↓** |
| **Motionlogger Microwatch** | **↔↔** |  |  |  |  |  |  |  |  |
| **MotionSense HRV** |  |  | **↔** |  |  |  |  |  |  |
| **MotionWare 8** | **↔** |  |  |  |  |  |  |  |  |
| **Mother** |  |  | **↔** |  |  |  |  |  |  |
| **Moto 360** |  |  | **↑** |  |  |  |  |  |  |
| **MovBand Model 2** |  |  | **↑** |  |  |  |  |  |  |
| **MOVEBAND** |  |  |  |  |  | **↓** |  |  |  |
| **Move II** |  |  | **↔** |  |  | **↔** |  |  |  |
| **Move 3** | **↔** |  |  |  |  |  |  |  |  |
| **Move 4** |  | **↑** |  |  |  |  |  |  |  |
| **MOX Activity Logger** |  |  |  |  |  |  |  | **↑↑** | **↑** |
| **Multi-sensor board** |  | **↑** | **↑** |  |  |  |  |  |  |
| **Muvone** |  |  |  |  |  | **↑** |  |  |  |
| **Mymo Tracker** |  |  |  |  |  |  |  |  |  |
| **myCanadian watch** | **↔** |  |  |  |  |  |  |  |  |
| **My Wellness Key** |  |  | **↑↑** |  |  |  |  |  |  |
| **New Lifestyle NL-800** |  |  |  |  |  | **↑** |  |  |  |
| **New Lifestyle NL-1000** |  |  | **↔↔↑↓↑** |  |  |  |  |  | **↑** |
| **New Lifestyle NL-2000** |  |  | **↔↑↔↑↑↑↑↑↔** |  |  | **↔** |  |  | **↔↑↓** |
| **Nike + Fuelband** |  |  | **↓↔↓↔↓↓↓↔↔↔↓↔** |  |  | **↓** |  |  |  |
| **Nokia Go** |  |  | **↔↔** |  |  |  |  |  |  |
| **Omron Active Style Pro** |  |  | **↓↑** |  |  |  |  |  | **↔** |
| **Omron B1** |  |  |  |  |  | **↑** |  |  |  |
| **Omron CaloriScan** |  |  | **↓** |  |  |  |  |  |  |
| **Omron HF-100** |  |  | **↔↔** |  |  | **↔** |  |  | **↔** |
| **Omron HJ-105** |  |  | **↓↔↑** |  |  | **↔** |  |  |  |
| **Omron HJ-109** |  |  | **↔** |  |  |  |  |  |  |
| **Omron HJ-112** |  |  | **↑↑↔** |  |  | **↑** |  |  | **↔** |
| **Omron HJ-113** |  |  | **↔** |  |  | **↑** |  |  | **↔** |
| **Omron HJ-151** |  |  | **↑↑** |  |  |  |  |  |  |
| **Omron HJ-203** |  |  | **↑↔** |  |  |  |  |  |  |
| **Omron HJ-301** |  |  | **↔** |  |  |  |  |  |  |
| **Omron HJ-303** |  |  | **↔↑** |  |  |  |  |  |  |
| **Omron HJ-304** |  |  | **↔** |  |  |  |  |  |  |
| **Omron HJ-320** |  |  | **↔** |  |  |  |  |  |  |
| **Omron HJ-321** |  |  | **↔↑↑** |  |  |  |  |  | **↔** |
| **Omron HJ-322** |  |  | **↔** |  |  |  |  |  |  |
| **Omron HJ-350IT** |  |  | **↓↔** |  |  | **↔** |  |  |  |
| **Omron HJ-700IT** |  |  | **↓↓** |  |  |  |  |  |  |
| **Omron HJ-720IT** |  |  | **↔↓↑↔↑↑↑↔↑↔↑↔** |  |  | **↔↔** |  |  | **↑↔↑** |
| **Omron HJA-306** |  |  | **↔** |  |  |  |  |  |  |
| **Omron Pro HJA-750C** |  |  | **↑** |  |  |  |  |  | **↔** |
| **ONStep 400** |  |  |  |  |  |  |  |  | **↓** |
| **Oregon Scientific PE316CA** |  |  | **↓↔** |  |  |  |  |  |  |
| **Össur patient activity monitor** |  |  |  |  |  | **↔** |  |  |  |
| **Oura Ring** | **↔** |  |  |  |  |  |  |  |  |
| **PADIS 2.0** |  |  | **↔** |  |  |  |  |  |  |
| **PALlite** |  |  | **↑** |  |  |  |  |  |  |
| **PALlite 3c** |  |  | **↑** |  |  |  |  |  |  |
| **Pebble Steel** |  |  | **↔↔↔** |  |  |  |  |  |  |
| **Personal Activity Monitor** |  |  | **↔** |  |  |  |  |  |  |
| **Philips Health Watch** |  |  |  |  | **↑** | **↑** |  |  | **↔** |
| **Physilog** |  | **↑** | **↑**^5^ |  |  | **↑**^5^ |  |  | **↑**^5^ |
| **PiezoRx** |  |  | **↑** |  |  | **↑** |  |  |  |
| **Piezo Step MV** |  |  |  |  |  |  |  |  | **↓** |
| **Polar A300** | **↑** |  | **↔** |  |  |  |  |  |  |
| **Polar A360** |  |  | **↔↓↔↓↓** |  |  |  |  |  |  |
| **Polar F6** |  |  | **↓** |  |  |  |  |  |  |
| **Polar H7** |  |  | **↓** |  |  |  |  |  | **↔** |
| **Polar HR monitor** |  |  | **↔** |  |  |  |  |  |  |
| **Polar Loop** |  |  | **↓↔↔↓↔** |  |  | **↔** |  |  |  |
| **Polar M200** |  |  | **↔** |  |  |  |  |  |  |
| **Polar M400** |  |  |  |  |  |  |  |  | **↔** |
| **Polar S410** |  |  | **↔** |  |  |  |  |  |  |
| **Polar S810i** |  |  | **↔** |  |  |  |  |  |  |
| **Polar V800** |  |  | **↔↔** |  |  |  |  |  |  |
| **Polar Vantage M** |  |  | **↔** |  |  |  |  |  | **↔** |
| **Polar Vantage XL** |  |  | **↓** |  |  |  |  |  |  |
| **Positional Activity Logger 2** |  |  |  |  |  |  |  | **↑** |  |
| **Prosthetic Activity Monitor** |  |  |  |  | **↔** | **↑** |  |  |  |
| **PulseOn** |  |  | **↓↔** |  |  |  |  |  |  |
| **Qualcomm Toq** |  |  | **↑** |  |  |  |  |  |  |
| **Quattrolter** |  |  |  |  | **↑** |  |  |  |  |
| **R3D** |  |  | **↔** |  |  |  |  |  |  |
| **RT6** |  |  | **↑** |  |  |  |  |  |  |
| **RT3** |  |  | **↔↑↔↔↓↔** |  |  |  |  |  | **↔** |
| **Samsung Galaxy Watch Active** |  |  | **↔** |  |  |  |  |  |  |
| **Samsung Gear 1** |  |  | **↔** |  |  |  |  |  |  |
| **Samsung Gear 2** |  |  | **↔↔↔** |  |  | **↔** |  |  |  |
| **Samsung Gear Fit** |  |  | **↔** |  |  |  |  |  |  |
| **Samsung Gear Fit2** |  |  | **↔↔↔** | **↓** |  |  |  |  |  |
| **Samsung Gear S** |  |  | **↔↔** |  | **↑** | **↑** |  |  |  |
| **Sartorio Xelometer** |  |  | **↔** |  |  |  |  |  |  |
| **SENS motion** |  |  |  |  |  |  |  | **↔** |  |
| **SenseWear Pro** |  |  | **↔↓↓↔↔↔↔↑↓↓↑↔↔↔↔↔↔↔↔↔↑↓↔** | **↔↑** |  | **↔↑↔↑↔↔↓↑** |  |  | **↔↔↑↔↑↓** |
| **SenseWear Pro 2** |  |  | **↓↔↑↓** |  |  | **↓↓↓** |  |  | **↔** |
| **SenseWear Pro 3** | **↔** |  | **↓↔↔↓↔↑↔↓↓↔↓** | **↔** |  | **↓↔** |  |  | **↑↓↔** |
| **SenseWear Mini** |  |  | **↓↓↑↓↓↓↔↔↑↔↓** |  |  | **↔** |  |  | **↔↓** |
| **Silva pedometer** |  |  | **↓** |  |  |  |  |  |  |
| **SmartHealth** |  |  | **↓** |  |  |  |  |  |  |
| **SmartLAB walk+** |  |  | **↔** |  |  |  |  |  |  |
| **SOMNOwatch** |  |  |  | **↑↔** |  |  |  |  |  |
| **Sony SmartBand** |  |  | **↔** |  |  |  |  |  |  |
| **Spire Activity Tracker** |  |  | **↓** |  |  |  |  |  |  |
| **Sportline** |  |  | **↑** |  |  |  |  |  |  |
| **Sportline 330** |  |  | **↓↓↓** |  |  | **↓** |  |  |  |
| **Sportline 340 Strider** |  |  | **↔** |  |  |  |  |  |  |
| **Sportline 345** |  |  | **↔↓** |  |  |  |  |  |  |
| **Sportline Traq** |  |  | **↔** |  |  |  |  |  |  |
| **SportBrain iStep X1** |  |  | **↓** |  |  |  |  |  |  |
| **Step-Keeper HSB-SKM** |  |  | **↔** |  |  | **↔** |  |  | **↔** |
| **Stepcount StepMX** |  |  | **↑** |  |  |  |  |  | **↑** |
| **Step-N-Tune** |  |  |  |  |  | **↔** |  |  |  |
| **Stepwatch** |  |  | **↑↑↑↔↑↔↑↑↑** |  |  | **↑↑↑↑↑** |  |  | **↑↑↑↔↑↑** |
| **Stepwatch 3** |  |  | **↑↑↑↑↔↔** |  |  |  |  |  | **↑↑** |
| **Stryd Power Meter** |  |  | **↑** |  |  |  |  |  |  |
| **Suunto Ambit2** |  |  | **↓** |  |  |  |  |  |  |
| **Suunto HR** |  |  | **↔** |  |  |  |  |  |  |
| **Suunto Sport** |  |  | **↔** |  |  |  |  |  |  |
| **Suunto Trainer** |  |  | **↑** |  |  |  |  |  |  |
| **Tanita AM-160** |  |  | **↔** |  |  |  |  |  |  |
| **Tanita Calorism Smart** |  |  | **↔** |  |  |  |  |  |  |
| **Tom Tom Cardio** |  |  | **↔↓↓** |  |  |  |  |  |  |
| **Tom Tom Touch** |  |  | **↓↔** |  |  |  |  |  |  |
| **Tom Tom Spark 3** |  |  | **↔** |  |  |  |  |  |  |
| **Tanita FB727** |  |  | **↔** |  |  |  |  |  |  |
| **THIM** | **↔** |  |  |  |  |  |  |  |  |
| **Tracmor** |  |  | **↑↑** |  |  |  |  |  |  |
| **Tractivity** |  |  | **↔** |  |  |  |  |  |  |
| **T-REX** |  |  |  | **↑** |  |  |  |  |  |
| **Tritrac-R3D** |  |  | **↑↔↔↔** |  |  |  |  |  | **↔** |
| **Tritrac T303A** |  |  | **↔** |  |  |  |  |  |  |
| **USB accelerometer X16-mini** |  |  |  |  | **↑** |  |  | **↑** |  |
| **Verisense IMU** |  |  | **↔** |  |  |  |  |  |  |
| **Vibe Actigraph** |  |  |  |  |  |  | **↔** |  |  |
| **Vitabit** |  | **↔** |  |  |  |  |  |  |  |
| **Vivago** |  |  | **↑** |  |  |  |  |  |  |
| **Vivago WristCare** |  |  |  | **↔** |  |  |  |  |  |
| **Walk4Life Elite** |  |  | **↓↑** |  |  |  |  |  | **↔** |
| **Walk4Life LS 2500** |  |  | **↔** |  |  | **↔** |  |  | **↔** |
| **Walk4Life LS 2525** |  |  | **↔↑** |  |  |  |  |  |  |
| **Walk4Life LS 7010** |  |  | **↓** |  |  |  |  |  |  |
| **Walk4Life Pro** |  |  | **↔** |  |  |  |  |  |  |
| **Walking Style X** |  |  |  |  |  | **↓** |  |  |  |
| **Watch_PAT100** |  |  |  | **↔** |  |  |  |  |  |
| **WHOOP Strap** | **↔↔** |  |  |  |  |  |  |  |  |
| **WIMU Pro** |  |  | **↑**^5^ |  |  | **↑**^5^ |  |  | **↑**^5^ |
| **Withings Pulse O2** |  |  | **↑↓↑↑↔↔↓↓** | **↔** |  |  |  |  |  |
| **Withings Pulse Ox** |  |  | **↓↔↔** |  |  |  |  |  | **↔** |
| **Withings Steel** |  |  | **↔** |  |  |  |  |  |  |
| **Xiaomi Mi Band** |  |  | **↔↔** |  |  |  |  |  |  |
| **Xiaomi Mi Band 2** | **↔** |  | **↔↑↔** | **↓** |  |  |  |  | **↑** |
| **Xiaomi Mi Band 3** | **↔** |  | **↔** |  |  |  |  |  | **↑** |
| **Xiaomi Mi Band 4** |  |  |  |  |  |  |  |  | **↑** |
| **Yamax Pedometer** |  |  | **↑** |  |  |  |  |  |  |
| **Yamax Digiwalker** |  |  | **↑↔↔** |  |  |  |  |  | **↔** |
| **Yamax Digiwalker CW-700** |  |  | **↑** |  |  | **↓** |  |  | **↔** |
| **Yamax Digiwalker DW-200** |  |  | **↔↔↔↑↔↔** |  |  | **↔** |  |  | **↔** |
| **Yamax Digiwalker DW-351** |  |  | **↓** |  |  |  |  |  |  |
| **Yamax Digiwalker DW-800** |  |  | **↑** |  |  |  |  |  |  |
| **Yamax Digiwalker SW-200** |  |  | **↔↔↔↔↑↔↔↔↓↔↔↔↔↔** |  |  | **↔↔↔↔↔↔↔** |  |  | **↔↓↔↓↔↔↔** |
| **Yamax Digiwalker SW-401** |  |  | **↔** |  |  | **↔** |  |  |  |
| **Yamax Digiwalker SW-500** |  |  | **↑** |  |  | **↑** |  |  |  |
| **Yamax Digiwalker SW-501** |  |  | **↓** |  |  |  |  |  |  |
| **Yamax Digiwalker SW-700** |  |  | **↔↔** |  |  | **↔** |  |  |  |
| **Yamax Digiwalker SW-701** |  |  | **↔↔↑↑↔↔↔↓** |  |  | **↔↓** |  |  | **↔↓** |
| **Yamax EX510** |  |  |  |  |  | **↑** |  |  | **↔** |
| **Yamax EX700** |  |  | **↔** |  |  |  |  |  |  |
| **Yamasa Skeletone** |  |  | **↔** |  |  |  |  |  |  |
| **Yamax Skeletone EM-180** |  |  | **↔** |  |  |  |  |  |  |
| **Z80-32k V1** | **↔** |  |  | **↔** |  |  |  |  |  |
| **Zulu watch** | **↔** |  |  |  |  |  |  |  |  |
| *Notes:*  ^1^ Biological States; ^2^ Posture/Activity Type; ^3^ Intensity; ^4^ Each symbol in a cell represents the findings from a unique study; ^5^ No age-group was reported. **↑**: Moderate to strong validity **↔**: Mixed validity **↓**: Poor/weak validity | | | | | | | | | |
